# Light in a Heartbeat: Bond Scission by a Single Photon above 800 nm

**DOI:** 10.1021/jacs.3c14197

**Published:** 2024-03-18

**Authors:** Marina Russo, Hana Janeková, Debora Meier, Melanie Generali, Peter Štacko

**Affiliations:** † Department of Chemistry, University of Zurich, Wintherthurerstrasse 190, Zurich CH-8057, Switzerland; ‡ Institute for Regenerative Medicine (IREM), University of Zurich, Wagistrasse 12, Zurich CH-8952, Switzerland

## Abstract

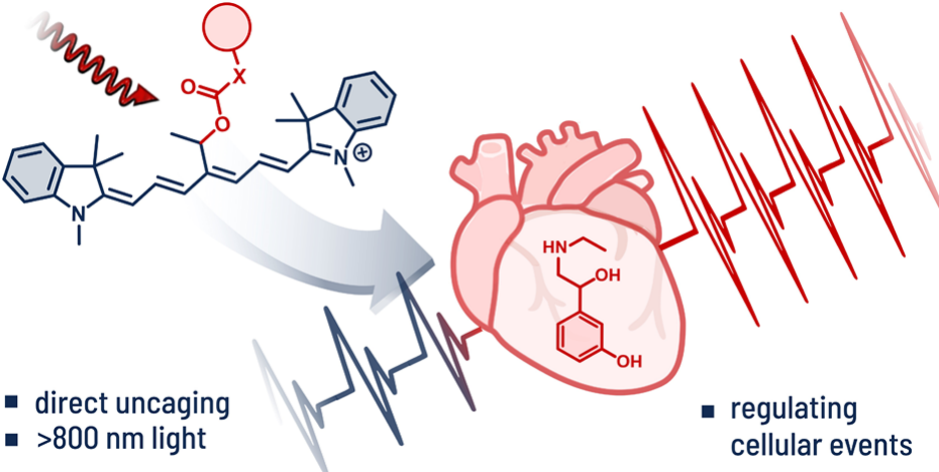

Photocages enable scientists to take full control over the activity of molecules using light as a biocompatible stimulus. Their emerging applications in photoactivated therapies call for efficient uncaging in the near-infrared (NIR) window, which represents a fundamental challenge. Here, we report synthetically accessible cyanine photocages that liberate alcohol, phenol, amine, and thiol payloads upon irradiation with NIR light up to 820 nm in aqueous media. The photocages display a unique chameleon-like behavior and operate via two distinct uncaging mechanisms: photooxidation and heterolytic bond cleavage. The latter process constitutes the first example of a direct bond scission by a single photon ever observed in cyanine dyes or at wavelengths exceeding 800 nm. Modulation of the beating rates of human cardiomyocytes that we achieved by light-actuated release of adrenergic agonist etilefrine at submicromolar concentrations and low NIR light doses (∼12 J cm^–2^) highlights the potential of these photocages in biology and medicine.

## Introduction

Photocages capitalize on the biorthogonality and high spatiotemporal resolution of light as a trigger to seize control over substrate activity.^1^ Exemplified by their success in activating proteins,^2,3^ nucleotides,^4,5^ drugs,^6,7^ and other biologically relevant molecules,^8−10^ photocages have gained significant traction in photoactivated chemotherapy^11−16^ (PACT), an emerging approach complementary to photodynamic therapy (PDT). Yet, to clinically establish photocages alongside PDT, it is critical to shift their absorption into the near-infrared, tissue-transparent window (NIR; 650–900 nm) without compromising the release efficacies.^17,18^

**Figure 1:**
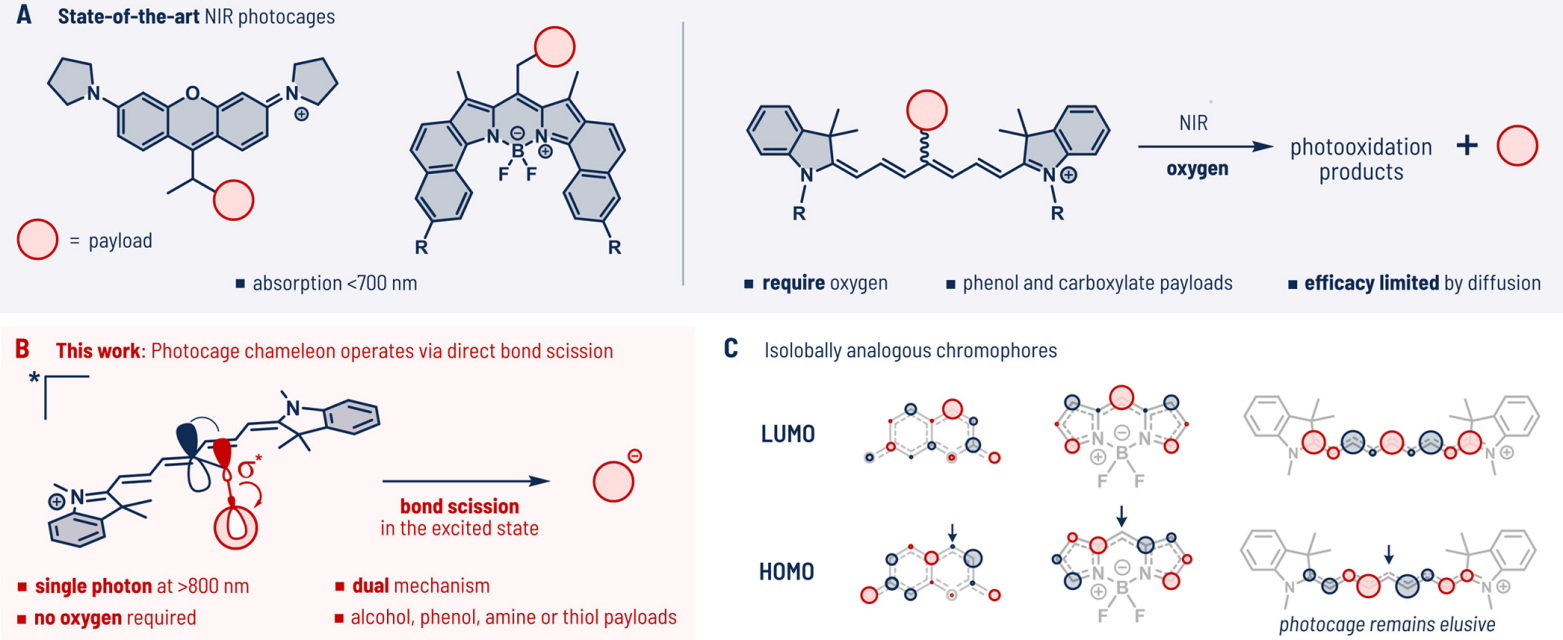
(A) State-of-the-art NIR photocages. (B) The concept of uncaging from Cy7 is based on direct bond scission. (C) Isolobal analogy of known photocages with a hitherto elusive analogous photocage based on the Cy7 scaffold.

Although remarkable advances in the field of photocages have been recorded in the past decades (Figure 1A),^1,19^ the discovery of novel scaffolds for organic photocages has historically relied mostly on a serendipity, and their development remains far from being an accomplished task.^20^ The challenges are even more daunting for NIR photocages as the excitation energy provided by NIR photons is low, excited states are short-lived, and the application demands compatibility with aqueous media.

In 2015, Šolomek et al. postulated a design strategy to predict new photocage scaffolds drawing on the Zimmerman’s effect and, at that time, known photocages.^21^ The concept relies on the accumulation of electron density in the excited state in a position adjacent to the carbon-payload bond (Figure 1C, denoted by arrows). Mixing of the filled orbital with the adjacent antibonding orbital weakens the carbon-payload bond and facilitates departure of the payload. Such shifts of electron density can be predicted by simple Hückel calculations of the frontier molecular orbital (HMO) coefficients. This approach was supported by the emergence of BODIPY-based photocages,^22,23^ which underwent impressive optimization in terms of efficacies and absorption properties.^24−27^ Reports of xanthene-,^28^ porphyrin-,^29^ and, most recently also,^30^ rhodamine-derived photocages emerged, all isolobally analogous chromophores. Yet, analogous photocages derived from heptamethine cyanine (Cy7) dyes that operate via heterolytic bond cleavage from the excited state have remained elusive despite sharing the same orbital topology (Figure 1C).

The Cy7-based uncaging reported to date relies exclusively on ^1^O_2_-mediated fragmentation of the Cy7 scaffold followed by thermal solvolysis of the intermediates to liberate the payloads of only a limited scope (Figure 1A). Schnermann and co-workers harnessed photooxidative cleavage of Cy7 to release phenols through a cascade of reactions.^15,31−33^ Recently, we and the group of Feringa simultaneously reported uncaging of carboxylic acids from Cy7 dyes,^34,35^ which was eventually experimentally confirmed to proceed through a related mechanism, i.e., only in the presence of singlet oxygen (^1^O_2_).^35^ The uncaging efficacies consequently strongly depend on the concentration of both the photocage and oxygen in the system, and the slow thermal steps limit their application in studies of fast processes. Aware of these drawbacks and convinced about the generality of the HMO approach, we leveraged it in Cy7 scaffolds.

Here, we report a family of Cy7 photocages that can release a broad palette of payloads including amines, phenols, alcohols, and thiols with tissue-penetrating NIR light (Figure 1B). Their applicability is illustrated in human cardiomyocytes by light-induced modulation of their beating rate. The photocages exhibit a chameleon-like behavior and operate through two distinct uncaging mechanisms. We finally provide evidence that direct uncaging from Cy7 dyes with a single near-infrared photon at wavelengths >800 nm is a viable strategy. Besides, further reinforcing the generality of the HMO design strategy, this pathway absolves Cy7 photocages of the reliance on oxygen and slow secondary steps and instill them as strong contenders in the pursuit of PACT.

## Results and Discussion

**Scheme 1:**
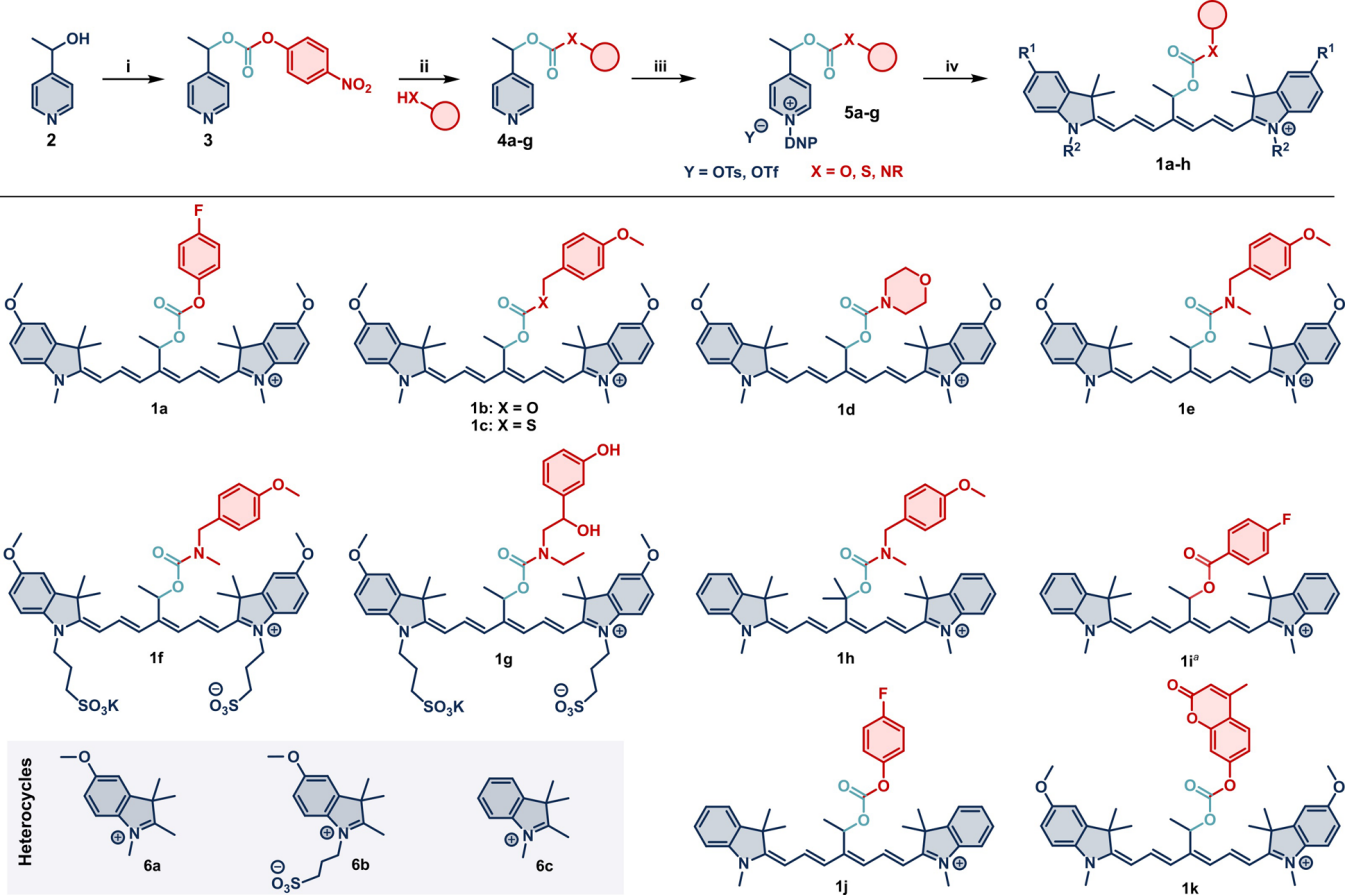
Synthesis of the Photocages (i) DMAP, bis(4-nitrophenyl)carbonate, MeCN. (ii) Amine/alcohol/thiol payload, MeCN or DMSO, 80 °C. (iii) DNP-OTs or DNP-OTf, MeCN, 40 °C. (iv) **6a**–**6c,** AcOK, MeCN, rt. Caged payloads are depicted in red. Counter anions are omitted for clarity. Reported in ref (35).

We synthesized the photocages **1a**–**1h** conveniently in four steps using the Zincke chemistry protocol (Scheme 1).^36^ The starting alcohol **2** was reacted with bis(4-nitrophenyl) carbonate to provide activated carbonate **3** in 85% yield. The payloads were subsequently introduced in excellent yields (up to 89%) by nucleophilic substitution of **3** with the corresponding alcohols, thiol, or amines in the presence of DIPEA or DMAP as auxiliary bases. The reaction of the pyridines **4a**–**4g** with 2,4-dinitrophenyl tosylate or triflate (DNP-OTs or DNP-OTf) in acetonitrile provided the corresponding Zincke salts **5a**–**5g**, which were subsequently transformed into the desired photocages **1a**–**1k** containing diverse payloads in a one-pot reaction with the terminal heterocycles **6a**–**6c** and AcOK as a base. Carbamate-containing photocages **1e**–**1h** were observed by ^1^H NMR spectroscopy as mixtures of two rotamers, which coalesced into a single spectrum at an elevated temperature. In contrast, carbamate analogues derived from primary amines remained elusive, likely due to abstraction of the N–H under the mildly basic reaction conditions.

Photocages **1a**–**1k** in PBS (pH 7.4, 10 mM, 20% DMSO) display a strong absorption located in the center of the NIR region at λ_max_ = 809–818 nm, matching the emission of commercial diode lasers and the isosbestic point of the hemoglobin-deoxyhemoglobin system (∼810 nm).^37^ The compounds exhibit weak emission (Φ_F_ < 0.02) with Stokes shifts of ∼500 cm^–1^, comparable to similar red-shifted cyanines.^38^ While **1f** and **1g** are fully soluble in aqueous media, some derivatives showed propensity to aggregate (*c* > 10^–5^ M) depending on lipophilicity of the payload. DMSO was therefore employed as a cosolvent in UV–vis studies to facilitate direct comparison between derivatives **1a**–**1h**.

**Figure 2:**
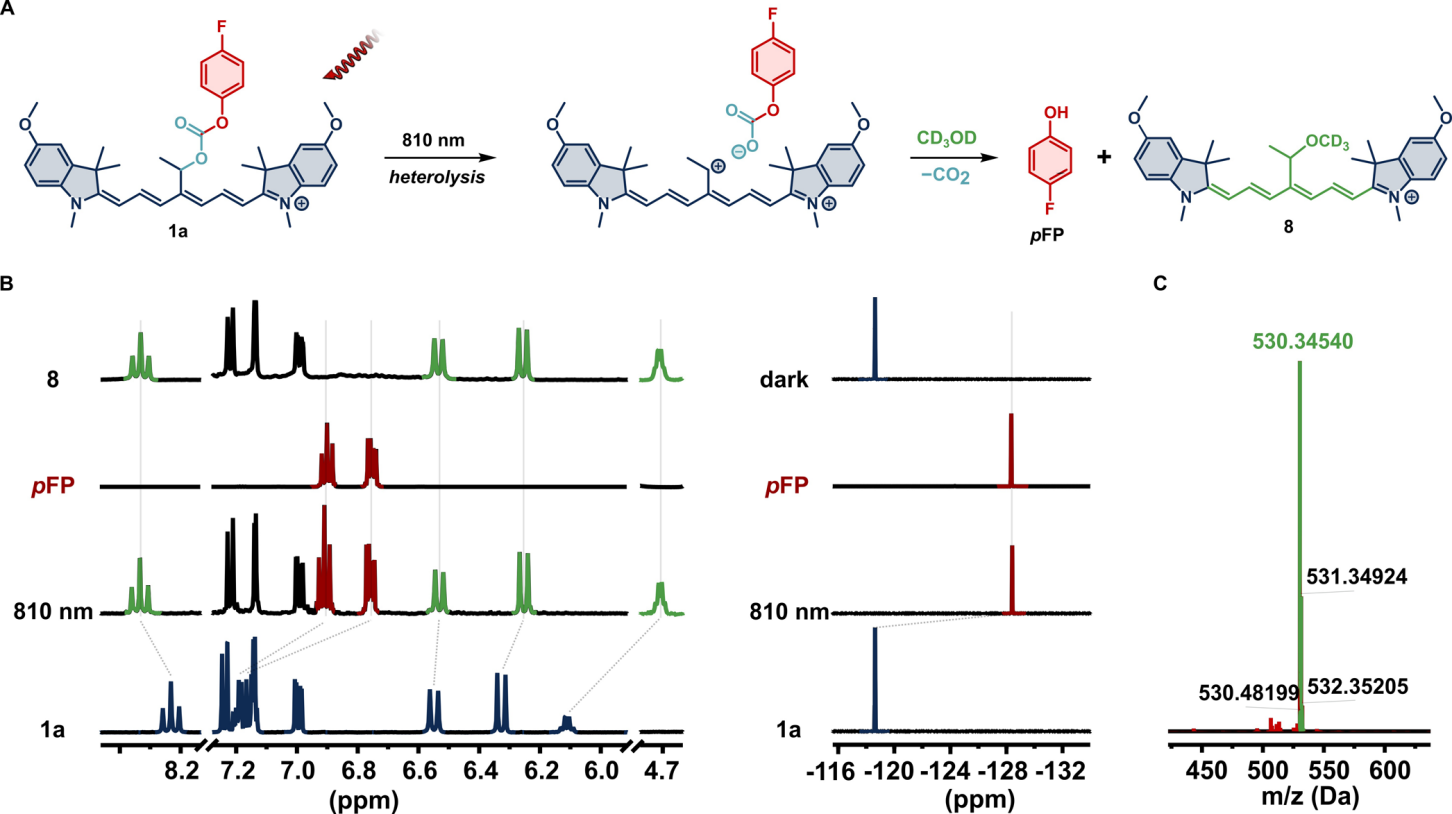
(A) Photouncaging of *p***FP** payload from **1a** via the direct pathway including a cationic intermediate, which is trapped by the solvent to give **8**. (B) ^1^H (left) and ^19^F (right) NMR spectra of **1a**, **1a** irradiated at 810 nm in oxygen-free conditions, *p***FP** for 44 h, and the photoproduct **8** (from bottom to top). (C) ESI-HRMS of the identical sample of **1a** irradiated at 810 nm in the absence of oxygen showing the clean formation of **8**.

**Scheme 2:**
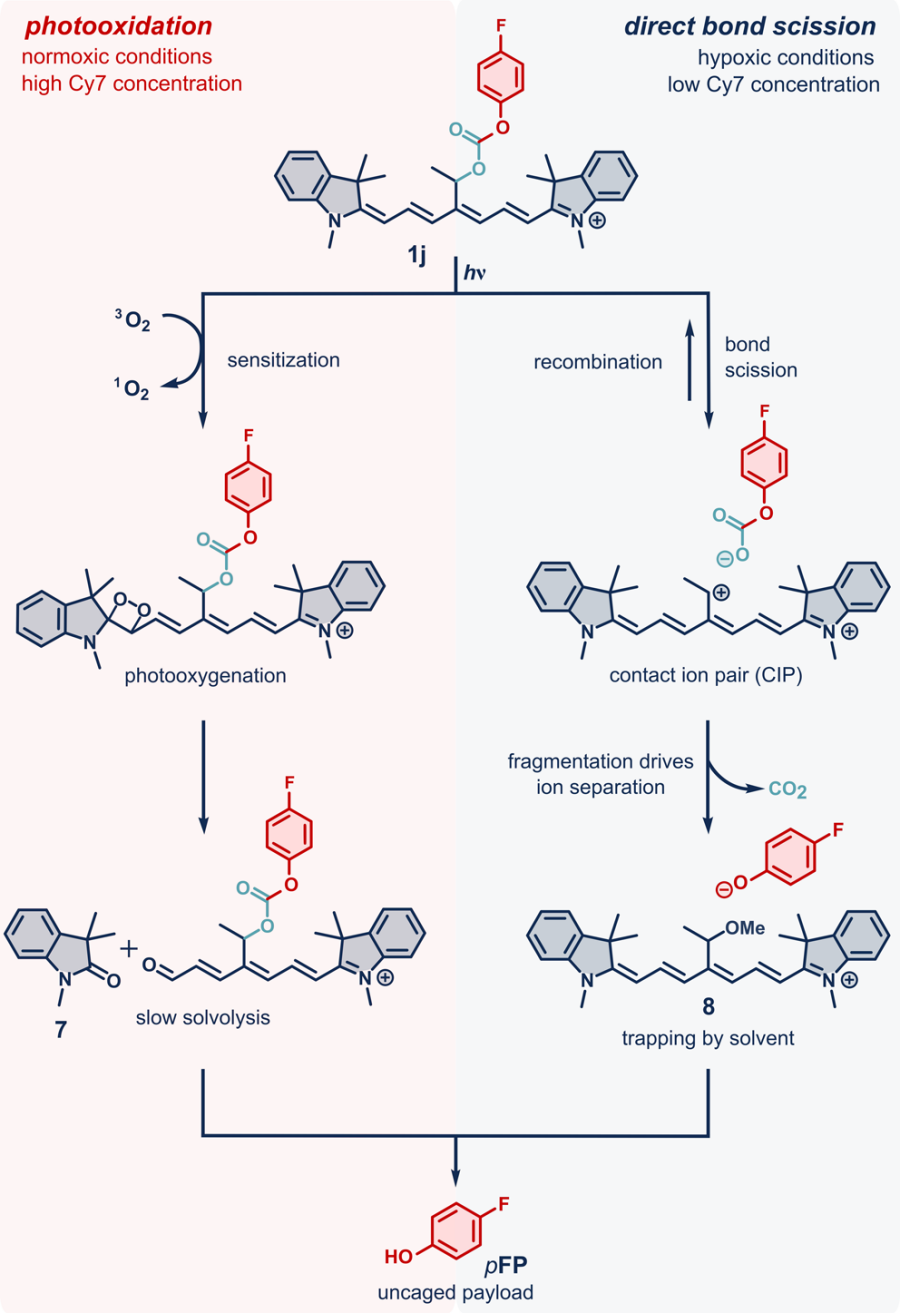
Suggested Photooxidative (Left) and Direct (Right) Uncaging Mechanisms That Operate in Cy7 Photocages

Irradiation of **1a** in CD_3_OD at 810 nm (∼300 mW cm^–2^) for 24 h under ambient conditions led to the complete loss of the green color typical for Cy7 dyes accompanied by liberation of the 4-fluorophenol (*p***FP**) payload observed by ^1^H and ^19^F NMR (Figure S27). Complete destruction of the Cy7 scaffold and the production of ketone **7** (*vide supra*Scheme 2) were consistent with the previously reported **1i** operating via the ^1^O_2_-mediated uncaging mechanism.^39,40^ In strong contrast, irradiation of **1a** with NIR light under the exclusion of oxygen leads to its clean conversion into a new Cy7 species with concurrent uncaging of *p***FP** as evidenced by ^1^H and ^19^F NMR spectroscopies (Figure 2A,B, depicted in green and red, respectively). The signal of the proton at the reaction center experiences a significant upfield shift from 5.98 to 4.59 ppm, consistent with the presence of a less electron-deficient residue in this position, i.e., a formal substitution of the payload by methanol. The anticipated structure **8** that originates from trapping the putative carbocation by CD_3_OD was further supported by HRMS (Figure 2C). To unequivocally establish its identity, we synthesized **8** independently and confirmed that its ^1^H NMR and UV–vis spectra perfectly match those produced by the irradiation of **1a**. We observed uncaging under oxygen-free conditions also for other payload functionalities (Table 1, e.g., **1b**, **1c**, and **1e**), wheareas no uncaging or formation of **8** was observed for the control samples kept in the dark (Figures S27–S36). These results are in strong contrast to the behavior of previously reported **1i** and provide compelling evidence that the payload from **1a** is released by a direct C–O bond scission, with intermediary formation of a carbocation at the cyanine residue. Both **1a** and its *des*-methoxy analogue **1j** release *p***FP** when irradiated under oxygen-free conditions with comparable efficiencies of 91 and 93%, respectively, demonstrating that the electron-donating substituents are not instrumental to the direct uncaging mechanism (Figures S44 and S45). The quantum yield of the direct uncaging pathway (Φ_het_) from **1a** in CD_3_OD was determined to be (6.8 ± 0.7) × 10^–4^, which is comparable to the BODIPY photocages that operate at wavelengths blue-shifted by ∼300 nm,^23^ and approximately an order of magnitude higher than the first generation of their red-shifted analogues.^24^ The uncaging cross section of **1a** (εΦ ∼ 80 M^–1^ cm^–1^) sits comfortably in the range desired for biological application.^19^

**Table 1:** Photophysical Properties of the Selected Photocages

photocage	λ_abs_/nm^a^	λ_em_/nm^b^	ε^a^*^,^*^c^	Φ_ox_ × 10^5^^a^^,^^d^	yield_aer_/%^e^^,^^g^	yield_deg_/%^f^^,^^g^
**1a**	809	843	117 300	4.5 ± 0.1	47 ± 2	88 ± 1 (100)
**1b**	809	839	120 900	3.4 ± 0.3	44 ± 2	92 ± 4 (46)
**1c**	811	843	94 200	5.8 ± 0.9	63 ± 3	103 ± 3 (100)
**1d**	808	844	115 300	2.2 ± 0.2	40 ± 7	n.d.
**1e**	810	847	100 400	2.7 ± 0.2	44 ± 2	67 ± 8 (26)
**1f**	818	854	117 800	2.3 ± 0.2	48 ± 2	n.d.
**1g**	816	856	85 000	2.5 ± 0.1	55 ± 5	n.d.
**1h**^b^	808	836	44 600	0.6 ± 0.1	41 ± 3	n.d.

a Determined in PBS (pH 7.4, 10 mM) with 20% DMSO.

b Determined in methanol.

c Molar absorption coefficient, ε_max_/mol^–1^ dm^3^ cm^–1^.

d Absolute quantum yields of photooxidative decomposition.

e Chemical yield of the payload uncaging with 820 nm light in CD_3_OD under ambient conditions.

f Chemical yield of the payload uncaging with 810 nm light in CD_3_OD under oxygen-free conditions, corrected for the conversion (in parentheses).

g Determined by ^1^H NMR spectroscopy. Average and standard deviations of the mean from at least three independent samples are given. n.d. stands for not determined.

**Figure 3:**
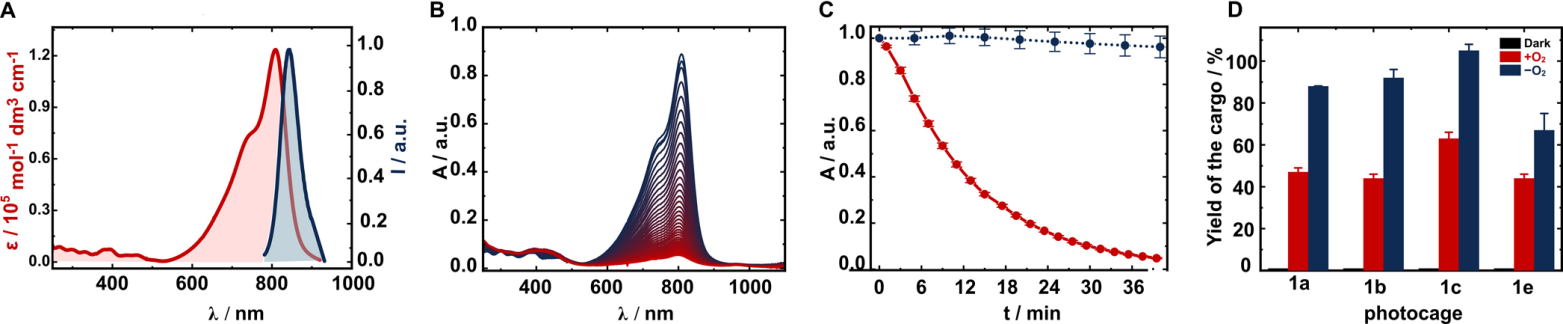
(A) UV–vis absorption (red) and emission (blue) spectra of **1a** in PBS and methanol, respectively. (B) Irradiation of **1a** in PBS at 820 nm followed by UV–vis spectroscopy every 30 s (blue to red) under ambient conditions. (C) Kinetic trace of the absorbance at 809 nm upon irradiation of **1a** at 820 nm (red) and in the dark (blue) under ambient conditions. (D) Chemical yields of the payloads uncaged from selected photocages in the dark (black), upon irradiation at 780 nm under ambient (red) and oxygen-free (blue) conditions.

We subsequently followed uncaging by UV–vis absorption spectroscopy (Figure 3A–C). Irradiation of **1a**–**1h** in PBS (pH 7.4, 10 mM, 20% DMSO) at 820 nm (∼40 mW cm^–2^) for 20–40 min under ambient conditions was accompanied by depletion of the cyanine absorption band, and in the case of **1a**–**1c**, a concurrent minor blue-shift (∼3–9 nm) of the absorption maxima (see Figures S11–S13). The former is attributed to photooxidation of the cyanine scaffold,^35,40^ whereas the latter suggests a concomitant formation of a new cyanine species that bears a less electron-accepting residue in the C4′ position, i.e., **8**.^38^ As anticipated, the photoxidation process was inhibited under oxygen-free conditions in both MeOH and PBS, but a blue-shift was observed, indicating that oxygen is not vital for the latter process (Figures S22–S26). LC-HRMS analysis of these samples detected the expected photoproduct formed by trapping the putative carbocation by MeOH or water. The quantum yields of the photooxidation (Φ_ox_) process summarized in Table 1 are comparable to other Cy7 dyes.^38^

Uncaging of the fluorogenic 4-methylumbelliferone payload from **1k** in aqueous media (PBS/DMSO, 4:1) was then followed by emission spectroscopy (Figures S19–S21). Under ambient conditions, the payload was uncaged at a rate comparable to the depletion of the cyanine band, without the obviously slow, thermal component previously observed for carboxylates.^35^ Removal of oxygen by purging with Ar (5 min) significantly suppressed the cyanine band depletion (∼14-fold), accompanied by a blue shift of the absorption maxima (8 nm). On the other hand, the rate of uncaging remained relatively fast (3-fold slower) and majority of the payload was still uncaged in the same time frame. The rates of uncaging in these two samples cannot be directly compared because the latter will be slower not only due to suppression of the photooxidation pathway but also because of the inner filter effect of the cyanine photoproduct. However, these results clearly demonstrate that the direct uncaging pathway also operates to a significant degree in aqueous media.

The uncaging from **1a**–**1h** under ambient conditions proceeds with a chemical yield of 40–60%, whereas the yield is significantly higher under oxygen-free conditions and nearly quantitative for **1a**–**c** as determined by ^1^H NMR spectroscopy (Table 1, Figure 3D). We hypothesized that the lower yield of uncaging **1e** in oxygen-free conditions could be due to the released amine payload attacking the putative carbocation, but we found no evidence of such species by LC-HRMS or ^1^H NMR spectroscopy (Figure S37). No detectable release was observed in the samples kept in the dark, except for **1h**, which exhibits a compromised solvolytic stability (∼4% uncaging after 24 h). On the other hand, photocages **1a** and **1e** displayed excellent stability also in aqueous media (H_2_O/MeCN, 9:1) and no trace of the uncaged payloads was observed by UPLC after incubation for 4 h in the dark (Figure S49). Interestingly, thiol was liberated from **1c** as the corresponding disulfide, likely due to secondary oxidation by ROS or via an electron transfer to Cy7. However, we do not expect this to prevent the application of thiol-releasing Cy7 photocages in a cellular environment as endogenous levels of gluthathione reduce disulfides to thiols.^41^

Next, we evaluated the toxicity of **1f** and **1g** and their corresponding photoproducts produced by exhaustive irradiation in cell viability assays on HeLa cells. The compounds exhibited no toxicity after 72 h of exposure at concentrations up to 50 μM (see Figures S52–S53). Their cell-permeable analogues are likely somewhat more toxic as evidenced in the literature.^35,42,43^

**Figure 4:**
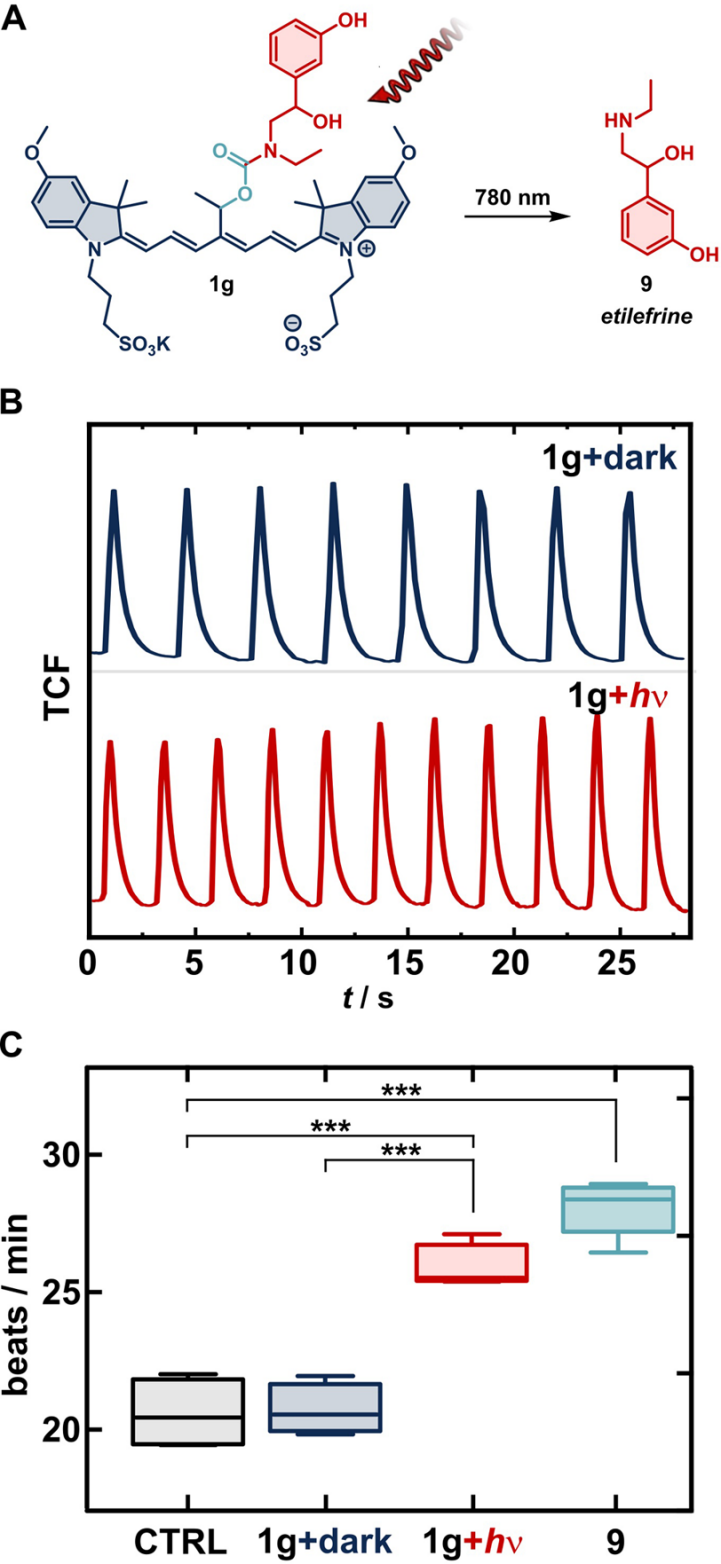
(A) Uncaging of etilefrine **9** from photocage **1g** with light at 780 nm. (B) Representative traces of the total cell fluorescence (TCF) of beating iPSC-derived cardiomyocytes loaded with calcium probe Fluo-8AM and **1g** (800 nM) kept in the dark (top) or irradiated at 780 nm (∼40 mW, 5 min, bottom). (C) Observed beating rates of the cardiomyocytes incubated with **1g** (800 nM) in the dark (blue) or irradiated with NIR light (red) or with etilefrine (400 nM, cyan) and the control (black). Median, IQR, and 5–95% percentiles are shown.

Guided by the ambitions of photocages in biomedical sciences, we sought to demonstrate their applicability in controlling biological processes that extend beyond just cellular death. We therefore synthesized caged etilefrine derivative **1g** (Figure 4A). Etilefrine **9**, an adrenergic agonist of primarily α_1_ and β_1_ receptors, is a cardiac stimulant that is approved as a hypotensive drug.^44^ We tested **1g** and its light-induced activity on spontaneously beating iPSC-derived cardiomyocytes, which were differentiated using a commercial kit and allowed to form a syncytium. The beating frequency under different conditions was quantified using an established protocol using calcium flux fluorescence imaging by Fluo-8AM (Figure 4B, see the Supporting Information for details).^45,46^ The use of a higher concentration of **1g** (800 nM) in comparison to the free etilefrine (400 nM) in control experiments compensated for the moderate chemical yield of uncaging of **9** from **1g** (Figure 3D, Table 1). The cardiomyocytes incubated with **1g** and subsequently irradiated with NIR light (780 nm, ∼40 mW per well for 5 min) displayed a significant increase in the beating frequency compared to both those kept in the dark and the control experiments (Figure 4C). The magnitude of the increase was comparable to that elicited by free etilefrine, whereas cardiomyocytes incubated with **1g** in the dark showed a beating frequency virtually identical to that of the control experiment. Similar effects were observed also in the HL-1 mouse cardiac muscle cell line. While we expect that etilefrine is uncaged for **1g** predominantly through photooxidation, we cannot exclude that part of it is uncaged via the direct uncaging pathway, especially given the low concentration of **1g**.

Contrary to the recent example using the BODIPY scaffold,^47^ Cy7-caged etilefrine **1g** showed no residual activity, suggesting that the charged and bulkier Cy7 scaffold is more efficient at blocking the binding compared to the BODIPY residue. In contrast, we achieved modulation of the beating frequency by direct irradiation of the cardiomyocytes with NIR light instead of irradiating the photocage prior to its incubation with the cells. Our results reaffirm the capacity of Cy7 photocages to directly manipulate delicate biological processes at submicromolar concentration by activation of drug-like molecules containing multiple functional groups at low NIR light doses (∼12 J cm^–2^), comparable to use in PDT.

## Mechanistic Considerations

The presented experimental evidence shows that these photocages can release payloads by two orthogonal mechanisms: self-sensitized photooxidation and direct uncaging through a cationic intermediate (Scheme 2). Since the former is a bimolecular reaction and its Φ_ox_ depends on the concentration of Cy7, whereas the Φ_het_ of the latter does not, one can find a concentration at which these quantum yields of the competing mechanisms are equal. Below this concentration (or at increasingly hypoxic conditions), the uncaging is dominated by the direct uncaging pathway, whereas above this concentration, the majority of the payload is released via the ^1^O_2_-mediated mechanism. We estimated this limit for **1a** in aqueous media to *c* ∼ 1–2 × 10^–4^ M (see the Supporting Information). Indeed, at sufficiently low starting concentrations of **1a** or **1e** (*c* ∼ 2 × 10^–4^ M in MeOH/H_2_O), we observed the photoproducts of the direct uncaging, **8**, its analogue formed by trapping with water, and the subsequent products of oxidation/elimination even in samples irradiated under ambient conditions (HRMS, Figures S41–S43).

Substituents on the heterocycles and anions are omitted for the sake of clarity.

Yet, the contrasting efficacies of the direct uncaging between (thio)carbonates (**1a**–**1c**) and the previously reported carboxylate^35^ (**1i**) payloads are puzzling. Since the direct uncaging from the latter cannot be observed, we estimate that it must be at least two orders of magnitude less efficient than for **1a**. However, such a drastic difference for comparable payloads (p*K*_a_s within 2–3 units) extends far beyond the Bro̷nsted dependence of Φ_rel_ that has been traditionally observed for other photocages.^48^ For reference, the Φ_rel_ difference of carbonate pairs in BODIPY^26^ and coumarin^48−50^ photocages suggests that alternative factors operate here.

We reason that the efficacy of the direct uncaging pathway from Cy7 photocages is governed by the ability of the formed contact ion pair (CIP) to undergo separation before its recombination back to the starting photocage and not by the activation barrier (Δ*G*^‡^) of the bond scission in the excited state (Scheme 2). In the case of **1a**–**1h** and **1j** and **1k**, the loss of CO_2_ from the liberated anions provides a pathway to perturb the solvent cage around the CIP to drive its escape, whereas no such option likely exists for the carboxylate payload in **1i**. While the rate of ion recombination is known to be high in coumarin photocages (*k*_rec_ ∼ 10^9^ s^–1^),^51^ it appears that its suppression is crucial for the function of Cy7 photocages. Recently, Winter and co-workers showed that the intramolecular trapping of the cation can increase the efficacy BODIPY photocages, reinforcing our reasoning.^25^ High significance of the recombination process might also suggest that direct uncaging occurs predominantly from the S_1_ excited state.

Hence, we reasoned that the direct uncaging pathway should be accelerated by promoting CIP separation, e.g., by increasing the ionic strength. We tested the hypothesis on **1i** containing a carboxylate payload that is not released in the absence of oxygen, i.e., via the direct uncaging pathway.^35^ Indeed, increasing the ionic strength by the addition of LiCl (100 mM) elicited direct, albeit very slow, uncaging of the previously latent carboxylate payload (∼13% after 46 h of irradiation at 810 nm). Like in the case of **1a**, the process was accompanied by the formation of a cyanine corresponding to the putative cation trapped by the solvent, as evidenced by NMR and HRMS spectroscopies (see Figures S38–S40). Such a “turn-on” behavior of the direct uncaging pathway by high ionic strength strongly supports the notion that recombination of the produced CIP, instead of the photodissociation step, is indeed the efficacy-limiting step for Cy7 photocages and perhaps for other red-shifted scaffolds (BODIPY, rhodamine) as well. Recently, Feringa and co-workers showed that the efficacy of photocages can be increased by carbocation stabilization.^52^ While the observed enhancements are impressive, this approach is destined to reach its limits in the context of hydrolytic stability as these parameters are invariably linked. We believe that targeting the recombination pathway, e.g., via geometric reorganization or cascade reactions of the cationic intermediates, provides an alternative productive avenue to empower NIR photocages.

## Conclusions

In summary, we report cyanine photocages that are synthetically accessible and liberate alcohol, phenol, amine, and thiol payloads upon irradiation with NIR light up to 820 nm in aqueous media. We showcase their biological utility through modulation of beating rates of iPSC-derived cardiomyocytes by the NIR light-actuated release of adrenergic agonist etilefrine at low light doses. The photocages exhibit a chameleon-like behavior by operating via two orthogonal uncaging mechanisms. The direct uncaging pathway observed here represents the first example of payload liberation from cyanine dyes, which is not reliant on oxygen. On a fundamental level, these observations constitute the piece of the puzzle that cements the previously postulated photocage design theory based on simple Hückel calculations as a generally applicable strategy across all chromophore families. We believe that the valuable lessons learned herein will guide the rational design of NIR photocages and spur the efforts to combat the nonproductive recombination pathways. Indeed, ongoing experiments in our laboratory suggest that strategies targeting the CIP separation significantly increase the photouncaging efficacies and will be reported in due course.

## Supporting Information Available

Materials, experimental procedures and methods, synthesis, photophysical and photochemical measurements; methodology of biological experiments, NMR spectroscopy, UV-vis absorption and emission spectroscopy, plots of photophysical and photochemical measurements, irradiation setups, biological experiments (PDF)

## Dedication

Dedicated to our friend, professor Petr Klán, on the occasion of his sixtieth birthday.
