# Supplementary material for: Light in a Heartbeat: Bond Scission by a Single Photon above 800 nm
Source: J Am Chem Soc. 2024 Mar 18;146(12):8417–24. doi: 10.1021/jacs.3c14197 (PMC10979397; doi:10.1021/jacs.3c14197)
Supplement: Supplementary file 1 — ja3c14197_si_001.pdf [file ja3c14197_si_001.pdf]

Supporting information

Light in a Heartbeat: Bond Scission by a Single Photon Above 800 nm

Marina Russo,[1,‡] Hana Janeková,[1,‡] Debora Meier,[2] Melanie Generali,[2] Peter Štacko[1,*]

[1] Department of Chemistry, University of Zurich, Wintherthurerstrasse 190, CH-8057 Zurich, Switzerland
[2] Institute for Regenerative Medicine (IREM), University of Zurich, Wagistrasse 12, CH-8952 Zurich, Switzerland

[‡]*These authors contributed equally*

Table of Contents

Author Contributions	S2
Materials and Methods	S2
Synthesis	S2
Photophysical and Photochemical Measurements Methodology	S12
Methodology of Biological Experiments	S15
NMR Spectroscopy	S17
UV-Vis Absorption and Emission Spectroscopy	S62
Plots of Photophysical and Photochemical Measurements	S66
Irradiation Setups	S85
Biological experiments	S86
References	S88

Author Contributions

P.Š., M.R, and H.J. synthesized the intermediates and final photocages. M.R. and H.J. performed the photophysico-chemical characterization of the photocages, H.J. performed the mechanistic study and M.R. performed the biological studies on cardiomyocytes. D.M. and M.G. differentiated and provided the iPSC-derived cardiomyocytes. P.Š., M.R, and H.J. conceived the project, designed the experiments, analyzed the data, and co-wrote the manuscript.

Materials and Methods

Reagents and solvents of the highest purity available were used as purchased, or they were purified/dried using standard methods when necessary. The intermediates **6a-c**[1,2,3]**, 12, 13a-b**[1]**, 14**[4]**,** were synthesized according to the published procedures or purchased from standard suppliers (Merck, TCI, Across Organics, etc.).

Flash column chromatography was performed using silica gel (230–400 mesh). ^{1}H NMR spectra were recorded on 400 or 500 MHz spectrometers; ^{13}C NMR spectra were obtained on 125 MHz instruments in $CDCl_3$, CD_3OD, and d_6-DMSO. ^{19}F NMR were obtained on 376 MHz or 470 MHz instruments. ^{1}H chemical shifts are reported in ppm relative to $CDCl_3$ (δ = 7.26 ppm), CD_3OD (δ = 3.31 ppm) and d_6-DMSO (δ = 2.50 ppm) as an internal reference. ^{13}C chemical shifts are reported in ppm with $CDCl_3$ (δ = 77.67 ppm), CD_3OD (δ = 49.30 ppm) and d_6-DMSO (δ = 39.52 ppm) as internal references. ^{19}F NMR chemical shifts are reported in ppm either without internal standard, or in case of irradiation experiments using C_6F_6 (δ = –165.35 ppm) in a sealed capillary as an internal reference. Deuterated solvents were kept under nitrogen atmosphere.

Absorption spectra and molar absorption coefficients were obtained on a UV-vis spectrometer with matched 1.0-cm quartz cells. Fluorescence and excitation spectra were measured using a fluorescence spectrometer in a 1.0 cm quartz fluorescence cuvette at 20 °C. The sample concentrations were adjusted to keep the absorbance below 0.2 at the corresponding excitation wavelength. Each sample was measured five times, and the spectra were averaged. Emission and excitation spectra were normalized and corrected by the photomultiplier sensitivity function using correction files supplied by the manufacturer.

The exact masses of the synthesized compounds were obtained using a triple quadrupole electrospray ionization mass spectrometer in a positive or negative mode coupled with direct-inlet.

Synthesis of the Intermediates and Photocages

4-Nitrophenyl (1-(pyridin-4-yl)ethyl) carbonate (3)

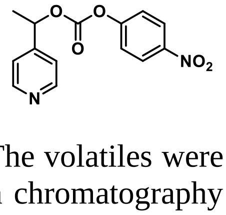

The alcohol **2** (1 g, 8.12 mmol) and bis(4-nitrophenyl)carbonate (3.7 g, 12.2 mmol) were dissolved in anhydrous MeCN (20 mL) and DIPEA (4.03 mL, 3.15 g, 24.4 mmol) was added at once. The solution turned yellow, and it was left stirring under N_2 atmosphere at room temperature for 16 h. EtOAc (30 mL) was added, and the mixture was extracted with water (2×30 mL). The volatiles were evaporated under reduced pressure and the residue was purified by column chromatography (SiO_2, gradient EtOAc/pentane 1:4 to 1:1) to afford the product. Yield: 2.0 g (85%). Orange solid. M.p. 84.1–84.4 °C. ^{1}H NMR (500 MHz, *d*-$CDCl_3$) δ (ppm) 8.65 (d, *J* = 4.9 Hz, 2H), 8.27 (d, *J* = 9.1 Hz, 2H), 7.37 (d, *J* = 9.1 Hz, 2H), 7.33 (d, *J* = 5.0 Hz, 2H), 5.80 (q, *J* = 6.7 Hz, 1H),

1.69 (d, *J* = 6.6 Hz, 3H). ^{13}C NMR (126 MHz, *d*-$CDCl_3$) δ (ppm) 155.5, 151.9, 150.5, 149, 145.6, 125.5, 121.8, 120.7, 76.5, 22.1. HRMS (ESI+) calcd. for [$C_{14}H_{13}N_2O_5^+$] 289.0824, found 289.0814.

Synthesis of 4-fluorophenyl (1-(pyridin-4-yl)ethyl) carbonate (4a)

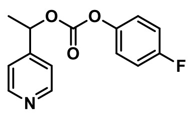

Mixed carbonate **3** (400 mg, 1.39 mmol) was mixed with 4-fluorophenol (**10a**) (187 mg, 1.67 mmol) in anhydrous MeCN (2 mL) and kept under N_2 atmosphere. DIPEA (460 uL, 360 mg, 2.78 mmol) was added and the reaction mixture was stirred under N_2 atmosphere at 40 °C for 16 h. Water (30 ml) was added, and the mixture was extracted with EtOAc (2×25 mL), the combined organic phase was dried with $MgSO_4$, and volatiles were evaporated under reduced pressure. Crude product was purified by column chromatography (SiO_2, EtOAc/pentane 1:1) to afford the product. Yield: 323 mg (89%). Colorless liquid. ^{1}H NMR (500 MHz, *d*-$CDCl_3$) δ (ppm) 8.64 (d, *J* = 5.0 Hz, 2H), 7.32 (d, *J* = 4.7 Hz, 2H), 7.17–7.10 (m, 2H), 7.10 – 7.01 (m, 2H), 5.77 (q, *J* = 6.7 Hz, 1H), 1.67 (d, *J* = 6.3 Hz, 3H). ^{13}C NMR (126 MHz, *d*-$CDCl_3$) δ (ppm) 160.5 (d, *J* = 245.0 Hz), 153.0, 150.3, 149.6, 146.95 (d, *J* = 2.9 Hz), 122.6 (d, *J* = 8.5 Hz), 120.7, 116.3 (d, *J* = 23.6 Hz), 75.9, 22.1. ^{19}F NMR (470 MHz, *d*-$CDCl_3$) δ (ppm) -116.26. HRMS (ESI+) calcd. for [$C_{14}H_{13}FNO_3^+$] 262.0879, found 262.0884.

Synthesis of 4-methoxybenzyl (1-(pyridin-4-yl)ethyl) carbonate (4b)

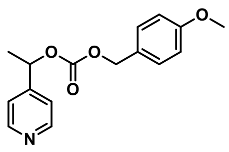

Mixed carbonate **3** (300 mg, 1.04 mmol) was mixed with 4-methoxyphenylmethanol (**10b**) (172 mg, 1.25 mmol) and DMAP (127 mg, 1.04 mmol) in anhydrous MeCN (5 mL) and kept under N_2 atmosphere. DIPEA (344 uL, 270 mg, 2.08 mmol) was added and the reaction mixture was stirred under N_2 atmosphere at 60 °C for 16 h. Water (30 mL) was added, and the mixture was extracted with EtOAc (2×25 mL), the combined organic phase was dried with $MgSO_4$ and volatiles were evaporated under reduced pressure. Crude product was purified by column chromatography (SiO_2, EtOAc/pentane 1:1) to afford the product. Colorless liquid. Yield: 262 mg (88%). ^{1}H NMR (400 MHz, *d*-$CDCl_3$) δ (ppm) 8.60 (dd, J_1 = 1.6 Hz, J_2 = 4.5 Hz, 2H), 7.31 (dd, J_1 = 2.1 Hz, J_2 = 6.6 Hz, 2H), 7.27–7.25 (m, 2H), 6.89 (dd, J_1 = 2.2 Hz, J_2 = 6.6 Hz, 2H), 5.69 (q, *J* = 6.7 Hz, 1H), 5.14–5.04 (dd, J_1 = 11.7 Hz, J_2 = 18.8 Hz, 2H), 3.81 (s, 3H), 1.57 (d, *J* = 6.7 Hz, 3H). ^{13}C NMR (126 MHz, *d*-$CDCl_3$) δ (ppm) 160.1, 154.5, 150.3, 150.1, 130.5, 127.2, 120.7, 114.2, 74.8, 70.0, 55.5, 22.2. HRMS (ESI+) calcd. for [$C_{16}H_{18}NO_4^+$] 288.1236, found 288.1235.

Synthesis of S-(4-methoxybenzyl) O-(1-(pyridin-4-yl)ethyl) carbonothioate (4c)

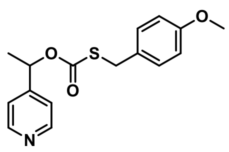

Mixed carbonate **3** (300 mg, 1.04 mmol) was mixed with DMAP (381 mg, 3.12 mmol) in anhydrous MeCN (9 mL) and kept under N_2 atmosphere. A solution of 4-methoxyphenylmethanethiol (**10c**) (160 µL, 176 mg, 1.14 mmol) in anhydrous MeCN (18 mL) was added and the reaction mixture was stirred under N_2 atmosphere at 40 °C for 3.5 h. Volatiles were evaporated, EtOAc (20 mL) was added, and mixture was extracted with water (5×30 mL). The combined organic phase was dried with $MgSO_4$, and volatiles were evaporated under reduced pressure. The crude product was purified by column chromatography (SiO_2, EtOAc/pentane 1:3) to afford the product. Colorless liquid. Yield: 230 mg (73%). ^{1}H NMR (500 MHz, *d*-$CDCl_3$) δ (ppm) 8.59 (d, *J* = 4.8 Hz, 2H), 7.24–7.22 (m, 4H), 6.83 (d, *J* = 8.1 Hz, 2H), 5.91 (q, *J* = 6.7 Hz, 1H), 4.06 (dd, J_1 = 13.7 Hz, J_2 = 22.3 Hz, 2H), 3.79 (s, 3H), 1.56 (d, *J* = 6.5 Hz, 3H). ^{13}C NMR (126 MHz, *d*-$CDCl_3$) δ (ppm) 170.5, 159.2, 150.2, 150.1, 130.2, 128.9, 120.7, 114.2, 74.1, 55.4, 35.1, 22.2. HRMS (ESI+) calcd. For [$C_{16}H_{18}NO_3S^+$] 304.1002, found 304.1001.

General Procedure for Synthesis of Carbamates 4d-g

Mixed carbonate **3** (1 eq), amine (2 eq) and DIPEA (3 eq) were dissolved in anhydrous DMF (1 mL/mmol) and stirred under N_2 atmosphere. The reaction mixture was stirred at room temperature for 2 h, quenched with water (5 mL/mmol) and extracted with EtOAc (3×7 mL/mmol). Combined organic phase was washed with water (3×4 mL/mmol), dried with $MgSO_4$ and volatiles were evaporated under reduced pressure. Crude product was purified by column chromatography (SiO_2, gradient EtOAc/pentane 1:1 to 1:0) to afford the product.

1-(Pyridin-4-yl)ethyl morpholine-4-carboxylate (4d)

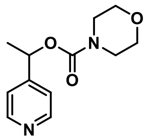

Prepared according to the general procedure from mixed carbonate **3** (200 mg, 0.7 mmol) morpholine (**10d**) (121 µL, 121 mg, 1.39 mmol) and DIPEA (344 µL, 270 mg, 2 mmol). Yield: 100 mg (61%). Colorless liquid. ^{1}H NMR (500 MHz, *d*$_4$-CD_3OD) δ (ppm) 8.56 (d, *J* = 5.1 Hz, 2H), 7.49 (d, *J* = 5.3 Hz, 2H), 5.81 (q, *J* = 6.7 Hz, 1H), 3.67 (s, 4H), 3.62 (s, 2H), 3.44 (s, 2H), 1.57 (d, *J* = 6.7 Hz, 3H).^{13}C NMR (126 MHz, *d*$_4$-CD_3OD) δ (ppm) 155.9, 155.2, 149.7, 122.5, 73.5, 67.6, 45.6, 22.5. HRMS (ESI+) calcd. for [$C_{12}H_{17}N_2O_3^+$] 237.1239, found 237.1241.

1-(Pyridin-4-yl)ethyl (4-methoxybenzyl)(methyl)carbamate (4e)

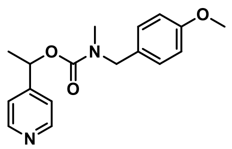

Prepared according to the general procedure from mixed carbonate **3** (250 mg, 0.87 mmol) *N*-methyl-4-methoxybenzylamine (**10e**) (262 mg, 1.73 mmol) and DIPEA (430 µL, 336 mg, 2.6 mmol). Yield: 208 mg (80%). Colorless liquid. Mixture of rotamers. ^{1}H NMR (500 MHz, *d*$_4$-CD_3OD) δ (ppm) 8.54–8.48 (m, 2H), 7.45 (s, 1H), 7.44 (s 1H), 7.19 (d, *J* = 11.5 Hz, 2H), 6.91 (d, *J* = 11.5 Hz, 2H), 5.81 (q, *J* = 6.8 Hz, 1H), 4.45 (s, 1H), 4.41 (s, 1H), 3.83–3.77 (m, 3H), 2.96–2.91 (m, 3H), 1.57 (s, 3H). ^{13}C NMR (126 MHz, *d*$_4$-CD_3OD) δ (ppm) 160.7, 154.4, 150.3, 130.1, 129.7, 122.3, 122.2, 115.1, 73.5, 55.7, 52.8, 35.0, 33.8, 22.64, 22.57. HRMS (ESI+) calcd. for [$C_{17}H_{21}N_2O_3^+$] 301.1552, found 301.1548.

Synthesis of 1-(pyridin-4-yl)ethyl ethyl(2-hydroxy-2-(3-hydroxyphenyl)ethyl)carbamate (4g)

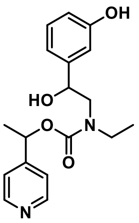

Mixed carbonate **3** (500 mg, 1.73 mmol), etilefrine hydrochloride (**9**) (452 mg, 2.1 mmol) and DIPEA (0.4 ml, 300 mg, 2.6 mmol) were dissolved in anhydrous DMSO (2.5 mL) and stirred under N_2 atmosphere at room temperature for 2 h. DMSO was subsequently removed under vacuum, and the crude product was purified by column chromatography (SiO_2, EtOAc) to afford the product. Yield 500 mg (88%). Colorless liquid. Mixture of two rotamers. ^{1}H NMR (500 MHz, *d*-$CDCl_3$) δ (ppm) 8.62–8.25 (m, 2H), 7.26–6.65 (m, 6H), 5.91–5.48 (m, 1H), 5.02–4.63 (m, 1H), 3.63–3.15 (m, 3H), 1.55–1.34 (m, 3H), 1.17–0.98 (m, 3H). ^{13}C NMR (126 MHz, *d*-$CDCl_3$) δ (ppm) 157.3, 157.1, 157.0, 156.8, 155.3, 155.2, 152.3, 152.2, 152.1, 149.5, 149.4, 149.2, 144.1, 143.9, 143.8, 129.9, 129.7, 121.0, 120.8, 117.4, 115.3, 115.2, 115.1, 113.4, 113.2, 113.1, 73.4, 73.1, 73.0, 72.6, 72.3, 72.2, 72.0, 71.7, 55.6, 55.5, 53.9, 50.7, 44.0, 43.8, 43.7, 43.3, 40.8, 22.37, 22.35, 13.7, 12.9. HRMS (ESI+) calcd. for [$C_{18}H_{23}N_2O_4$] 331.1658, found 331.1648.

4-Methyl-2-oxo-2H-chromen-7-yl (1-(pyridin-4-yl)ethyl) carbonate (4i)

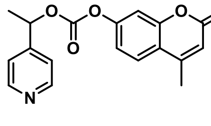

Mixed carbonate **3** (300 mg, 1.39 mmol) was mixed with 7-hydoxy-4-methylcoumarin (220 mg, 1.25 mmol) in anhydrous MeCN (2 mL) and kept under N_2 atmosphere. DIPEA (334 uL, 269 mg, 2.08 mmol) was added and the reaction mixture was stirred under N_2 atmosphere at 40 °C

for 16 h. Water (30 ml) was added, and the mixture was extracted with EtOAc (2×25 mL), the combined organic phase was dried with $MgSO_4$, and volatiles were evaporated under reduced pressure. Crude product was purified by column chromatography (SiO_2, EtOAc/pentane 1:1) to afford the product. Yield: 323 mg (89%). Colorless liquid. ^1H NMR (500 MHz, *d*-$CDCl_3$) δ (ppm) 8.64 (d, *J* = 5.9 Hz, 2H), 7.60 (d, *J* = 8.7 Hz, 1H), 7.32 (d, *J* = 6.0 Hz, 2H), 7.21 (d, *J* = 2.3 Hz, 1H), 7.14 (dd, *J* = 8.7, 2.4 Hz, 1H), 6.29 – 6.25 (m, 1H), 5.80 (q, *J* = 6.6 Hz, 1H), 2.42 (s, 3H), 1.68 (d, *J* = 6.6 Hz, 3H). ^{13}C NMR (126 MHz, *d*-$CDCl_3$) δ (ppm) 160.4, 154.3, 153.2, 152.2, 151.7, 150.5, 149.1, 125.7, 120.7, 118.2, 117.4, 114.9, 110.0, 76.3, 22.1, 18.9. HRMS (ESI+) calcd. for [$C_{18}H_{16}NO_5^+$] 326.1023, found 326.1024.

Synthesis of Carbamate 4h.

Synthesis of 4-nitrophenyl (2-(pyridine-4-yl)propan-2-yl) carbonate (11)

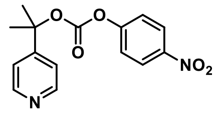

4-Pyridylpropan-2-ol (**12**) (600 mg, 4.37 mmol) was dissolved in anhydrous THF (36 mL) under N_2 atmosph ere and cooled with ice bath. Solution of *n*BuLi in *n*-hexane (3.43 ml, 1.4 M, 4.81 mmol) was added and the mixture was stirred in ice bath for 5 minutes. 4-Nitrophenyl chloroformate (**12**) (1.32 g, 6.56 mmol) was dissolved in anhydrous THF (6 mL) and added dropwise to the prepared alcoholate under N_2 atmosphere in ice bath. The mixture was stirred at room temperature for 16 h before quenching with water (15 mL). Mixture was extracted with EtOAc (2×60 mL) and the combined organic layer was dried with $MgSO_4$. Volatiles were evaporated under reduced pressure and the residue was purified by column chromatography (SiO_2, gradient EtOAc/pentane 1:4 to 1:2) to afford the product. Colorless liquid. Yield: 644 mg (49%). ^1H NMR (500 MHz, *d*-$CDCl_3$) δ (ppm) 8.64 (d, *J* = 4.8 Hz, 2H), 8.24 (d, *J* = 8.6 Hz, 2H), 7.36–7.31 (m, 4H), 1.88 (s, 6H). ^{13}C NMR (126 MHz, *d*-$CDCl_3$) δ (ppm) 155.5, 153.1, 150.4, 150.4, 145.5, 125.4, 121.9, 119.5, 84.3, 27.8. HRMS (ESI+) calcd. for [$C_{15}H_{15}N_2O_5^+$] 303.0981, found 303.0976.

Synthesis of 2-(pyridin-4-yl)propan-2-yl (4-methoxybenzyl)(methyl)carbamate (4h)

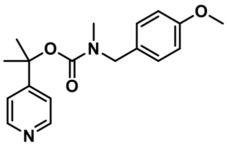

Mixed carbonate **11** (400 mg, 1.32 mmol) was mixed with DMAP (484 mg, 3.96 mmol) in anhydrous MeCN (12 mL) and kept under N_2 atmosphere. A solution of *N*-methyl-4-methoxybenzylamine (10**e**) (218 µL, 220 mg, 1.45 mmol) in anhydrous MeCN (8 mL) was added and the reaction mixture was stirred under N_2 atmosphere at 40 °C for 3.5 h. Volatiles were evaporated, EtOAc (20 mL) was added, and mixture was washed with water (5×30 mL). Organic phase was dried with $MgSO_4$ and volatiles were evaporated under reduced pressure. Crude product was purified by column chromatography (SiO_2, gradient EtOAc/pentane 1:2 to 1:4) to afford the product. Colorless liquid. Mixture of rotamers. Yield: 302 mg (73%). ^1H NMR (500 MHz, *d*-$CDCl_3$) δ (ppm) 8.55 (d, *J* = 16.6 Hz, 2H), 7.26–7.09 (m, 4H), 6.92–6.84 (m, 2H), 4.47–4.32 (m, 2H), 3.83–3.80 (m, 3H), 2.89–2.83 (m, 3H), 1.77 – 1.75 (m, 6H). ^{13}C NMR (126 MHz, *d*-$CDCl_3$) δ (ppm) 159.2, 155.9, 155.7, 155.2, 154.8, 149.9, 129.9, 129.8, 129.4, 128.7, 119.6, 114.2, 114.1, 79.9, 55.5, 52.3, 51.7, 34.5, 33.8, 28.7. HRMS (ESI+) calcd. for [$C_{18}H_{23}N_2O_3^+$] 315.1709, found 315.1708.

General Procedure for Synthesis of Zincke salts 5a–h.

The corresponding carbonate or carbamate **4a-h** (1 eq.) and 2,4-dinitrophenyltosylate (**13a**) or triflate (**13b**) (1.1 eq.) were suspended in acetone at 40 °C or in MeCN (1.4 mL/mmol) at r.t. for 18 h. After cooling to room temperature Et_2O (4 mL/mmol) was added, and the product was left to precipitate while stirred for 2 h. The solid was filtered, washed with Et_2O (8 mL/mmol) and dried on air.

1-(2,4-Dinitrophenyl)-4-(1-(((4-fluorophenoxy)carbonyl)oxy)ethyl)pyridin-1-ium 4-methylbenzenesulfonate (5a)

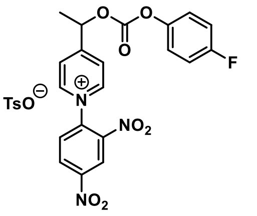

Prepared according to the general procedure from **4a** (269 mg, 1.03 mmol) and 2,4-dinitrophenyltosylate (**13a**) (418 mg, 1.24 mmol) in acetone. Yield 534 mg (87%). White solid. M.p. 199.3 °C (decomp.). ^{1}H NMR (500 MHz, d_6-DMSO) δ (ppm) 9.40 (d, J = 6.3 Hz, 2H), 9.12 (d, J = 2.5 Hz, 1H), 8.97 (dd, J_1 = 8.6 Hz, J_2 = 2.4 Hz, 1H), 8.51 (d, J = 6.4 Hz, 2H), 8.42 (d, J = 8.7 Hz, 1H), 7.49–7.43 (m, 2H), 7.41–7.34 (m, 2H), 7.34–7.27 (m, 2H), 7.10 (d, J = 7.7 Hz, 2H), 6.21 (q, J = 6.7 Hz, 1H), 2.28 (s, 3H), 1.75 (d, J = 6.7 Hz, 3H). ^{13}C NMR (126 MHz, d_6-DMSO) δ (ppm) 162.4, 159.9 (d, J = 242.4 Hz), 152.1, 149.2, 146.7 (d, J = 2.6 Hz), 146.4, 145.8, 143.1, 138.5, 137.5, 131.9, 130.2, 128.0, 125.5, 124.4, 123.2 (d, J = 8.7 Hz), 121.4, 116.3 (d, J = 23.6 Hz), 74.7, 21.2, 20.8. ^{19}F NMR (471 MHz, d-$CDCl_3$) δ (ppm) 116.23. HRMS (ESI+) calcd. for [$C_{20}H_{15}FN_3O_7^+$] 428.0894, found 428.0893.

1-(2,4-Dinitrophenyl)-4-(1-((((4-methoxybenzyl)oxy)carbonyl)oxy)ethyl)pyridin-1-ium 4-methylbenzenesulfonate (5b)

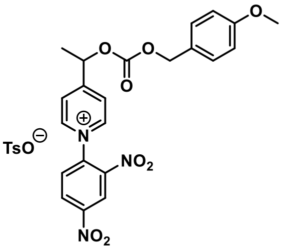

Prepared according to the general procedure from **4b** (240 mg, 0.84 mmol) and 2,4-dinitrophenyltosylate (**13a**) (339 mg, 1 mmol) in acetone. Yield 405 mg (78%). White solid. M.p. 176.1–176.3 °C. ^{1}H NMR (500 MHz, d_6-DMSO) δ (ppm) 9.36 (d, J = 6.3 Hz, 2H), 9.12 (d, J = 2.5 Hz, 1H), 8.96 (dd, J_1 = 8.7 Hz, J_2 = 2.5 Hz, 1H), 8.41 (dd, J_1 = 13.4 Hz, J_2 = 7.5 Hz, 3H), 7.46 (d, J = 7.7 Hz, 2H), 7.36 (d, J = 8.2 Hz, 2H), 7.10 (d, J = 7.7 Hz, 2H), 6.96 (d, J = 8.3 Hz, 2H), 6.10 (q, J = 6.7 Hz, 1H), 5.18–5.11 (m, 2H), 3.76 (s, 3H), 2.28 (s, 3H), 1.65 (d, J = 6.7 Hz, 3H). ^{13}C NMR (126 MHz, d_6-DMSO) δ (ppm) 162.8, 159.4, 153.3, 149.0, 146.2, 142.9, 138.4, 137.3, 131.73, 130.2, 130.0, 127.9, 126.8, 125.3, 124.24, 124.16, 121.2, 113.8, 73.4, 69.5, 55.0, 21.1, 20.6. HRMS (ESI+) calcd. for [$C_{22}H_{20}N_3O_8^+$] 454.1250, found 454.1239.

1-(2,4-Dinitrophenyl)-4-(1-((((4-methoxybenzyl)thio)carbonyl)oxy)ethyl)pyridin-1-ium 4-methylbenzenesulfonate (5c)

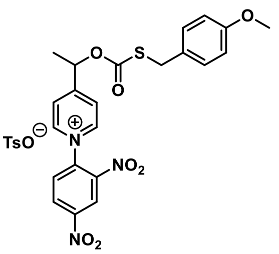

Prepared according to the general procedure from **4c** (197 mg, 0.65 mmol) and 2,4-dinitrophenyltosylate (**13a**) (242 mg, 0.71 mmol) in acetone. Yield 364 mg (87%). White solid. M.p. 181.6–181.9 °C. ^{1}H NMR (500 MHz, d_6-DMSO) δ (ppm) 9.37 (d, J = 6.3 Hz, 2H), 9.11 (d, J = 2.5 Hz, 1H), 8.96 (dd, J_1 = 8.7 Hz, J_2 = 2.5 Hz, 1H), 8.44–8.38 (m, 3H), 7.45 (d, J = 7.7 Hz, 2H), 7.29 (d, J = 8.2 Hz, 2H), 7.10 (d, J = 7.7 Hz, 2H), 6.89 (d, J = 8.6 Hz, 2H), 6.32 (q, J = 6.7 Hz, 1H), 4.16 (s, 2H), 3.73 (s, 3H), 2.28 (s, 3H), 1.66 (d, J = 6.6 Hz, 3H).^{13}C NMR (126 MHz, d_6-DMSO) δ (ppm) 169.7, 162.6, 158.6, 149.2, 146.5, 145.8, 143.1, 138.5, 137.5, 131.9, 130.2, 130.1, 128.7, 128.0, 125.5, 121.4, 114.0, 73.3, 55.1, 34.2, 21.2, 20.8. HRMS (ESI+) calcd. for [$C_{22}H_{20}N_3O_7S^+$] 470.1022, found 470.1022.

1-(2,4-Dinitrophenyl)-4-(1-((morpholine-4-carbonyl)oxy)ethyl)pyridin-1-ium trifluoromethanesulfonate (5d)

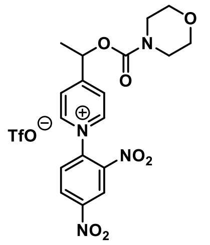

Prepared according to the general procedure from **4d** (100 mg, 0.423 mmol) and 2,4-dinitrophenyltriflate (**13b**) (176 mg, 0.51 mmol) in MeCN. Yield 195 mg (83%). Orange solid. M.p. 170.3–170.9°C. ^{1}H NMR (500 MHz, d_6-DMSO) δ (ppm) 9.33 (d, J = 6.2 Hz, 2H), 9.15–9.11 (m, 2H), 8.97 (dd, J = 8.8, 2.4 Hz, 1H), 8.45–8.39 (m, 3H), 6.08 (q, J = 6.8 Hz, 1H), 3.62–3.37 (m, 6H), 1.62 (d, J = 6.7 Hz, 3H). ^{13}C NMR (126 MHz, d_6-DMSO) δ (ppm) 164.7,

153.4, 149.2, 146.2, 143.1, 138.6, 132.0, 130.2, 124.2, 121.4, 71.0, 65.8, 44.1, 43.7, 40.1, 21.7. HRMS (ESI+) calcd. for [$C_{18}H_{19}N_4O_7^+$] 403.1254, found 403.1250.

1-(2,4-Dinitrophenyl)-4-(1-(((4-methoxybenzyl)(methyl)carbamoyl)oxy)ethyl)pyridin-1-ium 4-methylbenzenesulfonate (5f)

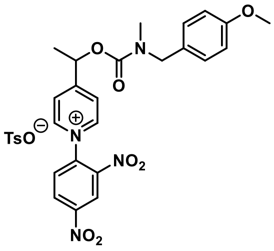

Prepared according to the general procedure from **4f** (340 mg, 1.13 mmol) and 2,4-dinitrophenyltosylate (**13a**) (459 mg, 1.36 mmol) in MeCN. Yield 512 mg (71%). White solid. Mixture of two rotamers. M.p. 152.2 °C (decomp.). ^{1}H NMR (500 MHz, d_6-DMSO) δ (ppm) ^{1}H NMR (500 MHz, DMSO-d_6) δ 9.38–9.29 (m, 2H), 9.12 (s, 1H), 8.96 (dd, J_1 = 8.6 Hz, J_2 = 2.4 Hz, 1H), 8.45–8.40 (m, 2H), 8.34 (d, J = 6.2 Hz, 1H), 7.46 (d, J = 7.9 Hz, 2H), 7.27–7.18 (m, 2H), 7.10 (d, J = 7.7 Hz, 2H), 6.95–6.90 (m, 2H), 6.09 (q, J = 6.8 Hz, 1H), 4.68–4.40 (m, 1H), 4.37 (s, 1H), 3.74 (s, 4H), 2.96–2.83 (m, 3H), 2.28 (s, 3H), 1.64–1.61 (m, 3H). ^{13}C NMR (126 MHz, d_6-DMSO) δ (ppm) 164.9, 164.7, 158.6, 154.7, 154.2, 149.1, 146.2, 145.8, 143.1, 143.1, 138.6, 137.5, 131.9, 130.2, 129.4, 129.2, 129.0, 128.9, 128.0, 125.5, 124.1, 121.4, 114.02, 113.98, 71.0, 55.1, 51.2, 51.1, 34.3, 33.5, 21.7, 21.6, 20.8. HRMS (ESI+) calcd. for [$C_{23}H_{23}N_4O_7^+$] 467.1567, found 467.1561.

1-(2,4-dinitrophenyl)-4-(1-((ethyl(2-hydroxy-2-(3-hydroxyphenyl)ethyl)carbamoyl)oxy) ethyl)pyridin-1-ium trifluoromethanesulfonate (5g)

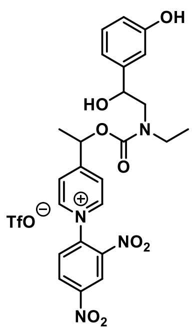

Prepared according to the general procedure from **4g** (485 mg, 1.5 mmol) and 2, 4-dinitrophenyltriflate (**13b**) (511 mg, 1.6 mmol) in MeCN. Yield 900 mg (95%). Pale orange solid. Mixture of two rotamers and two diastereomers. M.p. 86.0–86.2 °C. ^{1}H NMR (500 MHz, d_4-CD_3OD) δ (ppm) 9.33–9.05 (m, 3H), 8.95–8.86 (m, 1H), 8.42–7.90 (m, 3H), 7.23–7.11 (m, 1H), 6.95–6.65 (m, 3H), 6.09–5.80 (m, 1H), 4.79 (d, J = 9.7 Hz, 1H), 3.78–3.33 (m, 4H), 1.77–1.59 (m, 3H), 1.26–1.08 (m, 3H). ^{13}C NMR (126 MHz, d_4-CD_3OD) δ (ppm) 168.2, 167.9, 158.7, 158.5, 156.7, 156.3, 151.2, 147.3, 147.2, 147.1, 145.84, 145.80, 144.7, 140.00, 139.98, 132.6, 131.10, 131.07, 130.5, 130.4, 123.2, 119.0, 118.5, 118.4, 118.3, 115.8, 115.6, 115.5, 114.5, 114.1, 73.3, 73.1, 73.0, 73.0, 72.9, 56.0, 55.8, 54.9, 54.7, 44.7, 44.6, 44.3, 44.1, 22.01, 21.98, 21.95, 21.9, 13.9, 13.0, 12.9. HRMS (ESI+) calcd. for [$C_{24}H_{25}N_4O_8^+$] 497.1672, found 497.1658.

1-(2,4-Dinitrophenyl)-4-(2-(((4-methoxybenzyl)(methyl)carbamoyl)oxy)propan-2-yl)pyridin-1-ium 4-methylbenzenesulfonate (5h)

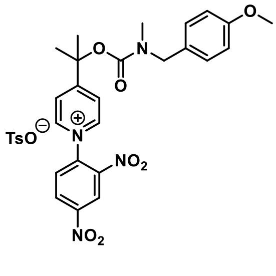

Prepared according to the general procedure from **4h** (200 mg, 0.64 mmol) and 2,4-dinitrophenyltosylate (**13a**) (237 mg, 0.7 mmol) in acetone. Yield 382 mg (92%). White solid. Mixture of two rotamers. M.p. 177.4–177.6°C. ^{1}H NMR (500 MHz, d_6-DMSO) δ (ppm) 9.34–9.29 (m, 2H), 9.12–9.11 (m, 1H), 8.96–8.93 (m, 1H), 8.51–8.40 (m, 1H), 8.38–8.24 (m, 2H), 7.45 (d, J = 7.4 Hz, 3H), 7.30–7.14 (m, 2H), 7.11 (d, J = 7.7 Hz, 2H), 6.98–6.89 (m, 2H), 4.51–4.28 (m, 2H), 3.76–3.73 (m, 3H), 2.92–2.77 (m, 3H), 2.28 (s, 3H), 1.85 (s, 3H), 1.81 (s, 3H). ^{13}C NMR (126 MHz, d_6-DMSO) δ (ppm) 169.3, 169.3, 159.1, 159.1, 154.7, 154.1, 149.6, 146.3, 146.22, 146.18, 143.7, 143.6, 139.0, 138.0, 132.5, 130.6, 130.0, 129.8, 129.4, 129.4, 128.5, 126.0, 124.0, 123.9, 121.9, 114.53, 114.45, 79.54, 79.51, 55.6, 55.5, 51.9, 51.3, 34.6, 34.2, 28.0, 27.9, 21.3. HRMS (ESI+) calcd. for [$C_{24}H_{25}N_4O_7^+$] 481.1723, found 481.1724.

1-(2,4-Dinitrophenyl)-4-(1-((((4-methyl-2-oxo-2H-chromen-7-yl)oxy)carbonyl)oxy)ethyl)pyridin-1-ium 4-methylbenzenesulfonate (5i)

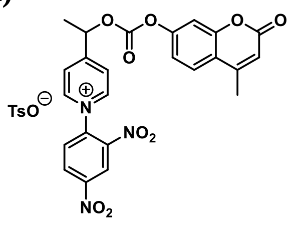

Prepared according to the general procedure from **4i** (236 mg, 0.73 mmol) and 2,4-dinitrophenyltosylate (**13a**) (270 mg, 0.8 mmol) in acetonitrile. Yield 330 mg (69%). White solid. ^{1}H NMR (500 MHz, d_6-DMSO) δ (ppm) 9.42 (d, J = 6.6 Hz, 2H), 9.11 (d, J = 2.4 Hz, 1H), 8.96 (dd, J_1 = 8.7 Hz, J_2 = 2.5 Hz, 1H), 8.53 (d, J = 6.6 Hz, 2H), 8.42 (d, J = 8.7 Hz, 1H), 7.88 (d, J = 8.7 Hz, 1H), 7.48 (d, J = 2.3 Hz, 1H), 7.45 (d, J = 7.8 Hz, 2H), 7.38 (dd, J = 8.7 Hz, J_2 = 2.3 Hz, 1H), 7.10 (d, J = 7.8 Hz, 2H), 6.43 (s, 1H), 6.25 (q, J = 6.6 Hz, 1H), 2.45 (s, 3H), 2.28 (s, 3H), 1.77 (d, J = 6.7 Hz, 3H). ^{13}C NMR (126 MHz, d_6-DMSO) δ (ppm) 162.3, 159.5, 153.5, 152.9, 151.5, 149.2, 146.4, 145.8, 143.1, 138.5, 137.6, 131.9, 130.2, 128.0, 126.8, 125.5, 121.4, 118.1, 117.7, 114.1, 109.6, 75.0, 21.2, 20.8, 18.2. HRMS (ESI+) calcd. for [$C_{24}H_{18}N_3O_9^+$] 492.1043, found 492.1032.

1-(2,4-Dinitrophenyl)-4-(1-methoxyethyl)pyridin-1-ium 4-methylbenzenesulfonate (15)

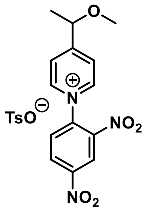

Prepared according to the general procedure from **14** (277 mg, 2.02 mmol) and 2,4-dinitrophenyltosylate (**13a**) (750 mg, 2.22 mmol) in acetone. Yield 951 mg (99%). Off-white solid. M.p. 174.0 °C (decomp.). ^{1}H NMR (500 MHz, d_6-DMSO) δ (ppm) 9.33 (d, J = 6.5 Hz, 2H), 9.11 (d, J = 2.5 Hz, 1H), 8.96 (dd, J_1 = 8.7 Hz, J_2 = 2.5 Hz, 1H), 8.40 (d, J = 8.7 Hz, 1H), 8.36 (d, J = 6.5 Hz, 2H), 7.45 (d, J = 8.0 Hz, 2H), 7.10 (d, J = 7.8 Hz, 2H), 4.85 (q, J = 6.6 Hz, 1H), 3.36 (s, 3H), 2.28 (s, 3H), 1.49 (d, J = 6.6 Hz, 3H). ^{13}C NMR (126 MHz, d_6-DMSO) δ (ppm) 166.5, 149.1, 146.0, 145.8, 143.1, 138.6, 137.5, 131.9, 130.2, 128.0, 125.5, 124.7, 121.4, 76.8, 56.9, 21.9, 20.8. HRMS (ESI+) calcd. for [$C_{14}H_{14}N_3O_5^+$] 304.0933, found 304.0925.

General Procedure for Synthesis of Cyanines 1a–h.

The corresponding Zincke salt **5a-h** (1 eq.) and heterocycle **6a-c** (3 eq) and AcOK (6 eq.) were mixed in MeCN (15 mL/mmol for **5a-c, 5h** and 3 mL/mmol for **5d-g**). The reaction mixture was stirred at room temperature for 5 – 18 h in a flask wrapped in aluminum foil. The volatiles were evaporated under reduced pressure, Et_2O (3 mL/mmol) added, and the precipitate was filtered, dried, washed with Et_2O (6 mL/mmol), H_2O (4 mL/mmol) and Et_2O (2.5 mL/mmol) (if not stated otherwise). The crude product was purified by column chromatography (SiO_2, gradient CH_2Cl_2/MeOH - 50:1 to 30:1, unless stated otherwise). In the case of carbamates, products were isolated as mixture of rotamers which merged upon heating.

2-((1*E*,3*Z*,5*E*)-4-(1-(((4-Fluorophenoxy)carbonyl)oxy)ethyl)-7-((E)-5-methoxy-1,3,3-trimethylindolin-2-ylidene)hepta-1,3,5-trien-1-yl)-5-methoxy-1,3,3-trimethyl-3*H*-indol-1-ium iodide (1a)

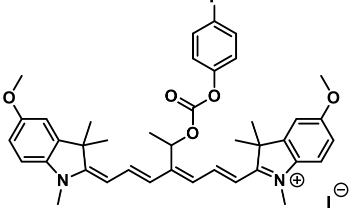

Prepared according to the general procedure from **5a** (300 mg, 0.5 mmol) and heterocycle **6a** (497 mg, 1.5 mmol), reaction time 18 h. Yield 254 mg (65%). Dark green solid. M.p. 141.4–141.5 °C. ^{1}H NMR (500 MHz, d_4-CD_3OD) δ (ppm) 8.20 (dd, J_1 = 13.3 Hz, J_2 = 13.4 Hz, 2H), 7.21 (d, J = 8.7 Hz, 2H), 7.20–7.13 (m, 2H), 7.14–7.09 (m, 4H), 6.96 (dd, J = 8.8, 2.5 Hz, 2H), 6.53 (d, J = 13.4 Hz, 2H), 6.30 (d, J = 13.3 Hz, 2H), 6.08 (q, J = 6.8 Hz, 1H), 3.84 (s, 6H), 3.61 (s, 6H), 1.77 (d, J = 6.8 Hz, 3H), 1.72 (s, 6H), 1.70 (s, 6H). ^{13}C NMR (126 MHz, d_4-CD_3OD) δ (ppm) 172.9, 161.8 (d, J = 243.4 Hz), 159.9, 159.0, 148.5 (d, J = 2.9 Hz), 144.4, 144.2, 138.0, 123.8 (d, J = 8.7 Hz), 122.6, 117.1 (d, J = 24.1 Hz), 114.8, 112.5, 110.0, 105.9, 75.7, 56.5, 50.5, 31.8, 30.7, 28.3, 28.1, 21.8. ^{19}F NMR (471 MHz, d_4-CD_3OD) δ (ppm) 118.87. HRMS (ESI+) calcd. for [$C_{40}H_{44}FN_2O_5^+$] 651.3234, found 651.3225.

5-Methoxy-2-((1*E*,3*Z*,5*E*)-7-((*E*)-5-methoxy-1,3,3-trimethylindolin-2-ylidene)-4-(1-((((4-methoxybenzyl)oxy)carbonyl)oxy)ethyl)hepta-1,3,5-trien-1-yl)-1,3,3-trimethyl-3*H*-indol-1-ium iodide (1b)

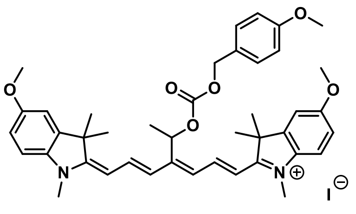

Prepared according to the general procedure from **5b** (300 mg, 0.48 mmol) and heterocycle **6a** (477 mg, 1.44 mmol), reaction time 18 h. Yield 232 mg (60%). Dark green solid. M.p. 131.2–131.4 °C. ^{1}H NMR (500 MHz, d_4-CD_3OD) δ (ppm) 8.14 (dd, J_1 = 13.2 Hz, J_2 = 13.4 Hz, 2H), 7.29–7.25 (m, 2H), 7.19 (d, *J* = 8.6 Hz, 2H), 7.09 (d, *J* = 2.5 Hz, 2H), 6.96 (dd, J_1 = 8.6 Hz, J_2 = 2.4 Hz, 2H), 6.88–6.84 (m, 2H), 6.43 (d, *J* = 13.4 Hz, 2H), 6.22 (d, *J* = 13.2 Hz, 2H), 5.96 (q, *J* = 6.8 Hz, 1H), 5.13–5.04 (m, 2H), 3.85 (s, 6H), 3.73 (s, 3H), 3.58 (s, 6H), 1.69–1.61 (m, 15H). ^{13}C NMR (126 MHz, d_4-CD_3OD) δ (ppm) ^{13}C NMR (126 MHz, d_4-CD_3OD) δ 172.8, 161.5, 160.0, 159.8, 155.7, 144.5, 144.2, 138.0, 131.4, 128.8, 122.9, 114.9, 114.7, 112.4, 110.0, 105.7, 74.7, 70.8, 56.5, 55.7, 50.5, 31.7, 28.3, 28.2, 21.9. HRMS (ESI+) calcd. for [$C_{42}H_{49}N_2O_6^+$] 677.3591, found 677.3582.

5-Methoxy-2-((1*E*,3*Z*,5*E*)-7-((*E*)-5-methoxy-1,3,3-trimethylindolin-2-ylidene)-4-(1-((((4-methoxybenzyl)thio)carbonyl)oxy)ethyl)hepta-1,3,5-trien-1-yl)-1,3,3-trimethyl-3*H*-indol-1-ium iodide (1c)

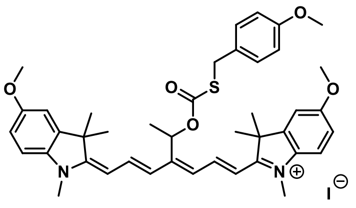

Prepared according to the general procedure from **5c** (145 mg, 0.23 mmol) and heterocycle **6a** (225 mg, 0.68 mmol), reaction time 18 h. Yield 88 mg (47%). Dark green solid. M.p. 138.8–139.2 °C. ^{1}H NMR (500 MHz, d_4-CD_3OD) δ (ppm) 8.12 (dd, J_1 = 13.4 Hz, J_2 = 13.4 Hz, 2H), 7.26–7.17 (m, 4H), 7.11 (s, 2H), 6.96 (d, *J* = 8.5 Hz, 2H), 6.82 (d, *J* = 8.1 Hz, 2H), 6.46 (d, *J* = 13.4 Hz, 2H), 6.33–6.13 (m, 3H), 4.07 (s, 2H), 3.85 (s, 6H), 3.73 (s, 3H), 3.60 (s, 6H), 1.74–1.61 (m, 15H). ^{13}C NMR (126 MHz, d_4-CD_3OD) δ (ppm) 172.8, 171.5, 160.6, 159.9, 159.4, 144.3, 144.2, 138.0, 131.1, 130.6, 122.6, 115.0, 114.8, 112.5, 110.0, 105.8, 74.0, 56.5, 55.7, 50.5, 35.7, 31.8, 28.3, 28.3, 21.7. HRMS (ESI+) calcd. for [$C_{42}H_{49}N_2O_5S^+$] 693.3362, found 693.3365.

5-Methoxy-2-((1*E*,3*Z*,5*E*)-7-((*E*)-5-methoxy-1,3,3-trimethylindolin-2-ylidene)-4-(1-((morpholine-4-carbonyl)oxy)ethyl)hepta-1,3,5-trien-1-yl)-1,3,3-trimethyl-3*H*-indol-1-ium iodide (1d)

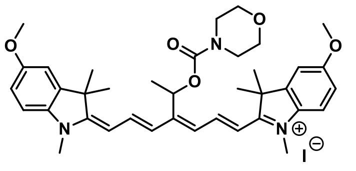

Prepared according to the general procedure from **5d** (100 mg, 0.25 mmol) and heterocycle **6a** (152 mg, 0.74 mmol), reaction time 5 h. Yield 57 mg (40%). Dark green solid. M.p. 143.1–143.2 °C. ^{1}H NMR (500 MHz, d_4-CD_3OD) δ (ppm) 8.11 (dd, J_1 = 13.6 Hz, J_2 = 13.3 Hz, 2H), 7.20 (d, *J* = 8.7 Hz, 2H), 7.14–7.09 (m, 2H), 6.97 (dd, J_1 = 8.7 Hz, J_2 = 2.2 Hz, 2H), 6.57 (d, *J* = 13.6 Hz, 2H), 6.31 (d, *J* = 13.3 Hz, 2H), 6.03 (q, *J* = 6.7 Hz, 1H), 3.85 (s, 8H), 3.66–3.61 (m, 4H), 3.61 (s, 8H), 3.60–3.35 (m, 4H), 1.70 (s, 12H), 1.62 (d, *J* = 6.7 Hz, 3H). ^{13}C NMR (126 MHz, d_4-CD_3OD) δ (ppm) 171.3, 159.3, 158.4, 154.9, 142.7, 142.6, 136.6, 119.9, 113.3, 111.0, 108.6, 104.3, 70.2, 66.2, 55.0, 49.0, 30.3, 26.9, 26.8, 20.3. HRMS (ESI+) calcd. for [$C_{38}H_{48}N_3O_5^+$] 626.3594, found 626.3597.

5-Methoxy-2-((1*E*,3*Z*,5*E*)-7-((*E*)-5-methoxy-1,3,3-trimethylindolin-2-ylidene)-4-(1-(((4-methoxybenzyl)(methyl)carbamoyl)oxy)ethyl)hepta-1,3,5-trien-1-yl)-1,3,3-trimethyl-3*H*-indol-1-ium iodide (1e)

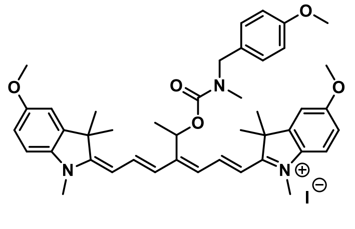

Prepared according to the general procedure from **5e** (180 mg, 0.36 mmol) and heterocycle **6a** (222 mg, 1.1 mmol), reaction time 16 h. Yield 108 mg (37%). Dark green solid. Mixture of rotamers. M.p. 143.4–143.5 °C. ^{1}H NMR (400 MHz, DMSO-d_6, 80 °C) δ 8.00 (dd, J_1 = 13.3 Hz, J_2 = 13.4 Hz, 2H), 7.27 (d, J = 8.7 Hz, 2H), 7.22 – 7.14 (m, 4H), 6.97 (dd, J_1 = 8.7 Hz, J_2 = 2.6 Hz, 2H), 6.89 (d, J = 8.7 Hz, 2H), 6.57 (d, J = 13.2 Hz, 2H), 6.30 (d, J = 13.4 Hz, 2H), 6.02 (q, J = 6.6 Hz, 1H), 4.41 (s, 2H), 3.83 (s, 6H), 3.72 (s, 3H), 3.60 (s, 6H), 2.84 (s, 3H), 1.65 (d, J = 4.8 Hz, 12H), 1.5 (d, J = 6.7 Hz, 3H). ^{13}C NMR (126 MHz, d_4-CD_3OD, 47 °C) δ (ppm) 172.7, 159.8, 144.1, 144.1, 138.0, 130.7, 130.2, 129.8, 121.1, 115.1, 115.0, 114.7, 112.4, 110.0, 105.7, 71.8, 71.2, 56.5, 55.7, 52.8, 50.5, 34.9, 34.1, 31.8, 28.4, 28.2, 21.6. HRMS (ESI+) calcd. for [$C_{43}H_{52}N_3O_5^+$] 690.3907, found 690.3895.

Potassium 3-(5-methoxy-2-((1*E*,3*Z*,5*E*)-7-((*E*)-5-methoxy-3,3-dimethyl-1-(3-sulfonatopropyl)indolin-2-ylidene)-4-(1-(((4-methoxybenzyl)(methyl)carbamoyl)oxy) ethyl)hepta-1,3,5-trien-1-yl)-3,3-dimethyl-3*H*-indol-1-ium-1-yl)propane-1-sulfonate (1f)

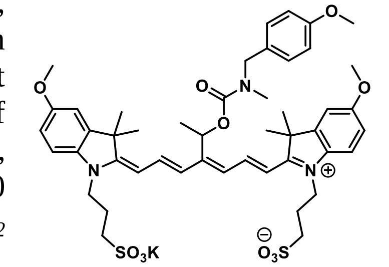

Prepared according to the general procedure from **5e** (169 mg, 0.36 mmol) and heterocycle **6b** (321 mg, 1.1 mmol), reaction time 18 h. Column chromatography CH_2Cl_2/MeOH – gradient 50:1 to 10:1. Yield 53 mg (14%). Dark green solid. Mixture of rotamers. M.p. 145.4–145.6 °C. ^{1}H NMR (500 MHz, d_6-DMSO, 80 °C) δ (ppm) 8.06 (dd, J_1 = 13.3 Hz, J_2 = 13.4 Hz, 2H), 7.40 (d, J = 8.7 Hz, 2H), 7.19–7.17 (m, 4H), 6.95 (dd, J_1 = 8.7 Hz, J_2 = 2.5 Hz, 2H), 6.88 (d, J = 8.4 Hz, 2H) 6.60 (d, J = 13.2 Hz, 2H), 6.46 (d, J = 13.3 Hz, 2H), 6.02 (q, J = 6.8 Hz, 1H), 4.41 (s, 2H), 4.27 (t, J = 7.6 Hz, 4H), 3.82 (s, 6H), 3.72 (s, 3H), 2.84 (s, 3H), 2.60 (t, J = 6.8 Hz, 4H), 2.06 (m, 4H), 1.65 (s, 6H), 1.64 (s, 6H), 1.58 (d, J = 6.6 Hz, 3H). ^{13}C NMR (126 MHz, d_4-CD_3OD, 47 °C) δ (ppm) 172.1, 160.6, 159.8, 144.4, 137.2, 130.6, 130.2, 129.9, 115.1, 114.9, 112.7, 110.0, 105.9, 56.4, 55.7, 52.8, 50.6, 44.0, 28.5, 28.4, 24.3. HRMS (ESI+) calcd. for [$C_{47}H_{58}N_3O_{11}S_2^-$] 904.3513, found 904.3522.

Potassium 3-(2-((1*E*,3*Z*,5*E*)-4-(1-((ethyl(2-hydroxy-2-(3-hydroxyphenyl)ethyl)carbamoyl) oxy)ethyl)-7-((*E*)-5-methoxy-3,3-dimethyl-1-(3-sulfonatopropyl)indolin-2-ylidene)hepta-1,3,5-trien-1-yl)-5-methoxy-3,3-dimethyl-3*H*-indol-1-ium-1-yl)propane-1-sulfonate (1g)

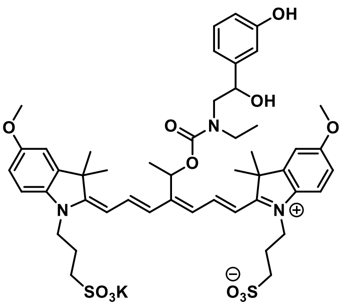

Prepared according to the general procedure from **5g** (250 mg, 0.43 mmol) and heterocycle **6b** (380 mg, 1.3 mmol), reaction time 18 h. Crude product was extracted using CH_2Cl_2/brine and purified by column chromatography CH_2Cl_2/MeOH – gradient 50:1 to 10:1. Yield 130 mg (33%). Dark green solid. Mixture of diastereomers and rotamers. M.p. 118.8–119.0 °C. ^{1}H NMR (500 MHz, d_6-DMSO, 80 °C) δ (ppm) 9.08 (brs, 1H),), 8.02 (2×dd, J_1 = 13.1 Hz, J_1 = 12.9 Hz, 2H), 7.39 (dd, J = 8.8, 1.3 Hz, 1H), 7.18 (d, J = 2.5 Hz, 2H), 7.08 (m, 1H), 6.95 (dd, J_1 = 8.7 Hz, J_2 = 2.5 Hz, 1H), 6.77 (m, 2H), 6.62 (2×d, J = 13.0 Hz, 2H), 6.49 (2×d, J = 13.5 Hz, 2H), 5.96 (m, 1H), 5.20 (brs, 1H), 4.70 (m, 1H), 4.26 (t, J = 7.6 Hz, 4H), 3.82 (s, 6H), 3.33 (m, 4H), 2.60 (t, J = 6.8 Hz, 4H), 2.06 (m, 4H), 1.65 (m, 12H), 1.54 (m, 3H), 1.05 (t, J = 7.0 Hz, 1H). ^{13}C

NMR (126 MHz, d_6-DMSO, 47 °C) δ (ppm) 170.5, 158.1, 157.7, 143.3, 136.4, 129.4, 114.5, 114.1, 112.4, 109.6, 105.4, 70.3, 56.4, 49.4, 49.4, 48.5, 43.5, 28.1, 28.0, 24.1, 21.6. HRMS (ESI+) calcd. for [$C_{48}H_{60}N_3O_{12}S_2^-$] 934.3618, found 934.3638.

2-((1*E*,3*Z*,5*E*)-4-(2-(((4-Methoxybenzyl)(methyl)carbamoyl)oxy)propan-2-yl)-7-((*E*)-1,3,3-trimethylindolin-2-ylidene)hepta-1,3,5-trien-1-yl)-1,3,3-trimethyl-3*H*-indol-1-ium iodide (1h)

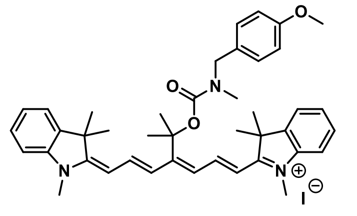

Prepared according to the general procedure from **5h** (200 mg, 0.31 mmol) and heterocycle **6c** (276 mg, 0.92 mmol), reaction time 18 h. Yield 39 mg (15%). Dark green solid. Mixture of rotamers. M.p. 135.4–135.6 °C. ^{1}H NMR (500 MHz, d_4-CD_3OD) δ (ppm) 8.03–7.78 (m, 2H), 7.52–6.83 (m, 11H), 6.81–6.30 (m, 5H), 4.57–4.28 (m, 2H), 3.92–3.37 (m, 9H), 3.03–2.68 (m, 3H), 1.92–1.47 (m, 18H). ^{13}C NMR (126 MHz, d_4-CD_3OD) δ (ppm) 173.4, 169.7, 160.5, 156.7, 149.0, 148.3, 144.5, 142.1, 130.6, 130.11, 130.05, 129.8, 126.0, 123.3, 122.2, 121.8, 115.1, 114.9, 111.6, 106.0, 105.8, 83.3, 55.7, 55.6, 53.2, 52.3, 50.3, 34.4, 31.9, 28.6, 28.4, 28.3, 28.2. HRMS (ESI+) calcd. for [$C_{42}H_{50}N_3O_3^+$] 644.3852, found 644.3849.

2-((1*E*,3*Z*,5*E*)-4-(1-(((4-Fluorophenoxy)carbonyl)oxy)ethyl)-7-((*E*)-1,3,3-trimethylindolin-2-ylidene)hepta-1,3,5-trien-1-yl)-1,3,3-trimethyl-3*H*-indol-1-ium iodide (1j)

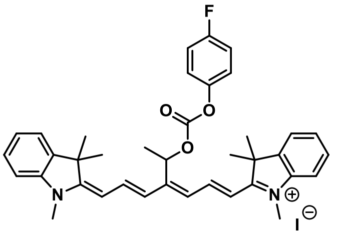

Prepared according to the general procedure from **5a** (100 mg, 0.17 mmol) and heterocycle **6c** (151 mg, 0.5 mmol), reaction time 18 h. Yield 94 mg (78%). Dark green solid. M.p. 140.0–140.5 °C. ^{1}H NMR (500 MHz, d_4-CD_3OD) δ (ppm) 8.28 (dd, J_1 = 13.4 Hz, J_2 = 13.4 Hz, 2H), 7.49 (dd, J_1 = 7.5 Hz, J_2 = 1.1 Hz, 2H), 7.44 – 7.38 (m, 2H), 7.31 – 7.24 (m, 4H), 7.18 – 7.09 (m, 4H), 6.60 (d, J = 13.5 Hz, 2H), 6.39 (d, J = 13.3 Hz, 2H), 6.10 (q, J = 6.8 Hz, 1H), 3.65 (s, 6H), 1.78 (d, J = 6.8 Hz, 3H), 1.73 (s, 6H), 1.71 (s, 6H). ^{13}C NMR (126 MHz, d_4-CD_3OD) δ (ppm) 173.9, 161.8 (d, J = 243.4 Hz), 160.4, 154.3, 148.5 (d, J = 2.7 Hz), 145.7, 143.5 (d, J = 238.9 Hz), 129.8, 126.3, 123.83, 123.77, 123.3, 117.1 (d, J = 24.0 Hz), 111.9, 106.3, 75.6, 50.4, 31.7, 30.8, 28.3, 28.1, 21.9. HRMS (ESI+) calcd. for [$C_{38}H_{40}FN_2O_3^+$] 591.3023, found 591.3023.

5-Methoxy-2-((1*E*,3*Z*,5*E*)-7-((*E*)-5-methoxy-1,3,3-trimethylindolin-2-ylidene)-4-(1-((((4-methyl-2-oxo-2*H*-chromen-7-yl)oxy)carbonyl)oxy)ethyl)hepta-1,3,5-trien-1-yl)-1,3,3-trimethyl-3*H*-indol-1-ium iodide (1k)

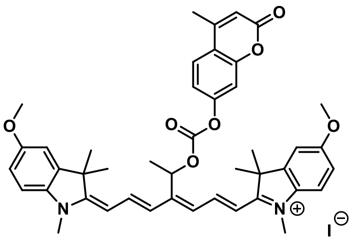

Prepared according to the general procedure from **5i** (130 mg, 0.2 mmol) and heterocycle **6a** (195 mg, 0.59 mmol), reaction time 18 h. Yield 92 mg (56%). Dark green solid. ^{1}H NMR (500 MHz, d_6-DMSO) δ (ppm) 8.00 (dd, J_1 = 13.4 Hz, J_2 = 13.3 Hz, 2H), 7.82 (d, J = 8.8 Hz, 1H), 7.33 (d, J = 2.4 Hz, 1H), 7.30 (d, J = 8.7 Hz, 2H), 7.25 (d, J = 2.5 Hz, 2H), 7.23 (dd, J = 8.7, 2.4 Hz, 1H), 6.96 (dd, J = 8.7, 2.5 Hz, 2H), 6.55 (d, J = 13.3 Hz, 2H), 6.40 (d, J = 1.3 Hz, 1H), 6.37 (d, J = 13.4 Hz, 2H), 6.10 (q, J = 6.9 Hz, 1H), 3.79 (s, 6H), 3.59 (s, 6H), 2.42 (d, J = 1.3 Hz, 3H), 1.69 (d, J = 6.7 Hz, 3H), 1.65 (s, 6H), 1.63 (s, 6H). ^{13}C NMR (126 MHz, d_6-DMSO) δ (ppm) ^{13}C NMR (126 MHz, DMSO-d_6) δ 170.7, 159.6, 157.7, 155.6, 153.6, 152.9, 152.7, 151.5, 142.8, 141.6, 136.6, 126.8, 118.0, 117.5, 114.1, 113.5, 111.8, 109.4, 109.1, 105.4, 74.6, 55.9, 48.9, 40.1, 31.5, 27.4, 27.2, 21.3, 18.2. HRMS (ESI+) calcd. for [$C_{44}H_{47}N_2O_7^+$] 715.3383, found 715.3382.

5-Methoxy-2-((1*E*,3*Z*,5*E*)-7-((*E*)-5-methoxy-1,3,3-trimethylindolin-2-ylidene)-4-(1-methoxyethyl)hepta-1,3,5-trien-1-yl)-1,3,3-trimethyl-3*H*-indol-1-ium iodide (8)

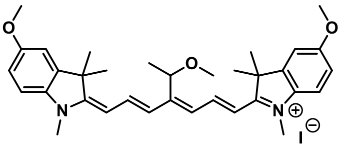

Prepared according to the general procedure from **14** (150 mg, 0.32 mmol) and heterocycle **6a** (313 mg, 0.95 mmol), reaction time 18 h at 40°C under dry and oxygen–free conditions. Yield 31 mg (15%). Dark green solid. M.p. 144.0 °C (decomp.). ^{1}H NMR (400 MHz, d_4-CD_3OD) δ (ppm) 8.32 (dd, J_1 = 13.5 Hz, J_1 = 13.5 Hz, 2H), 7.21 (d, J = 8.7 Hz, 2H), 7.12 (d, J = 2.5 Hz, 2H), 6.98 (dd, J = 8.7, 2.5 Hz, 2H), 6.52 (d, J = 13.4 Hz, 2H), 6.24 (d, J = 13.5 Hz, 2H), 4.71 (q, J = 6.6 Hz, 1H), 3.87 (s, 6H), 3.61 (s, 6H), 3.36 (s, 3H), 1.71 (s, 6H), 1.70 (s, 6H), 1.58 (d, J = 6.7 Hz, 3H). ^{13}C NMR (126 MHz, d_4-CD_3OD) δ (ppm) 171.3, 161.7, 158.3, 143.3, 142.7, 136.6, 113.3, 110.9, 108.6, 104.0, 55.5, 55.0, 49.0, 48.2, 30.2, 26.9, 26.8, 22.0. HRMS (ESI+) calcd. for [$C_{34}H_{43}N_2O_3^+$] 527.3274, found 527.3276.

Synthesis of 1,2-bis(4-methoxybenzyl)disulfane (16)

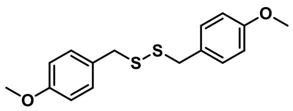

Thiol **10c** (170 mg, 1.1 mmol) was dissolved in MeOH (2 mL). A solution of NaOH (1.4 mL, 2.5 M aq. solution, 2.75 mmol) was added and the mixture was left stirring for 5 minutes. Subsequently, saturated aqueous solution of CuSO4 (0.2 mL, 1.1 mmol) was added and the reaction mixture was stirred under compressed air overpressure at room temperature for 2 h. Suspension was filtered, washed with water and the solid was suspended in AcOEt. The mixture was filtered again, liquid phase was evaporated under reduced pressure to afford product. Yield 151 mg (90%). White solid. Characterization was in accordance with literature.[5]

Photophysical and Photochemical Measurements Methodology

UV-Vis Absorption and Emission Measurements.

Fluorescence Measurements.

Emission spectra were measured in methanol using a fluorescence spectrometer in a 1.0 cm quartz fluorescence cuvette at 20 °C. The sample concentrations were adjusted to keep the absorbance <0.15 at the corresponding excitation wavelength. Each sample was measured five times, and the spectra were averaged. Emission spectra were normalized and corrected by the photomultiplier sensitivity function using correction files supplied by the manufacturer. The fluorescence quantum yields (Φ_F) were determined using integration sphere, each sample was measured five times using independent solutions keeping A<0.15, and the spectra were averaged. In all cases, the Φ_F were below 2% which is the detection limit of the machine in this spectral range.

Irradiation Experiments and Dark Stability.

A solution of photocage **1a–h** (c ~1–1.5 × 10^{-5} M, 3100 μL, A < 1.5) in aerated methanol or PBS (pH 7.4, 10 mM, I = 100 mM, with 20% DMSO) was stirred and left to equilibrate for 2–3 min at 20 °C. Afterward, the sample was irradiated with LEDs at 820 nm (~25 mW/cm^2) and the progress of the irradiation was monitored at the given time intervals by UV-vis spectrometry. The total irradiation time was selected to reach >95% conversion and to obtain minimum of 30 experimental points. The procedure was repeated three times. The dark stability of **1a–h** was recorded using the same procedure with exclusion of the irradiation source.

Decomposition Quantum Yield Determination.

A solution of photocage **1a–h** (c < 1 × 10^{-5} M, 3100 μL, A < 1.5) in aerated PBS (pH 7.4, 10 mM, I = 100 mM with 20% DMSO) was stirred and left to equilibrate for 2–3 min at 20 °C.

Afterward, the sample was irradiated with a beam of collimated light at 780 nm (Ø=7 mm) and UV-vis spectra were recorded periodically using diode-array spectrophotometer. The radiant power (flux; Φ_e) of the light source was determined using calibrated Si-photodiode and optical power meter (~40 mW). The total irradiation time was selected to reach <10% conversion of the photocages and to obtain ten experimental points. The procedure was repeated three times. The quantum yield of decomposition $\boldsymbol{\Phi}_{\mathbf{dec}}$ was calculated according to the equation:

$$\phi_{\text{dec}} = \frac{\Delta n_{dec}}{\Delta n_{abs}^{p}} \qquad \text{(Eq. 1)}$$

where Δ*n* is the number of moles of the photodecomposed photocage **1a–h** calculated from the absorbance change at $\lambda_{\max}$, and Δn^{p}_{abs} is the number of moles of photons absorbed by the sample in the give time period, calculated according to the equation:

$$\Delta n_{abs}^{p} = \frac{\int_0^t \int_0^\infty (1 - 10^{-A(\lambda,t)})\ I_\lambda^{em}\ d\lambda\ dt}{6.022\ \times\ 10^{23}} \qquad \text{(Eq. 2)}$$

where $A(\lambda,$t) is the absorbance of the sample at the wavelength λ in time *t,* and I_λ^{em} is the photon flux of the LED source at the wavelength λ determined according to the equation:

$$I_\lambda^{em} = \ q_n(\lambda)\ \frac{\Phi_e}{\int_0^\infty \frac{hc}{\lambda}\ q_n(\lambda)\ d\lambda} \qquad \text{(Eq. 3)}$$

where $q_n(\lambda)$ is the emission spectrum of the LED source provided by manufacturer (counts vs. wavelength) and Φ_e is the radiant power (flux) measured by the optical power meter.

For experiments under exclusion of oxygen, the sample prepared as described above was treated with Ar using continuous flux of Ar (2 bubbles per second) from a needle attached to a balloon filled with Ar, for 15 minutes.

NMR Irradiation and Chemical Yield Quantification Experiments.
Photocage **1a–h** (~0.6–0.8 mg) was dissolved in aerated or degassed *d*$_4$-CD_3OD (0.5 mL). Methanol for experiments under oxygen-free conditions was extensively degassed by 5 freeze-pump-thaw cycles using liquid N_2 and the samples were prepared in a glovebox. For the purpose of the chemical yield quantification, C_6F_6 and TMSB (1,4-bis(trimethylsilyl)benzene) were used as internal standards for ^{19}F NMR and ^{1}H NMR spectroscopy respectively. The NMR tube was then irradiated with a set of LEDs at 820 nm (~25 mW/cm^2; at from the NMR tube ~5 cm) and ^{1}H and ^{19}F NMR spectra with prolonged acquisition times were recorded after indicated time intervals. In case of **1a,** the NMR sample after the irradiation under oxygen-free conditions was fully characterized using NMR spectroscopy and subsequently analyzed by HRMS upon dilution. In the control dark experiments, samples were prepared as described above and kept in dark throughout the duration of the experiment.

Irradiation Experiments in the Presence of LiCl.
Photocage **1i** (~0.6 mg)[6] was dissolved in degassed *d*$_4$-CD_3OD (0.6 mL) containing LiCl (100 mM). *d*$_4$-CD_3OD for this experiment was prepared as described above and contained same internal standards. The NMR tube was then irradiated with LED light source at 810 nm (~300 mW cm^{-2}),^{1}H NMR and ^{19}F NMR spectra were recorded at indicated time intervals. Measurement was repeated three times. Control samples were prepared as described above and

kept in dark. Experiment was repeated as described above in CH_3OH to further prove nature of species formed in the reaction.

Quantum Yield of Uncaging via Photoheterolytic Pathway.
Photocage **1a** (1.83–1.85 mg) was dissolved in degassed d_4-CD_3OD (0.6 mL) and quantitatively transferred into NMR tube in glovebox. d_4-CD_3OD for this experiment was prepared as described above and contained same internal standards. The NMR tube was then irradiated with an LED light source at 810 nm (~300 mW cm^{-2}, at a fixed distance of ~3 cm, cooled by a fan at 1200 rpm) and ^{1}H NMR spectra were recorded using acquisition times at indicated time intervals. As a reference, ICG (indocyanine green) (1.79–1.81 mg) was dissolved in aerated d_4-CD_3OD (0.55 mL) and irradiated using the same LED light source at 810 nm. The quantum yield of decomposition of ICG Φ_{ICG} was estimated using equation:

$$\Phi_{ICG} = \Phi_{\Delta} \frac{k_r[ICG]}{k_d + k_r[ICG]}$$

where the $\Phi_{\Delta} = 0.008$, $k_r = (8.7 \pm 0.5) \times 10^6$ M^{-1} s^{-1} and $k_d = 4.41 \times 10^3$ s^{-1} in d_4-CD_3OD. [ICG] corresponds to initial concentration of ICG (~4 mM). [7,8]

Graph of Δn_{dec} or $\Delta n_{release}$ vs. time (t (s)) was then constructed and the $\Phi_{release}$ was calculated using equation:

$$\Phi_{release} = \Phi_{ICG} \frac{slope(1a)}{slope(\mathrm{ICG})}$$

where Φ_{ICG} is quantum yield of decomposition of ICG calculated above and slope(X) and slope(ICG) are slopes of the linear regression of dependence of Δn_{dec} or $\Delta n_{release}$ vs. time (t (s)).

Both measurements (sample and the reference) were repeated three times and the value $\Phi_{release}$ is given as an average with a standard deviation of the mean.

Irradiation in MeOH/H_2O at Low Concentration Analyzed by HRMS.

Photocage **1a** (~0.6 mg) was dissolved MeOH/H_2O (3mL, 1:1) in UV-vis cuvette under ambient conditions. The cuvette was then irradiated with a set of LEDs at 820 nm (~25 mW/cm^2) and HRMS spectrum was recorded after 40 minutes.

Calculating the Limiting Concentration for Photooxidation vs. Direct Uncaging

Method A

The concentration of **1a** at which Φ_{het} and Φ_{ox} are equal can be crudely estimated from the comparison of slopes in Figures S108. Dividing the slopes for the two processes tells us that the rate of ICG photooxidation is ~17 times faster than uncaging from **1a**. Since photooxidation is concentration dependent, decreasing concentration (c ~ 3.8 mM) of ICG 17-fold will result in equal rates of these two processes, i.e. at c ~2×10^{-4} M. However, this assumes that **1a** has the same affinity towards $^1O_2$ and its quantum yield of $^1O_2$ production (Φ_{Δ}) is also the same as ICG.

Method B

At the limiting concentration, the quantum yields Φ_{het} and Φ_{ox} must be equal:

$$\Phi_{het} = \Phi_{ox}$$

and Φ_{ox}, which depends on concentration of **1a**, can be derived from the rate law for **1a** disappearance and steady-state approximation[11] for $^1O_2$:

$$\Phi_{ox} = \Phi_{\Delta} \frac{k_r[\mathbf{1a}]}{k_d + k_r[\mathbf{1a}]}$$

where Φ_{Δ} is the quantum yield of $^1O_2$ production, k_d is the rate constant of $^1O_2$ quenching by the solvent and k_r is the rate constant of the bimolecular reaction between **1a** and $^1O_2$.

Combining these two equations we get:

$$\Phi_{het} = \Phi_{\Delta} \frac{k_r[\mathbf{1a}]}{k_d + k_r[\mathbf{1a}]}$$

The substituent in C4′ in **1a** is electronically disconnected from the cyanine core, and we can thus assume that its k_d and k_r are comparable to those of analogous unsubstituted Cy7 dyes. This is a reasonable assumption given that these properties vary minimally between unsubstituted Cy7, and Cy7 substituted with C3′-Me and C3′-Ph.[7]

Solving this equation for values $\Phi_{het} = 6.8 \times 10^{-4}$, $\Phi_{\Delta} = 0.0089$, $k_r = 7.1 \times 10^6$ M^{-1} s^{-1} and $k_d = 9\times10^4$ s^{-1} in methanol, gives the limiting concentration of [**1a**] at which these processes in methanol proceed with the same efficiency equal to c~ 1×10^{-4} M.[7,8,9]

Methodology of Biological Experiments

Cell Viability Assays.
HeLa cells were seeded in 96 well plates at a density of 4×10^3 (1.5×10^3 for 72 h viability assay) cells per well and grown to 30% confluency for 24 hours 37°C at 5% CO_2 atmosphere. The half of the media (50 µL) was removed and substituted with media containing different concentrations of **1f** or **1g** or the photoproducts of **1f** or **1g** (DMSO stock solution with c ~ 1×10^{-2} M of parent compound was diluted with DMEM (4.5 g/L glucose, L-glutamine, 1% pyruvate, 10% FBS, 1% P/S) to obtain stock solution with 1% DMSO, which was further diluted with DMEM (4.5 g glucose, L-glutamine, 1% pyruvate, 10% FBS, 1% P/S) to reach the final concentration of compound). The amount of DMSO in well was kept stable at 0.1%. The photoproducts were prepared by irradiation of solution of **1f** or **1g** (c ~ 1×10^{-2} M) in DMSO at 780 nm light (25 mW/cm^2) for 16 hours. The cells were incubated for 24 h or 72 h at 37 °C at 5% CO_2 atmosphere after the addition of compound. The cells were then left to equilibrate to room temperature for 30 minutes and a half of the media (50 µL) media was removed. Then 50 µL of CellTiter-Glo (prepared according to Promega protocol) was added and the cells were mixed with the reagent using rocking shaker at maximum speed for 15 minutes. Then the cells were left at room temperature in dark for 30 minutes. The luminescence was detected using plate reader in luminescence mode as an integral over all wavelengths (1000 ms integration time), and a plot of the cell viability was obtained from three replicates under each condition.

All experiments were repeated three times using cells from different passages. The cell viability was calculated using cells with 0 µM concentration of compound as a refence.

Culturing and Differentiation of iPSC Cardiomyocytes

Human iPSC were cultured on iMatrix-511 (Takara) coated plates in StemBrew medium (Miltenyi Biotec). The culture medium was changed every day. At 80% confluence iPSC were seeded for cardiomyocytes differentiation following the instructions based on StemMACS CardioDiff Kit XF (Miltenyi Biotec). First, iPSC were cultured for 24h in mesoderm induction media to initiate the differentiation. Then cells were cultured for additional 24 h in cardiac maintenance and afterwards cardiac induction media. Cardiomyocytes started to beat around day 7. At day 10 cells were detached using a 1:1 trypsin and Stempro accutase mixture, followed by collagenase diluted in RPME 1640 (Gibco) for cell dissociation. Then, cells were seeded for further processing. All cells were incubated at 37°C in an atmosphere with 95% humidity and 5% CO_2.

Quantification of the Beating Rate in iPSC-Derived Cardiomyocytes.

The growth medium was aspirated from the 48-well plate containing confluent iPSC cardiomyocyte cells. Fluo 8 AM (5 µM,) (AAT Bioquest Cat. No. 21081) in a mixture 1:1 (0.4 mL) of growth medium and Hepes Buffered Tyrode's solution (ThermoFisher Cat. No. 50151910) was used to stain the cells. Afterwards the cells were incubated at 37°C at 5% CO_2 atmosphere for 1 hour and imaged by a widefield microscope.

The cells were subsequently incubated with **1g** (800 nM, 0.01 % DMSO) and kept in the dark or irradiated with LEDs (λ_{max}=780 nm, power ~ 40 mW) for 5 minutes, or incubated with free etilefrine (400 nM) as a control experiment. After 10 minutes of incubation, the cells were imaged by fluorescence microscopy. The cells were placed in light microscope and kept at 37°C at 5% CO_2 atmosphere throughout the duration of experiments. The cells were visualized using fully motorized, inverted microscope (Zeiss Axio Observer) with a fluorescence light source Lumencore SpectraX at 20× magnification: Fluo 8 AM fluorescence channel (exc.484 nm, 3% intensity, exposure 200 ms, 525 nm detection), in 150 cycles. The images were processed using ImageJ. The trace of the total cell fluorescence in a period of ~30 seconds was plotted from 30–50 living cells. Photobleaching correction was applied using bleach correction plugin for Fiji with exponential fitting.[10] The experiments were performed using 4–6 independent wells per experiment, and the mean of the determined beating rates and the IQR are given. The beating frequency was calculated by counting the number of local maxima (beats) of the total cell fluorescence and dividing the total duration between the first and the last beat by this number.

NMR Spectroscopy

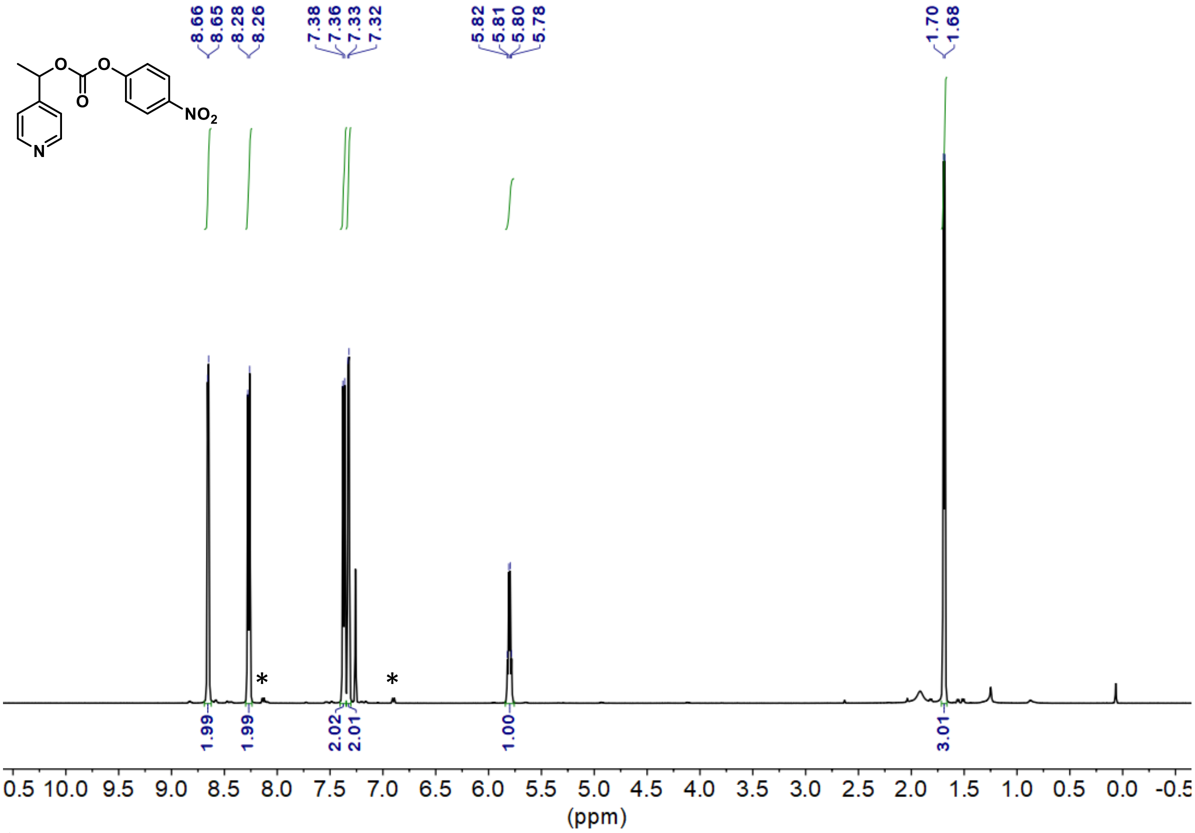

^{1}H NMR (500 MHz, *d*-$CDCl_3$): **3,** *4-nitrophenol

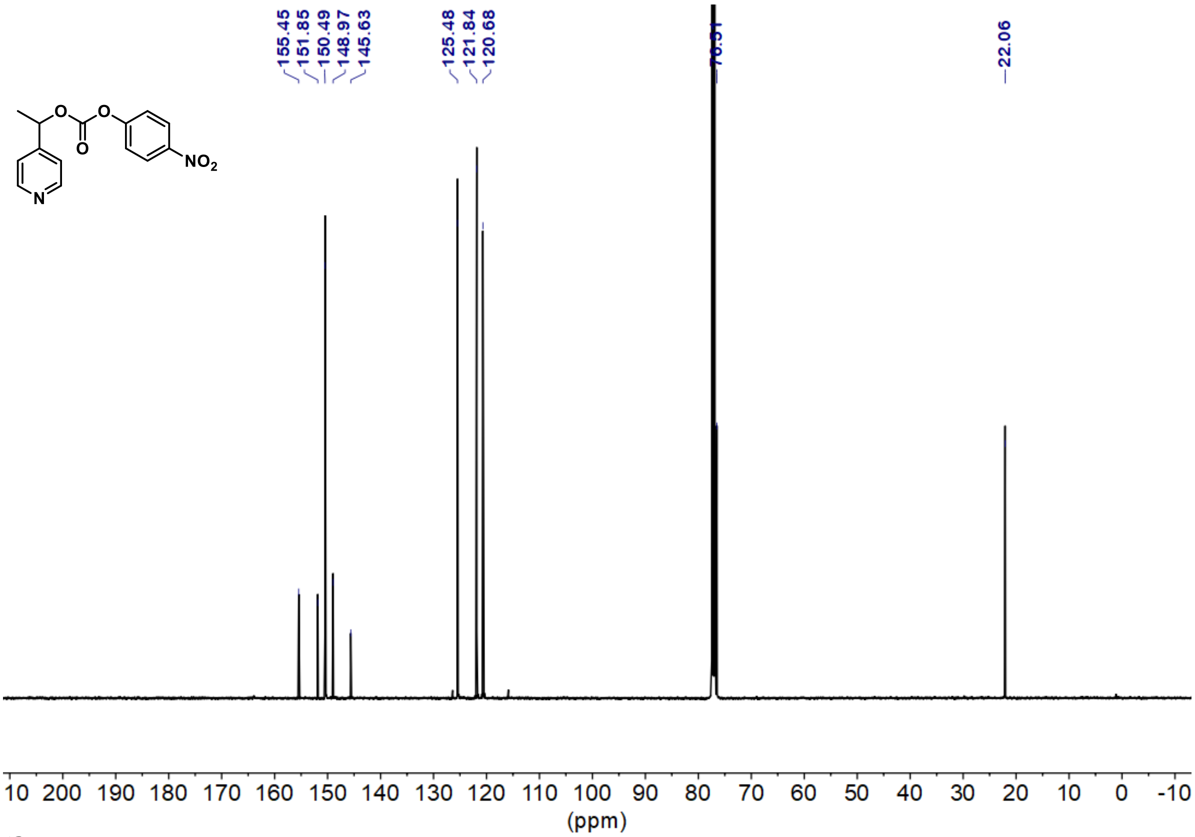

^{13}C NMR (126 MHz, *d*-$CDCl_3$): **3**

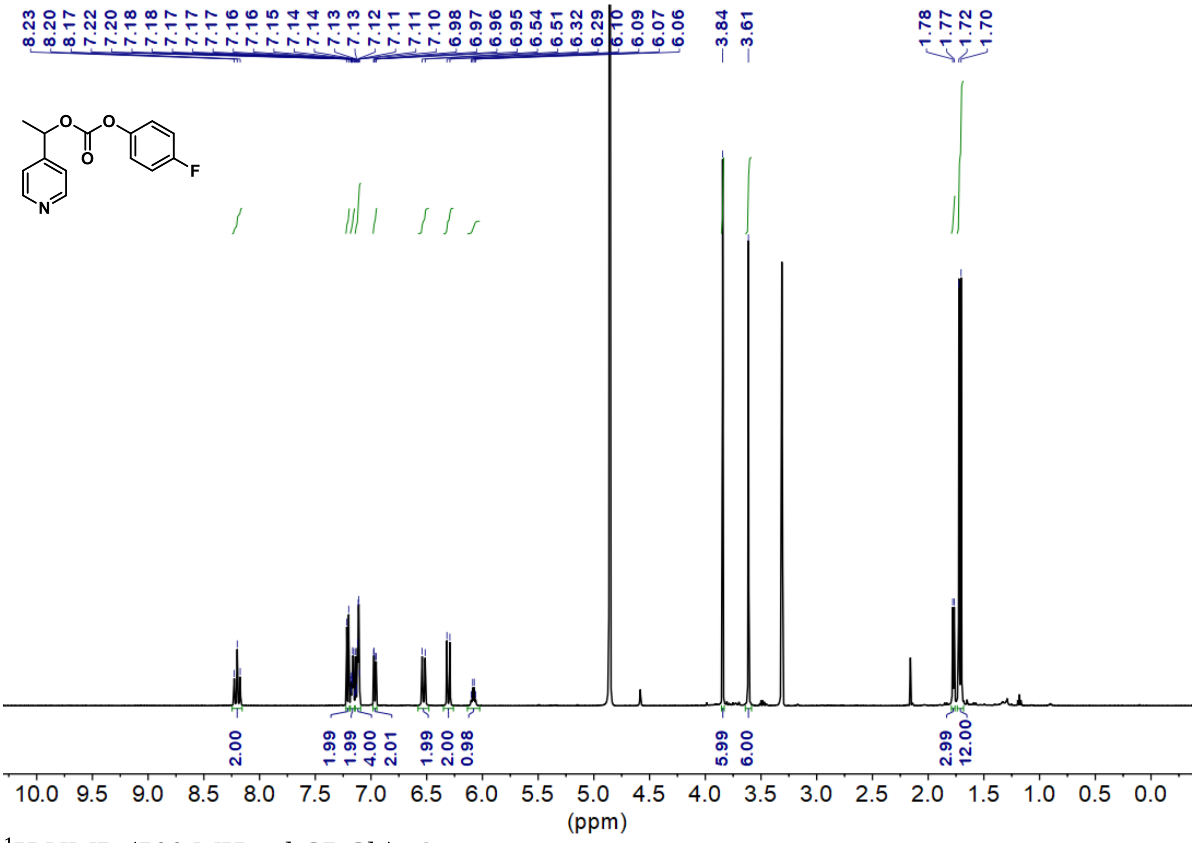

^{1}H NMR (500 MHz, *d*-$CDCl_3$): **4a**

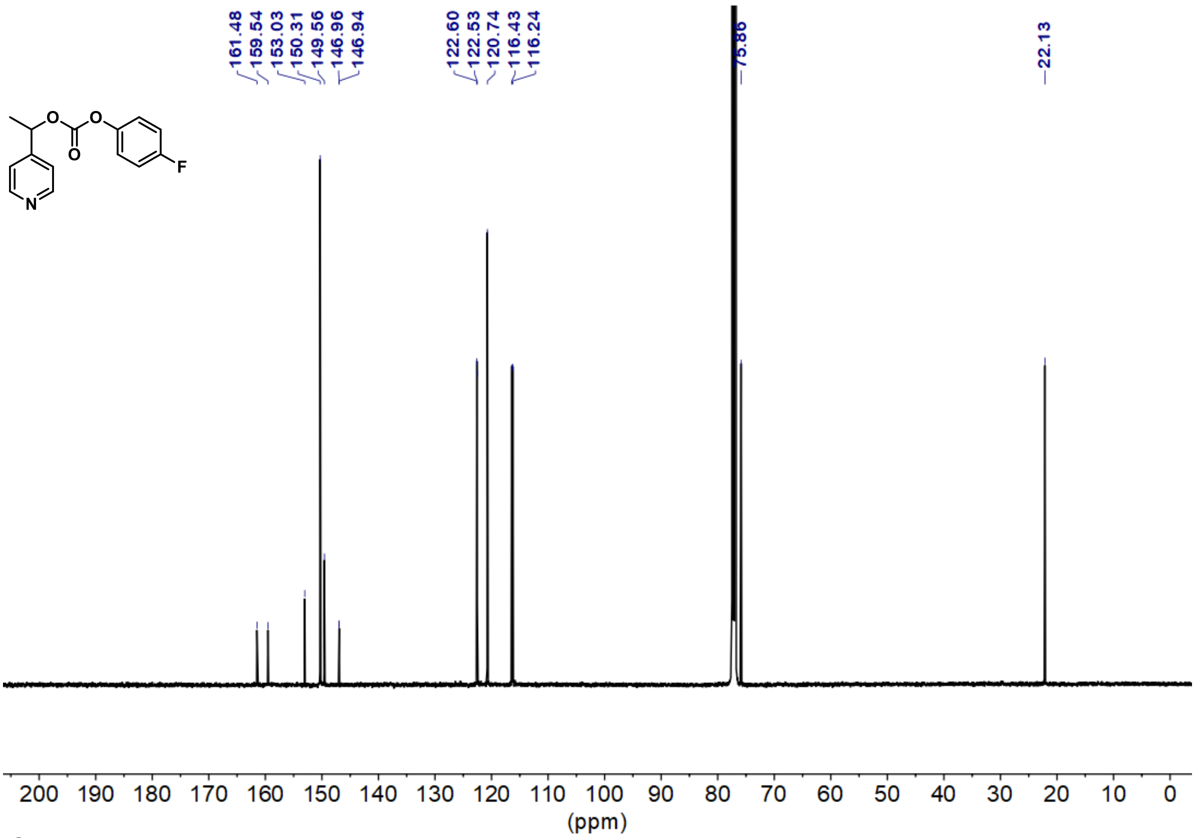

^{13}C NMR (126 MHz, *d*-$CDCl_3$): **4a**

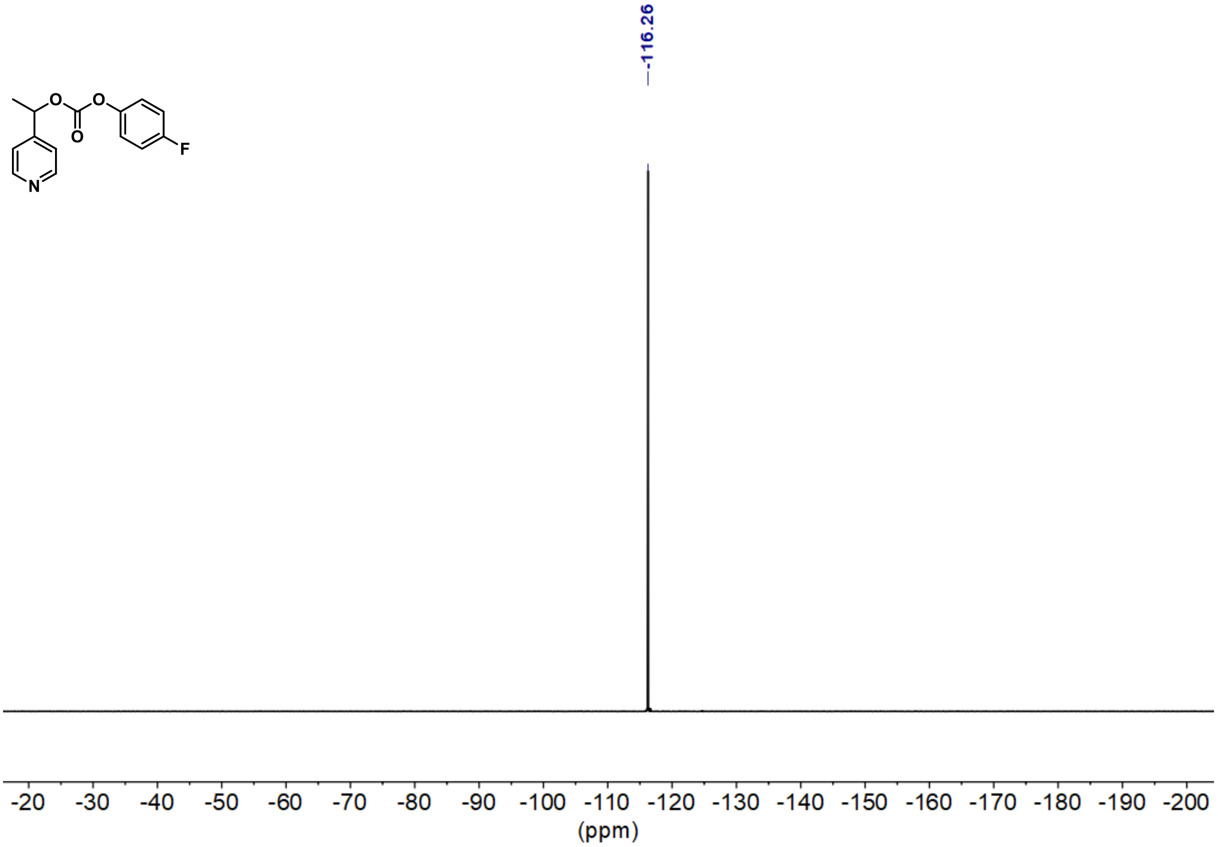

^{19}F NMR (470 MHz, *d*-$CDCl_3$): **4a**

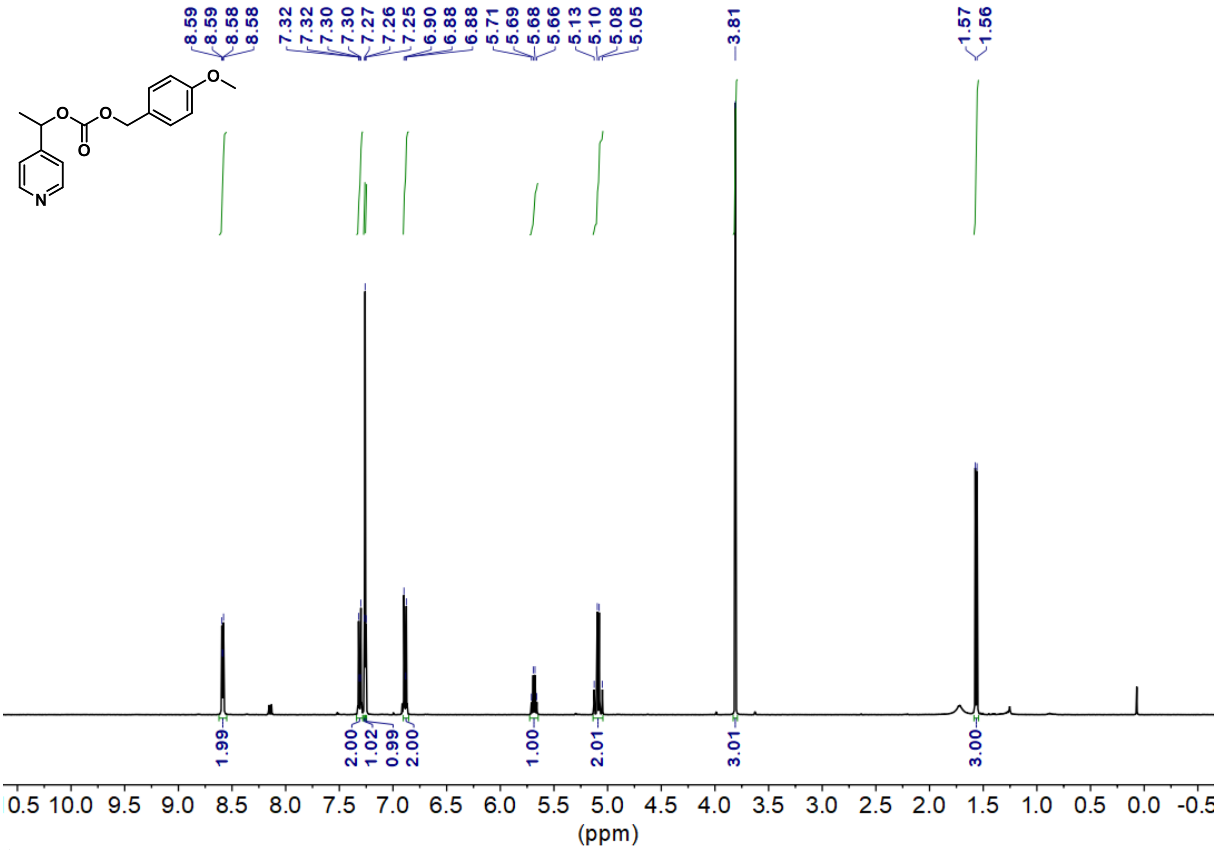

^{1}H NMR (400 MHz, *d*-$CDCl_3$): **4b**

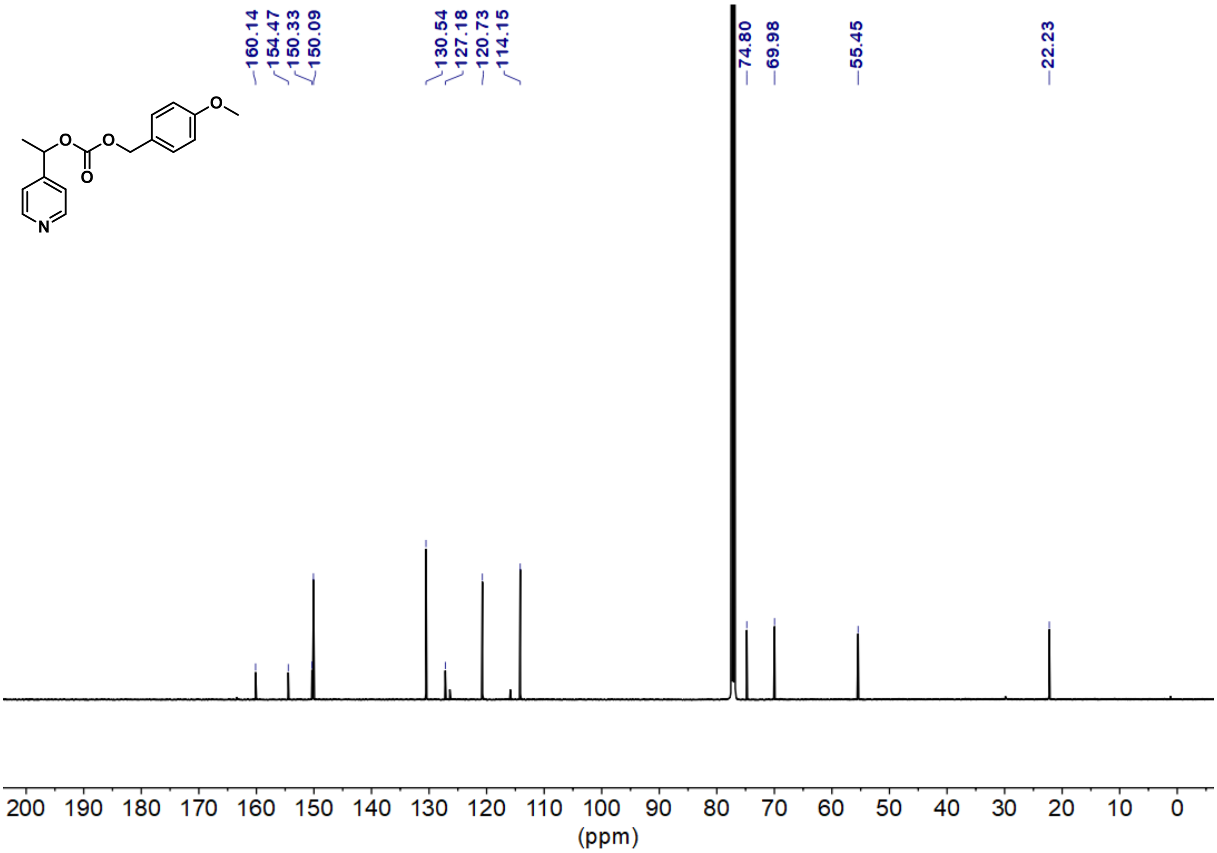

^{13}C NMR (126 MHz, *d*-$CDCl_3$): **4b**

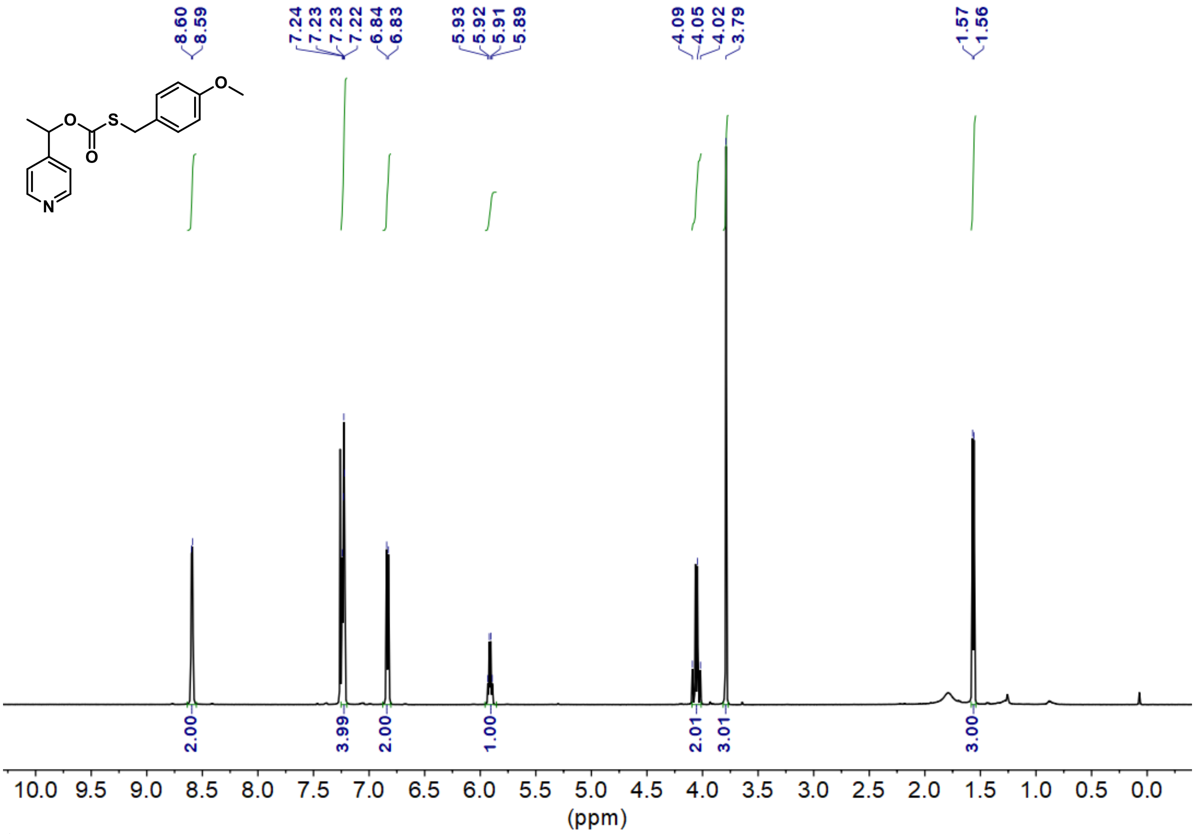

^{1}H NMR (500 MHz, *d*-$CDCl_3$): **4c**

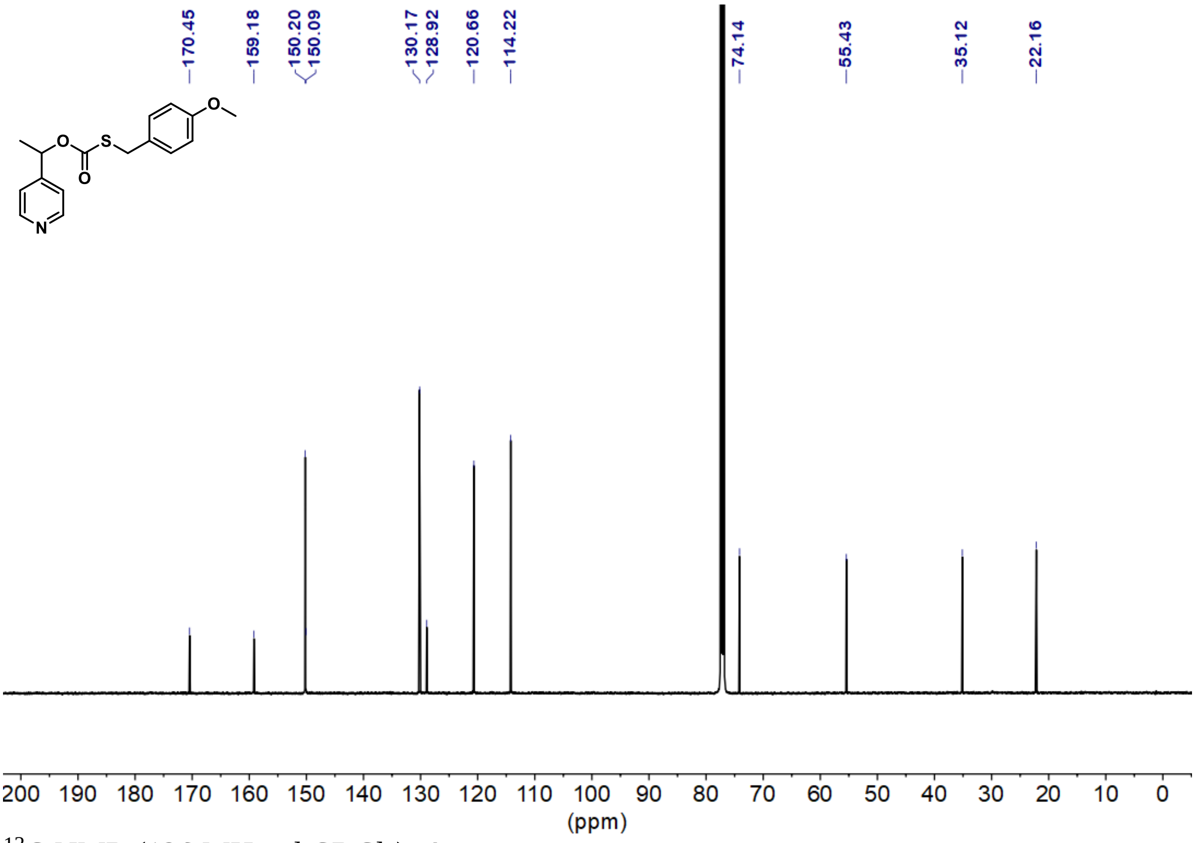

^{13}C NMR (126 MHz, *d*-$CDCl_3$): **4c**

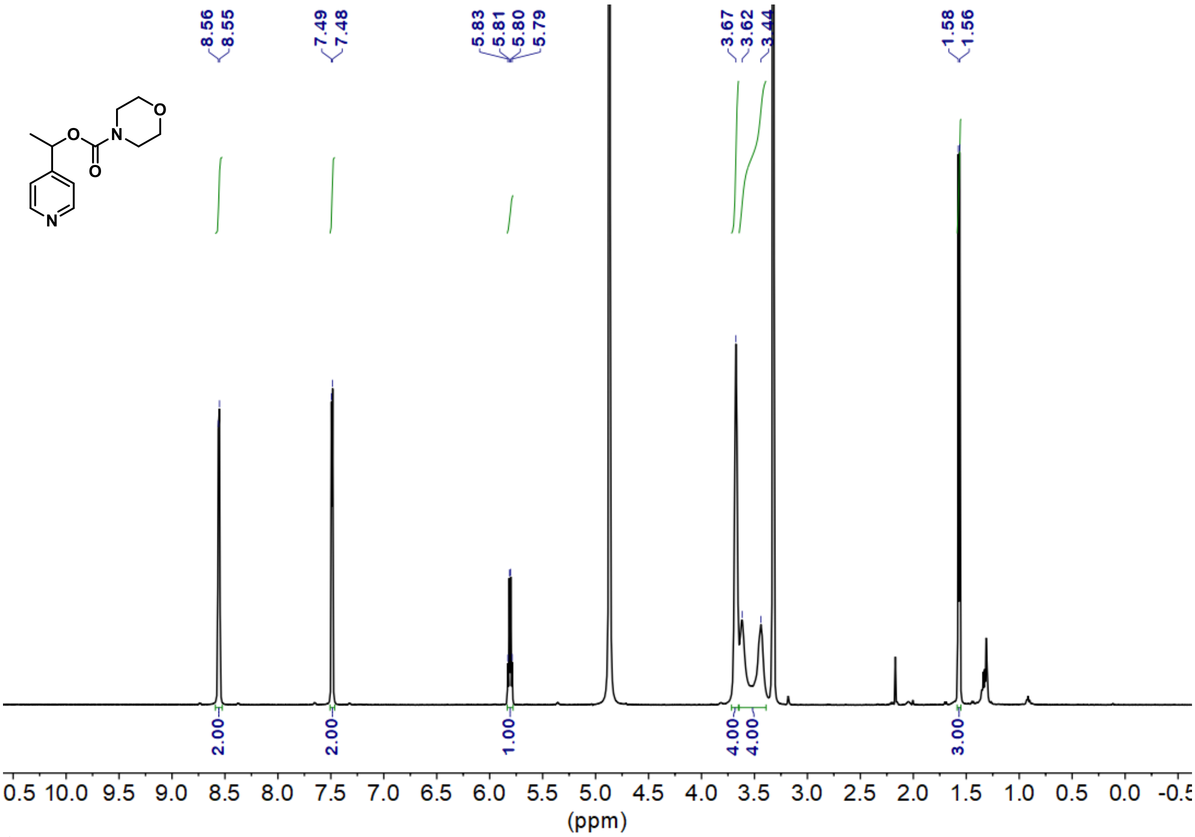

^{1}H NMR (500 MHz, *d*$_4$-CD_3OD): **4d**

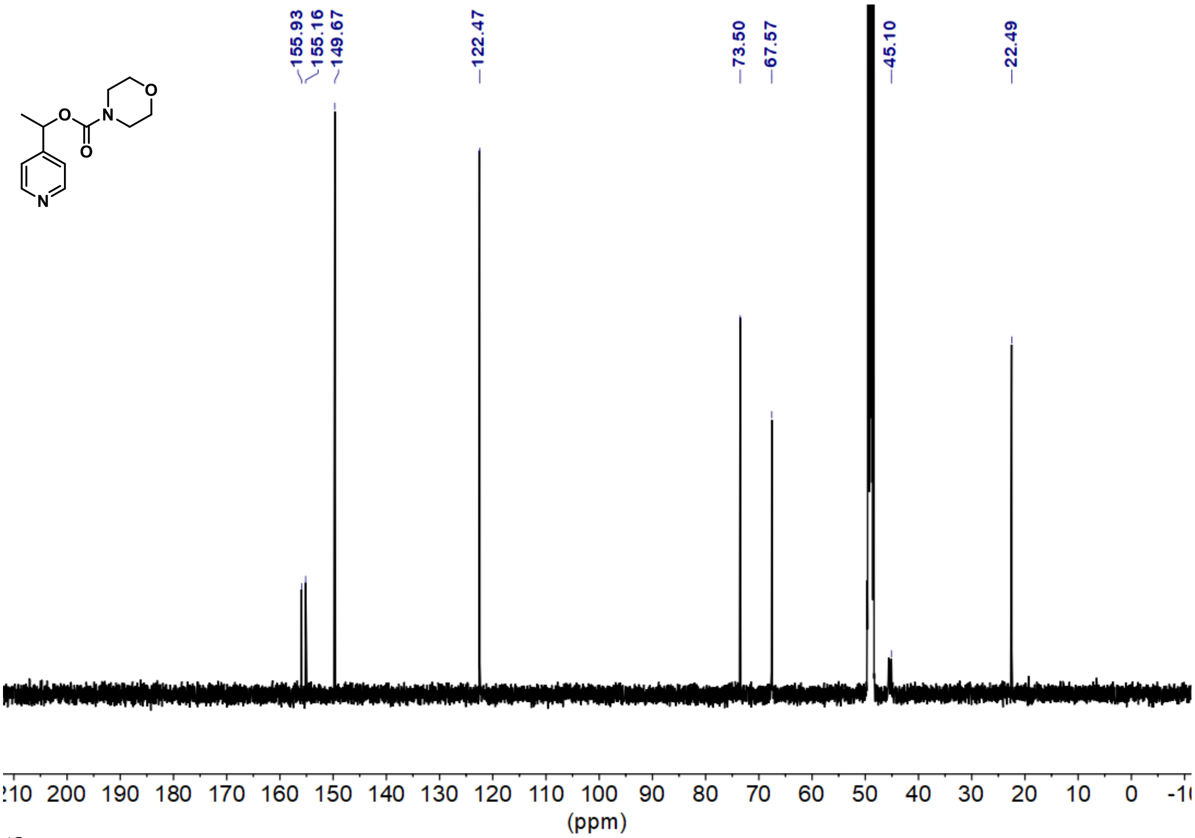

^{13}C NMR (126 MHz, d_4-CD_3OD): **4d**

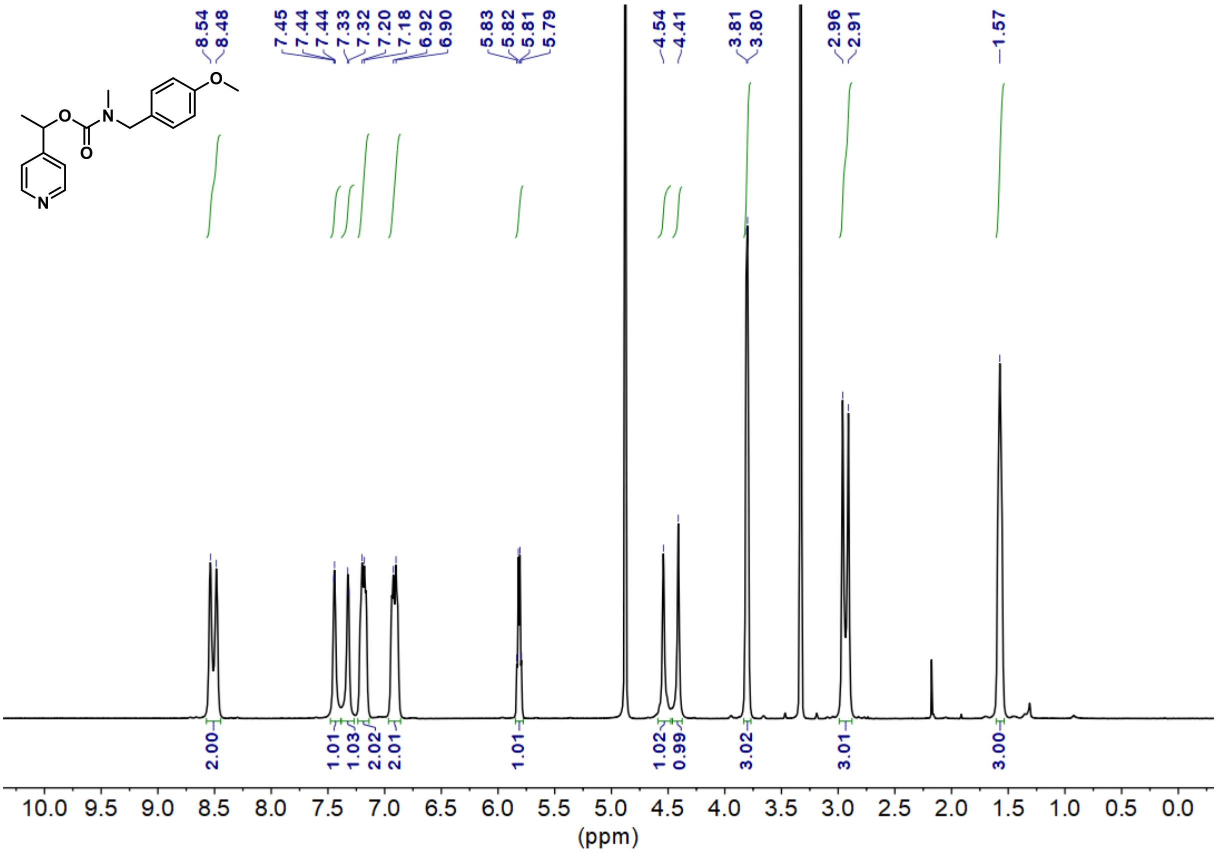

^{1}H NMR (500 MHz, d_4-CD_3OD): **4e**

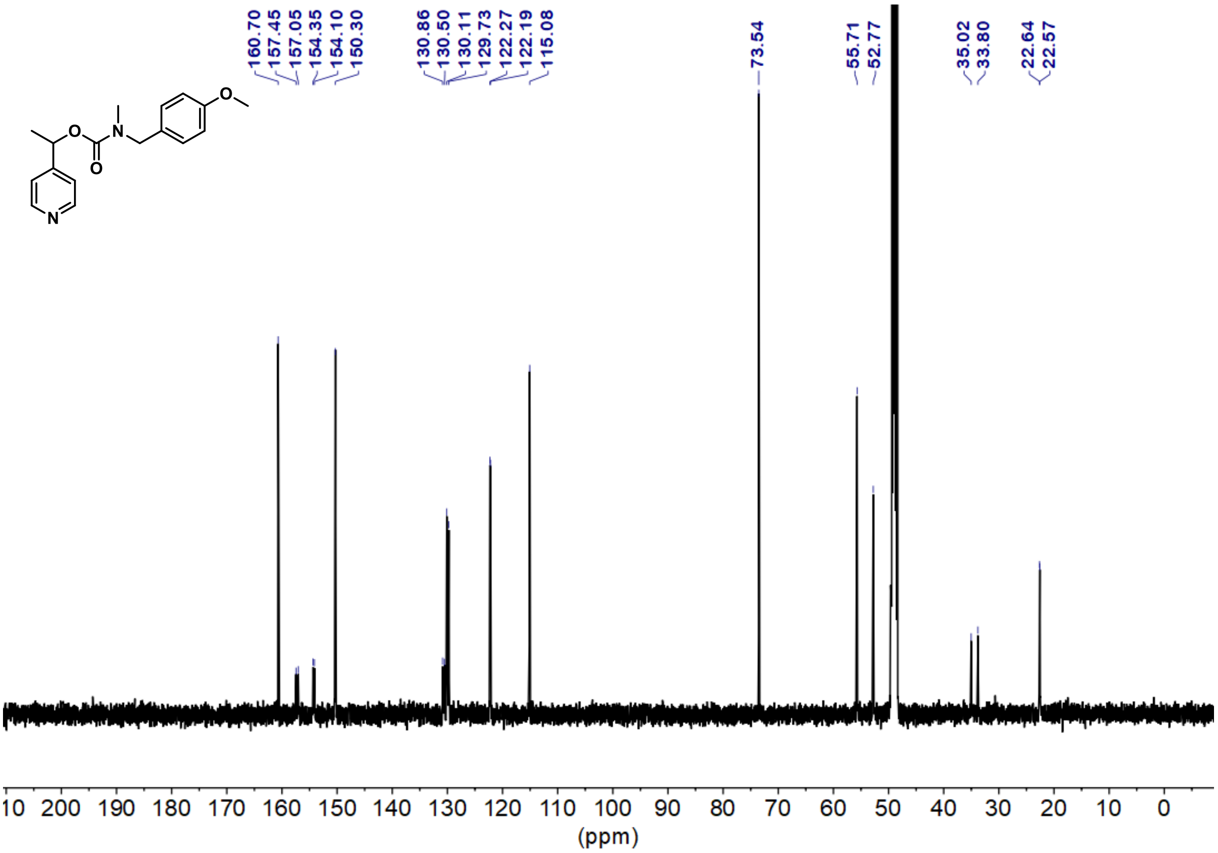

^{13}C NMR (126 MHz, *d*$_4$-CD$_3$OD): **4e**

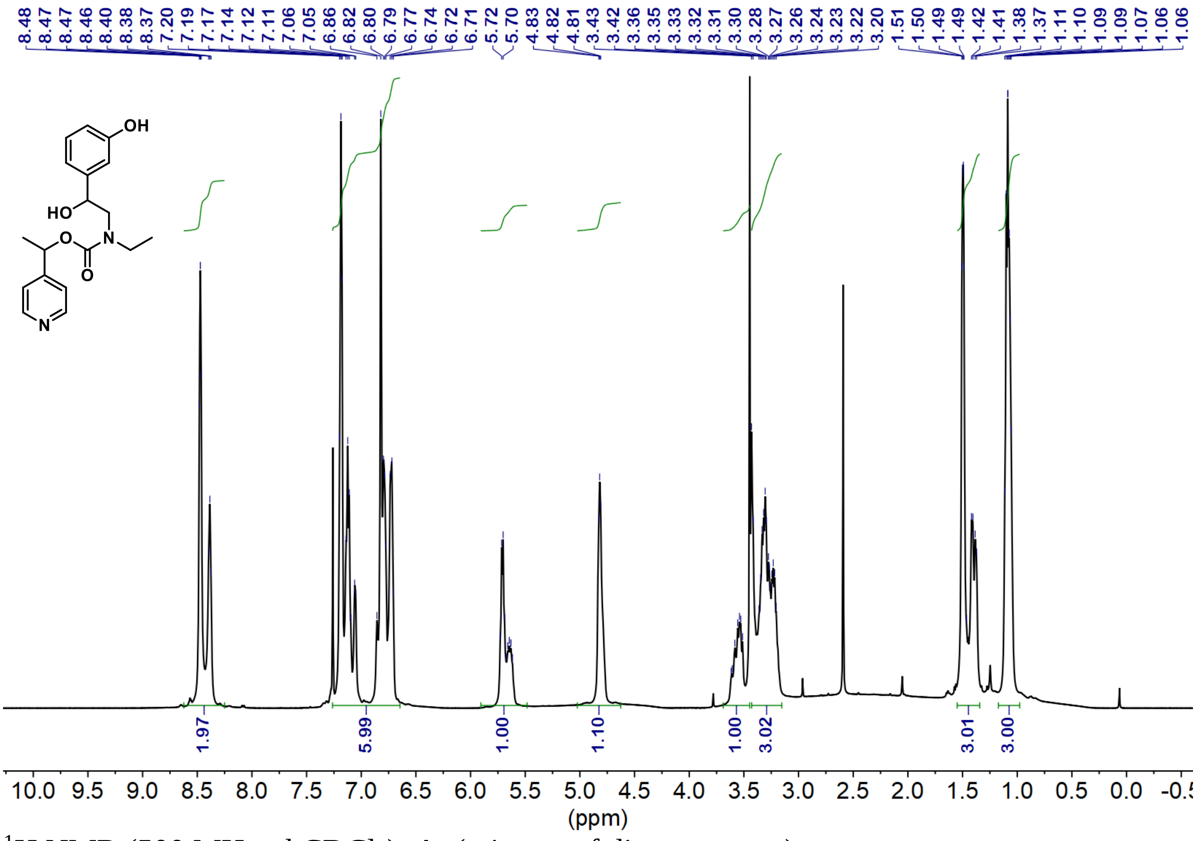

^{1}H NMR (500 MHz, *d*-CDCl$_3$): **4g** (mixture of diastereomers)

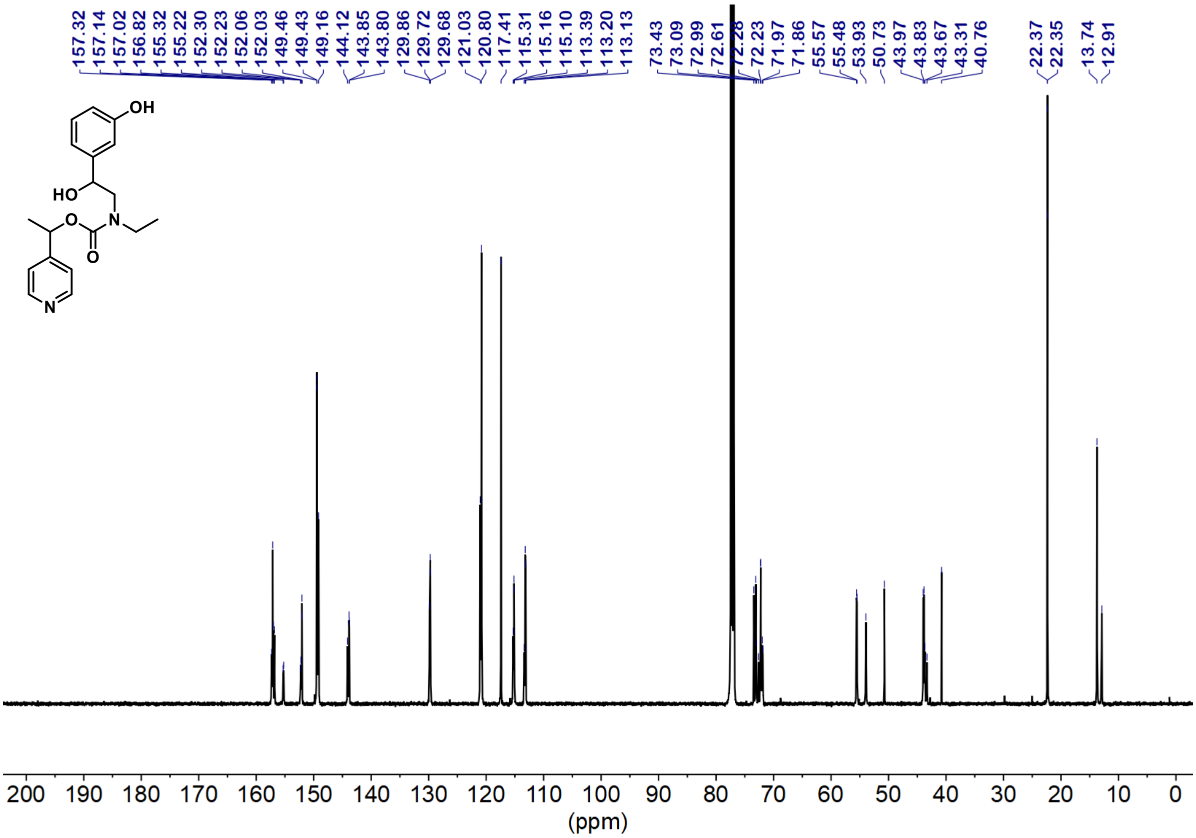

^{1}H NMR (126 MHz, *d*-$CDCl_3$): **4g** (mixture of diastereomers)

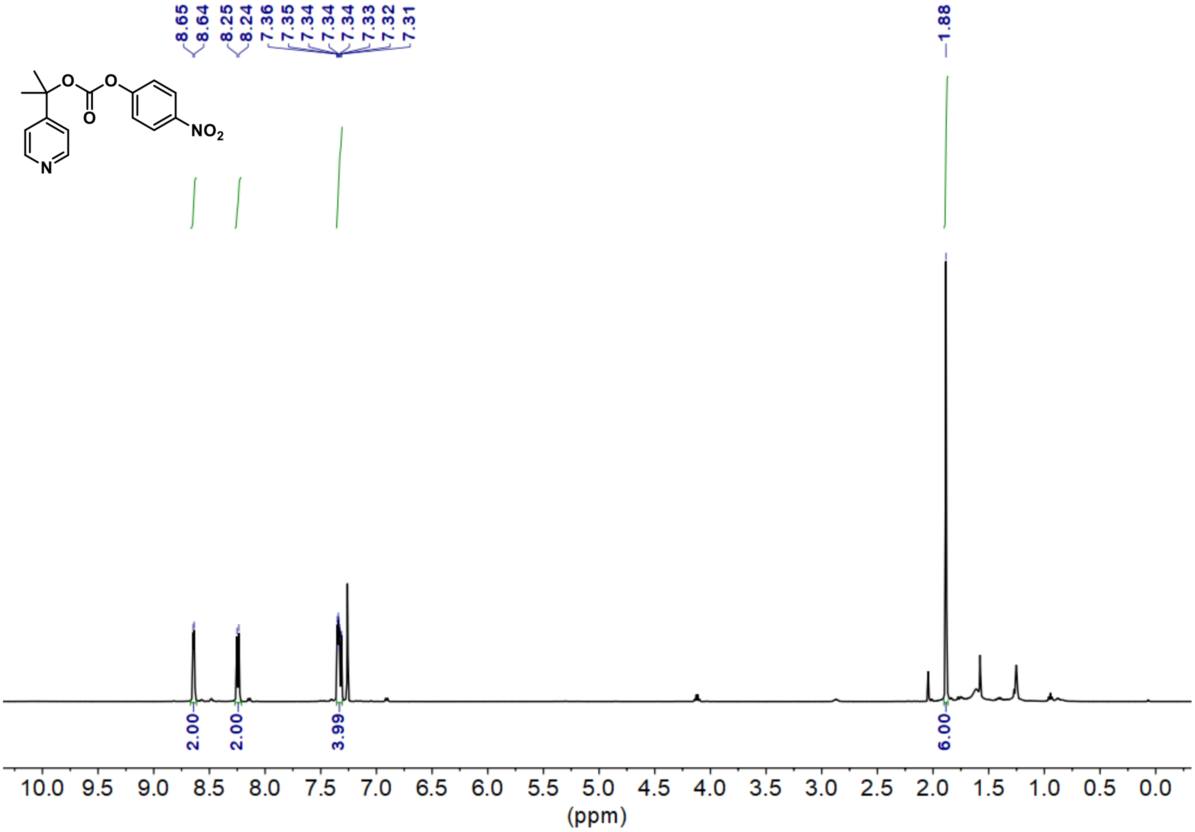

^{1}H NMR (500 MHz, *d*-$CDCl_3$): **11**

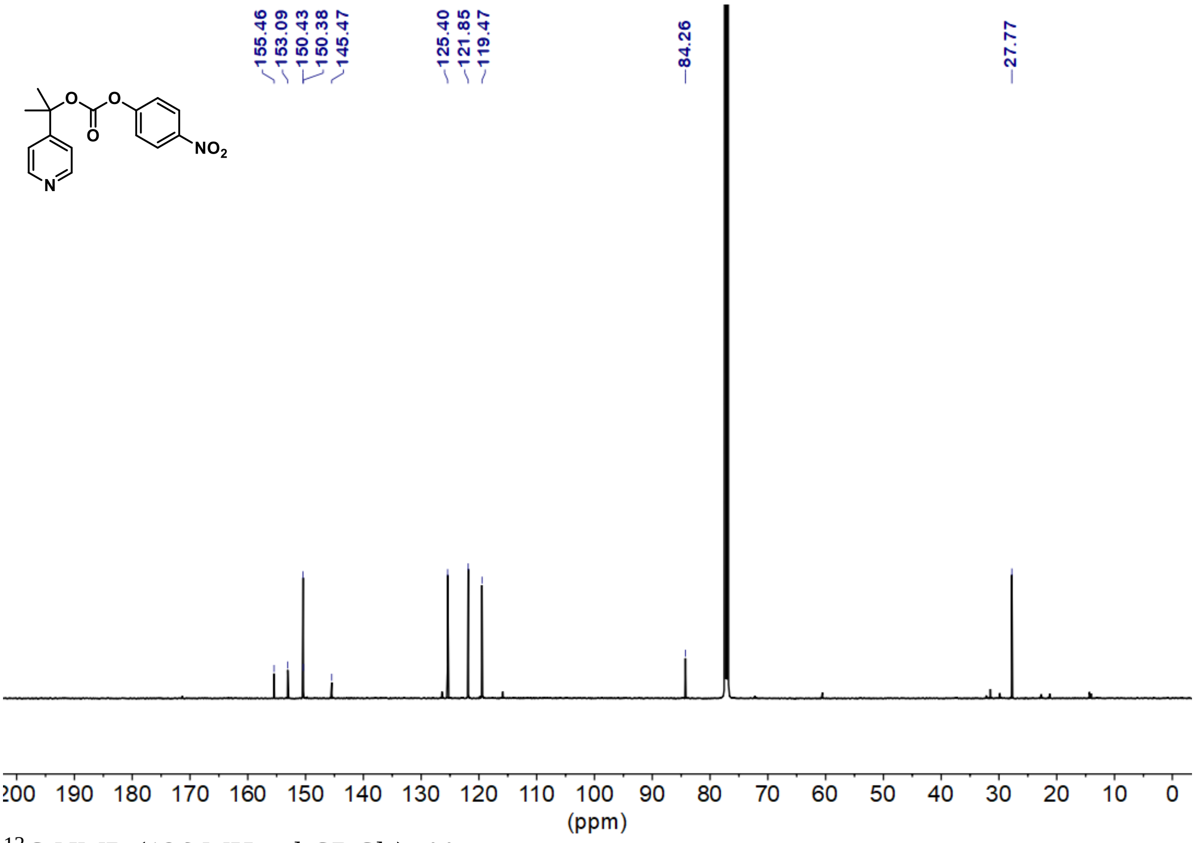

^{13}C NMR (126 MHz, *d*-$CDCl_3$): **11**

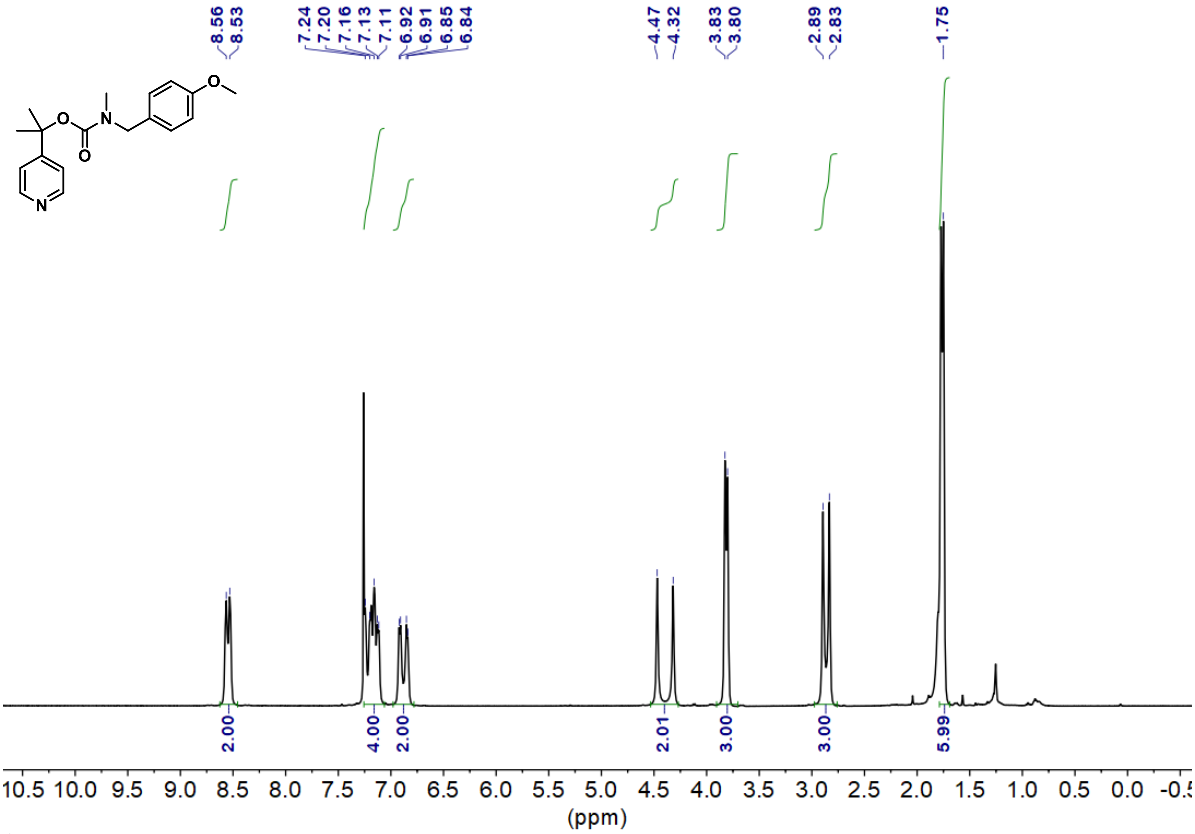

^{1}H NMR (500 MHz, *d*-$CDCl_3$): **4h**

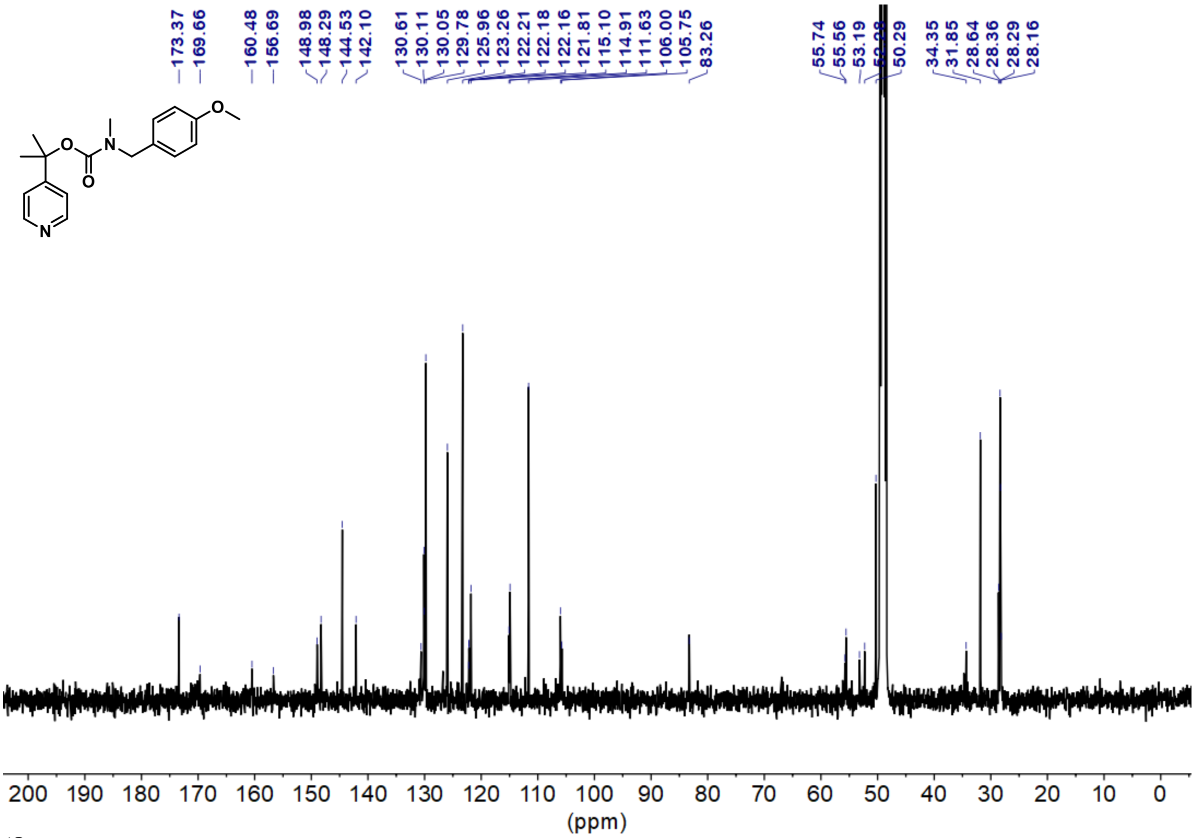

^{13}C NMR (126 MHz, *d*-$CDCl_3$): **4h**

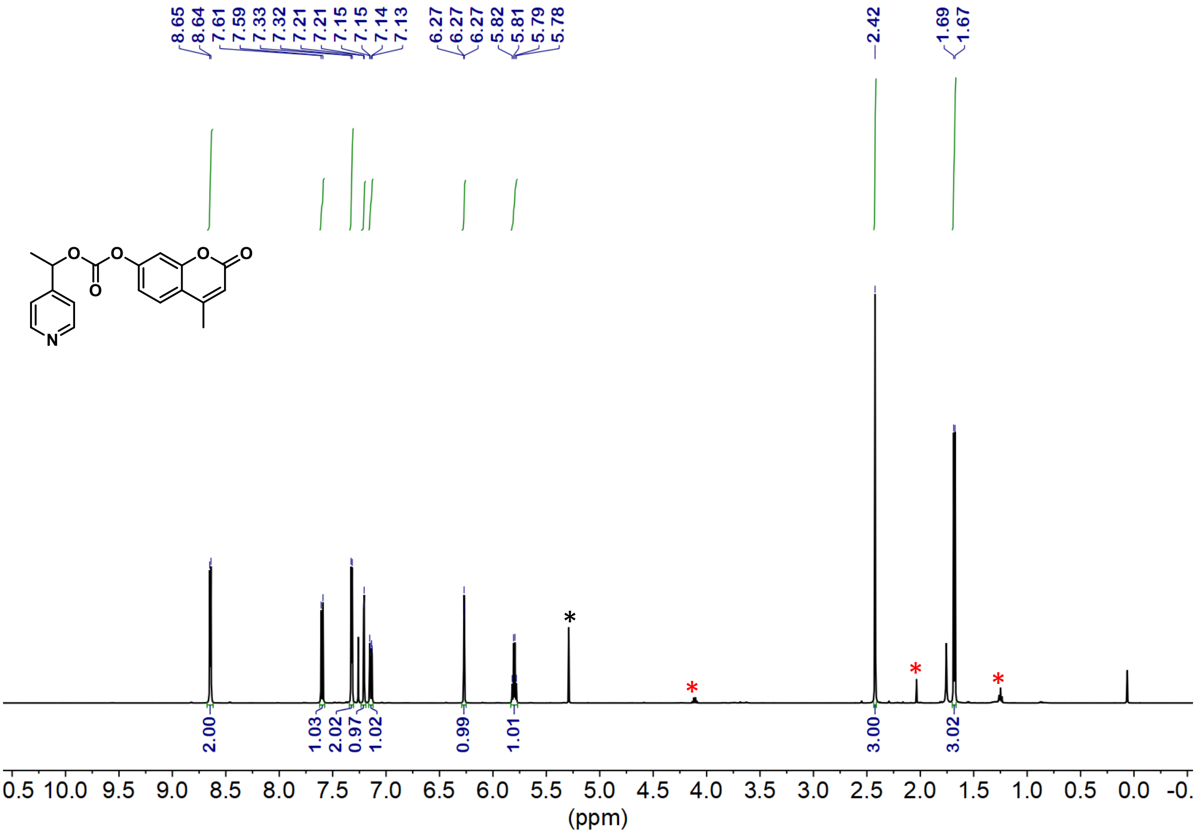

^{1}H NMR (500 MHz, *d*-$CDCl_3$): **4i,** *dichloromethane, *ethylacetate

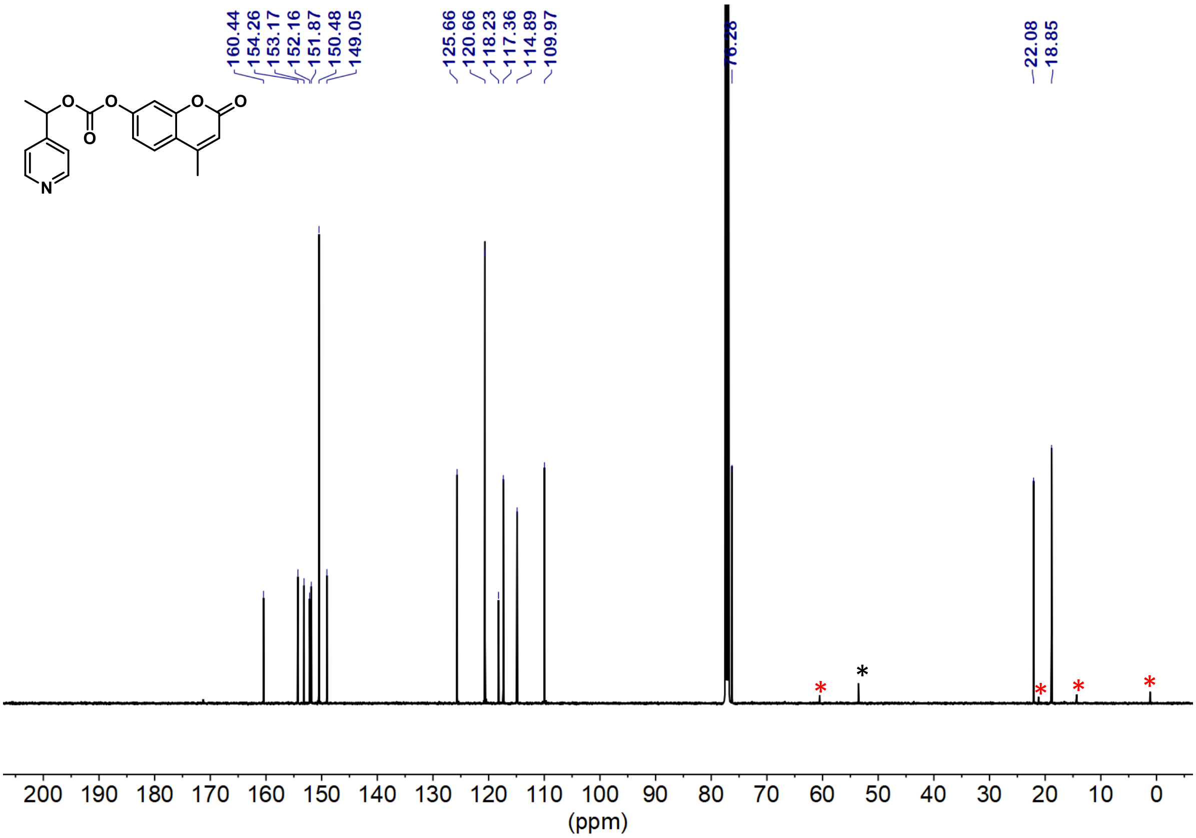

^{13}C NMR (126 MHz, *d*-$CDCl_3$): **4i**, *dichloromethane, *ethylacetate

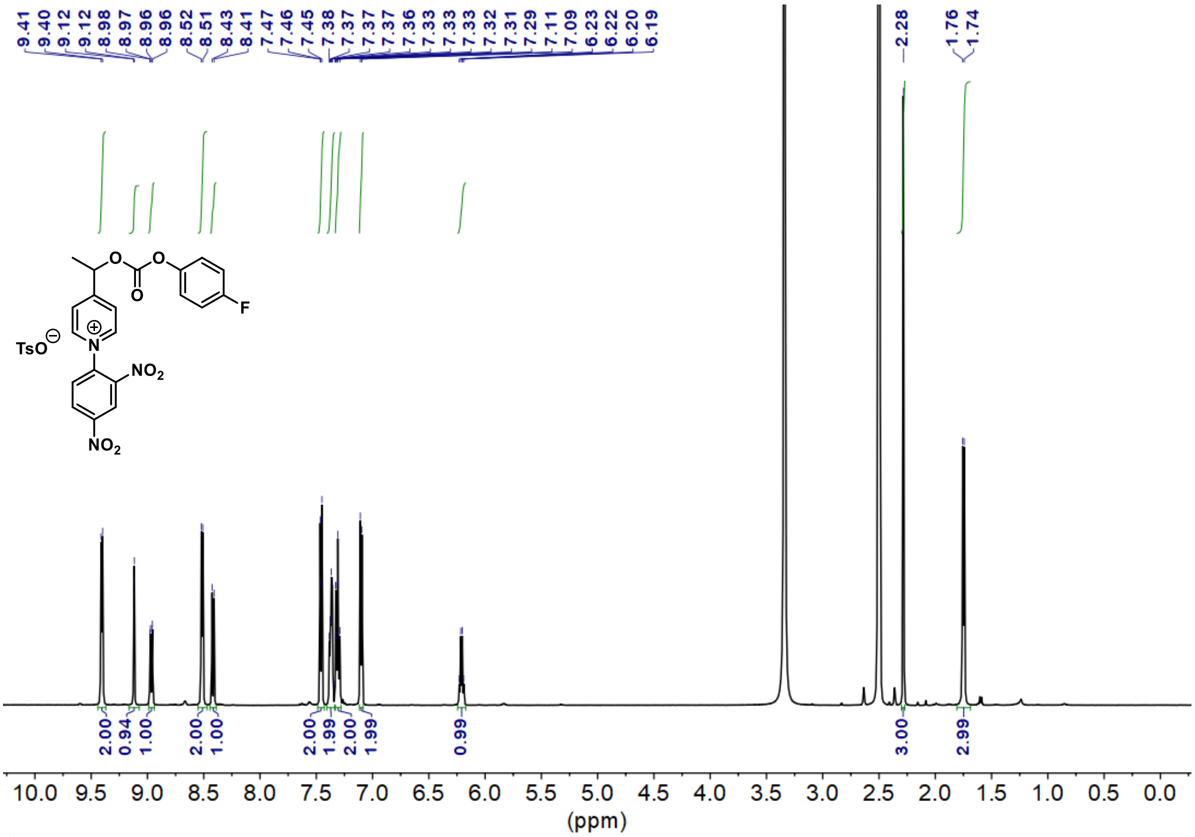

^{1}H NMR (500 MHz, d_6-DMSO): **5a**

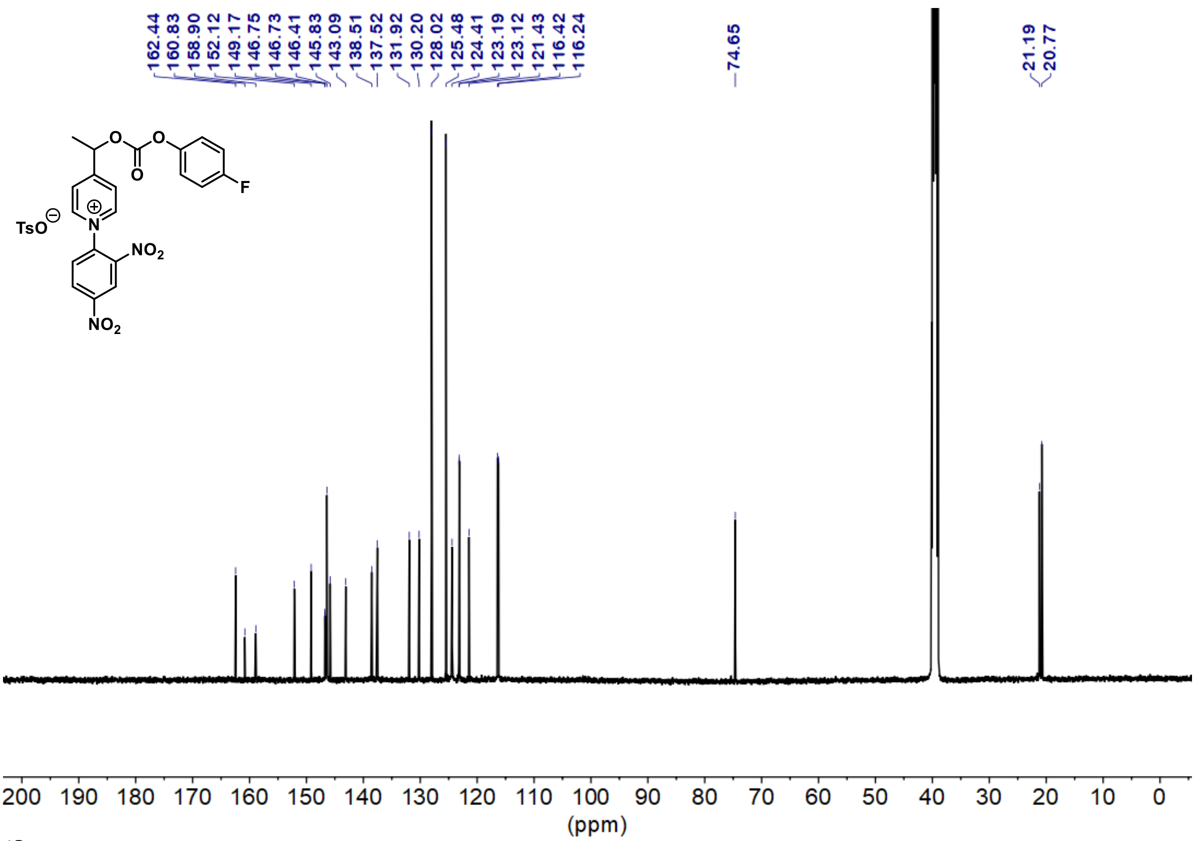

^{13}C NMR (126 MHz, d_6-DMSO): **5a**

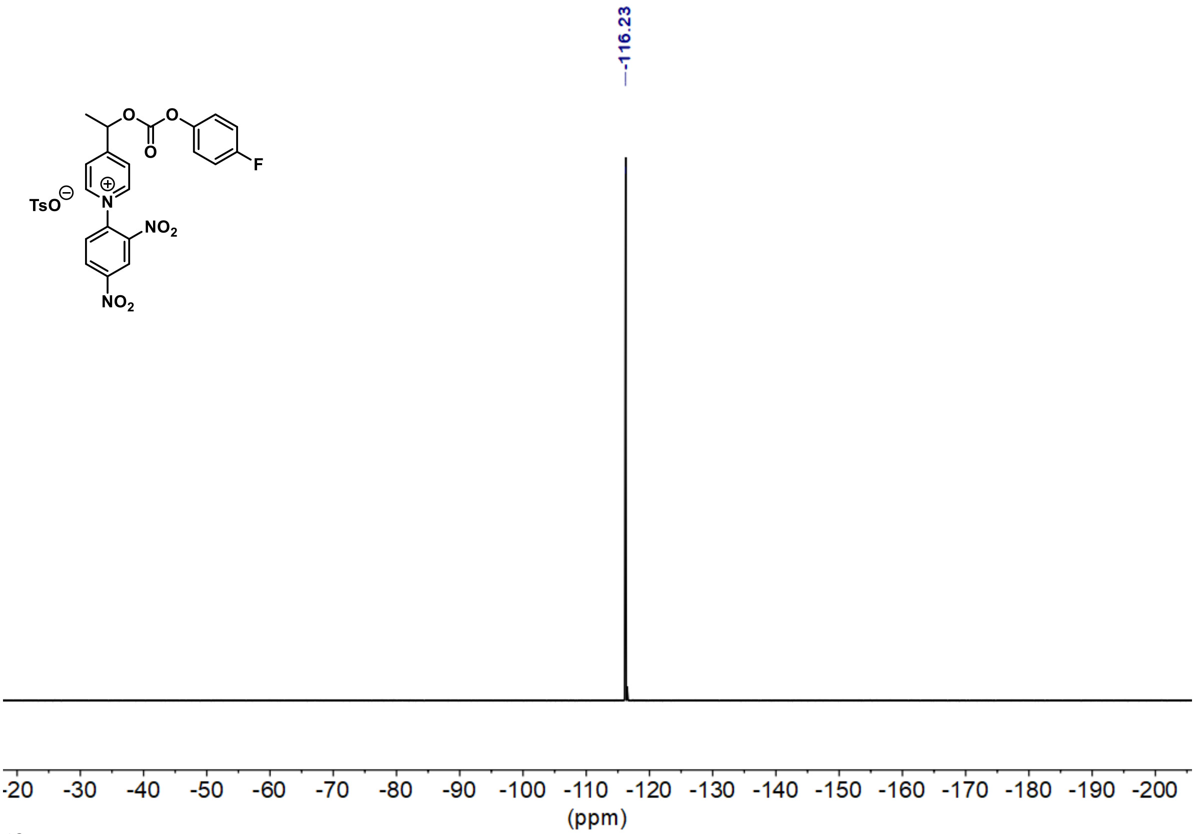

^{19}F NMR (471 MHz, d-$CDCl_3$): **5a**

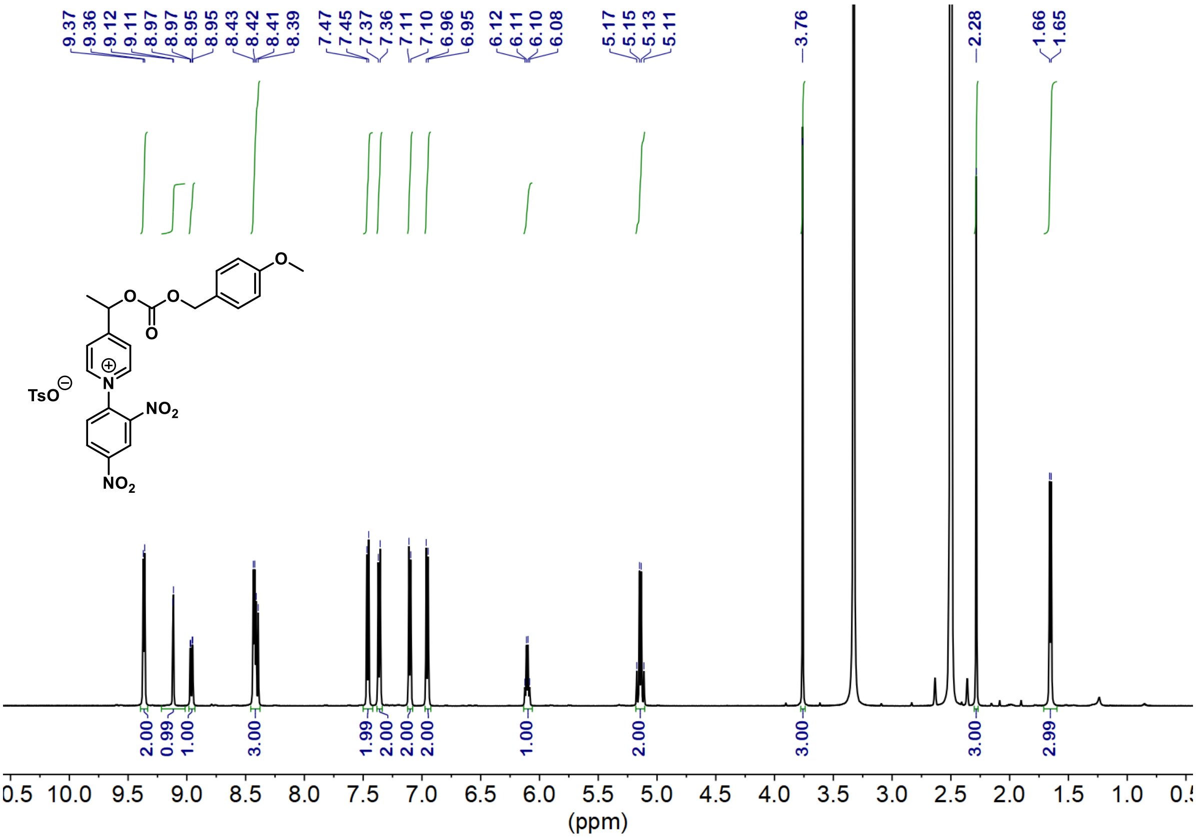

^{1}H NMR (500 MHz, d_6-DMSO): **5b**

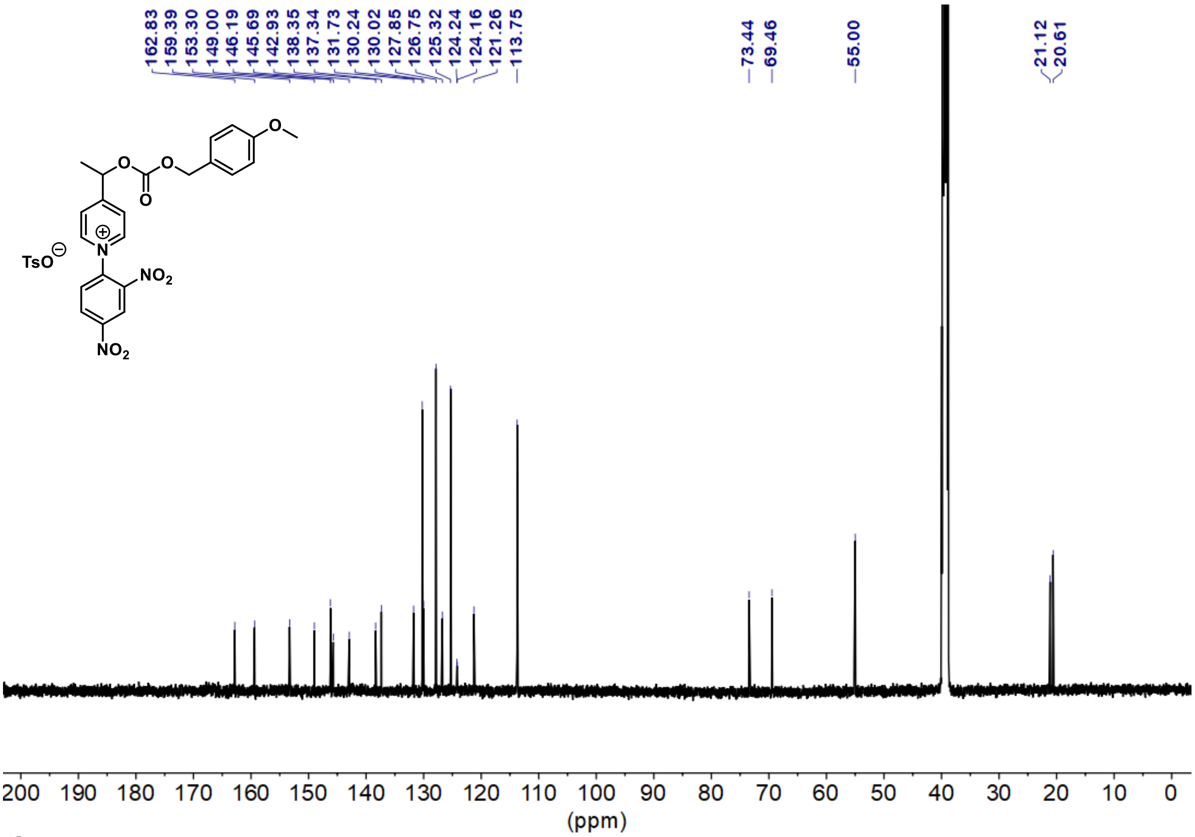

^{13}C NMR (126 MHz, d_6-DMSO): **5b**

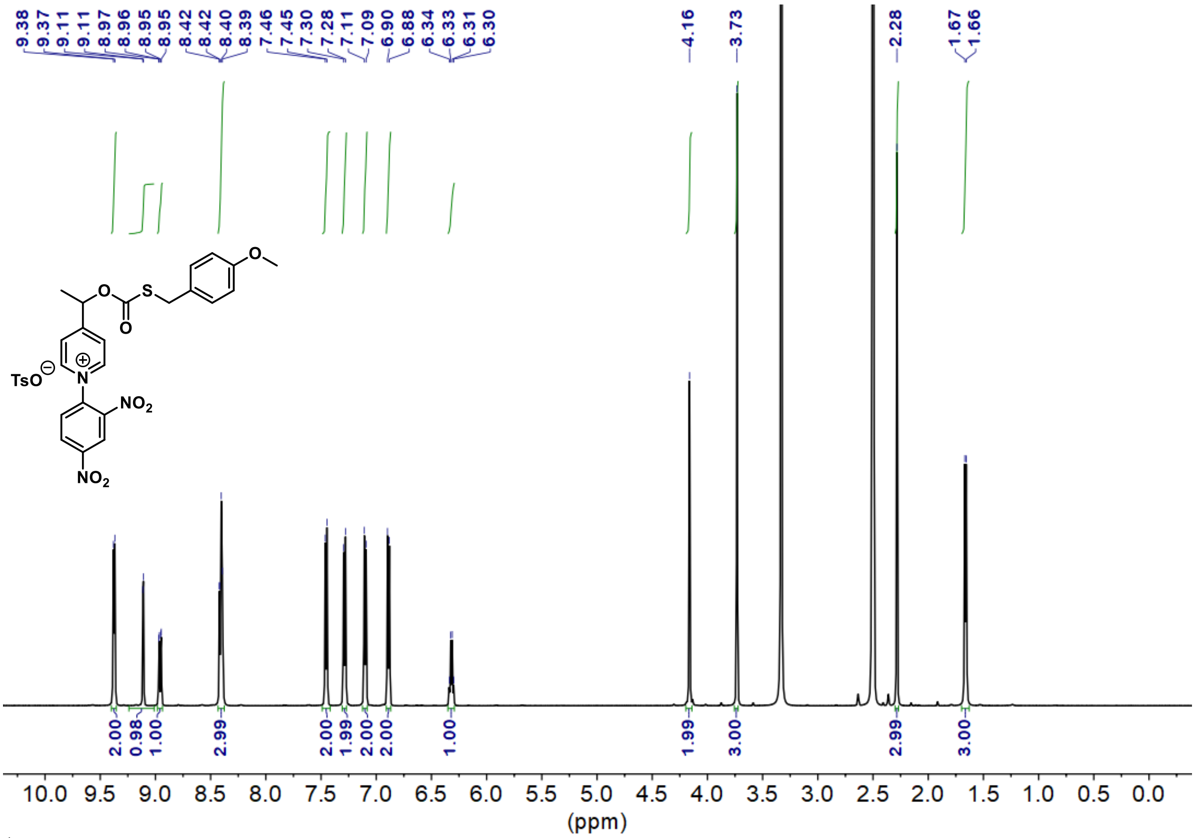

^{1}H NMR (500 MHz, d_6-DMSO): **5c**

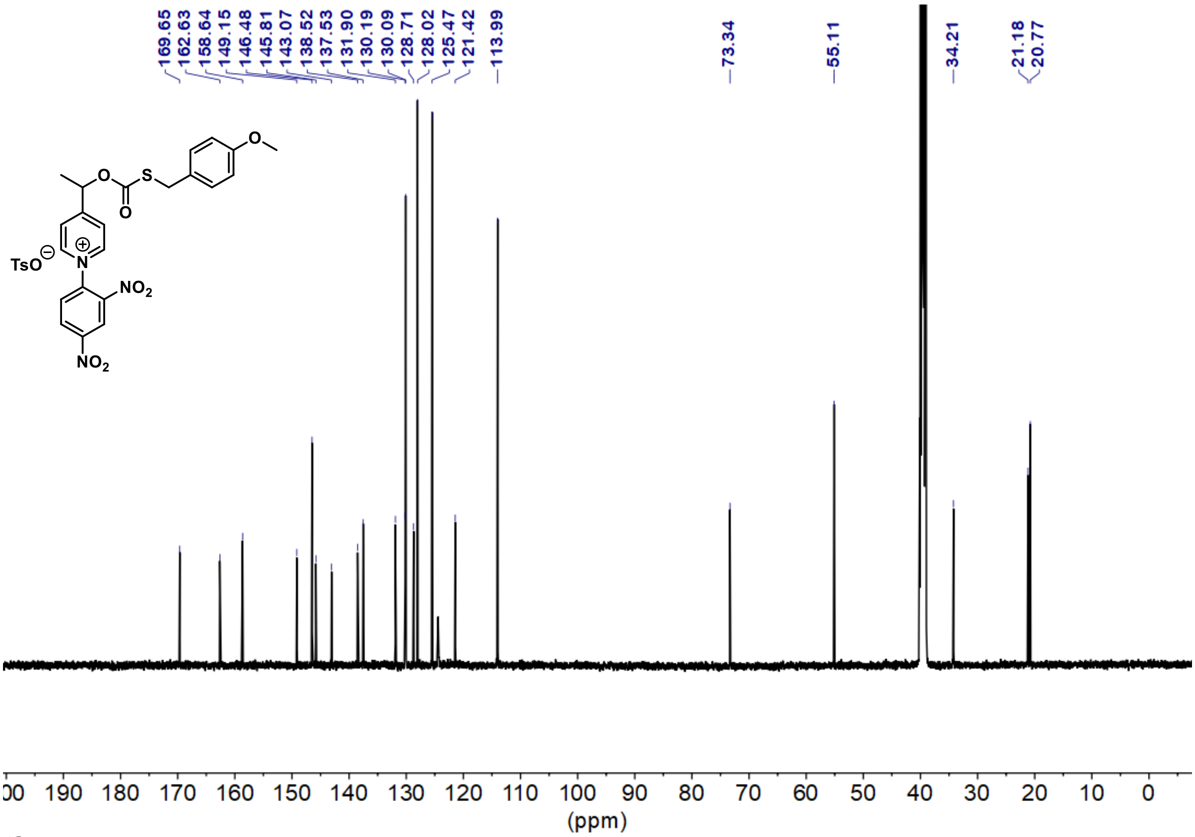

^{13}C NMR (126 MHz, d_6-DMSO): **5c**

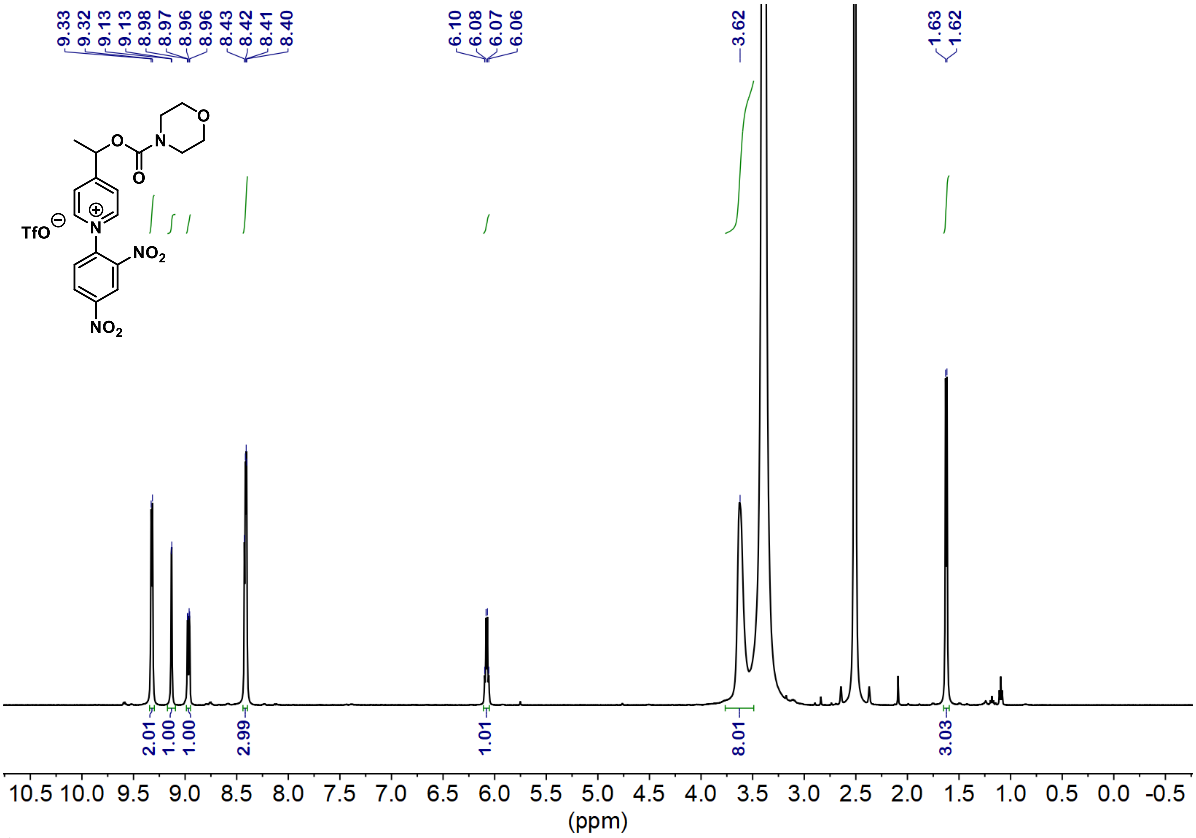

^{1}H NMR (500 MHz, d_6-DMSO): **5d**

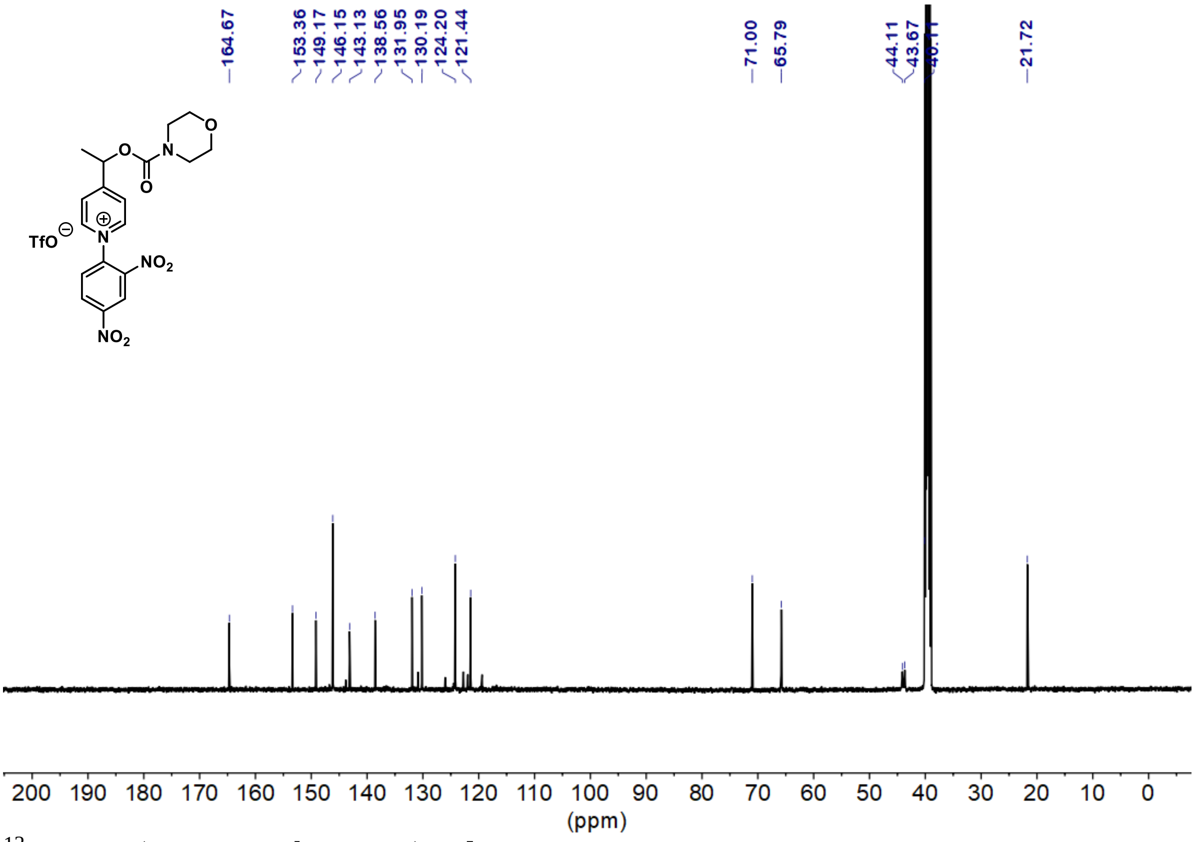

^{13}C NMR (126 MHz, d_6-DMSO): **5d**

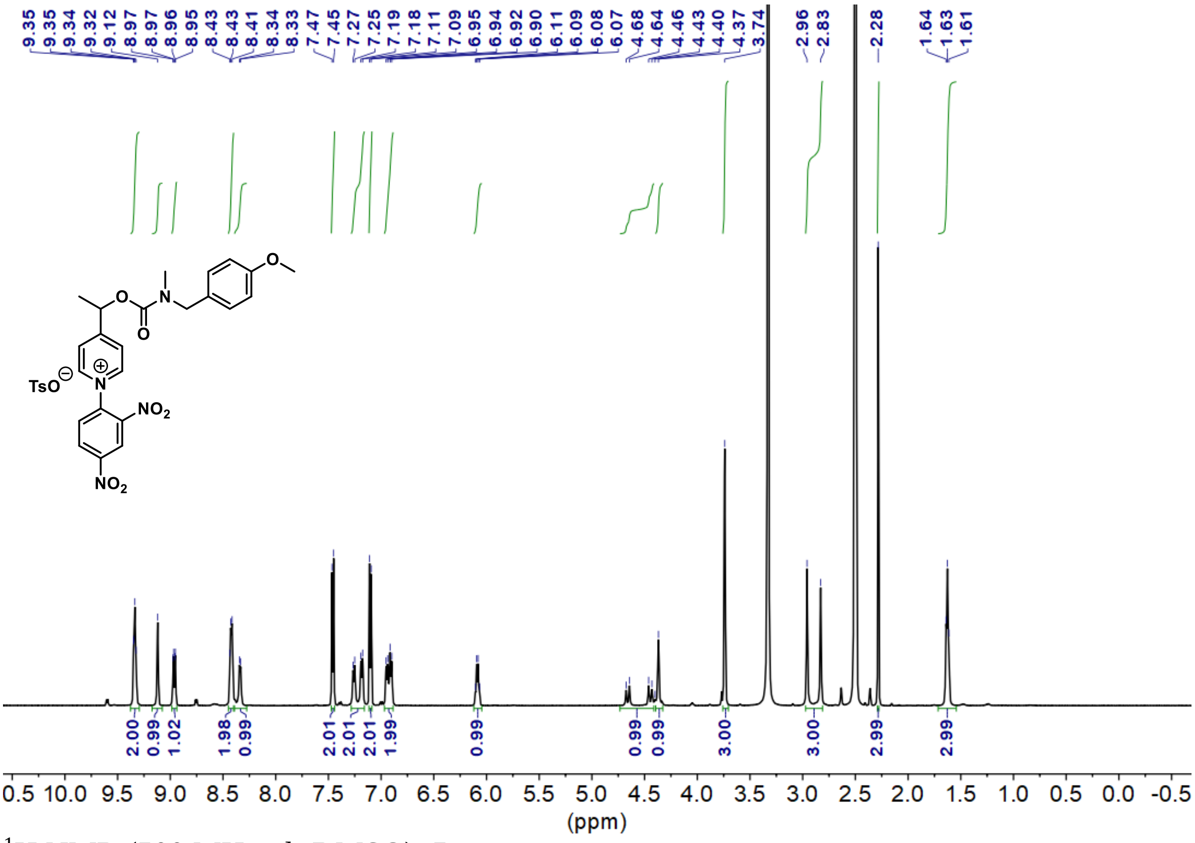

^{1}H NMR (500 MHz, d_6-DMSO): **5e**

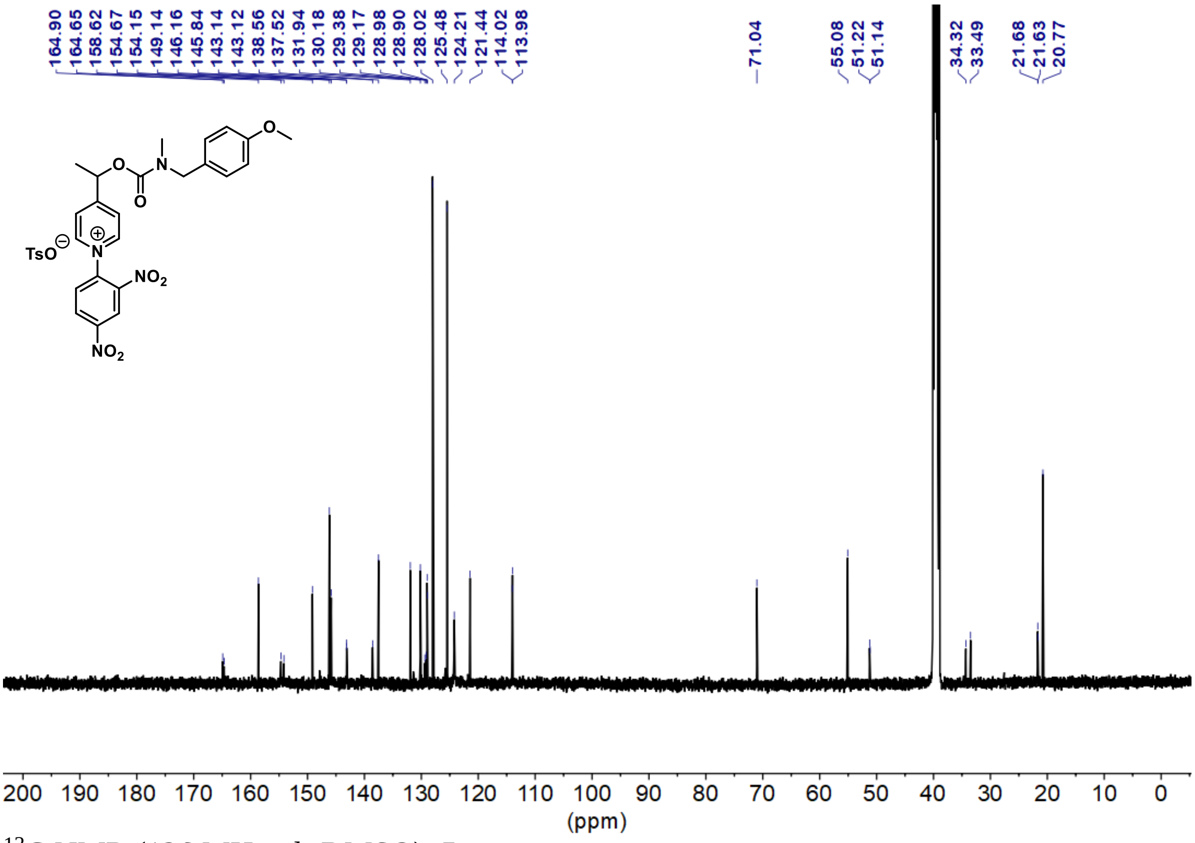

^{13}C NMR (126 MHz, d_6-DMSO): **5e**

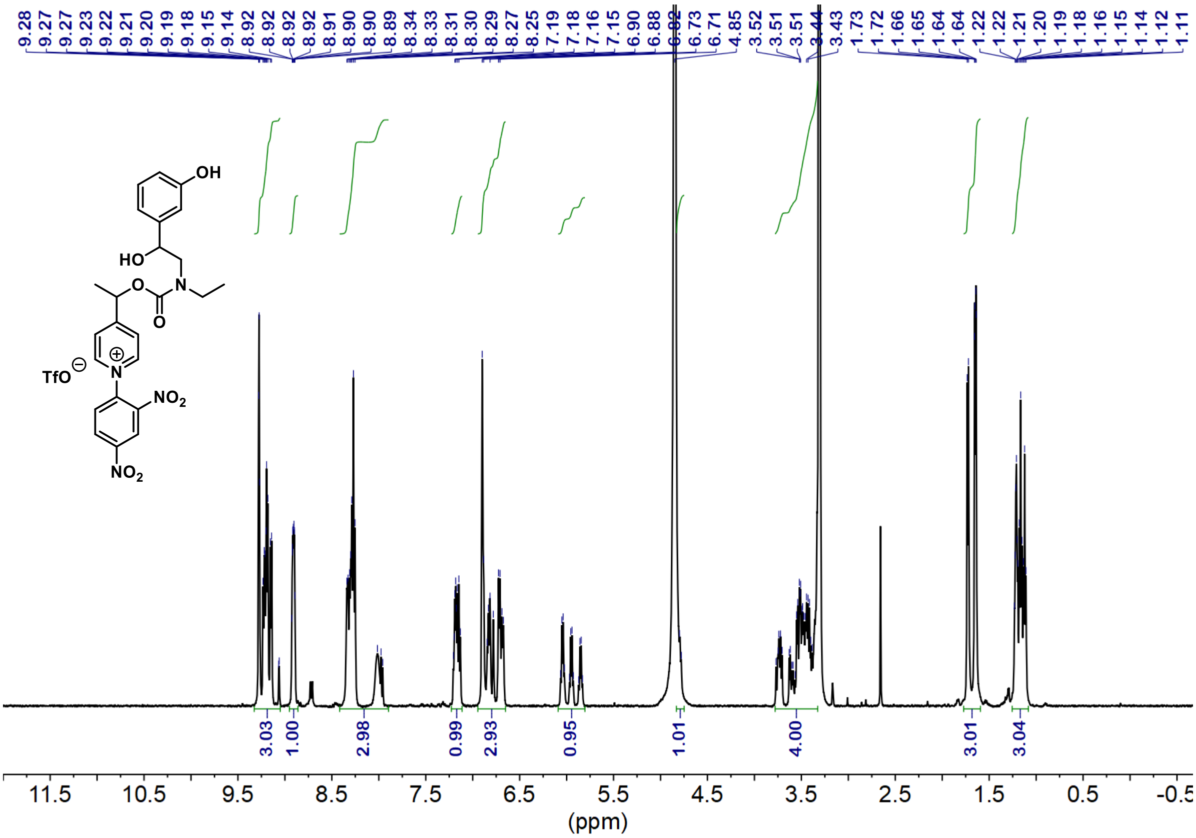

^{1}H NMR (500 MHz, *d*4-CD3OD): **5g** (mixture of diastereomers)

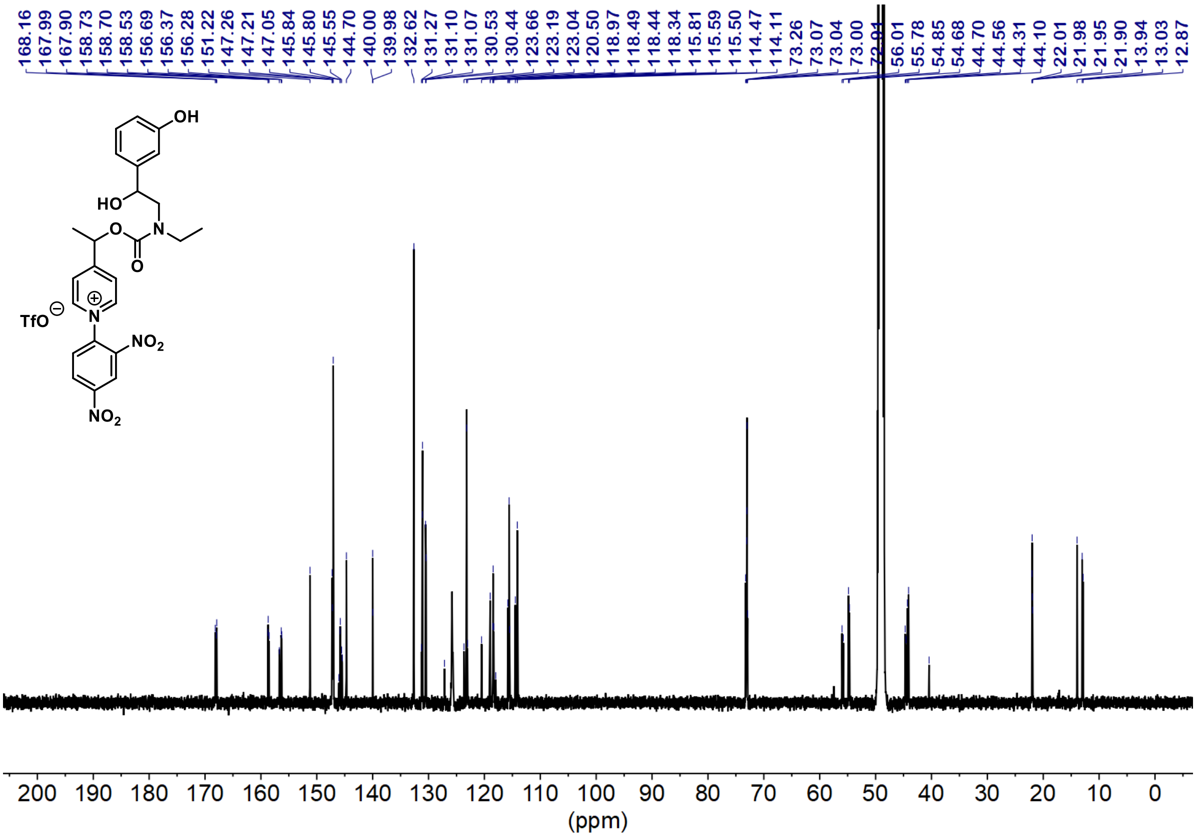

^{13}C NMR (126 MHz, *d*4-CD3OD): **5g** (mixture of diastereomers)

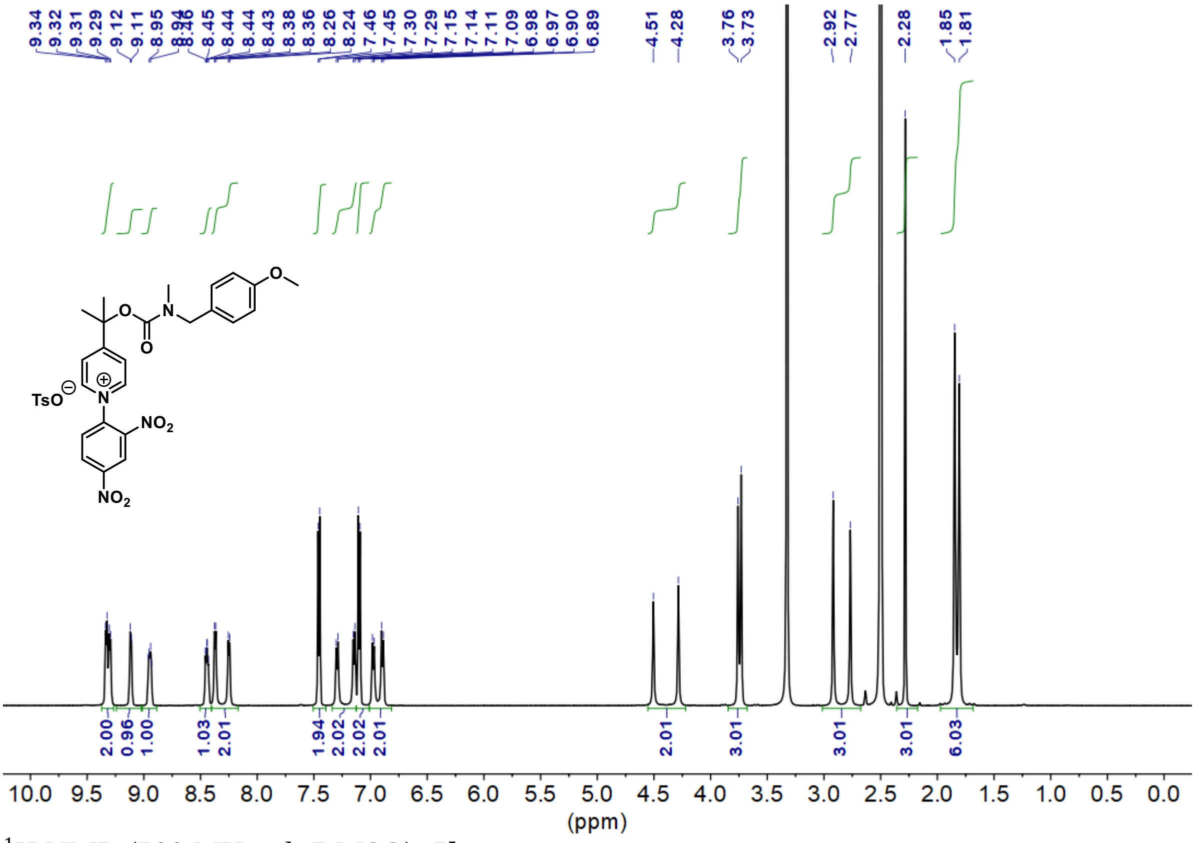

^{1}H NMR (500 MHz, d_6-DMSO): **5h**

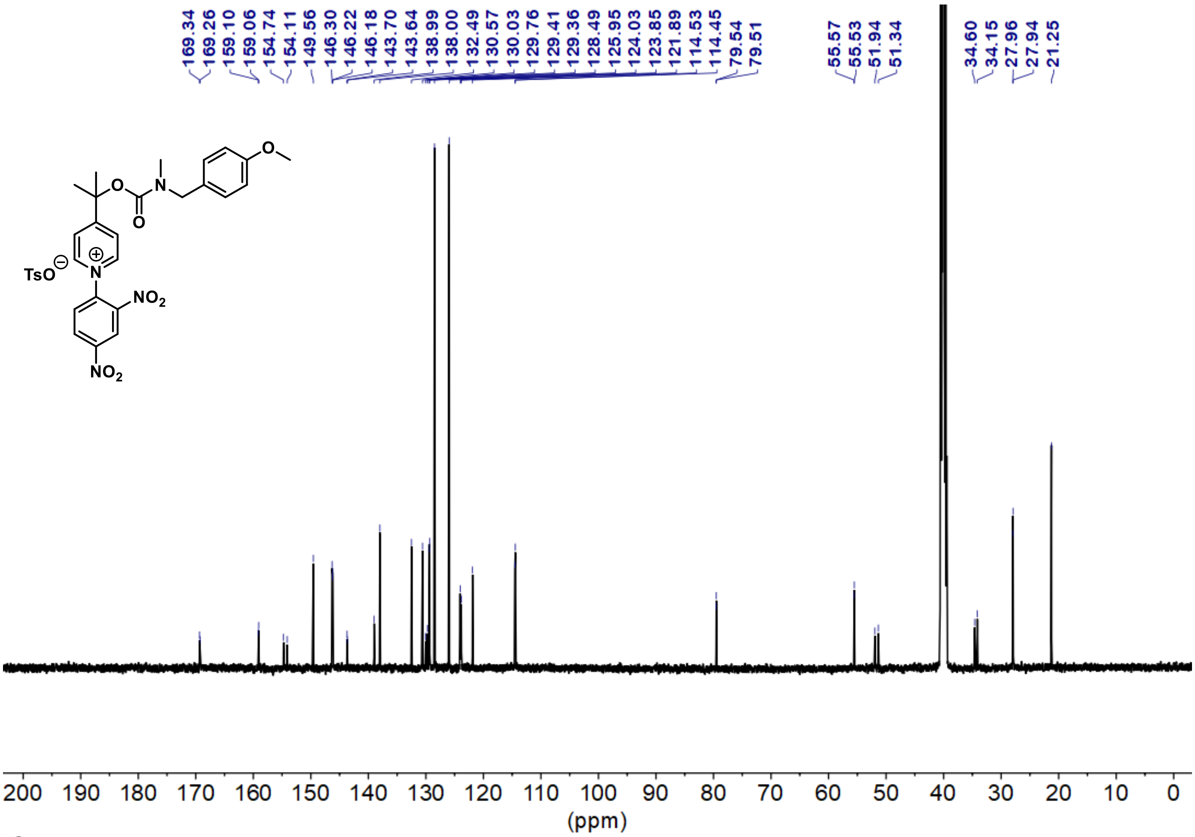

^{13}C NMR (126 MHz, d_6-DMSO): **5h**

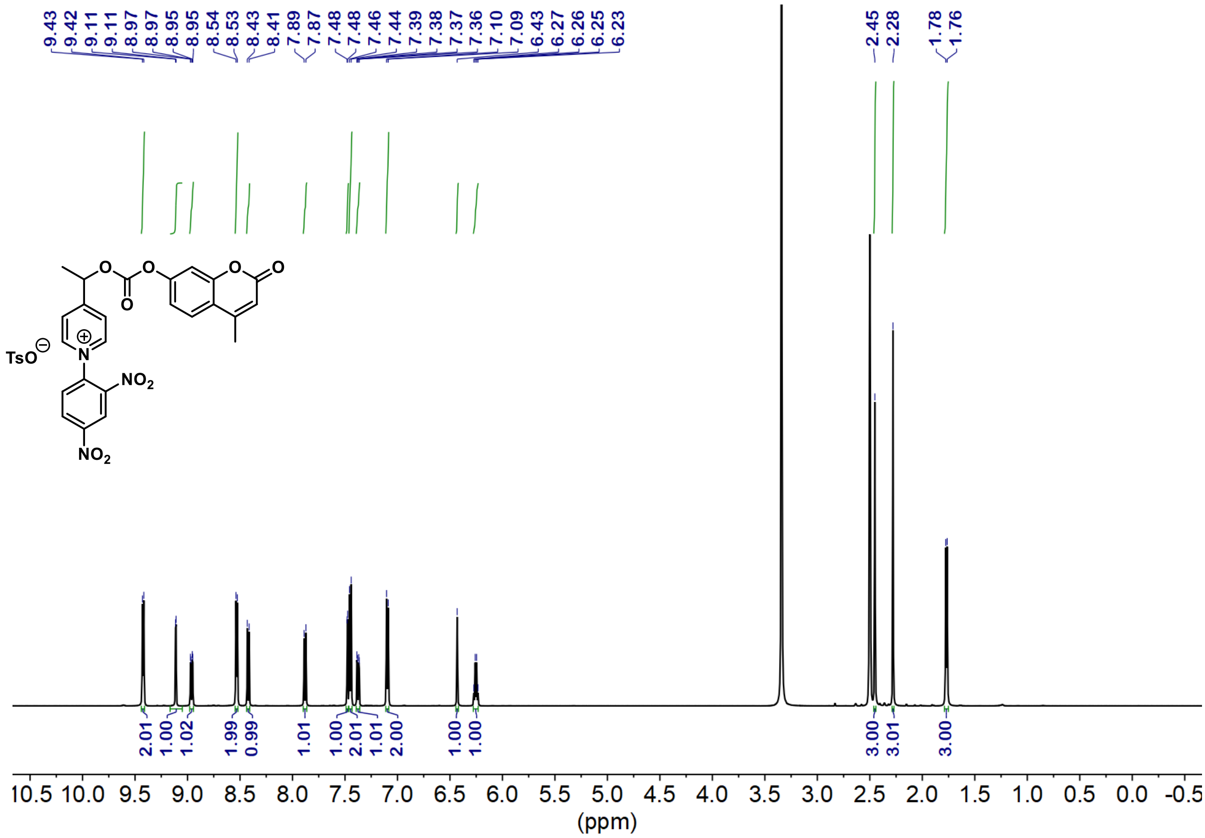

Figure SX. ^{1}H NMR (500 MHz, d_6-DMSO): **5i**

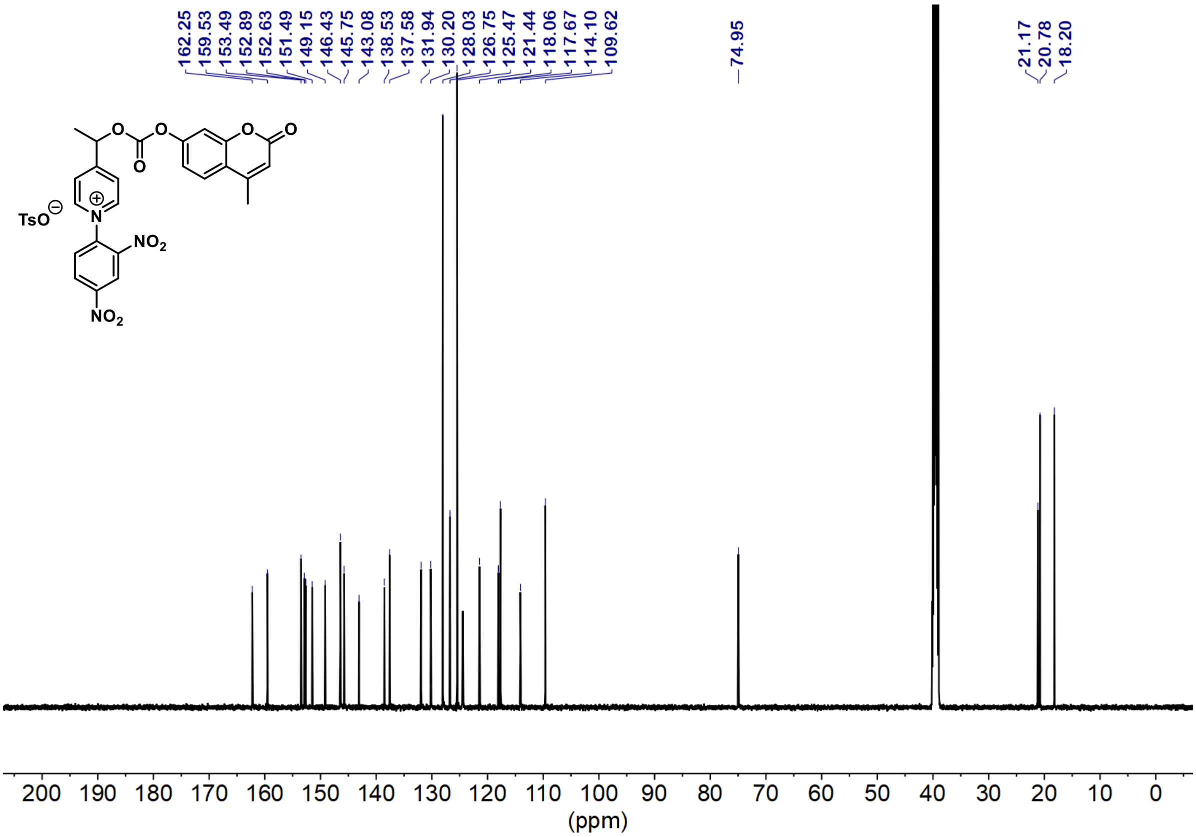

Figure SX. ^{13}C NMR (126 MHz, d_6-DMSO): **5i**

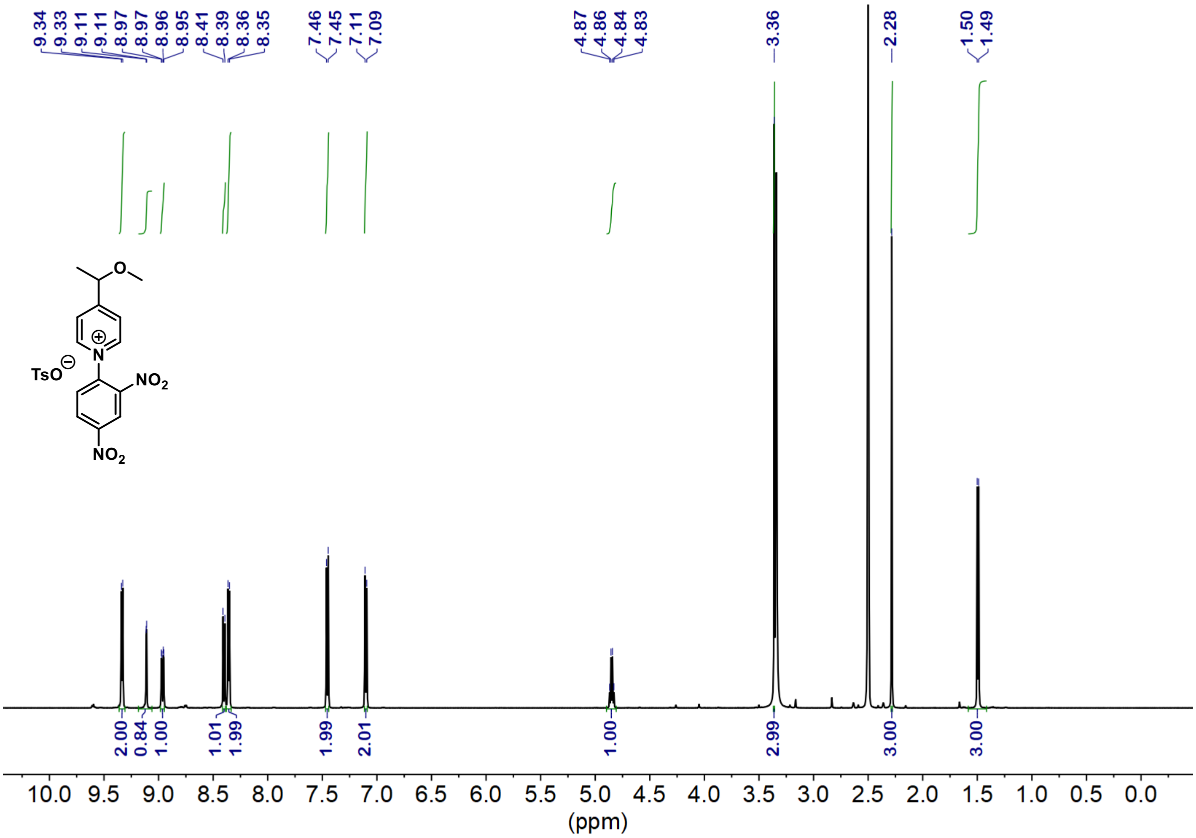

^{1}H NMR (500 MHz, d_6-DMSO): **15**

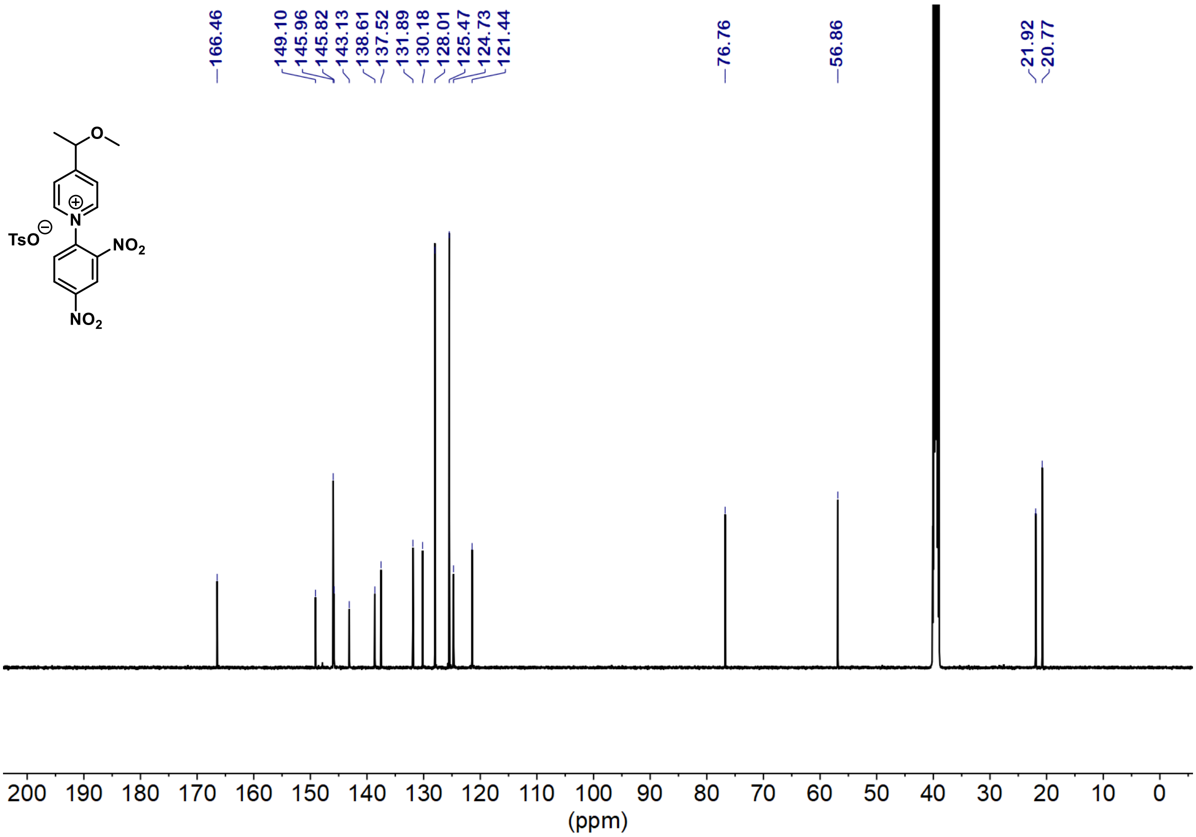

^{13}C NMR (126 MHz, d_6-DMSO): **15**

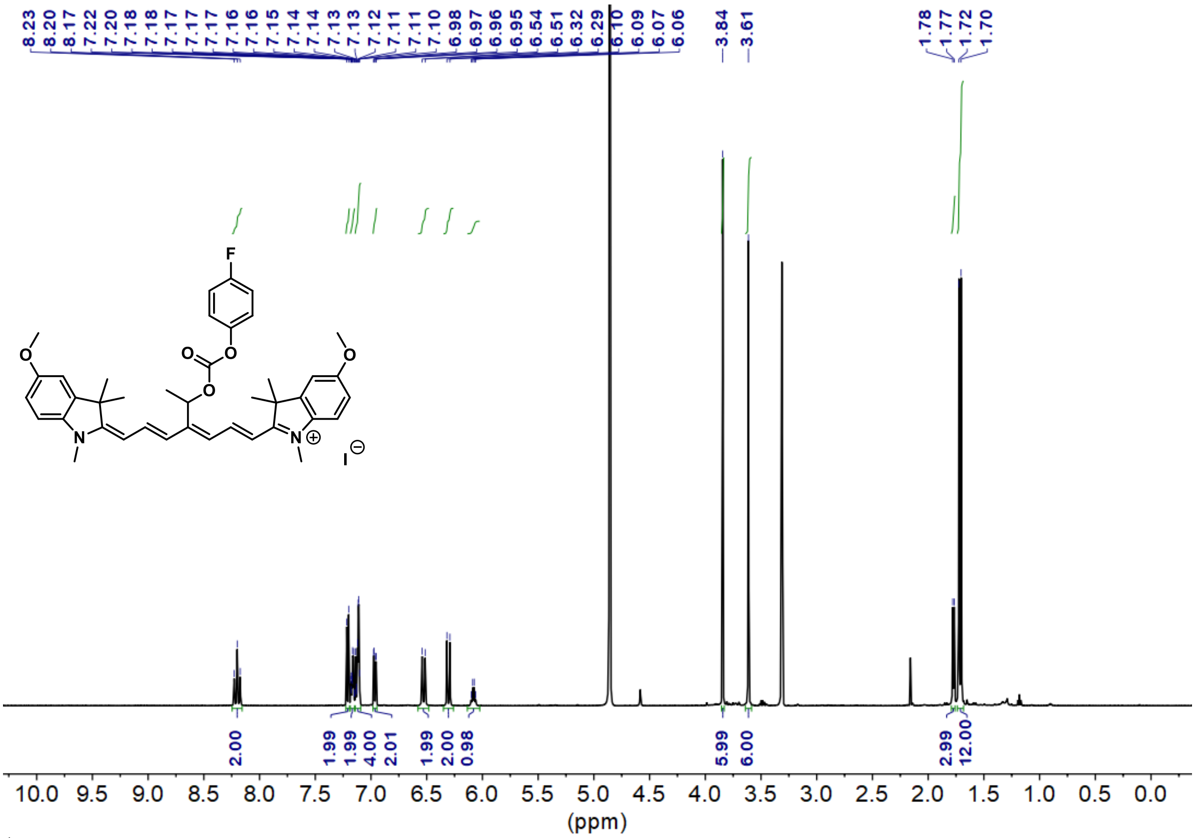

^{1}H NMR (500 MHz, d_4-CD_3OD): **1a**

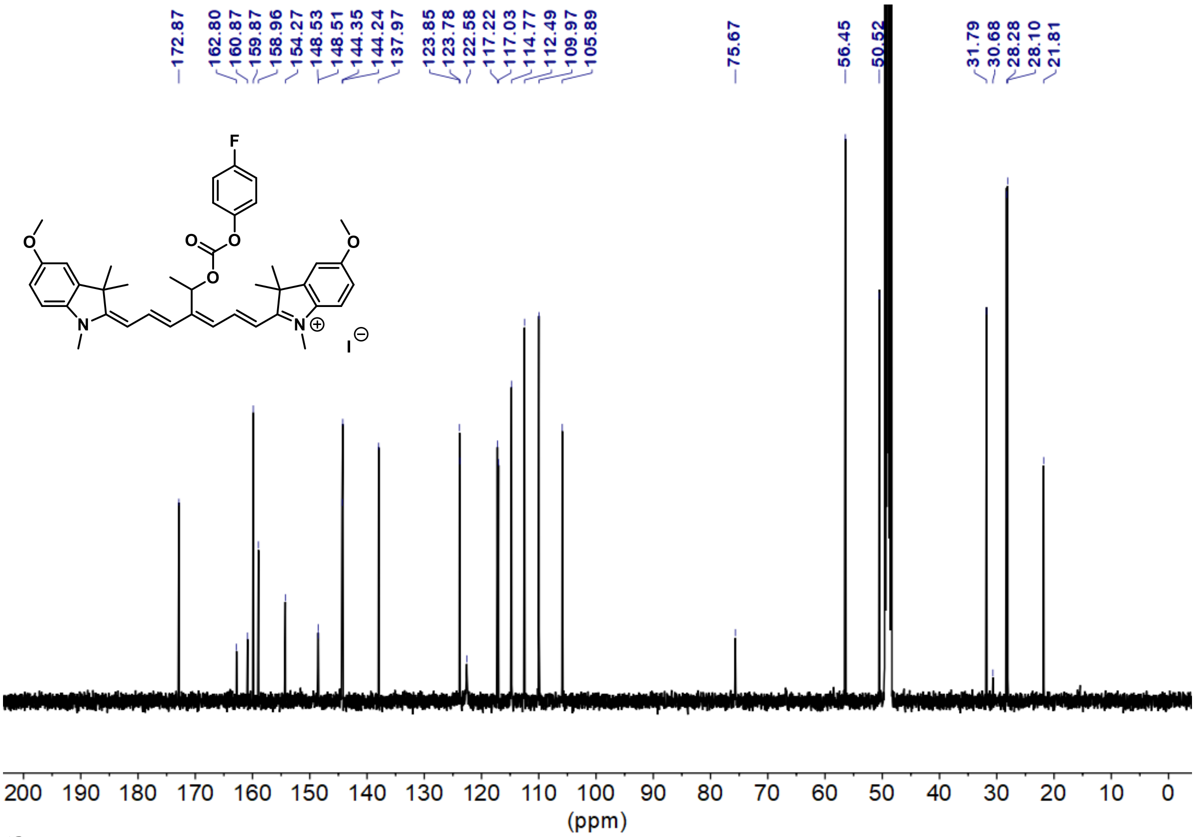

^{13}C NMR (126 MHz, d_4-CD_3OD): **1a**

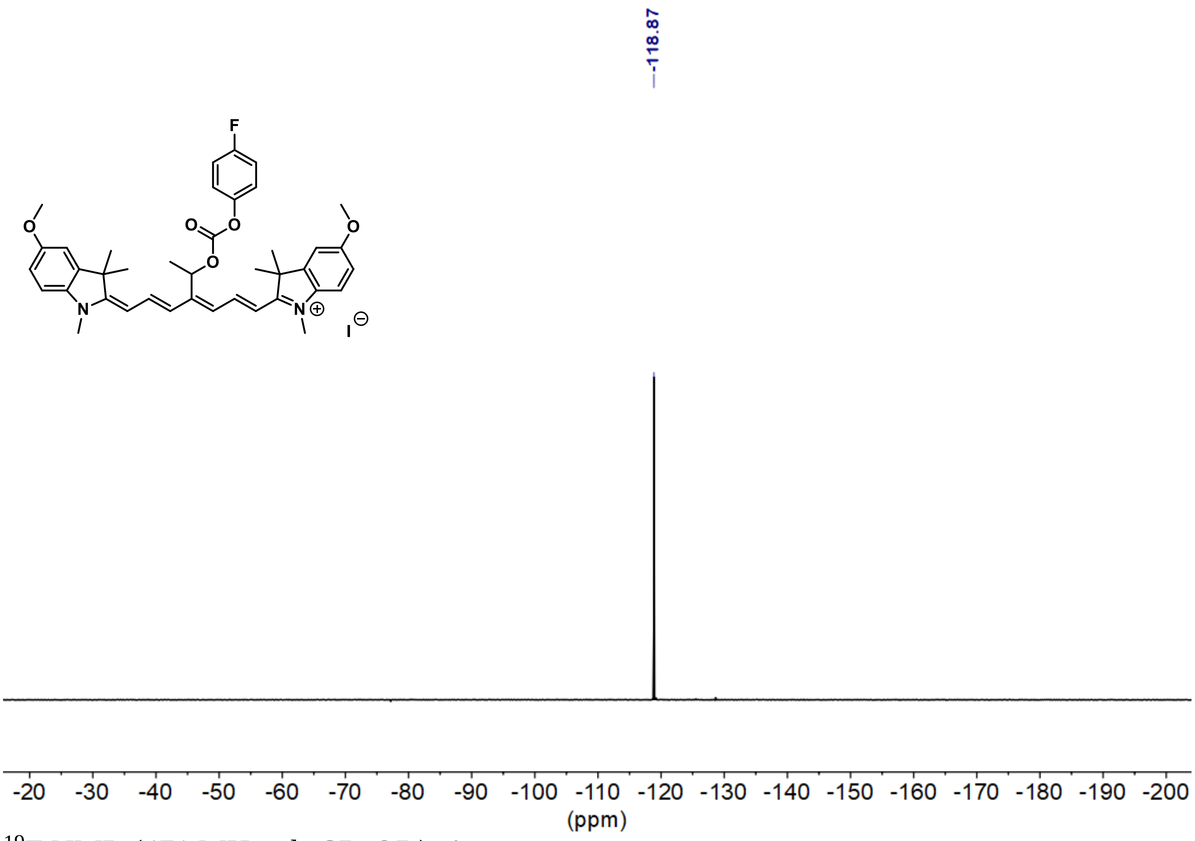

^{19}F NMR (471 MHz, d_4-CD_3OD): **1a**

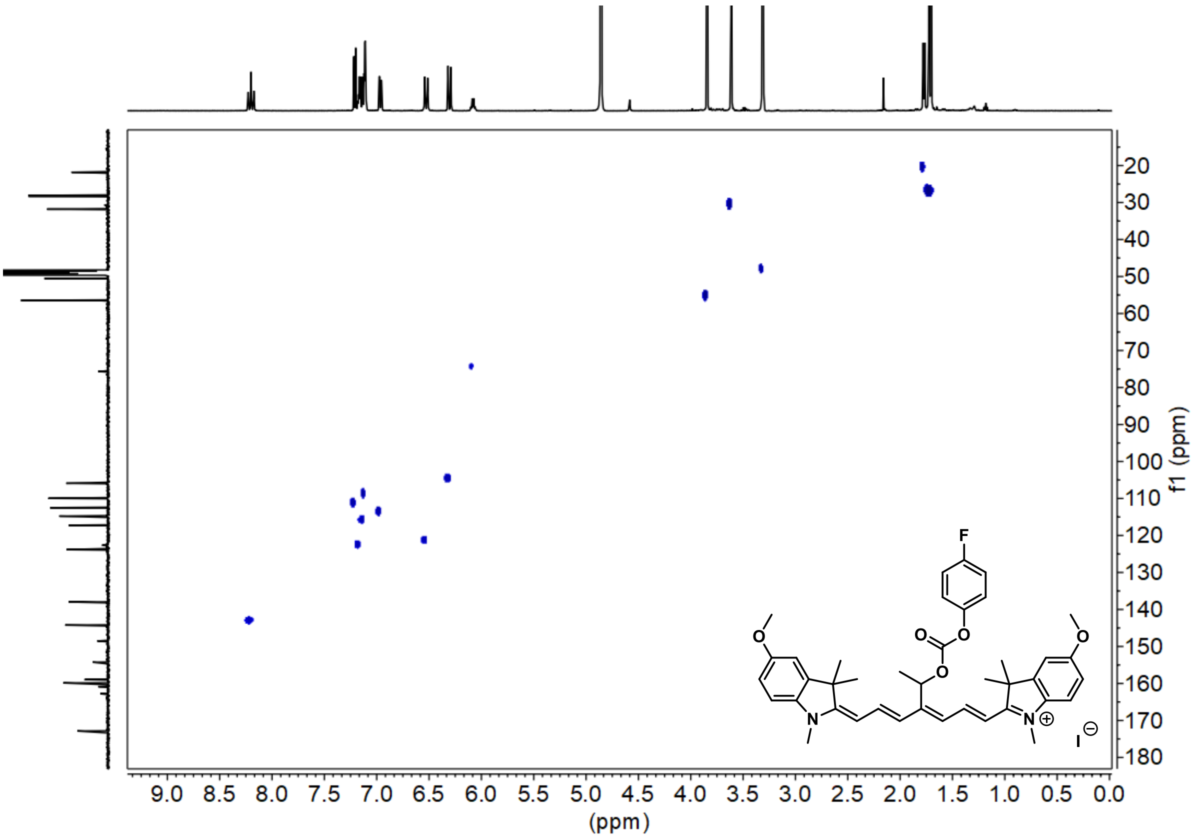

HSQC (500 MHz, 126 MHz, d_4-CD_3OD): **1a**

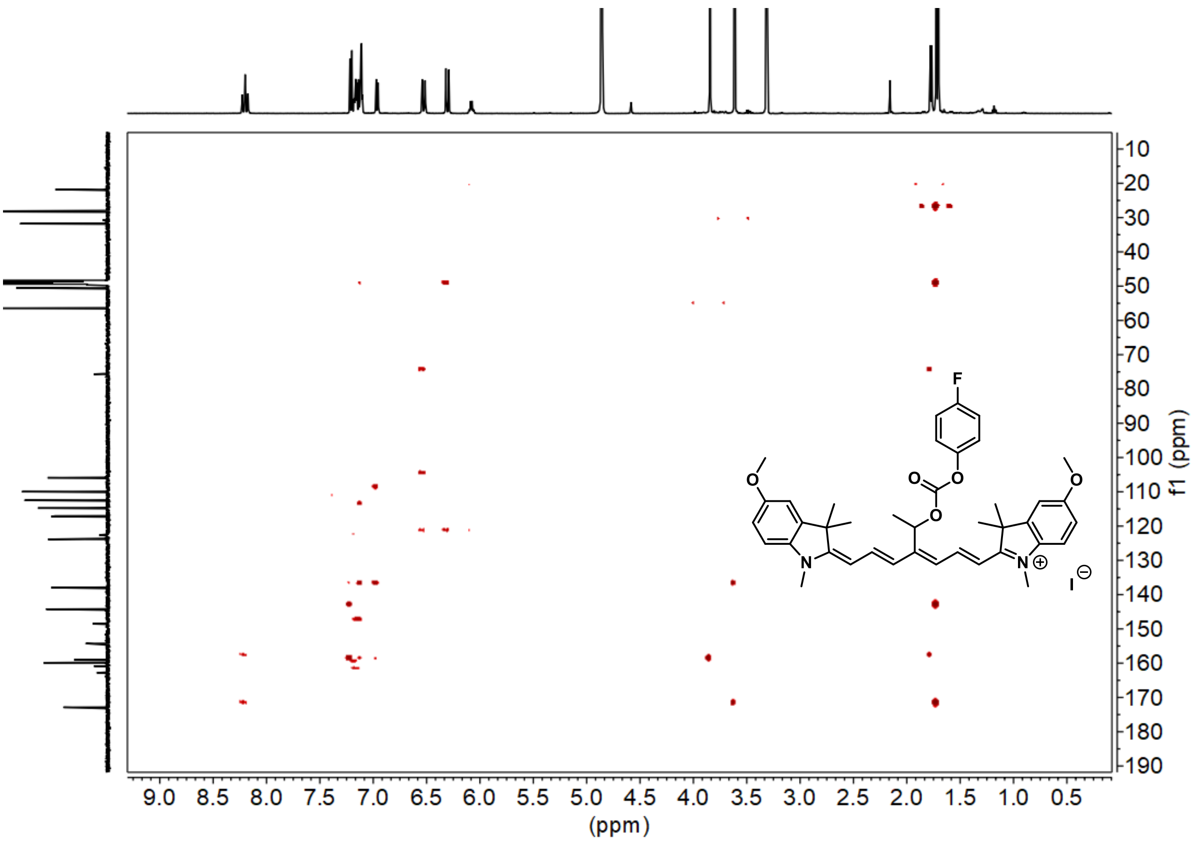

HMBC (500 MHz, 126 MHz, d_4-CD_3OD): **1a**

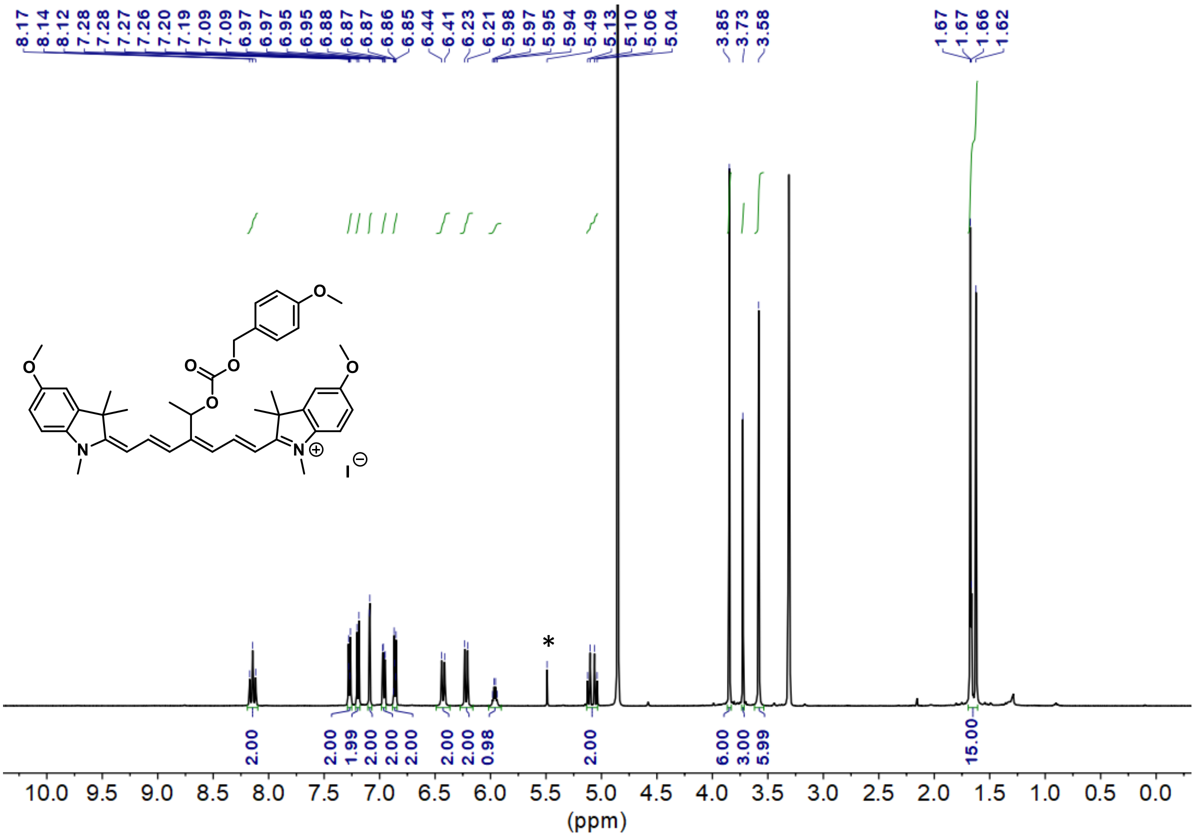

^{1}H NMR (500 MHz, d_4-CD_3OD): **1b**, *dichloromethane

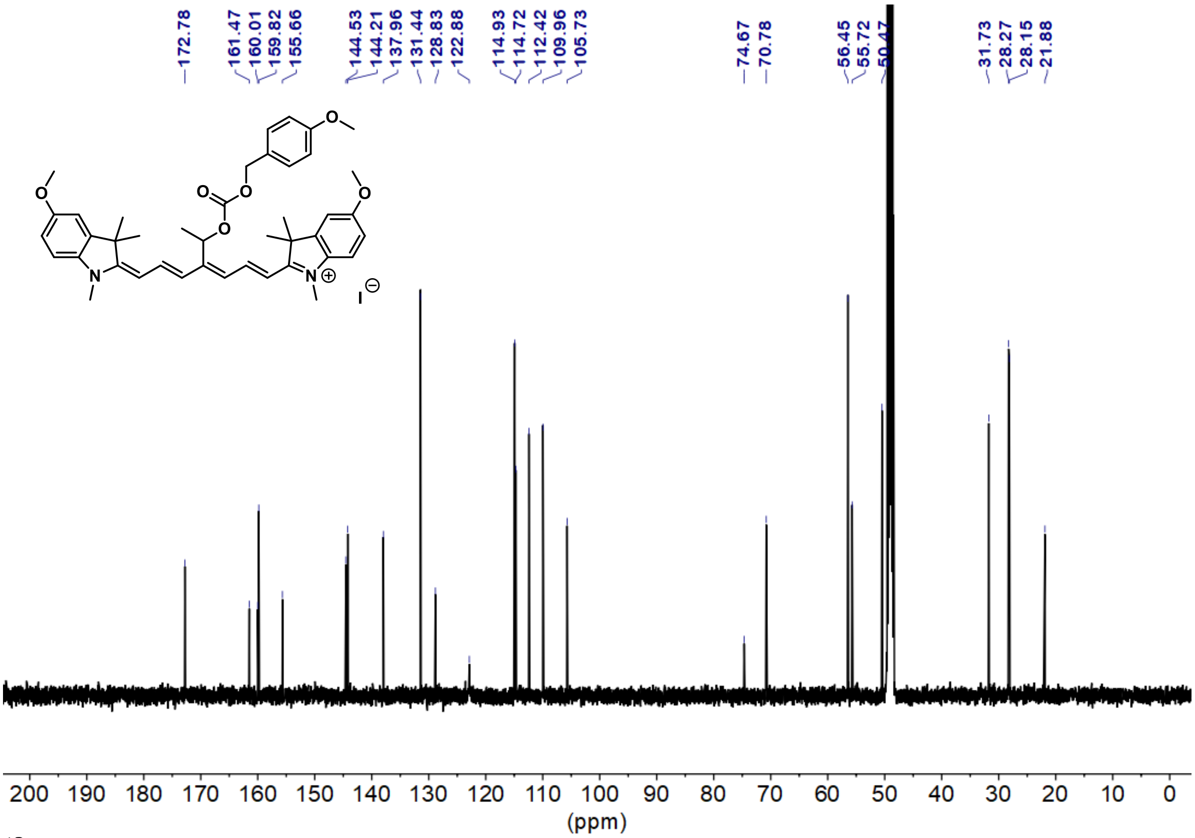

^{13}C NMR (126 MHz, d_4-CD_3OD): **1b**

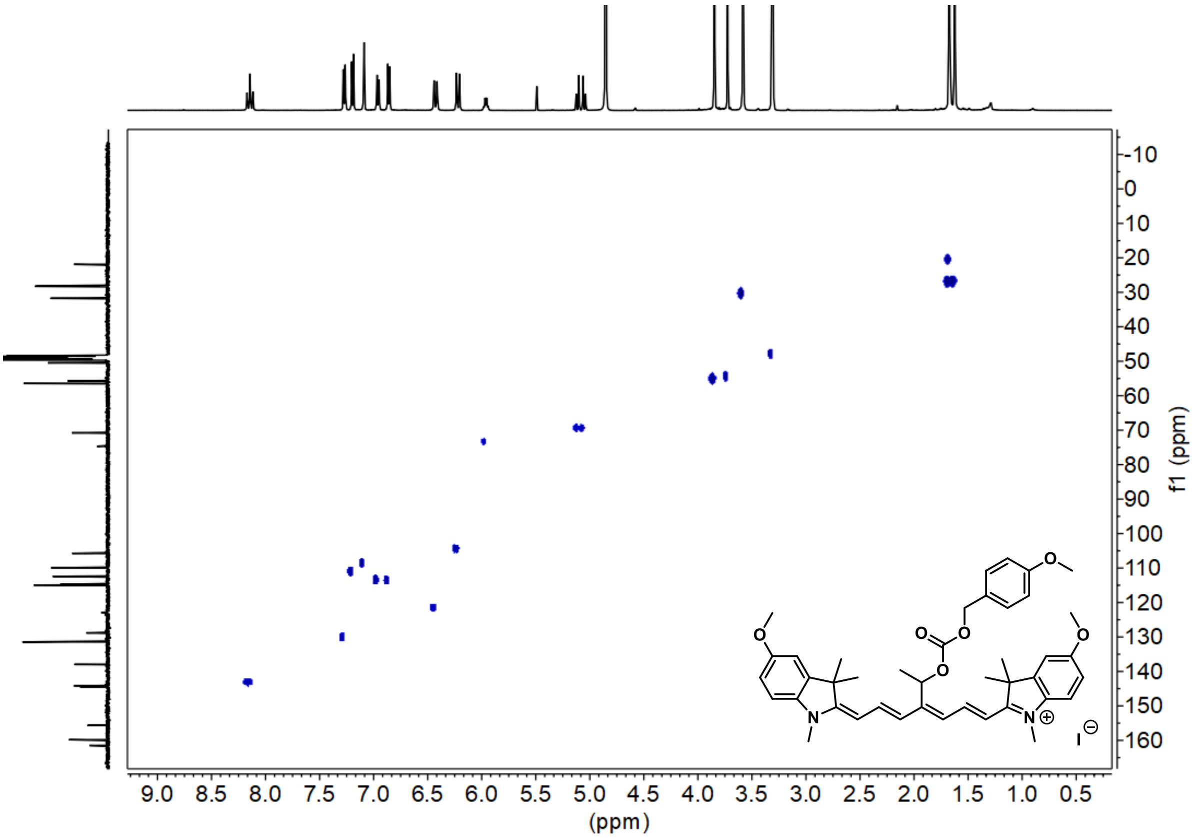

HSQC (500 MHz, 126 MHz, d_4-CD_3OD): **1b**

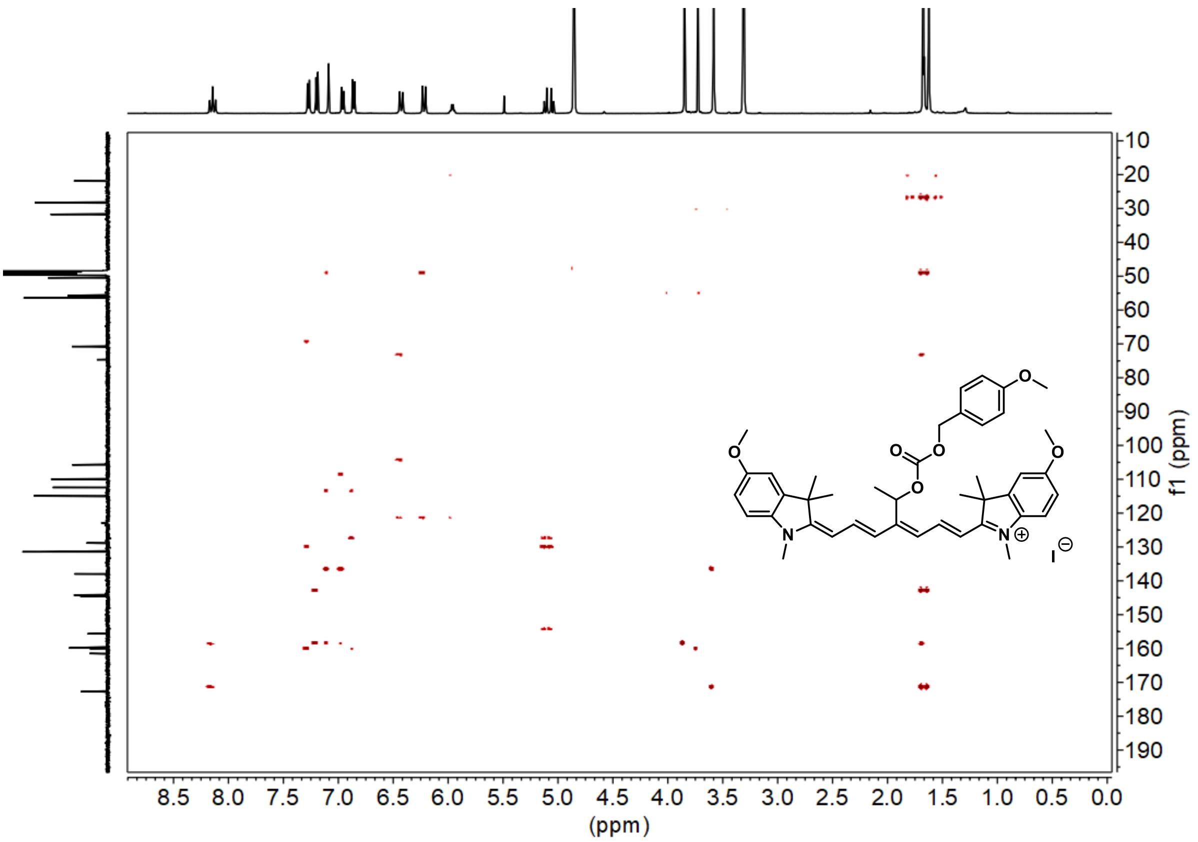

HMBC (500 MHz, 126 MHz, d_4-CD_3OD): **1b**

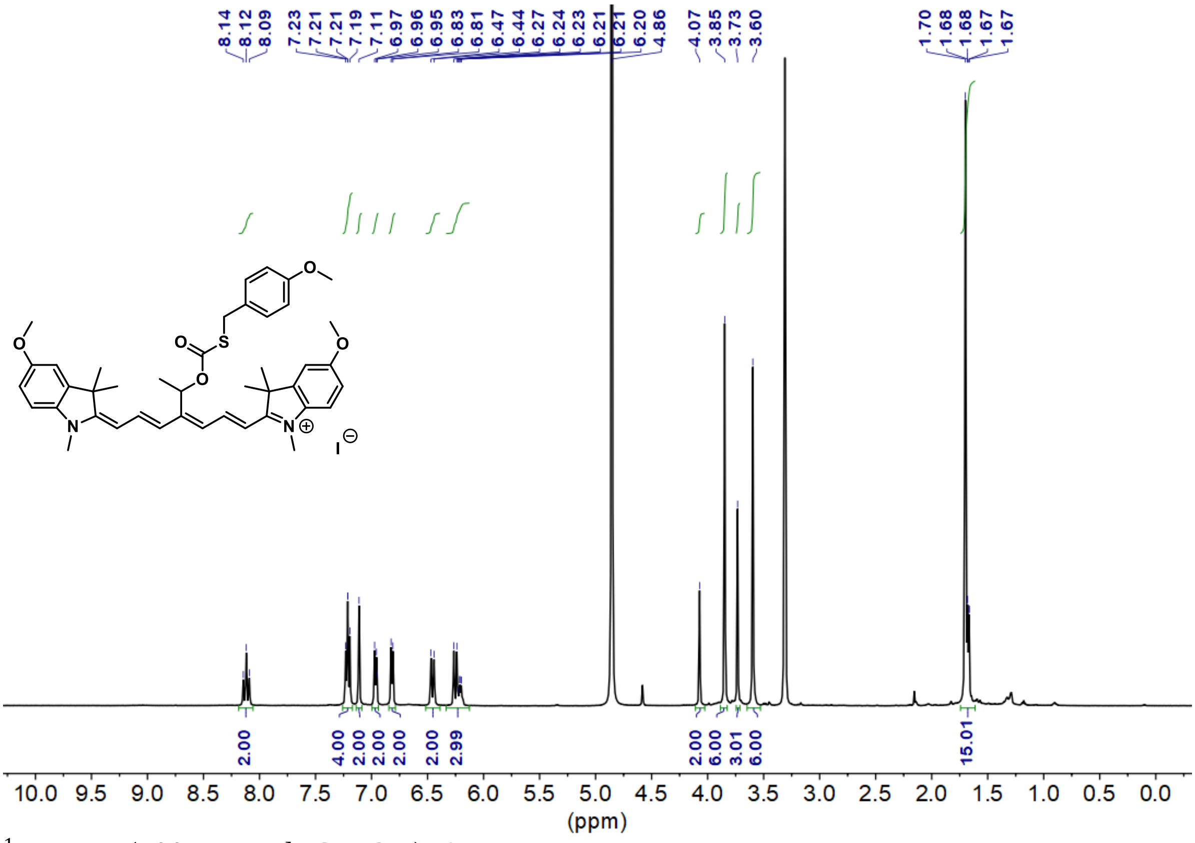

^1H NMR (500 MHz, d_4-CD_3OD): **1c**

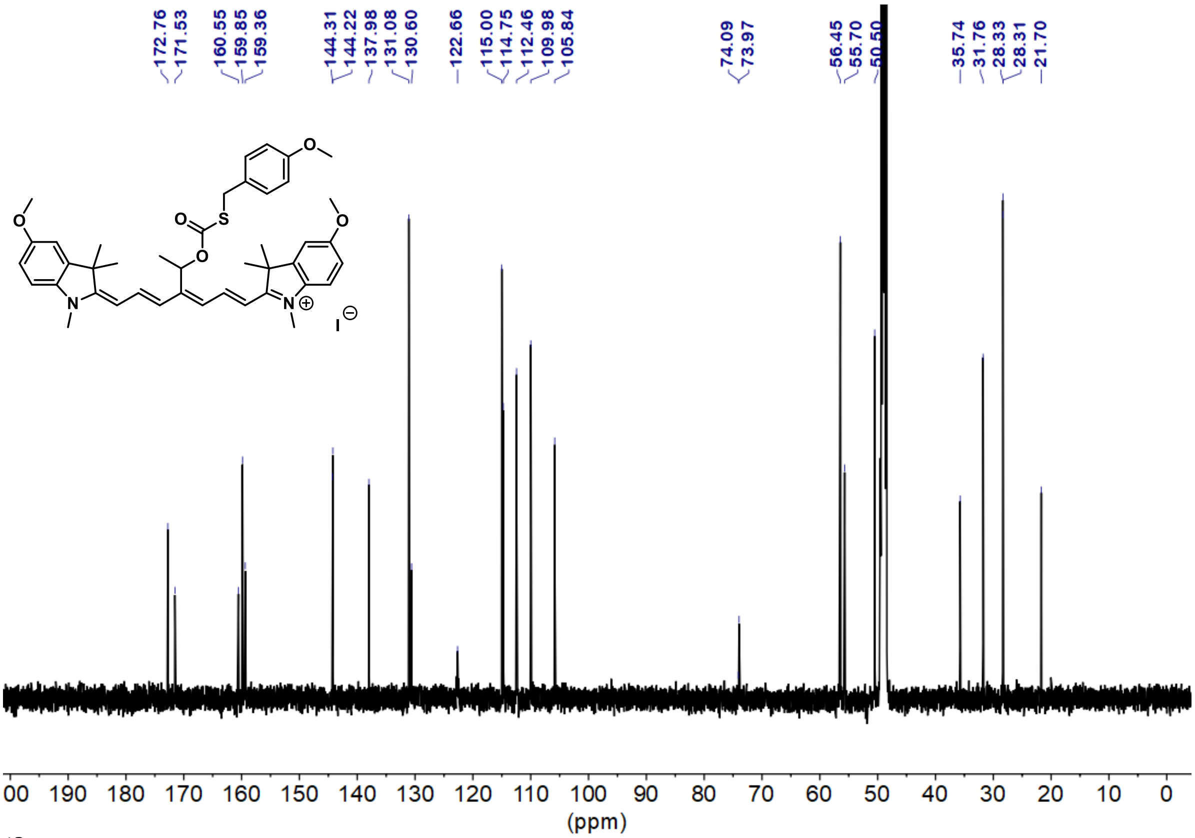

^{13}C NMR (126 MHz, d_4-CD_3OD): **1c**

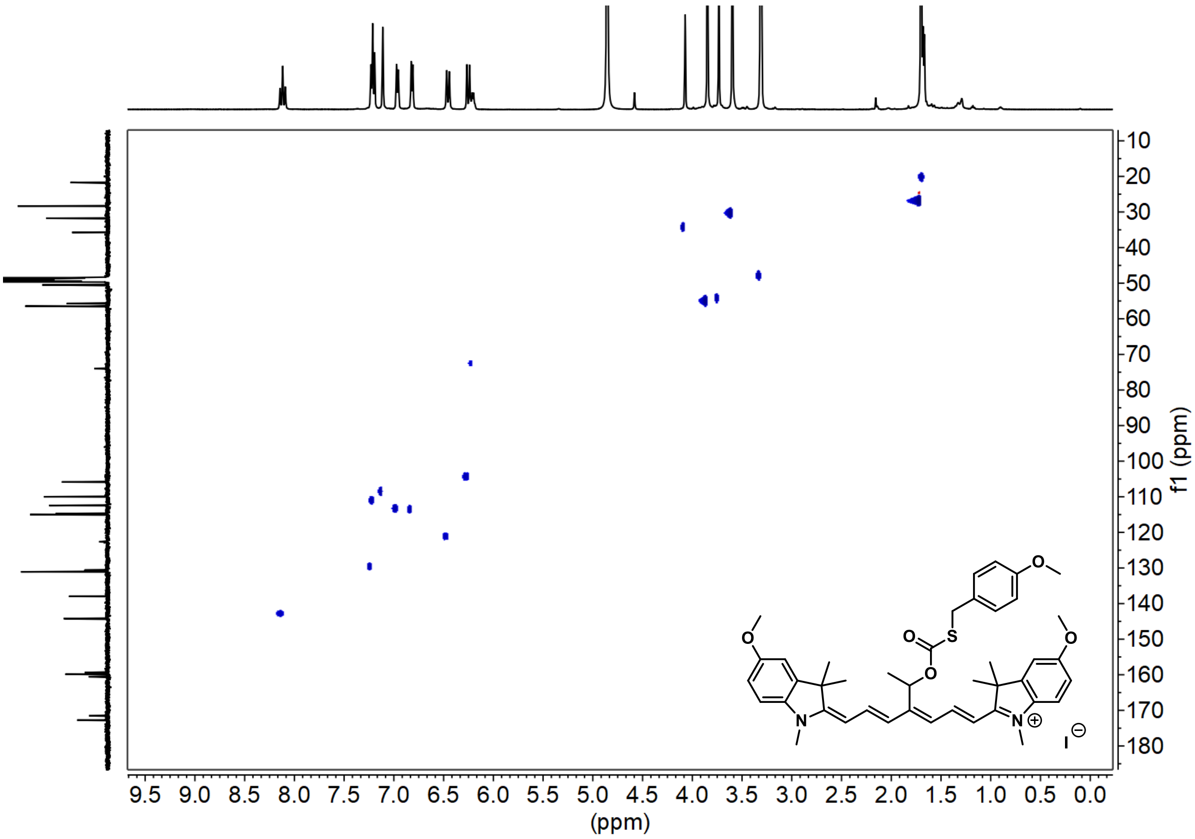

HSQC (500 MHz, 126 MHz, d_4-CD_3OD): **1c**

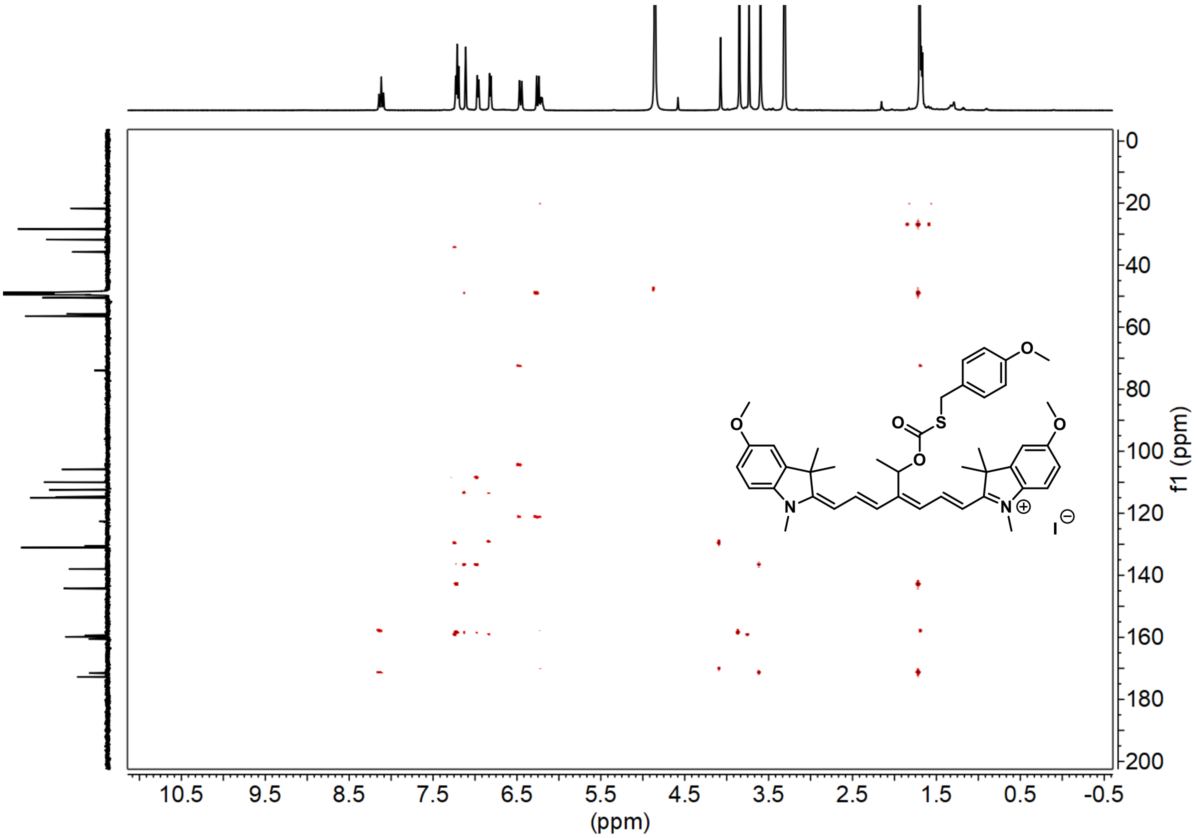

HMBC (500 MHz, 126 MHz, d_4-CD_3OD): **1c**

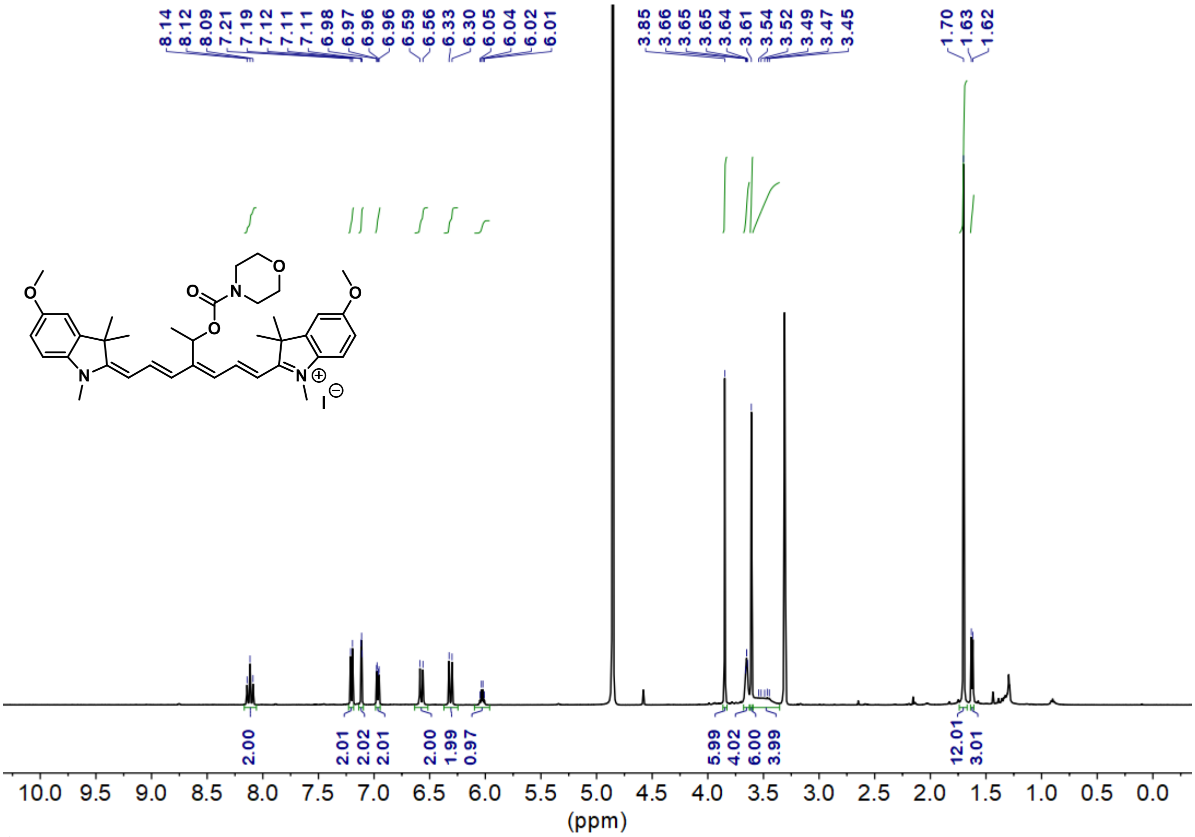

^{1}H NMR (500 MHz, d_4-CD_3OD): **1d**

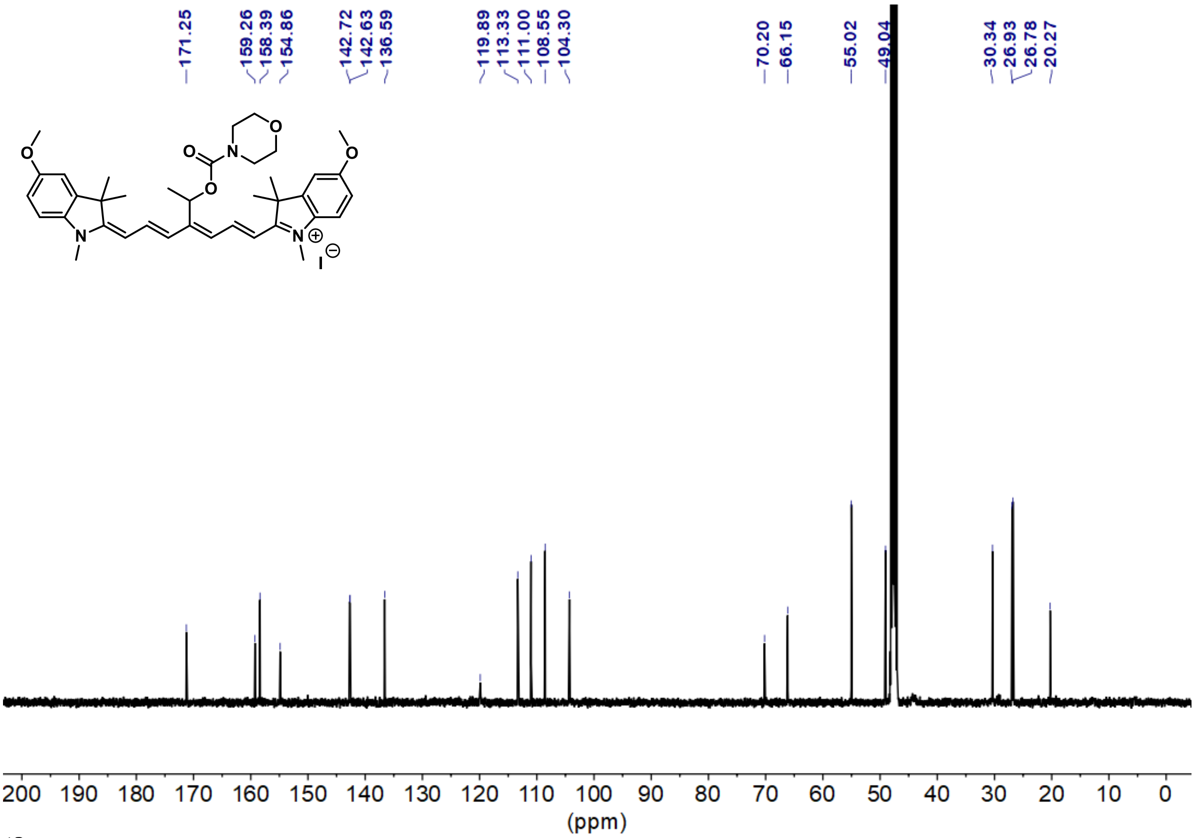

^{13}C NMR (126 MHz, d_4-CD_3OD): **1d**

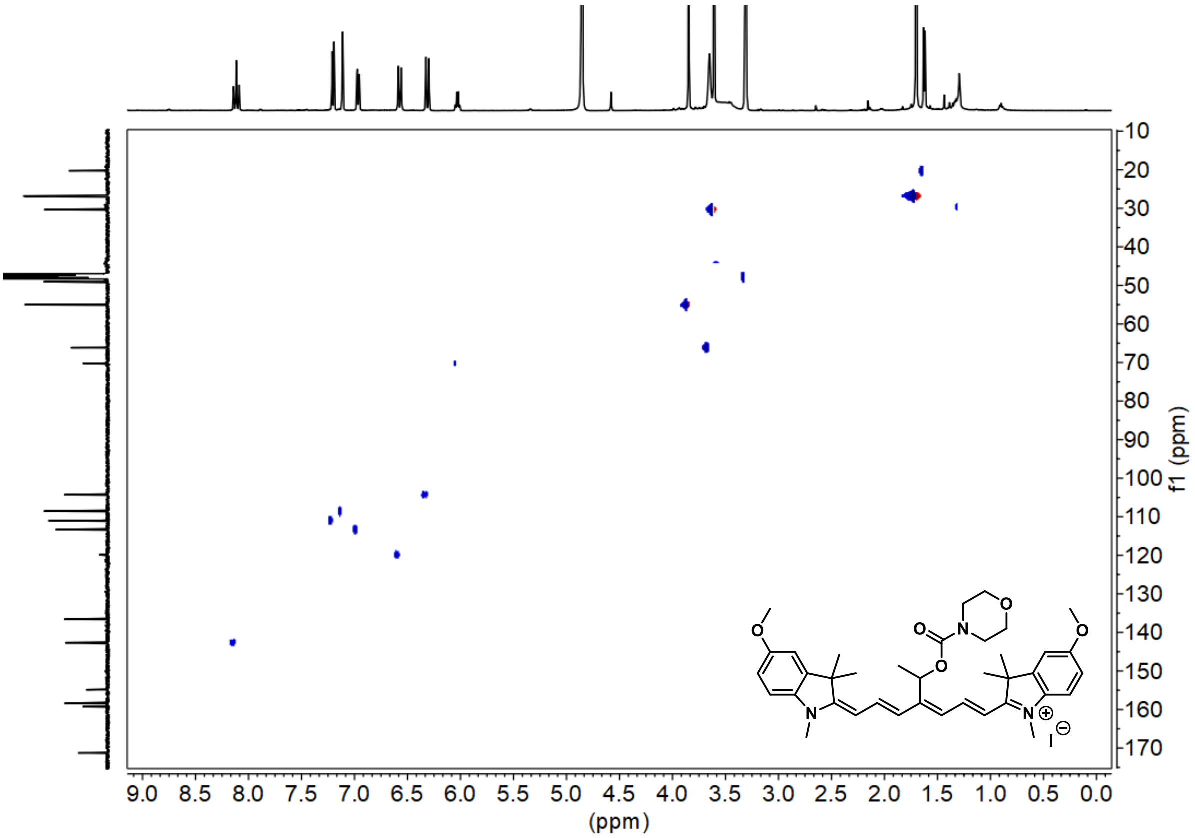

HSQC (500 MHz, 126 MHz, d_4-CD_3OD): **1d**

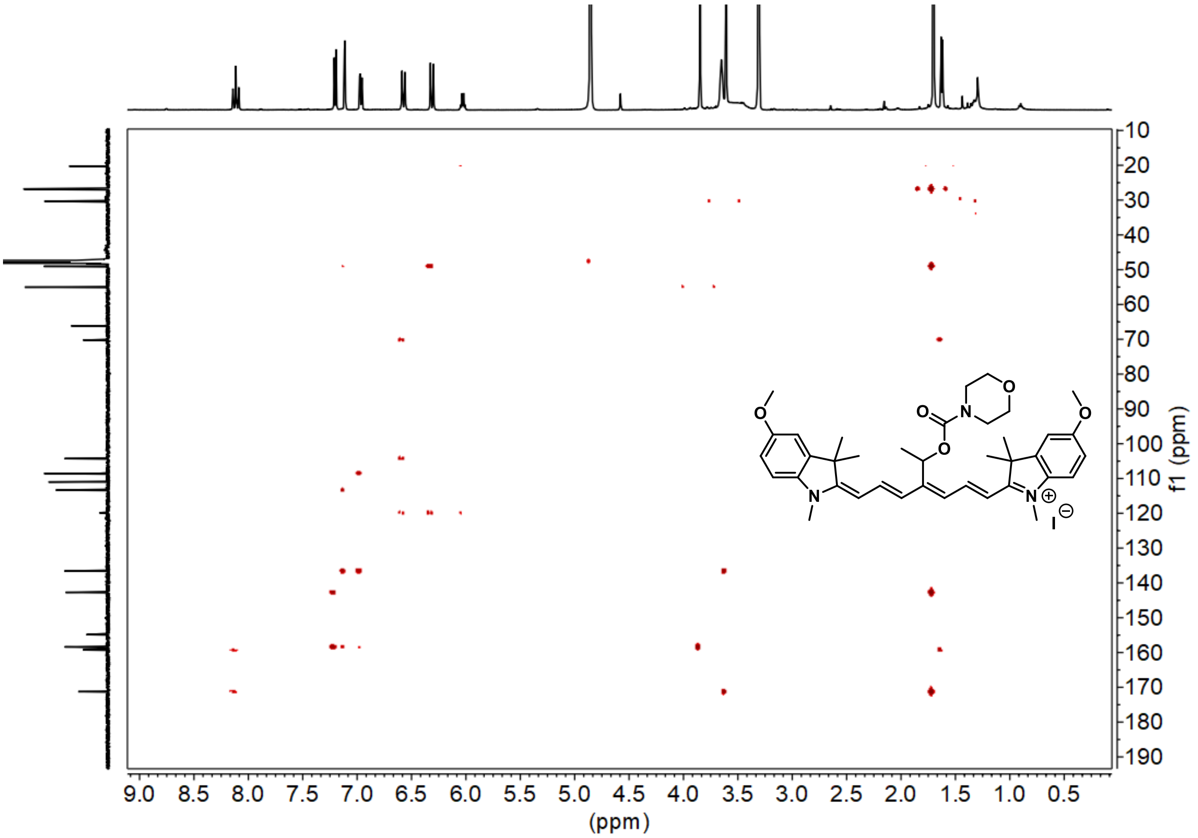

HMBC (500 MHz, 126 MHz, d_4-CD_3OD): **1d**

Note on Characterization of 1e–g by ^{1}H and ^{13}C NMR Spectroscopy

Characterization of 1e–g by ^{1}H and ^{13}C NMR spectroscopy was complicated by slow exchange room temperature and the existence of rotamers, presumably around the amidic bond. Heating to 80 °C was required to facilitate fast exchange and coalescence into a single spectrum – we include spectra recorded at both 25 °C and 80 °C. However, the photocages were not completely stable at 80 °C in d_6-DMSO for the duration required to record ^{13}C NMR spectra. Therefore, ^{13}C NMR spectra were recorded at room temperature. The structures were additionally confirmed by 2D NMR experiments – recorded at 47°C which is the maximum operational temperature for our NMR instrument equipped with a cryo-probe. These issues were further exacarbated in 1g which was prepared as a mixture of two diasteromers which are clearly visible in ^{1}H NMR spectra even at 80 °C, after the rotamers coalesce.

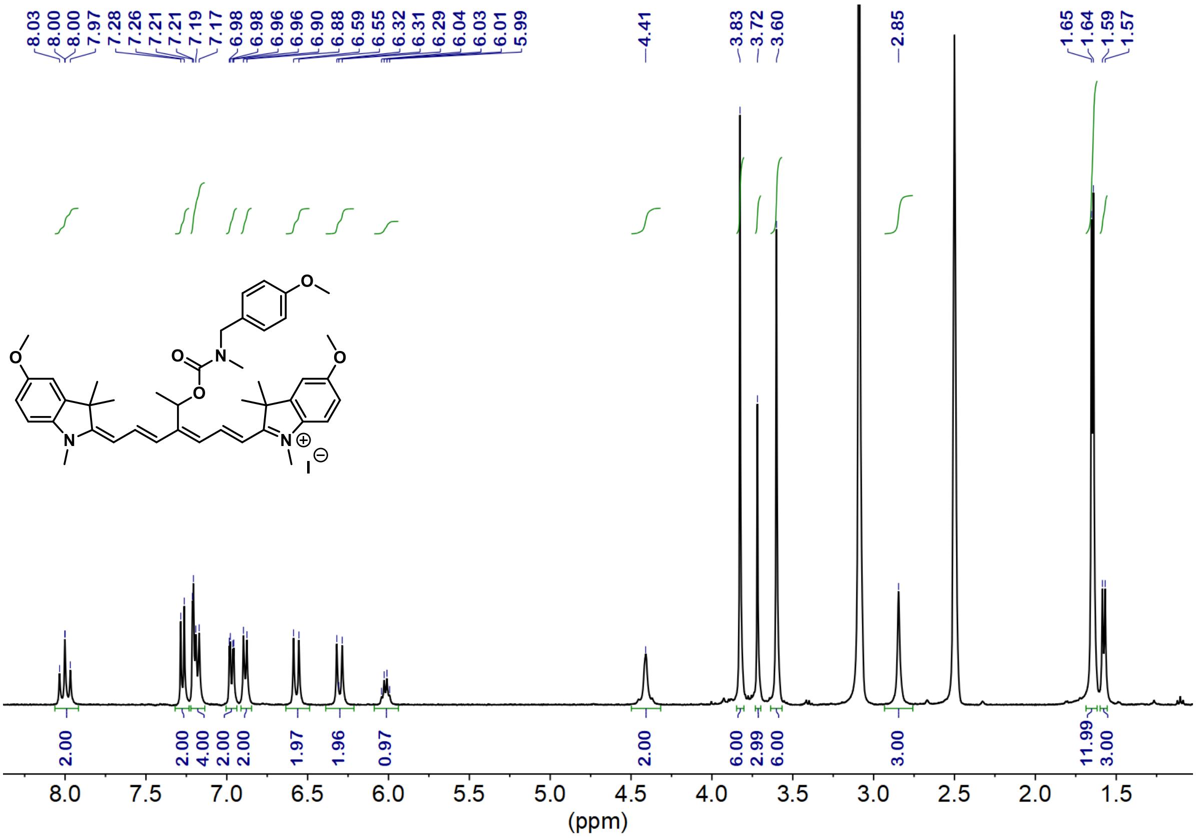

^{1}H NMR (400 MHz, d_6-DMSO, 80 °C): **1e**

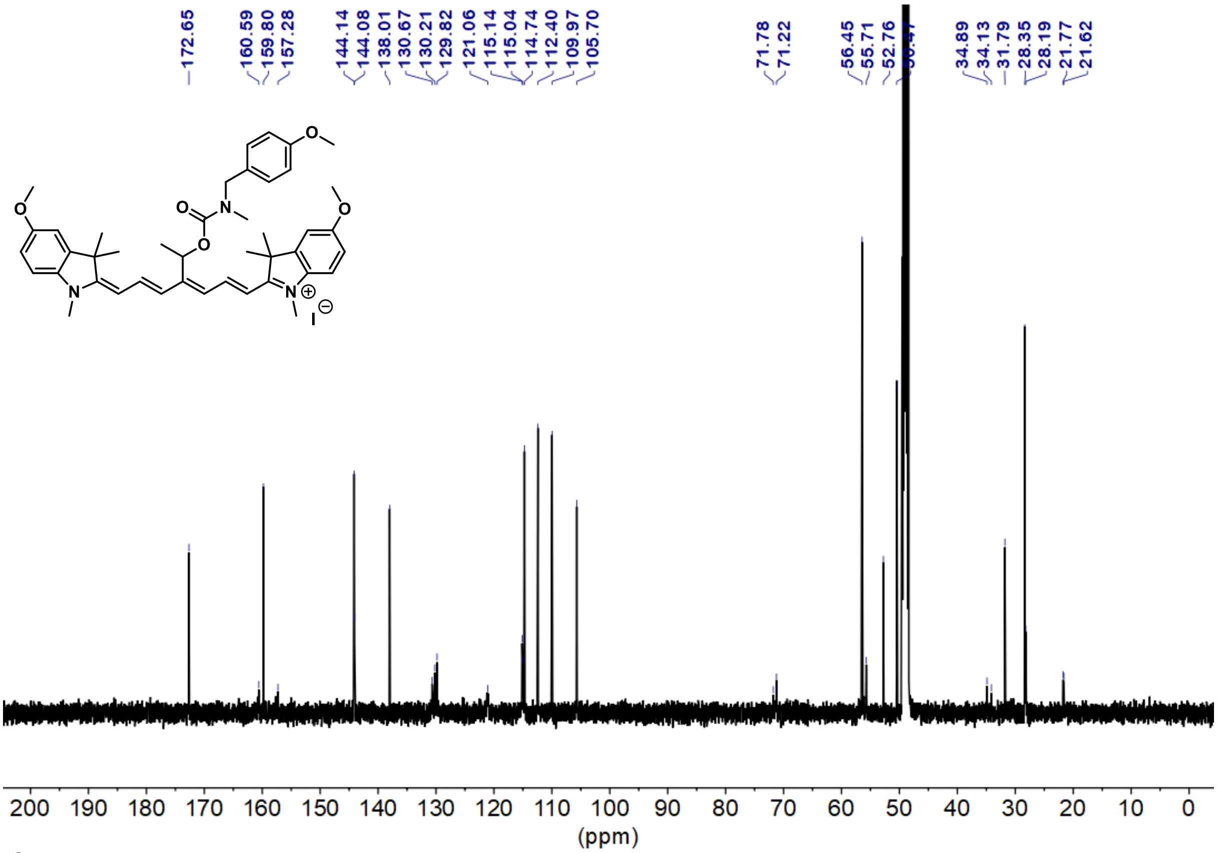

^{13}C NMR (126 MHz, d_4-CD_3OD, 25 °C): **1e**, mixture of rotamers

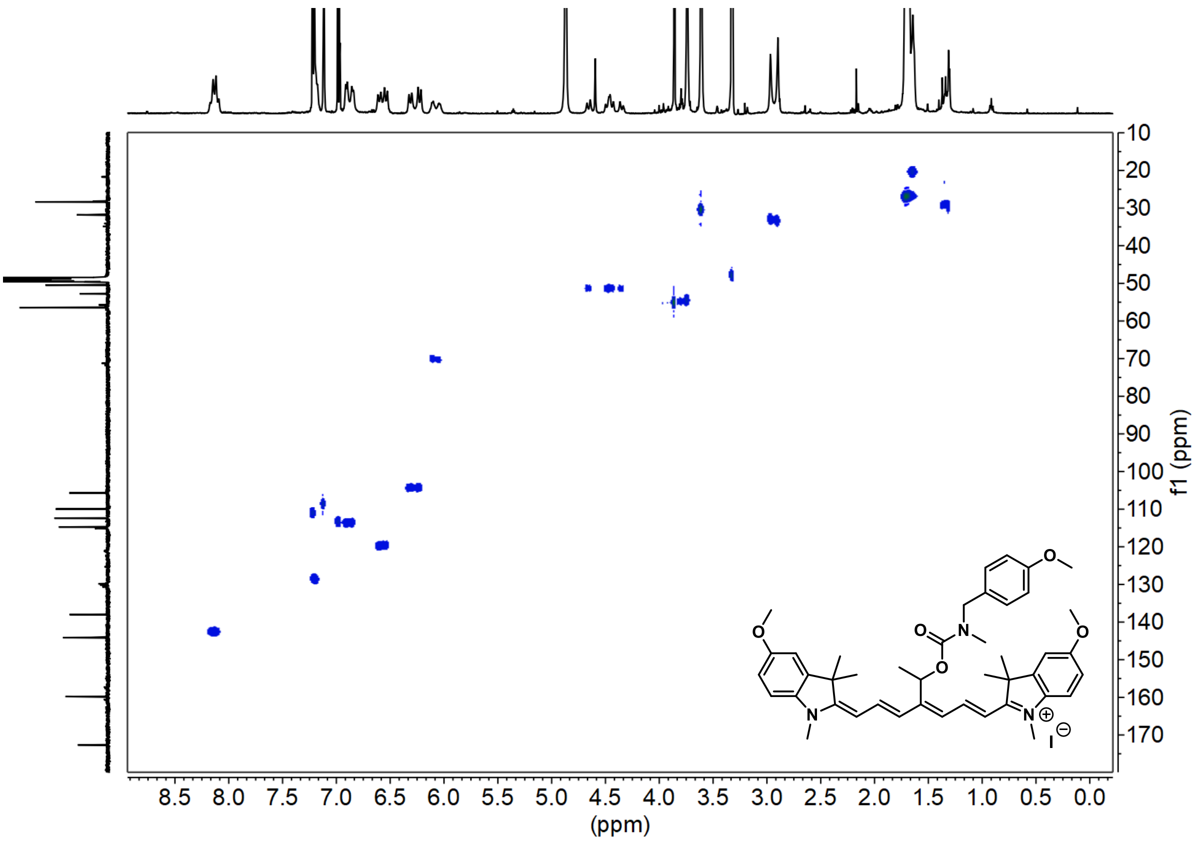

HSQC (500 MHz, 126 MHz, d_4-CD_3OD, 25 °C): **1e**, mixture of rotamers

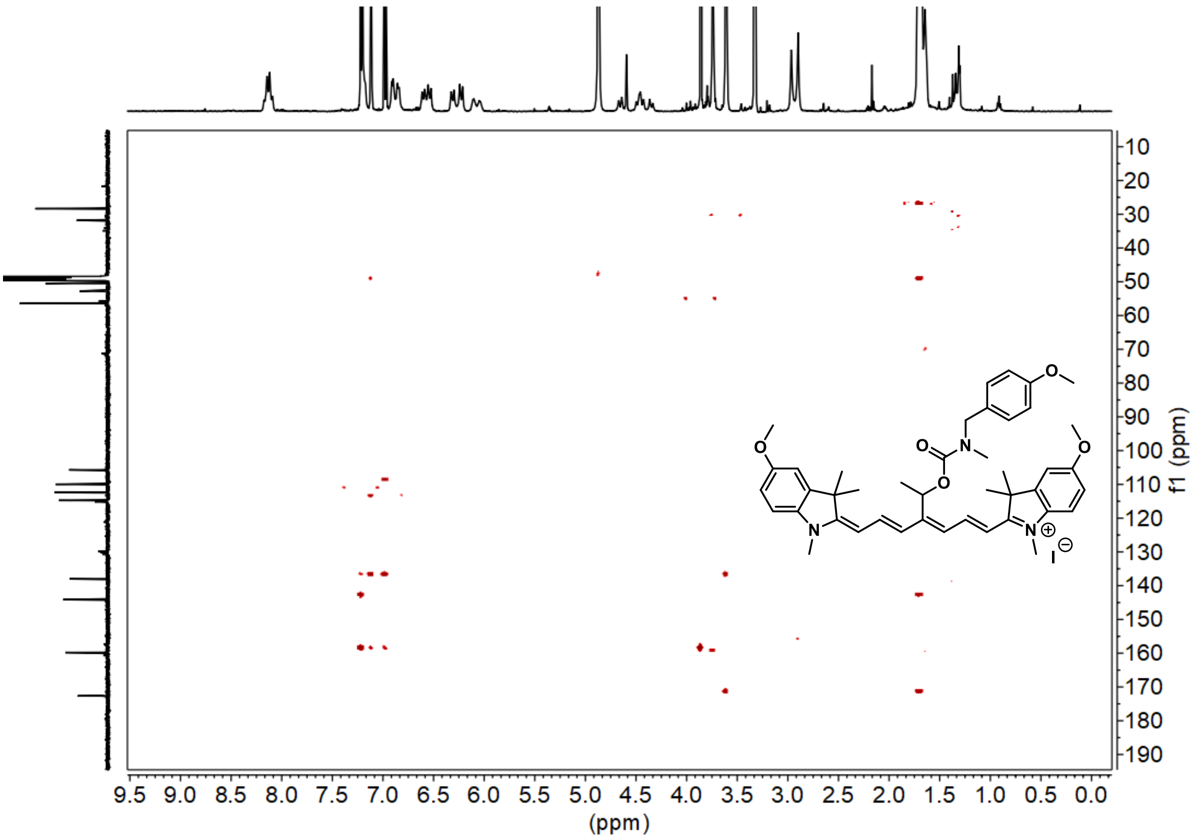

HMBC (500 MHz, 126 MHz, d_4-CD_3OD, 25 °C): **1e**, mixture of two rotamers

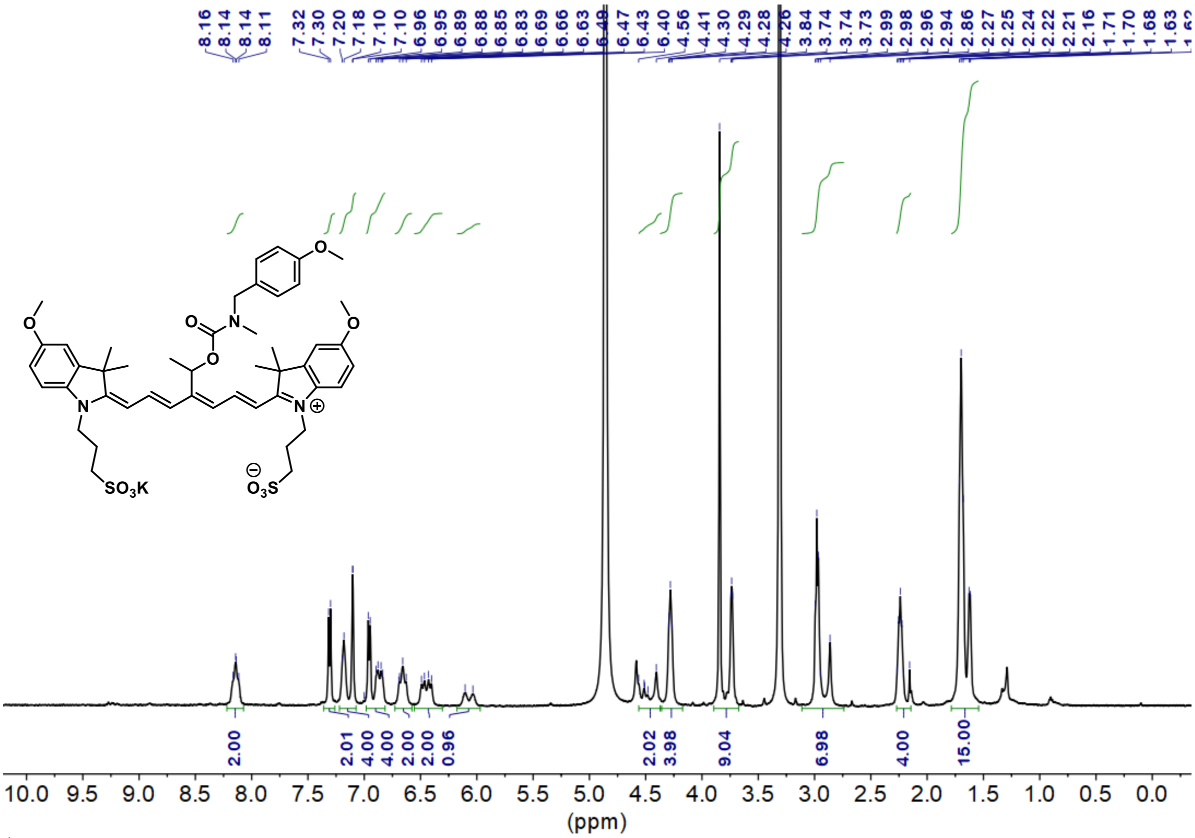

^{1}H NMR (500 MHz, d_4-CD_3OD, 25 °C): **1f**, mixture of rotamers

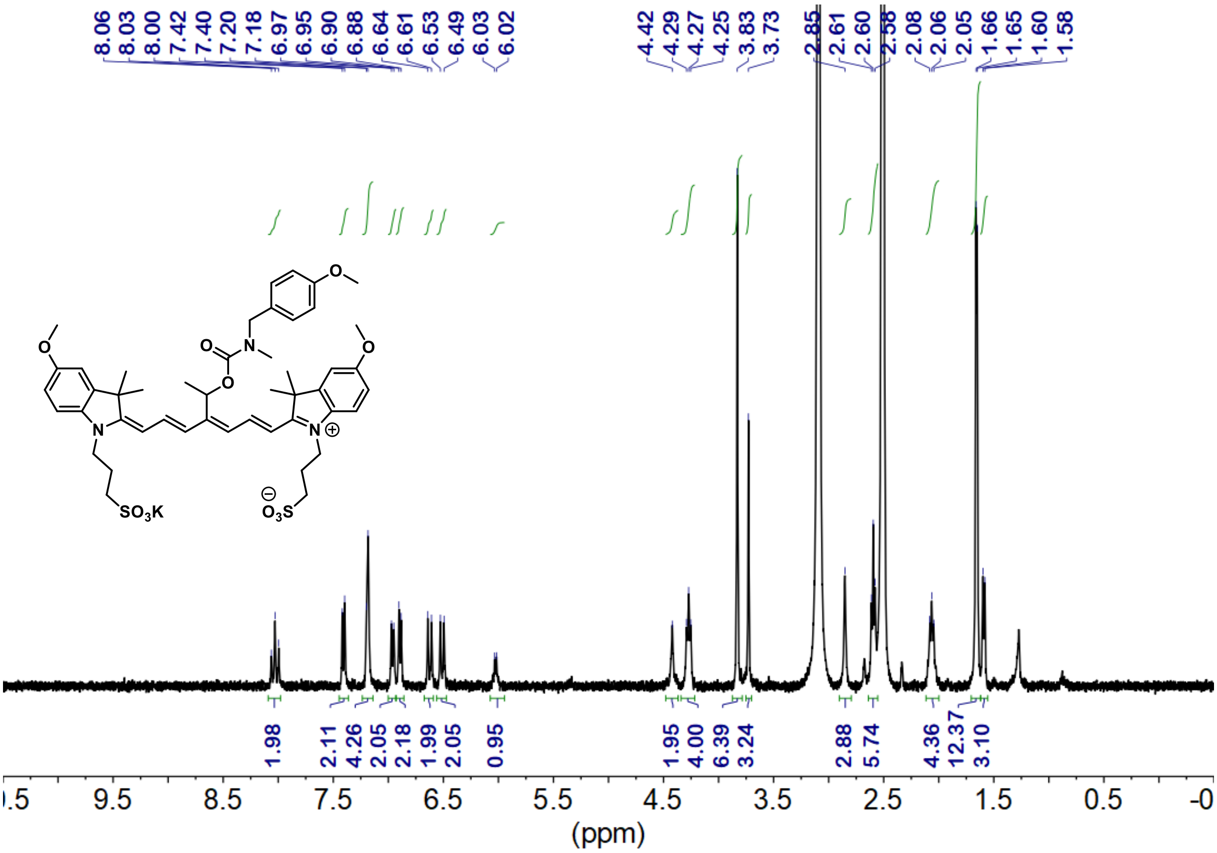

^{1}H NMR (500 MHz, d_6-DMSO, 80 °C): **1f**

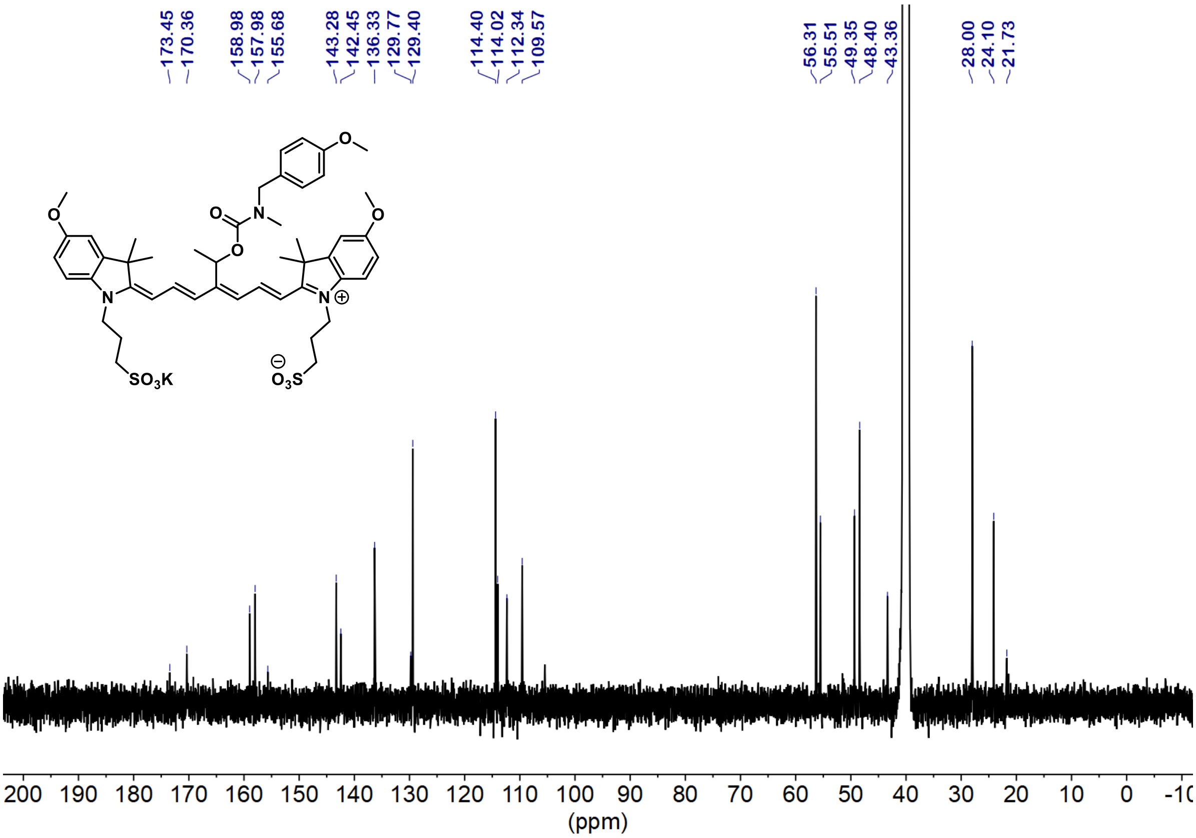

^{13}C NMR (126 MHz, d_6-DMSO, 25 °C): **1f**, mixture of rotamers

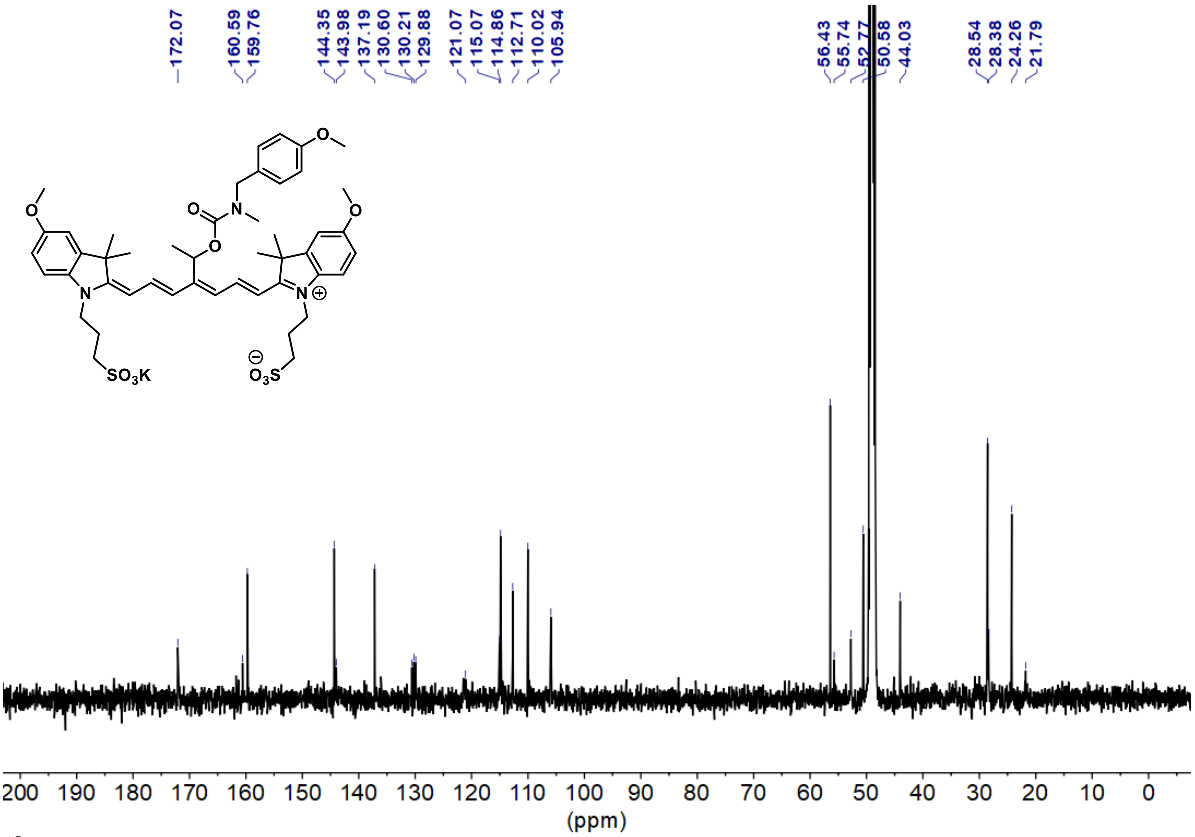

^{13}C NMR (126 MHz, d_4-CD_3OD, 47 °C): **1f**, mixture of rotamers

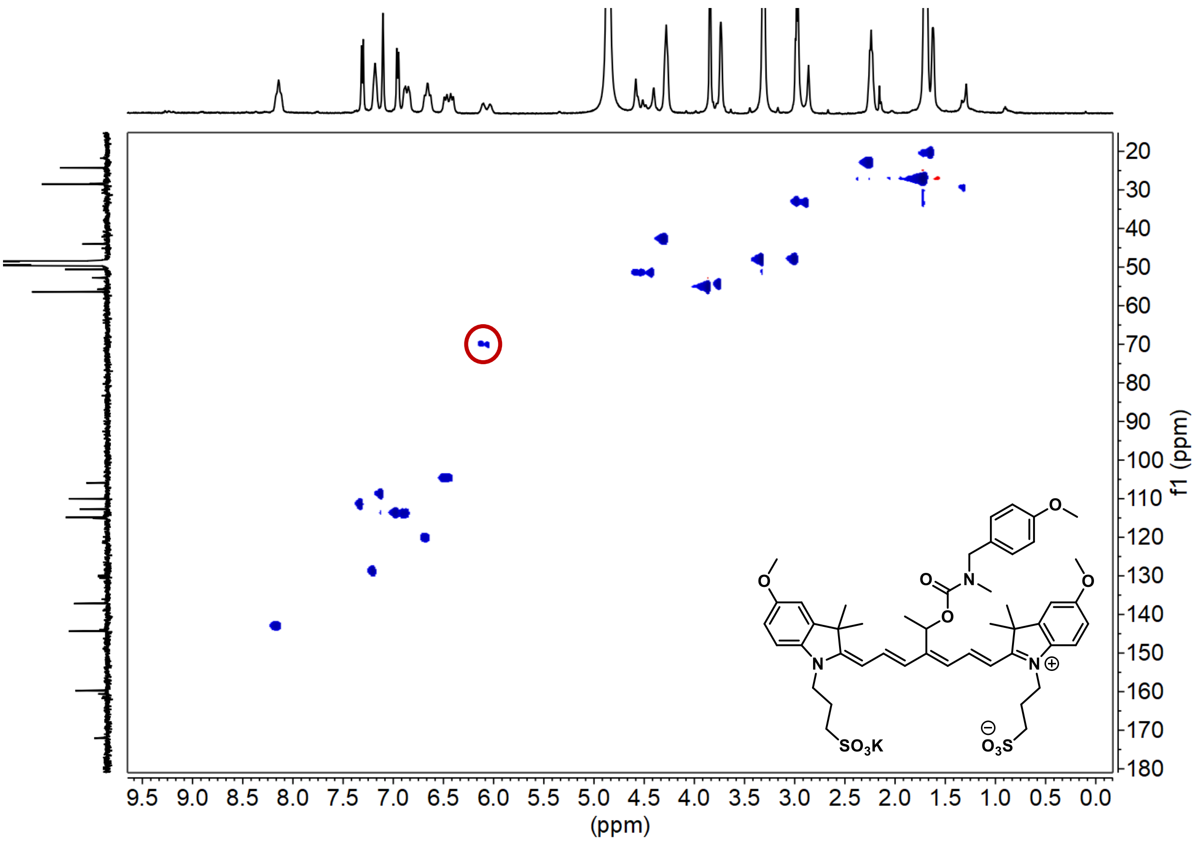

HSQC (500 MHz, 126 MHz, d_4-CD_3OD, 47 °C): **1f**, mixture of rotamers

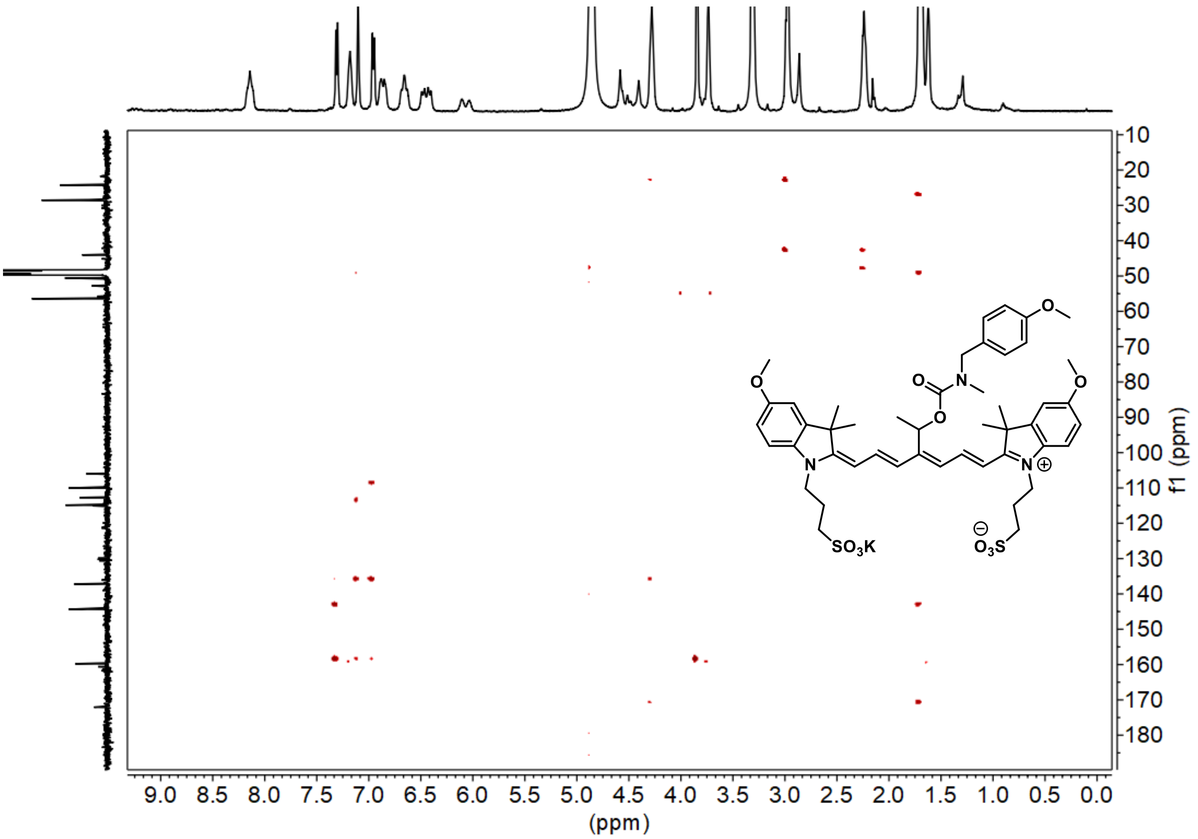

HMBC (500 MHz, 126 MHz, d_4-CD_3OD, 47 °C): **1f**, mixture of rotamers

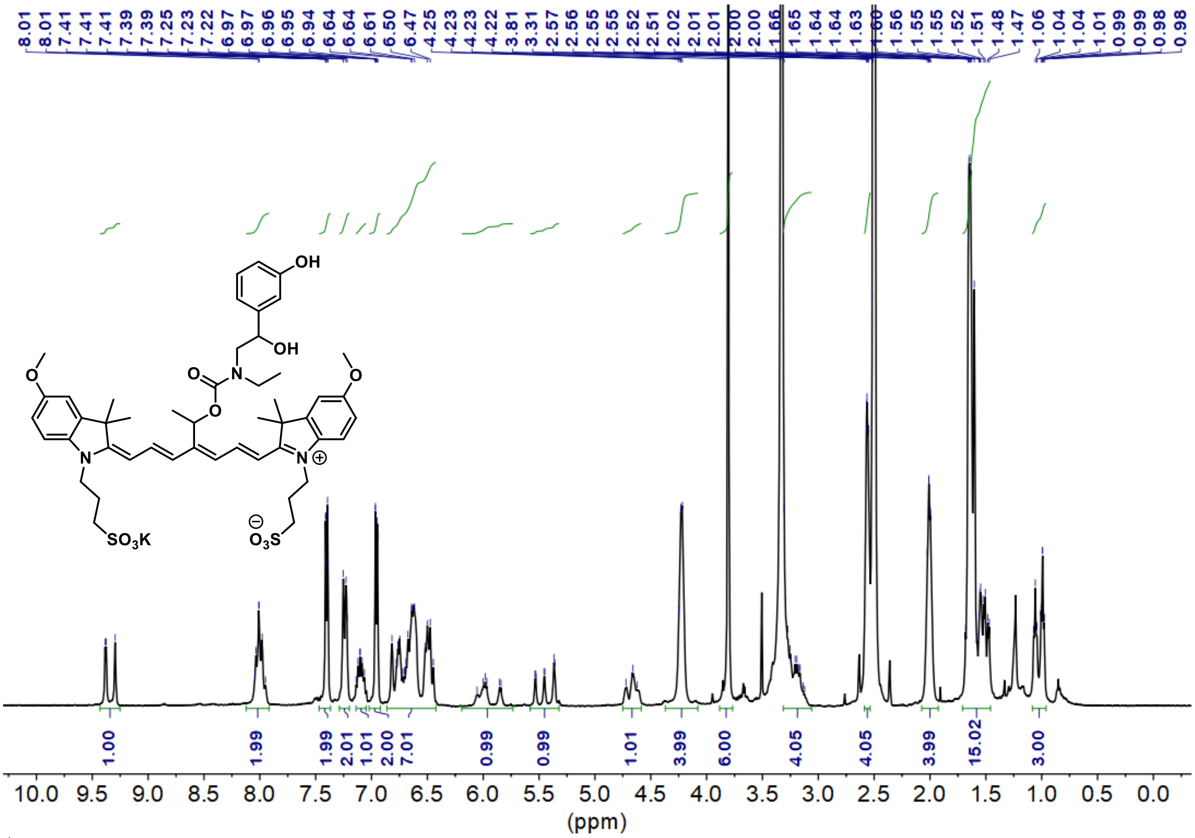

^{1}H NMR (500 MHz, d_6-DMSO, 25 °C): **1g**, mixture of diastereomers and rotamers

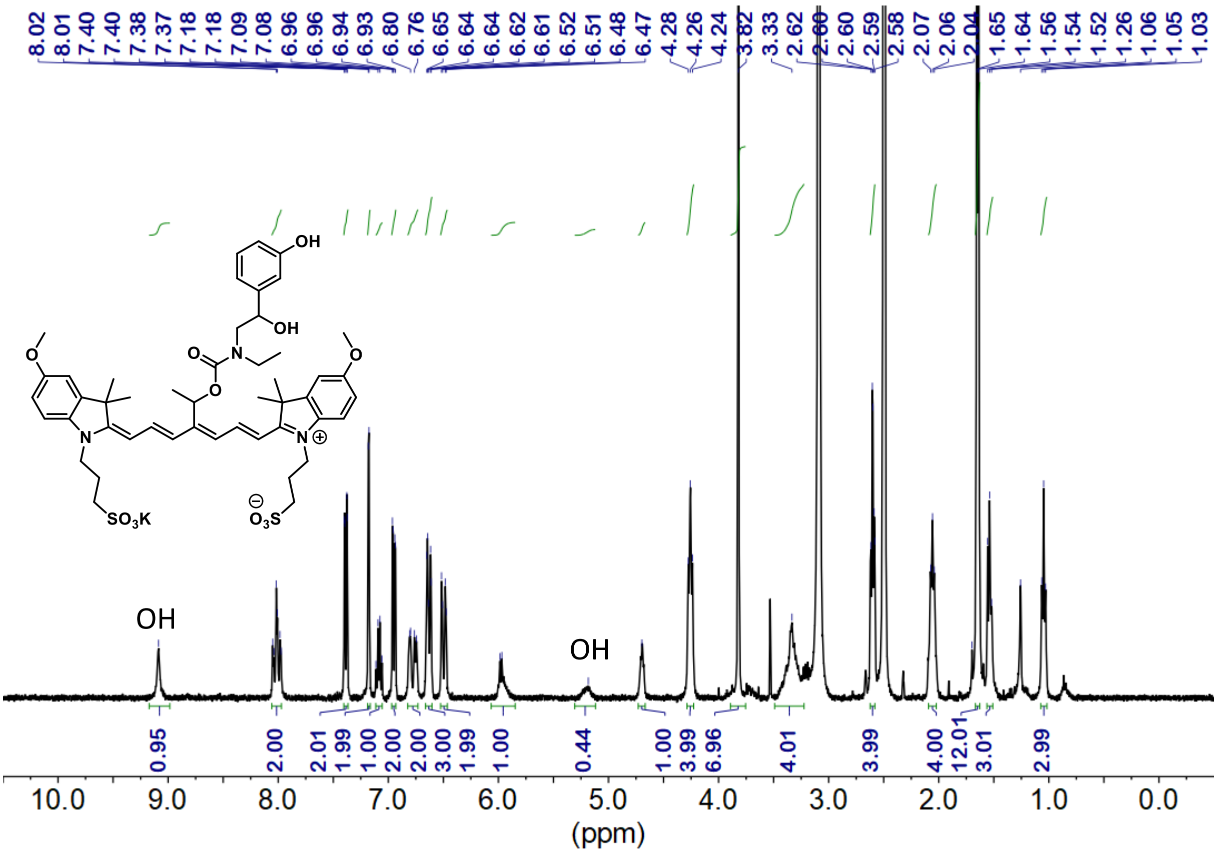

^{1}H NMR (500 MHz, d_6-DMSO, 80 °C): **1g**, mixture of diastereomers

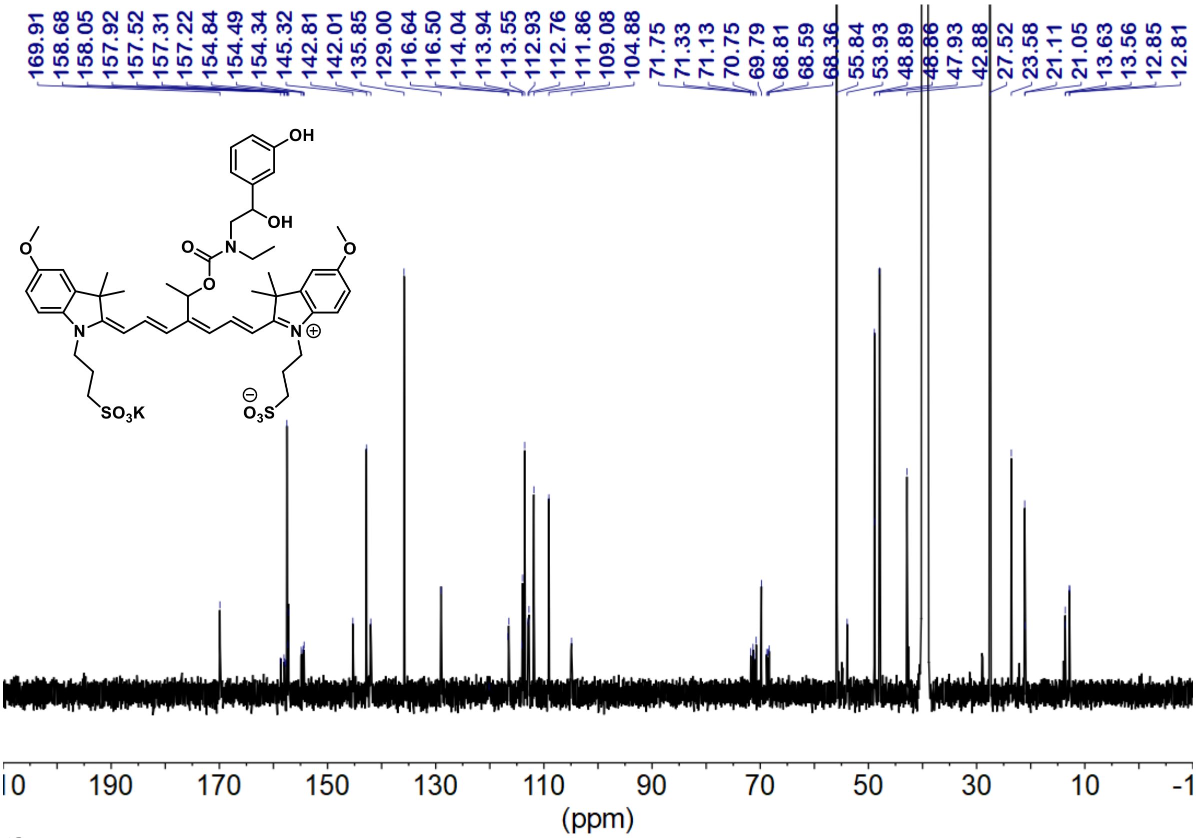

^{13}C NMR (126 MHz, d_6-DMSO, 25 °C): **1g**, mixture of diastereomers and rotamers

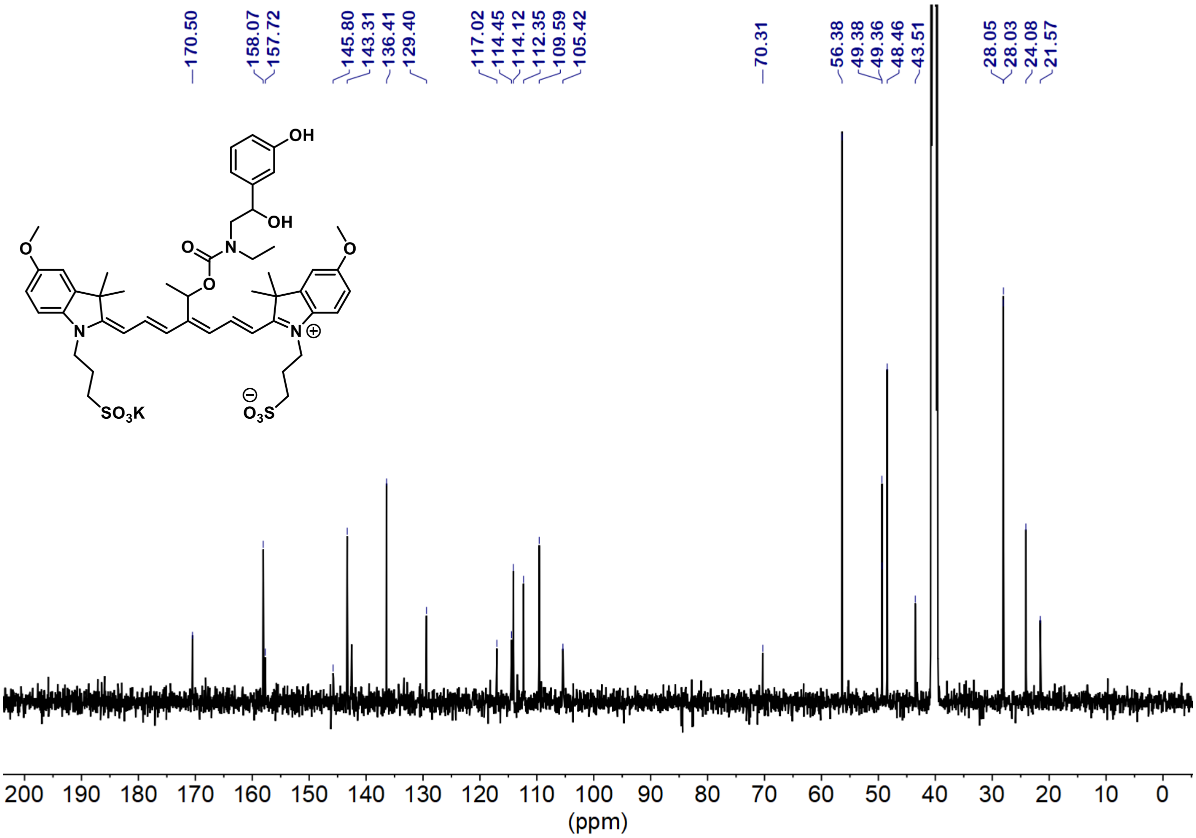

^{13}C NMR (126 MHz, d_6-DMSO, 47 °C): **1g**, mixture of diastereomers and rotamers

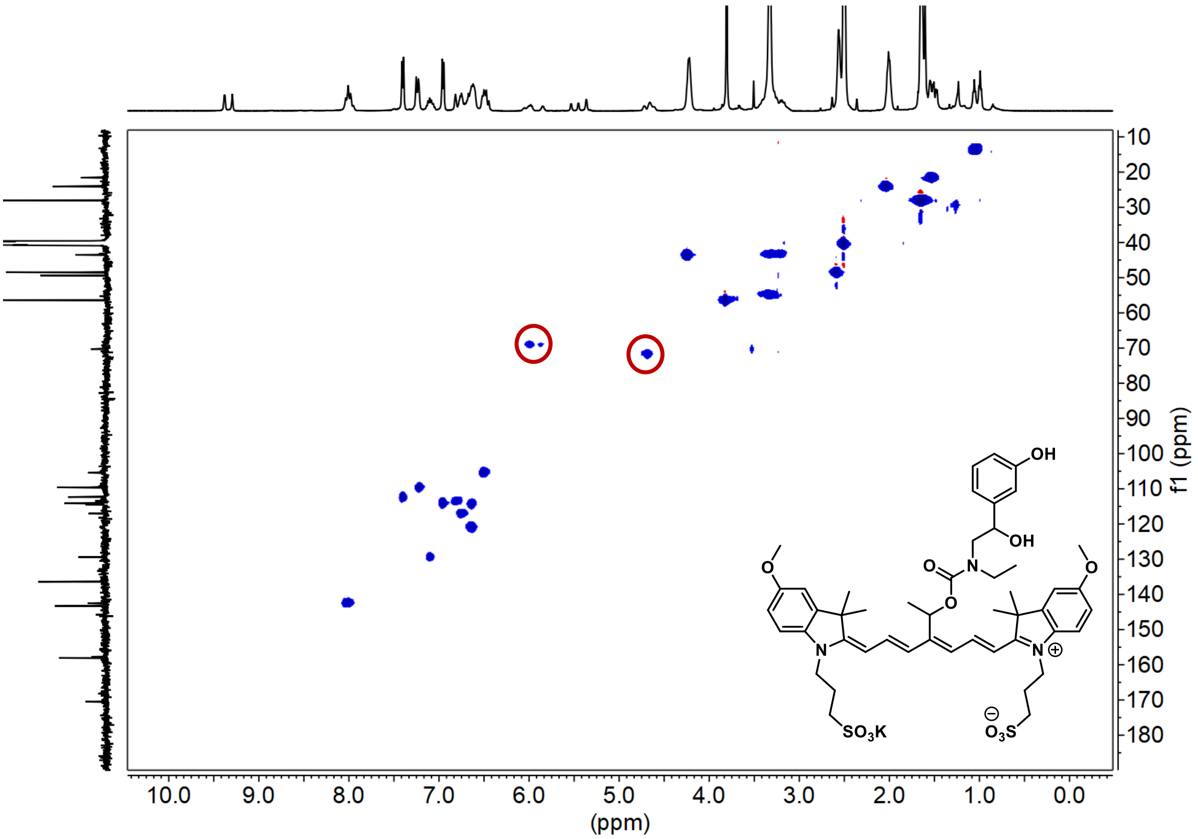

HSQC (500 MHz, 126 MHz, d_6-DMSO, 47 °C): **1g**, mixture of diastereomers and rotamers

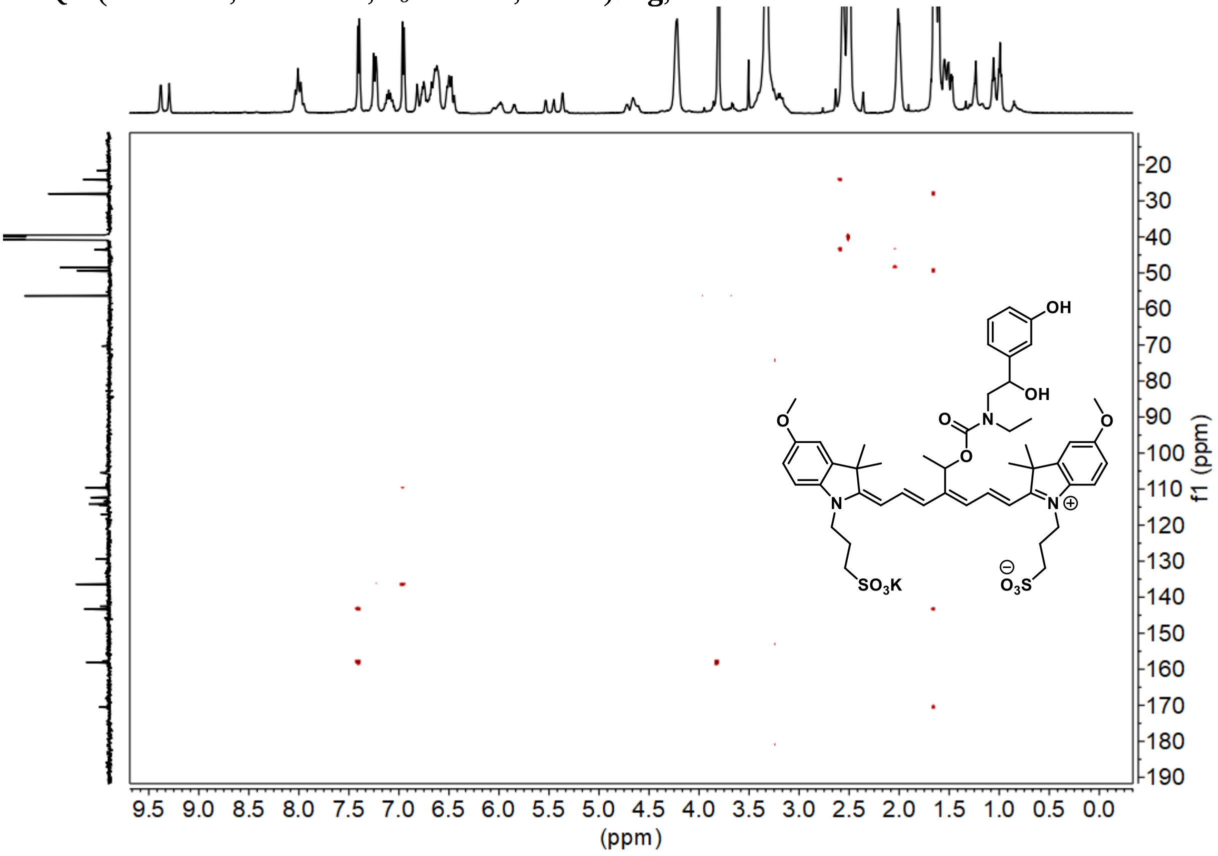

HMBC (500 MHz, 126 MHz, d_6-DMSO): **1g**, mixture of diastereomers and rotamers

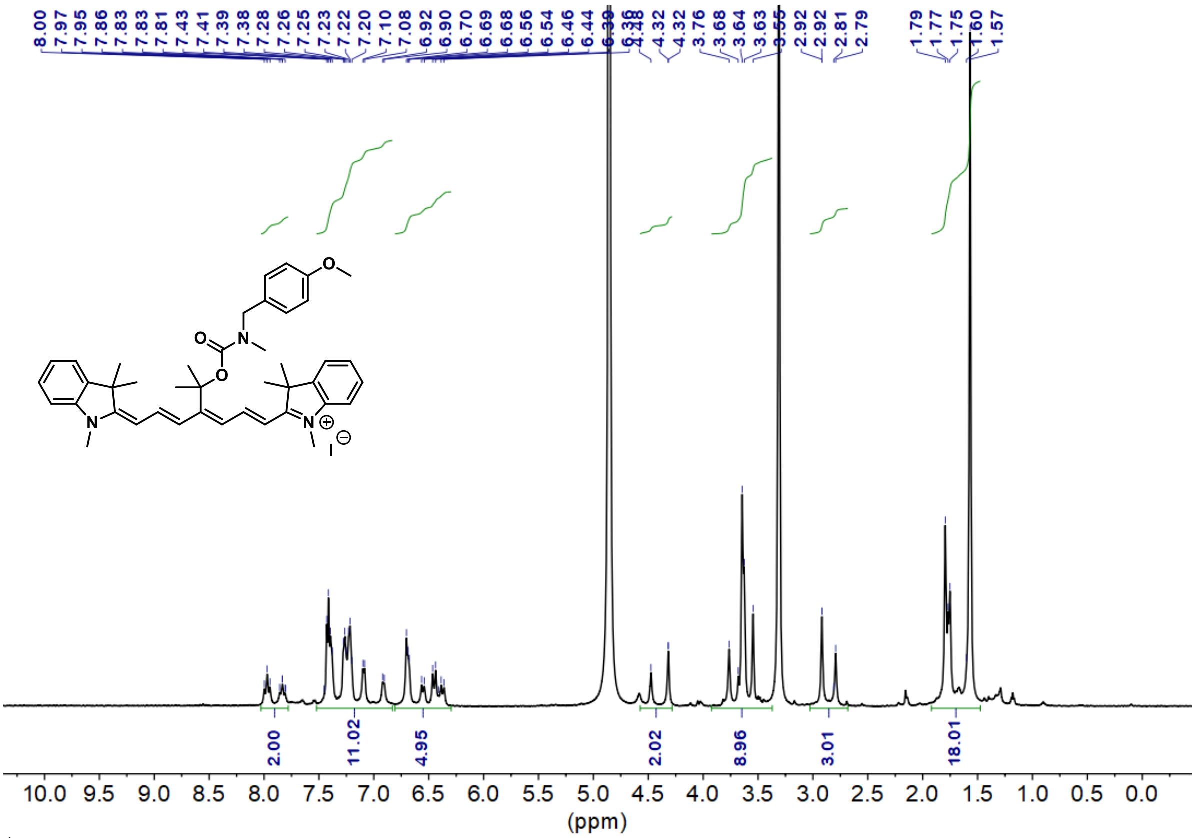

^{1}H NMR (500 MHz, d_4-CD_3OD, 25 °C): **1h**, mixture of rotamers

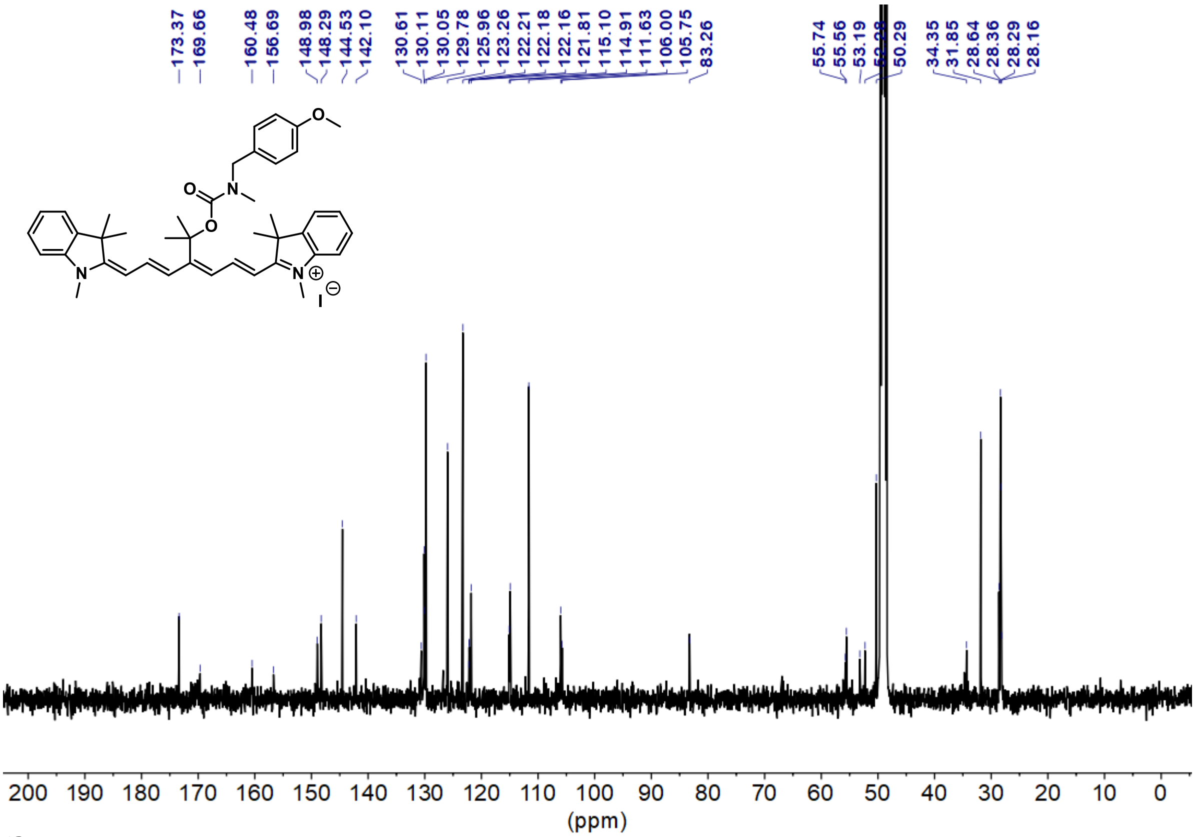

^{13}C NMR (126 MHz, d_4-CD_3OD, 25 °C): **1h**, mixture of rotamers

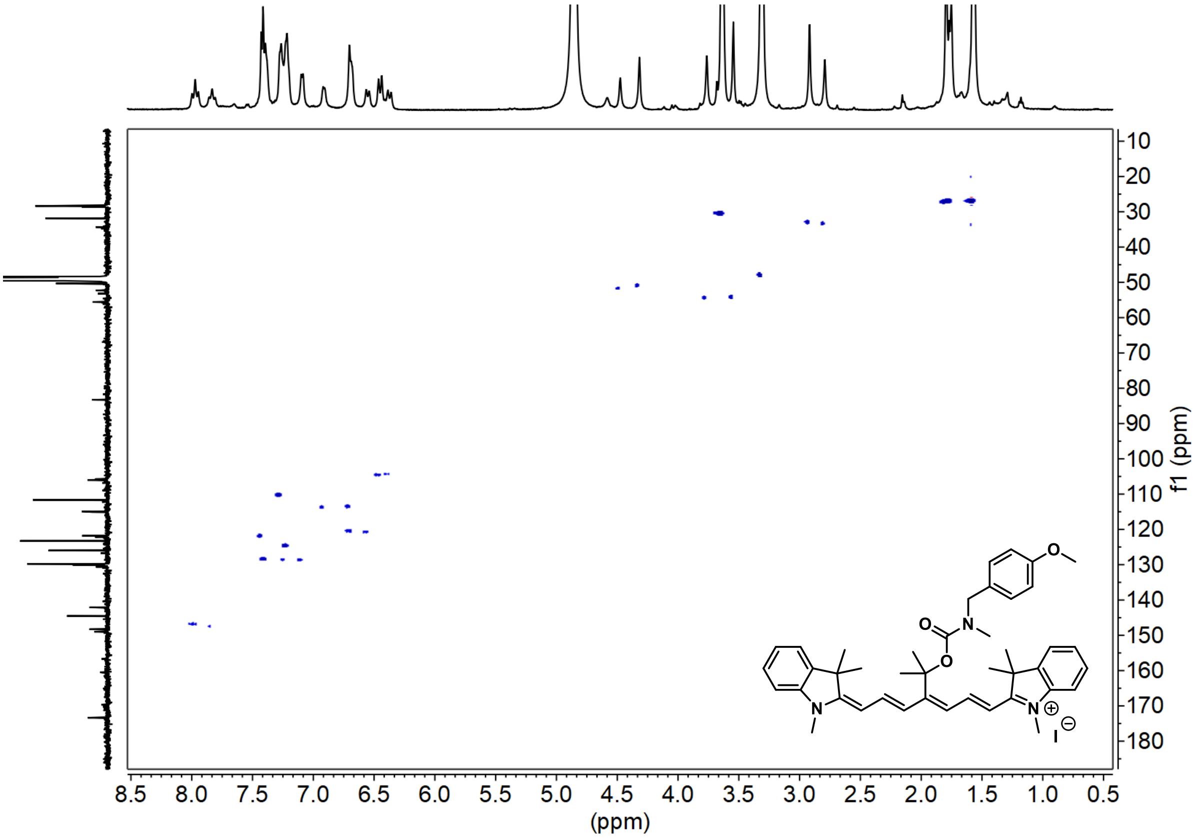

HSQC (500 MHz, 126 MHz, *d*$_4$-CD_3OD): **1h**, mixture of rotamers

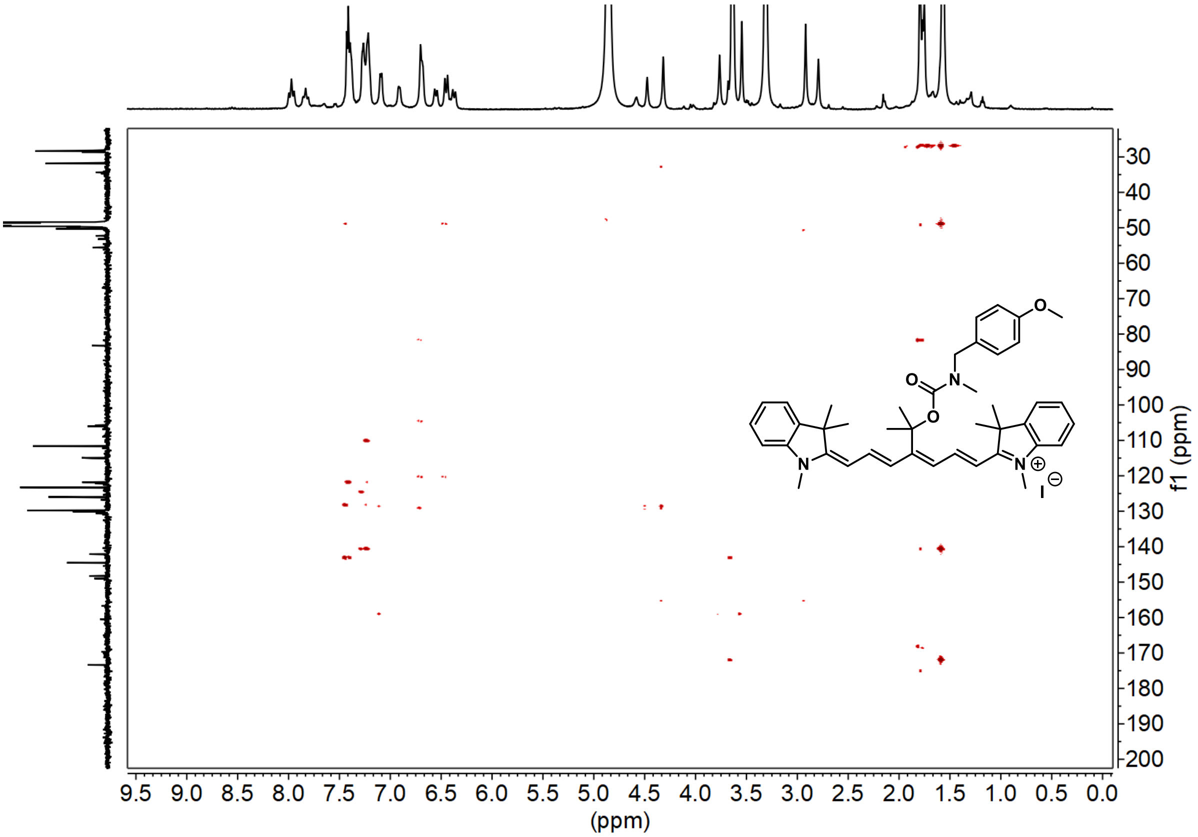

HMBC (500 MHz, 126 MHz, *d*$_4$-CD_3OD): **1h**, mixture of rotamers

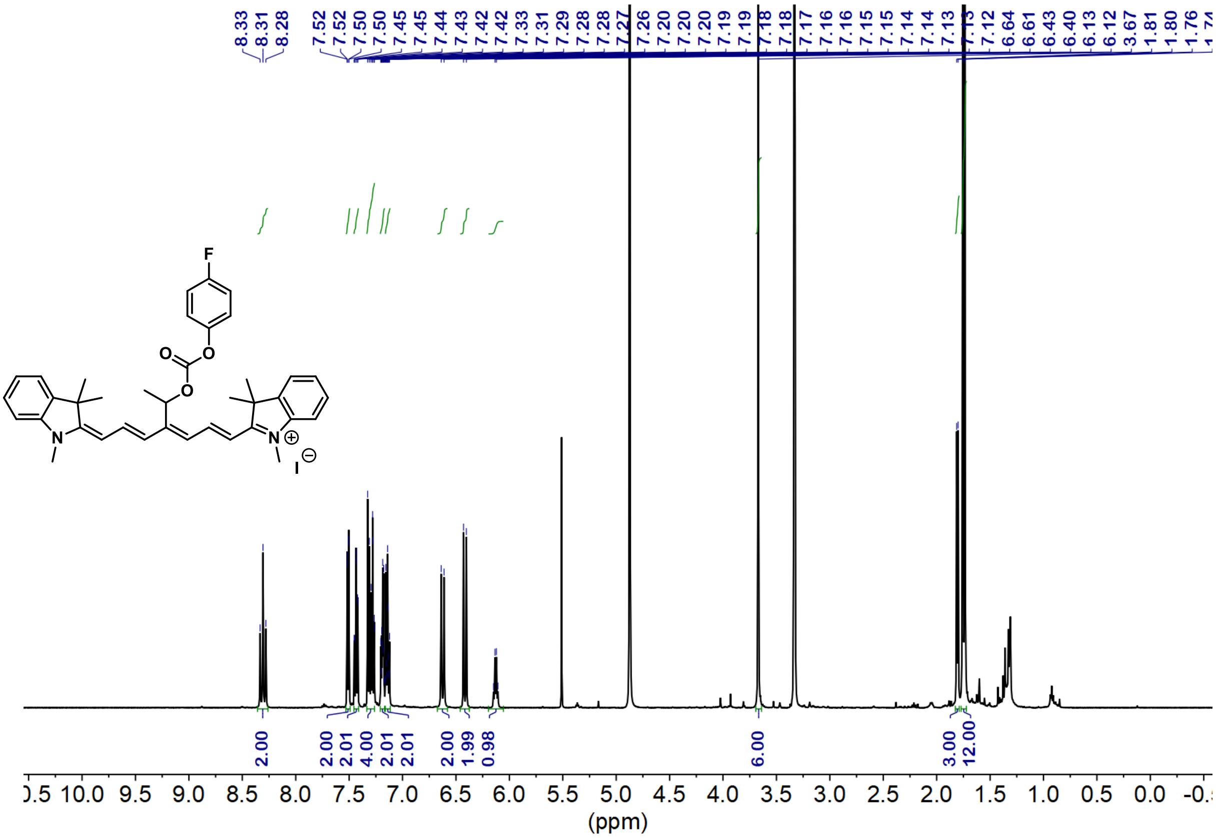

^{1}H NMR (500 MHz, d_4-CD_3OD): **1j**

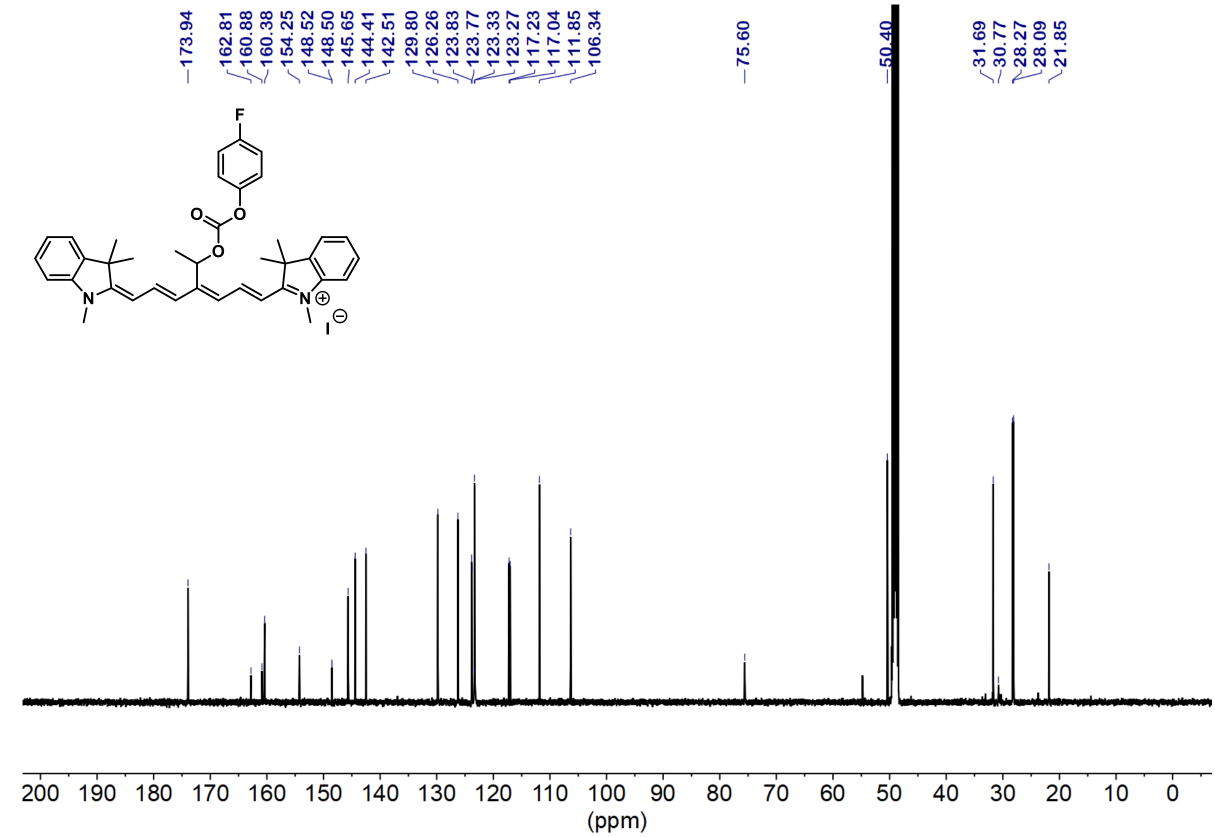

^{13}C NMR (126 MHz, d_4-CD_3OD): **1j**

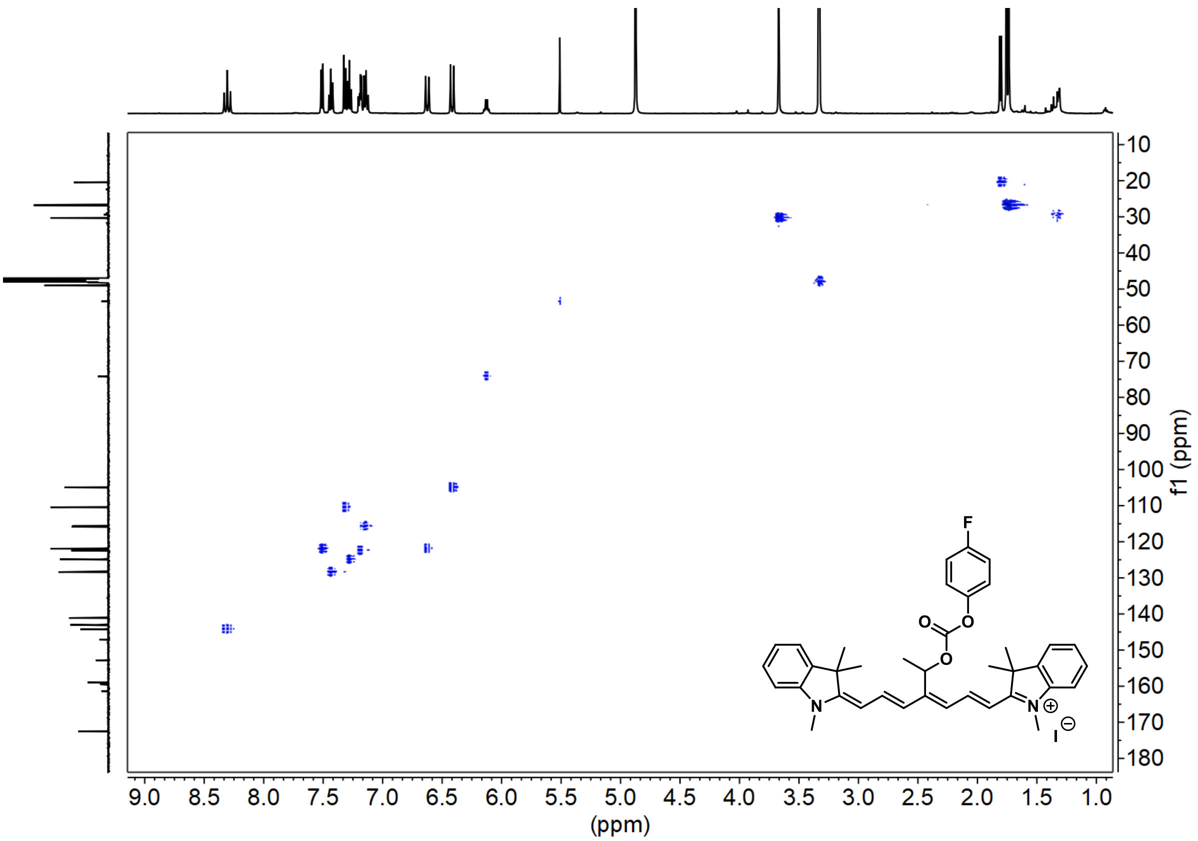

HSQC (500 MHz, 126 MHz, *d*$_4$-CD$_3$OD): **1j**

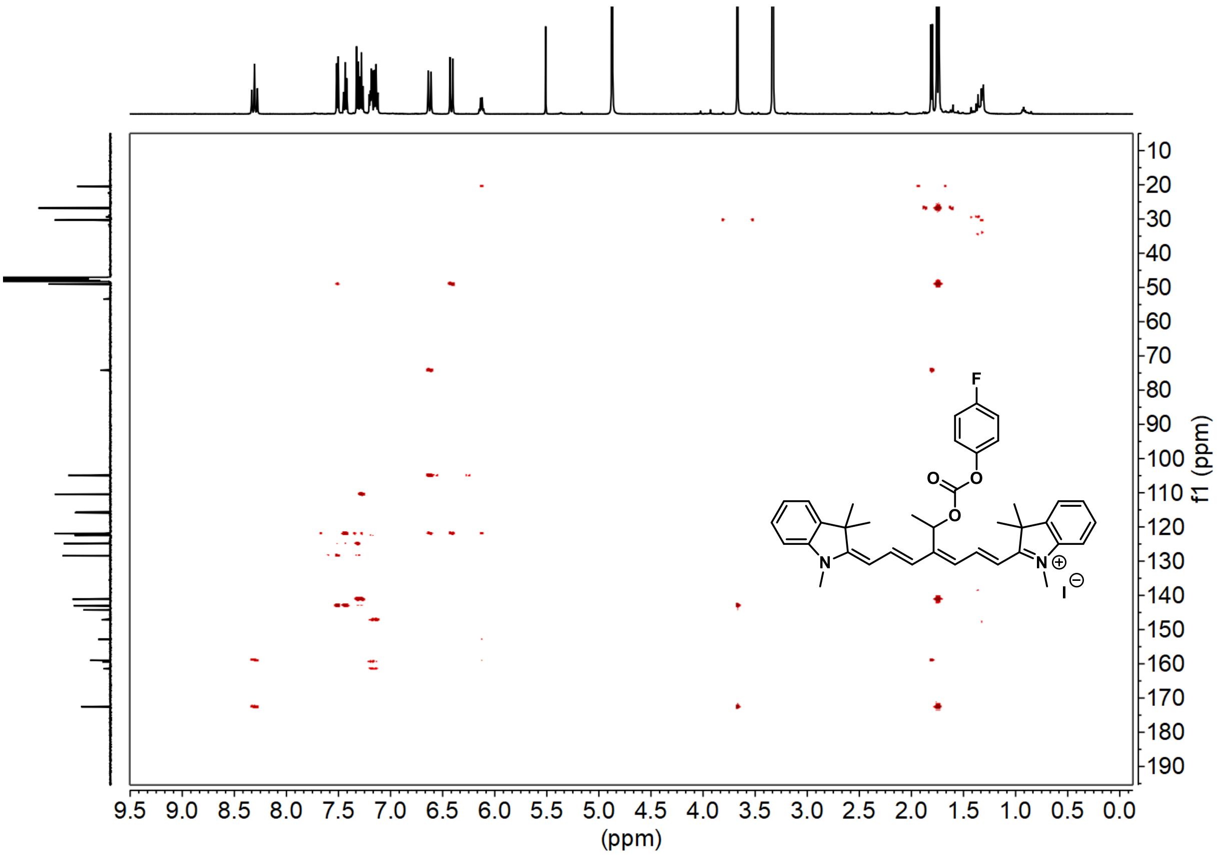

HMBC (500 MHz, 126 MHz, *d*$_4$-CD$_3$OD): **1j**

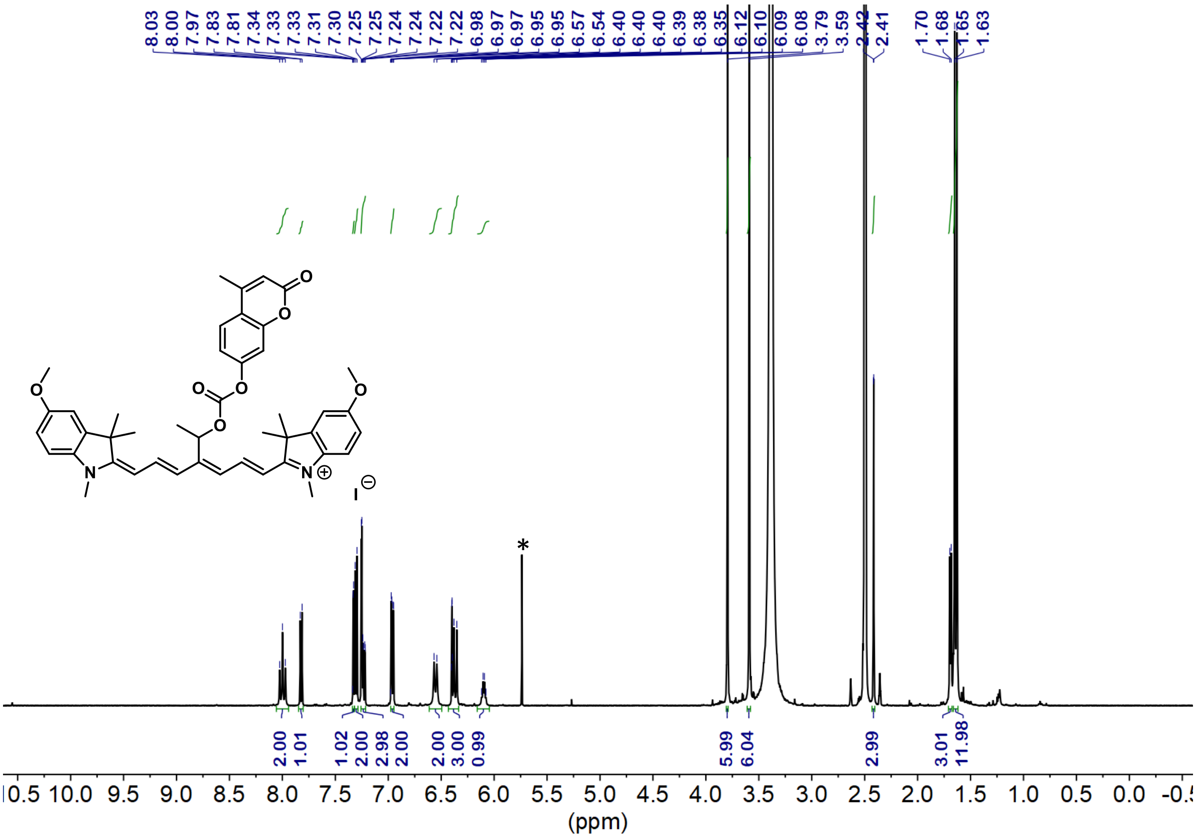

^{1}H NMR (500 MHz, d_6-DMSO): **1k**, *dichloromethane

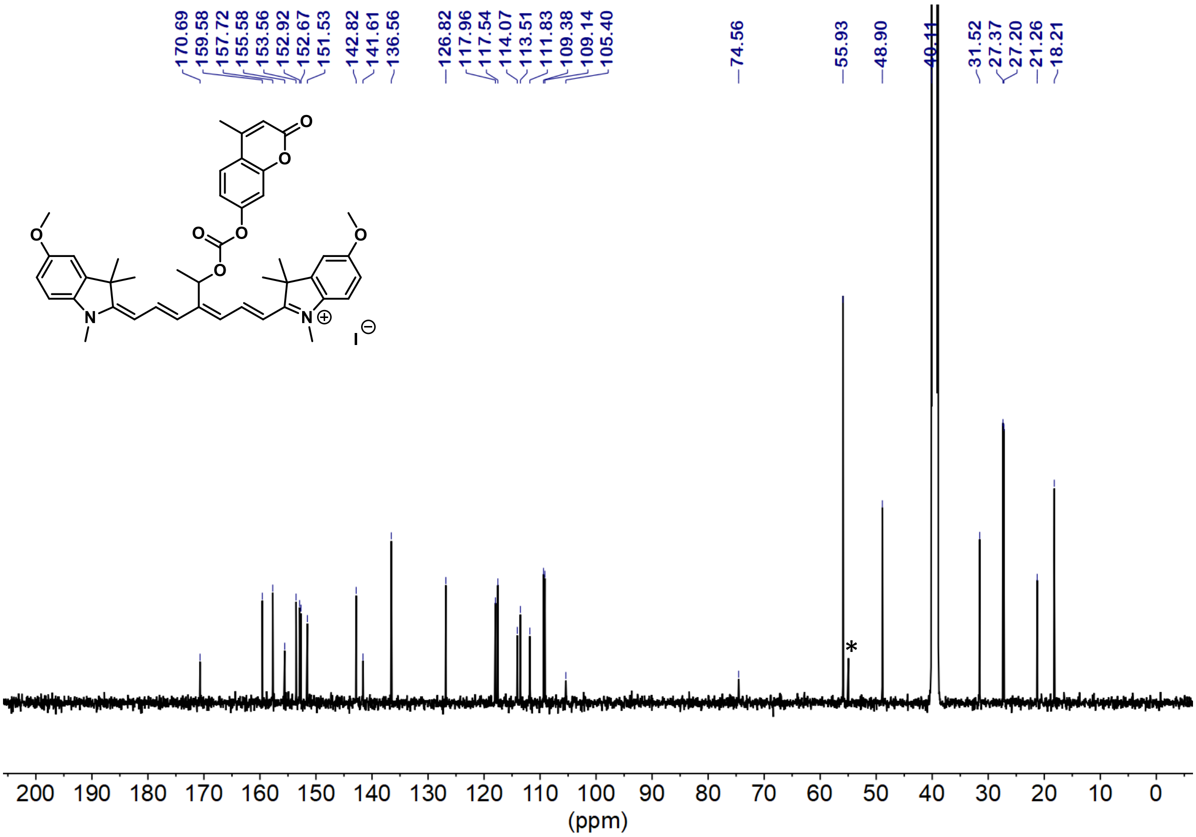

^{13}C NMR (126 MHz, d_6-DMSO): **1k**, *dichloromethane

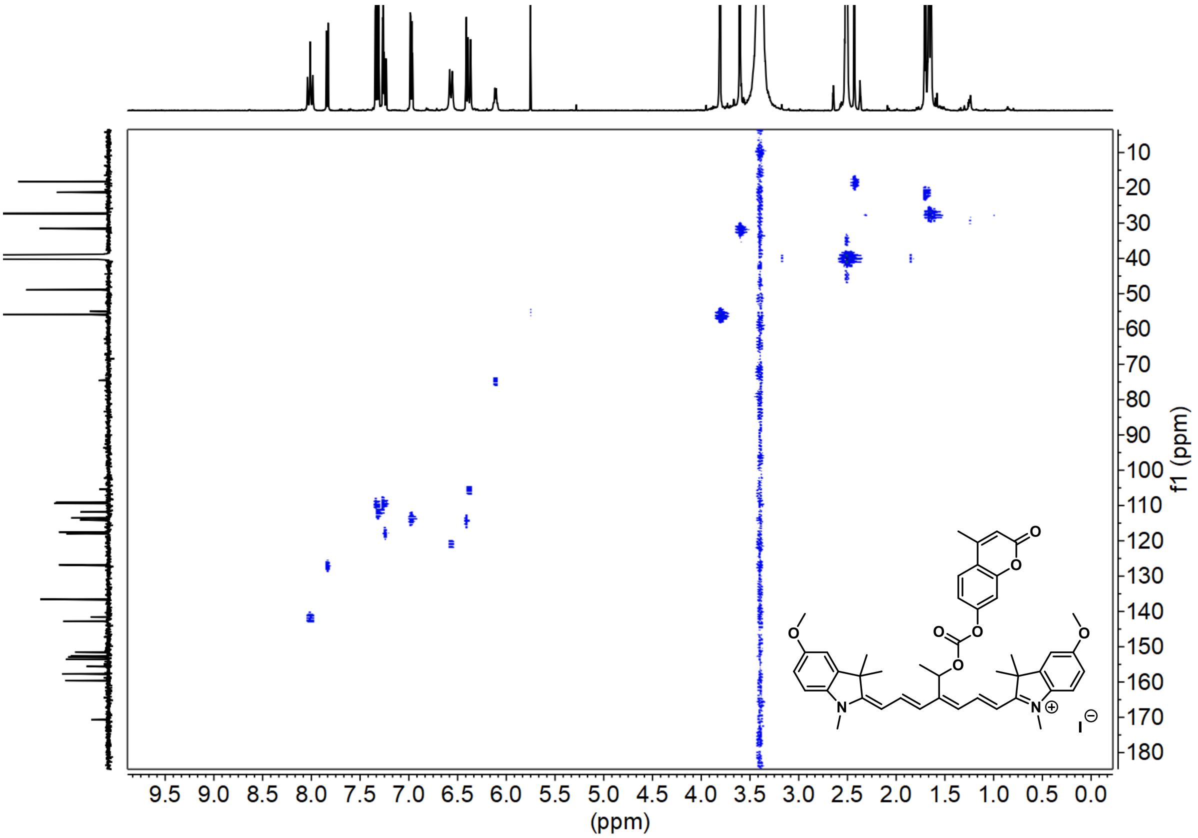

HSQC (500 MHz, 126 MHz, d_6-DMSO): **1k**

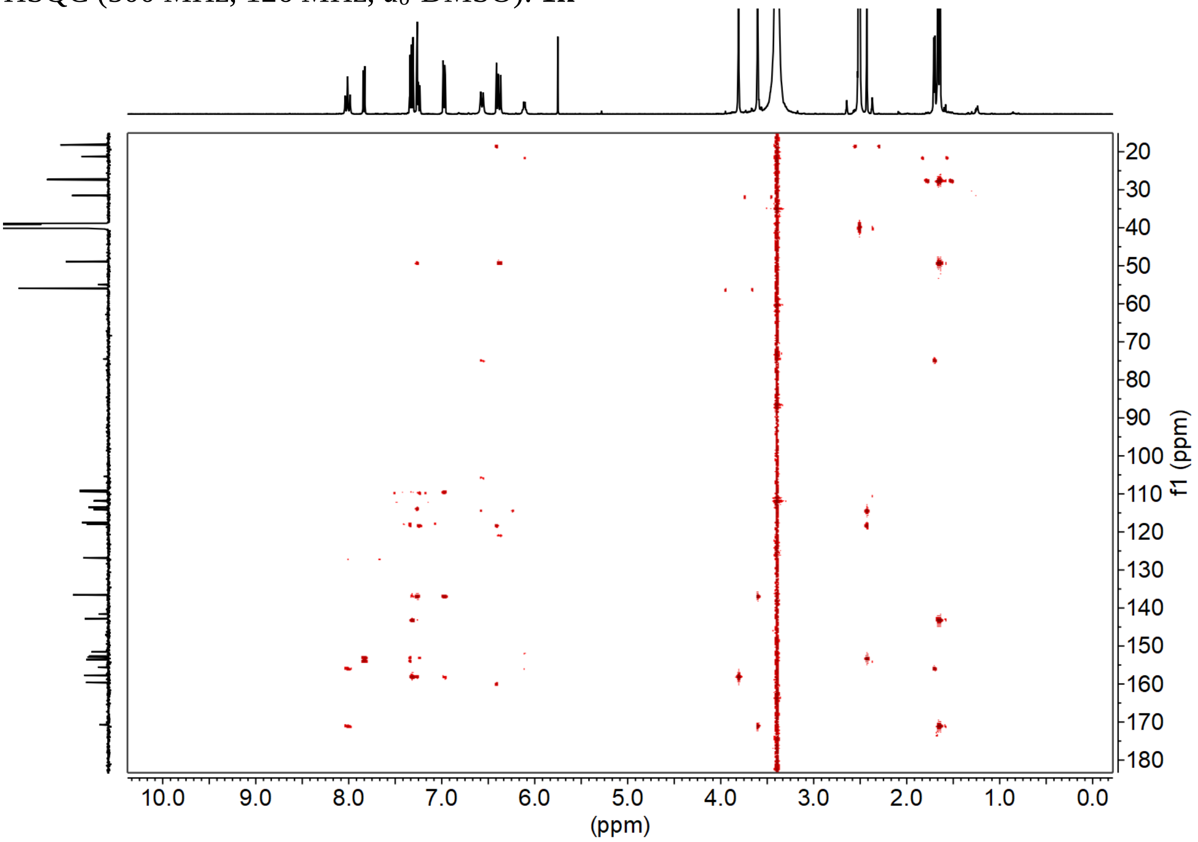

HSQC (500 MHz, 126 MHz, d_6-DMSO): **1k**

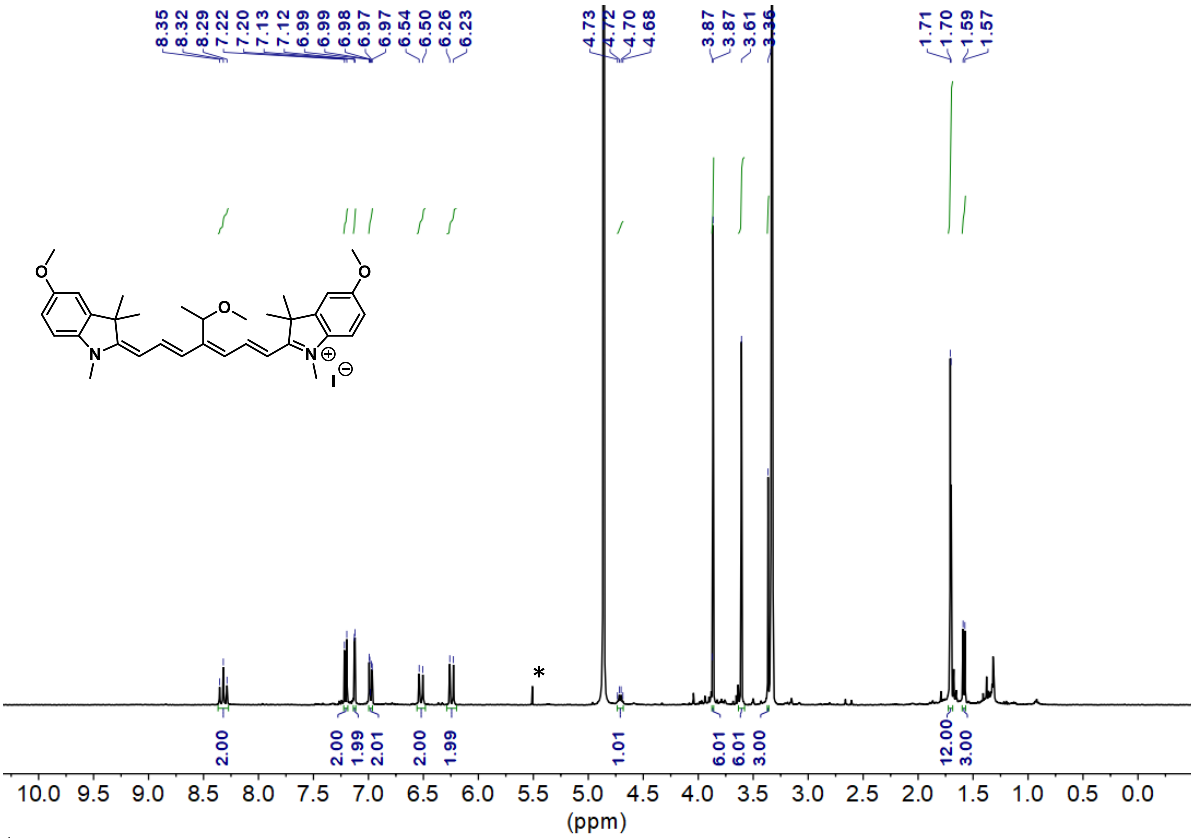

^{1}H NMR (500 MHz, d_4-CD_3OD): **8**, *dichloromethane

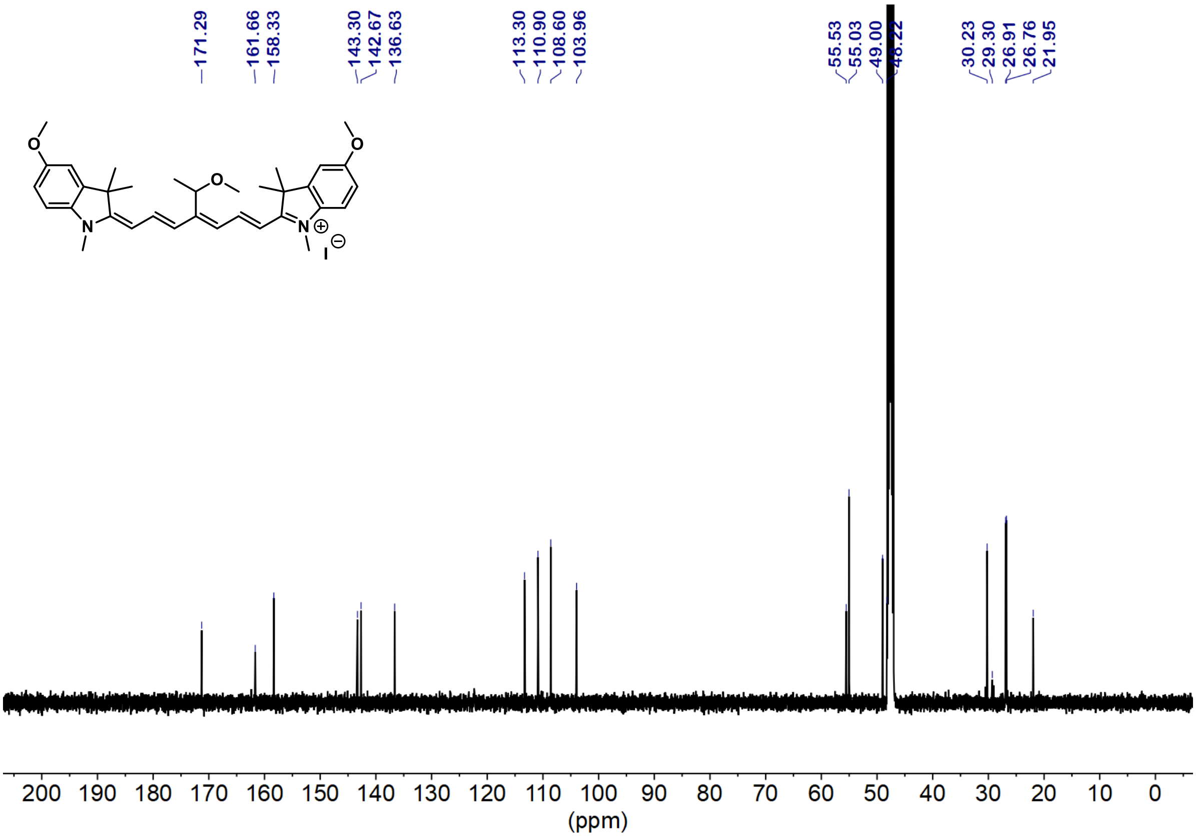

^{13}C NMR (126 MHz, d_4-CD_3OD): **8**

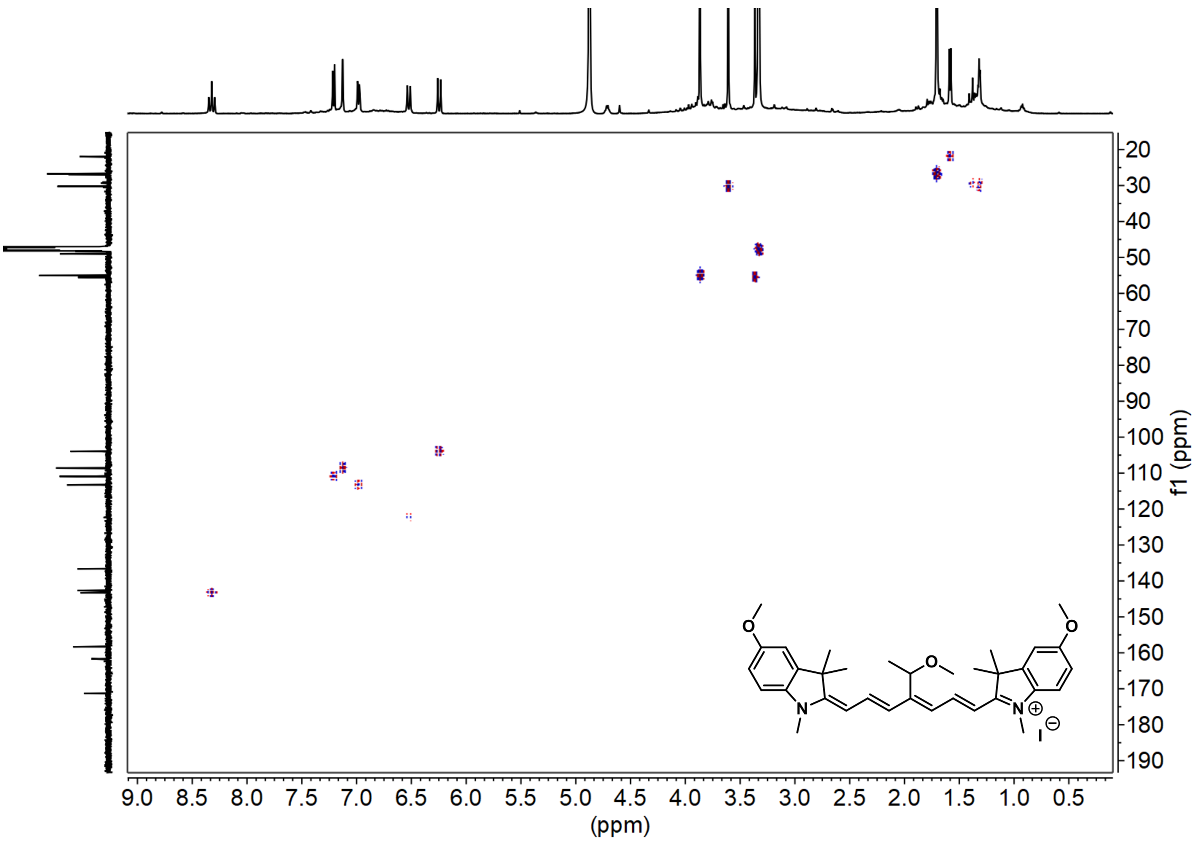

HSQC (500 MHz, 126 MHz, d_4-CD_3OD): **8**

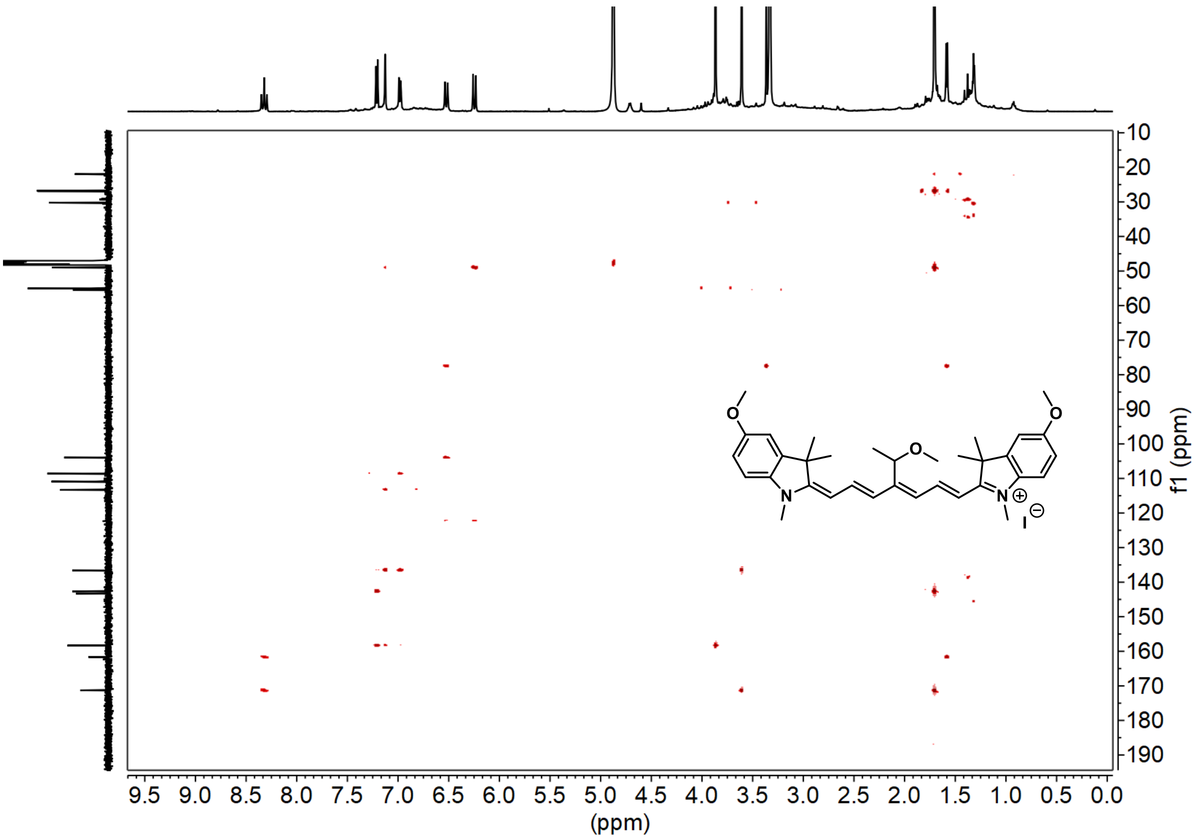

HMBC (500 MHz, 126 MHz, d_4-CD_3OD): **8**

UV-Vis Absorption and Emission Spectroscopy

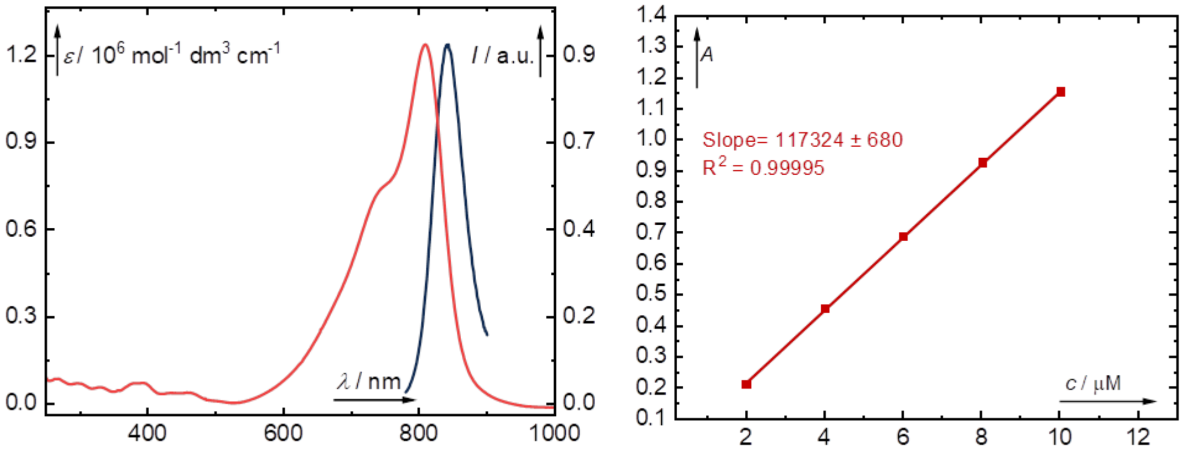

Figure S1. (left) UV-Vis absorption (red) and emission (blue) spectra of **1a** in PBS (100 mM, pH = 7.4) with 20% of DMSO and in methanol, respectively. (right) Dependence of absorption at λ_{max} on the concentration of **1a** in PBS (100 mM, pH = 7.4) with 20% of DMSO (red).

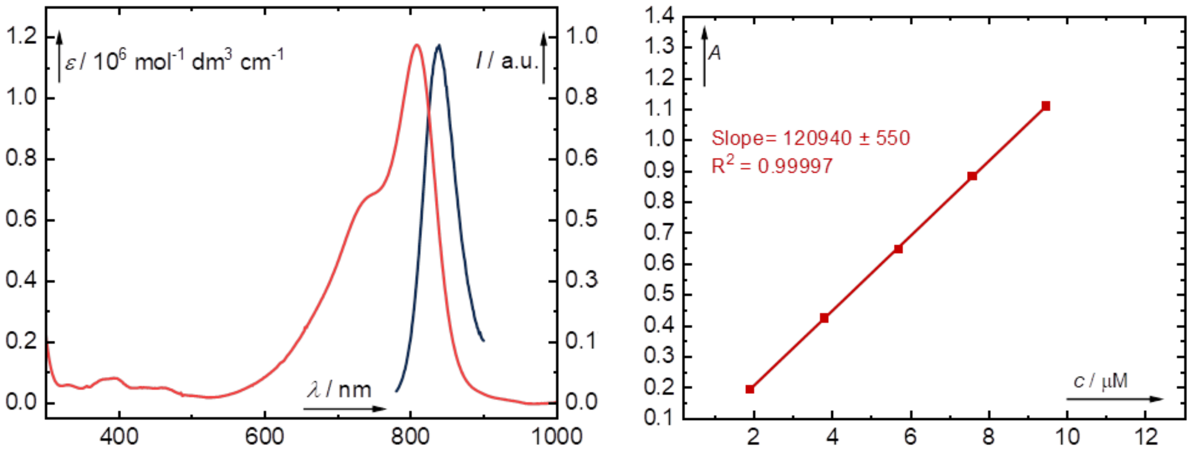

Figure S2. (left) UV-Vis absorption (red) and emission (blue) spectra of **1b** in PBS (100 mM, pH = 7.4) with 20% of DMSO and in methanol, respectively. (right) Dependence of absorption at λ_{max} on the concentration of **1b** in PBS (100 mM, pH = 7.4) with 20% of DMSO (red).

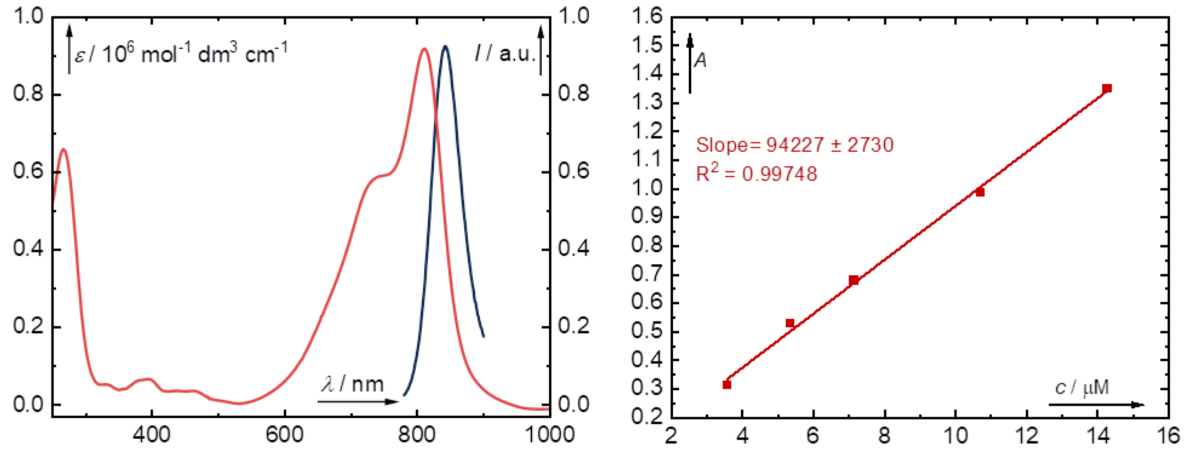

Figure S3. (left) UV-Vis absorption (red) and emission (blue) spectra of **1c** in PBS (100 mM, pH = 7.4) with 20% of DMSO and in methanol, respectively. (right) Dependence of absorption at λ_{max} on the concentration of **1c** in PBS (100 mM, pH = 7.4) with 20% of DMSO (red).

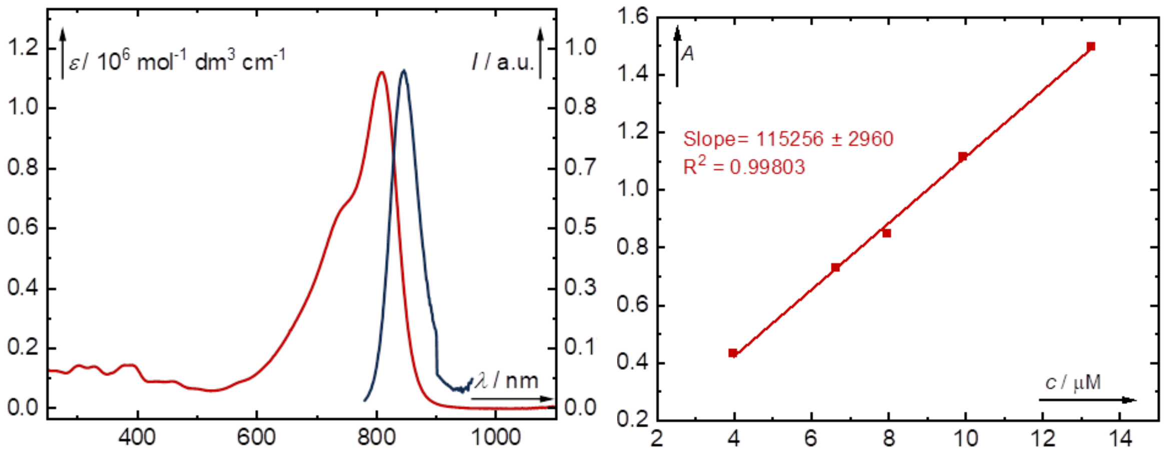

Figure S4. (left) UV-Vis absorption (red) and emission (blue) spectra of **1d** in PBS (100 mM, pH = 7.4) with 20% of DMSO and in methanol, respectively. (right) Dependence of absorption at λ_{max} on the concentration of **1d** in PBS (100 mM, pH = 7.4) with 20% of DMSO (red).

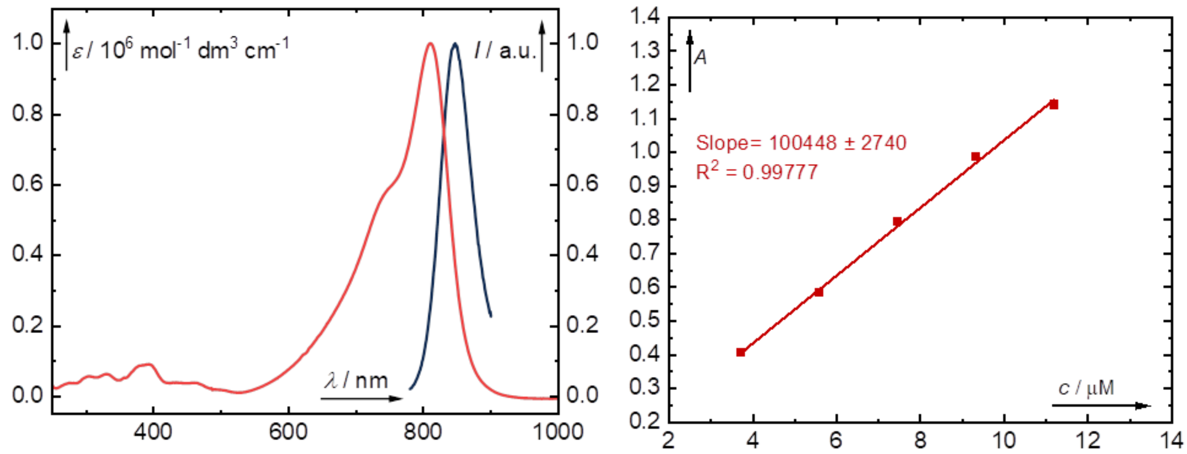

Figure S5. (left) UV-Vis absorption (red) and emission (blue) spectra of **1e** in PBS (100 mM, pH = 7.4) with 20% of DMSO and in methanol, respectively. (right) Dependence of absorption at λ_{max} on the concentration of **1e** in PBS (100 mM, pH = 7.4) with 20% of DMSO (red).

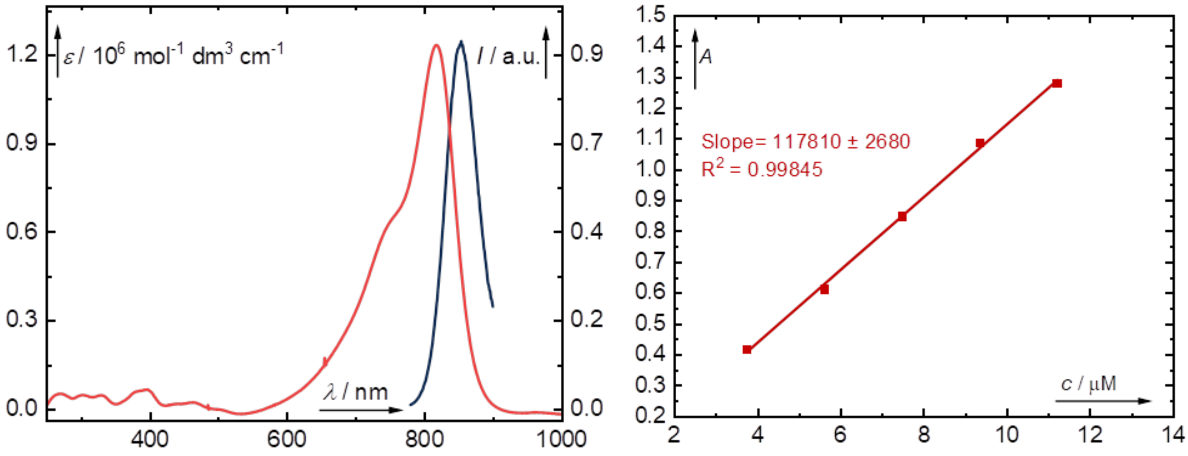

Figure S6. (left) UV-Vis absorption (red) and emission (blue) spectra of **1f** in PBS (100 mM, pH = 7.4) with 20% of DMSO and in methanol, respectively. (right) Dependence of absorption at λ_{max} on the concentration of **1f** in PBS (100 mM, pH = 7.4) with 20% of DMSO (red).

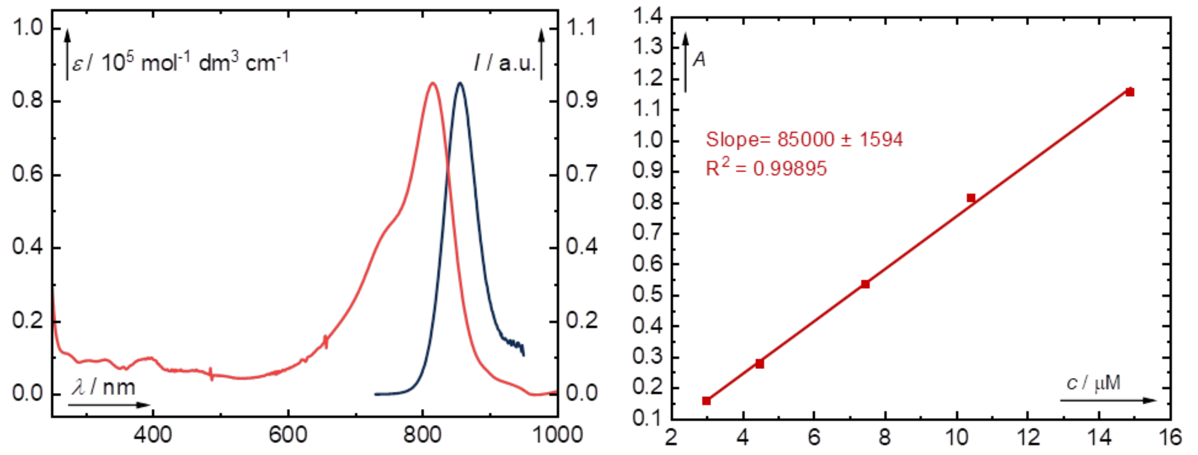

Figure S7. (left) UV-Vis absorption (red) and emission (blue) spectra of **1g** in PBS (100 mM, pH = 7.4) with 20% of DMSO and in methanol, respectively. (right) Dependence of absorption at λ_{max} on the concentration of **1g** in PBS (100 mM, pH = 7.4) with 20% of DMSO (red).

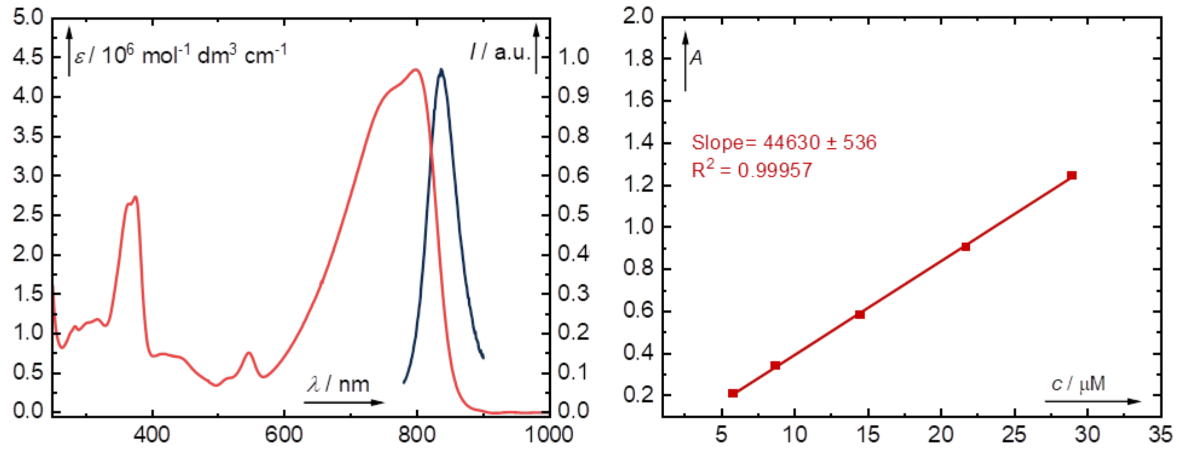

Figure S8. (left) UV-Vis absorption (red) and emission (blue) spectra of **1h** in PBS (100 mM, pH = 7.4) with 20% of DMSO and in methanol, respectively. (right) Dependence of absorption at λ_{max} on the concentration of **1h** in MeOH (red).

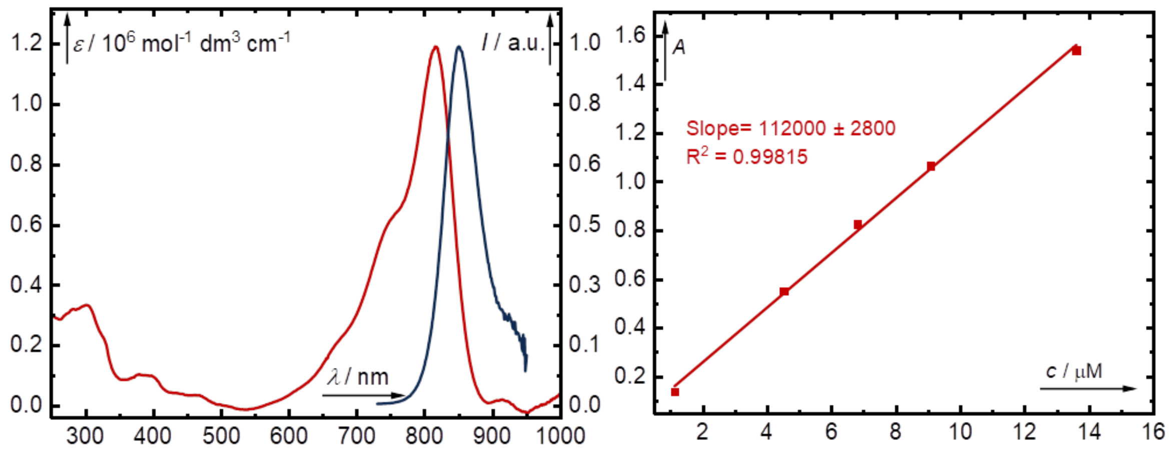

Figure S9. (left) UV-Vis absorption (red) and emission (blue) spectra of **1k** in methanol. (right) Dependence of absorption at λ_{max} on the concentration of **1k** in methanol (red).

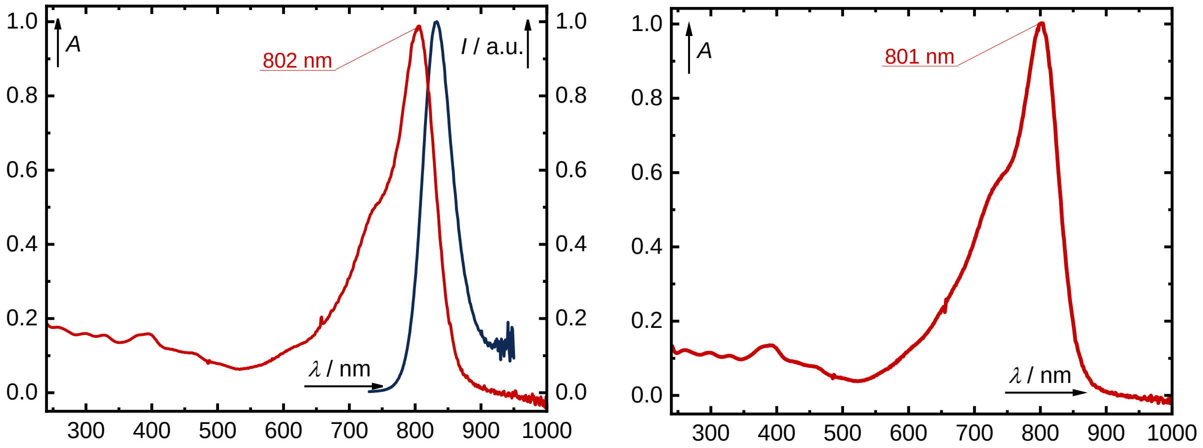

Figure S10. UV-Vis absorption (red) and emission (blue) spectra of **8** MeOH (left) and absorption spectrum of **8** in PBS (100 mM, pH = 7.4, 20% DMSO) (right).

Photophysical and Photochemical Measurements

Irradiation of the photocages followed by UV-Vis Spectroscopy

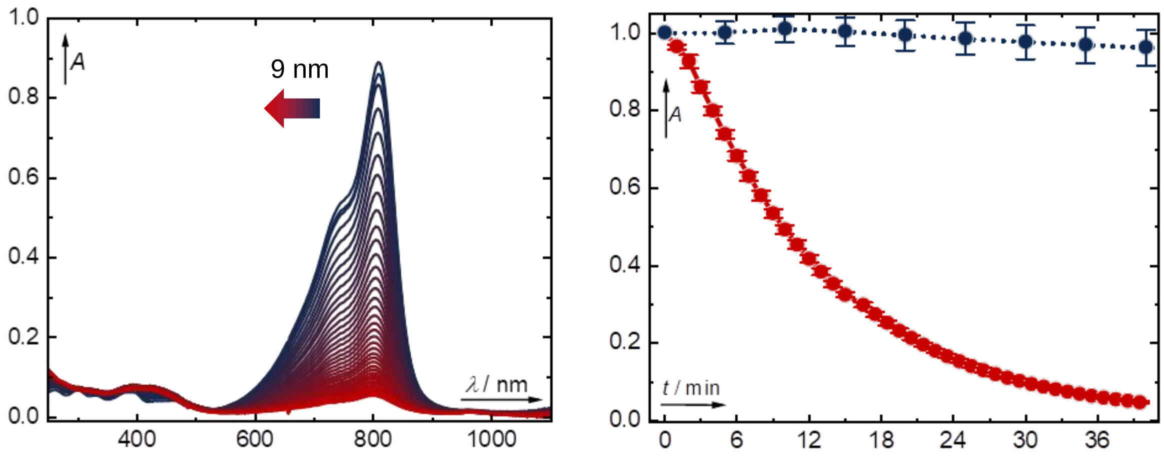

Figure S11. (left) Irradiation of **1a** at 820 nm in PBS (100 mM, pH = 7.4) with 20% of DMSO at ambient conditions followed by UV-vis spectroscopy in 30 sec intervals (blue to red). (right) Kinetic traces measured at λ = 786 nm for **1a** in dark (blue) and under irradiation at 820 nm (red) PBS (100 mM, pH = 7.4) with 20% of DMSO. Normalized to A = 1.0 at t = 0 min. The error bars represent standard deviation of the mean from three independent samples.

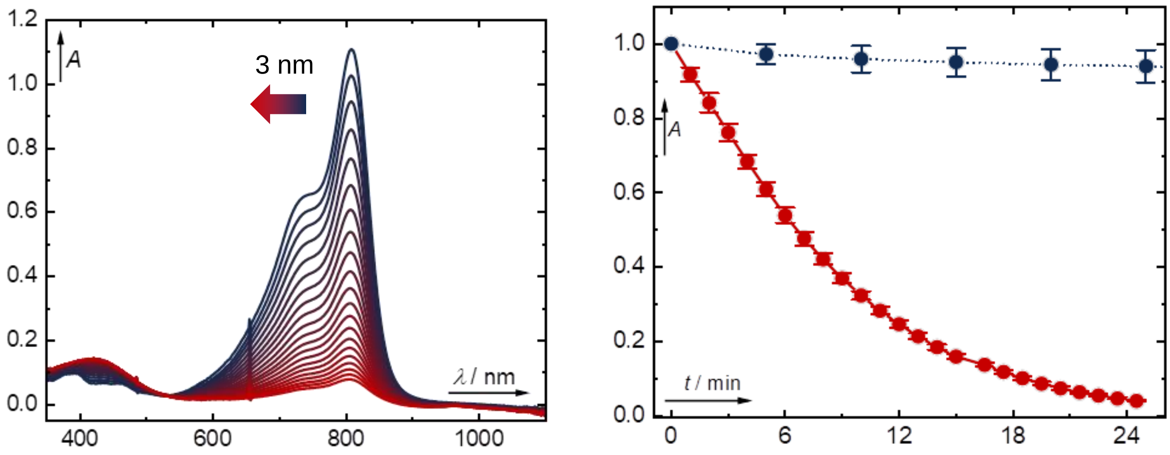

Figure S12. (left) Irradiation of **1b** at 820 nm in PBS (100 mM, pH = 7.4) with 20% of DMSO at ambient conditions followed by UV-vis spectroscopy in 60 sec intervals (blue to red). (right) Kinetic traces measured at λ = 786 nm for **1b** in dark (blue) and under irradiation at 820 nm (red) PBS (100 mM, pH = 7.4) with 20% of DMSO. Normalized to A = 1.0 at t = 0 min. The error bars represent standard deviation of the mean from three independent samples.

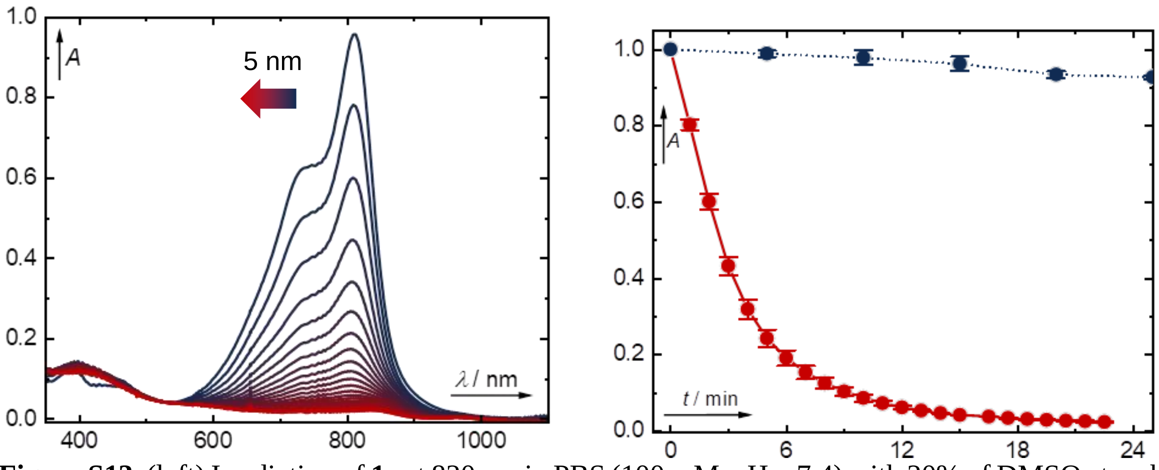

Figure S13. (left) Irradiation of **1c** at 820 nm in PBS (100 mM, pH = 7.4) with 20% of DMSO at ambient conditions followed by UV-vis spectroscopy in 60-sec intervals (blue to red). (right) Kinetic traces measured at λ = 786 nm for **1c** in dark (blue) and under irradiation at 820 nm (red) PBS (100 mM, pH = 7.4) with 20% of DMSO. Normalized to A = 1.0 at t = 0 min. The error bars represent standard deviation of the mean from three independent samples.

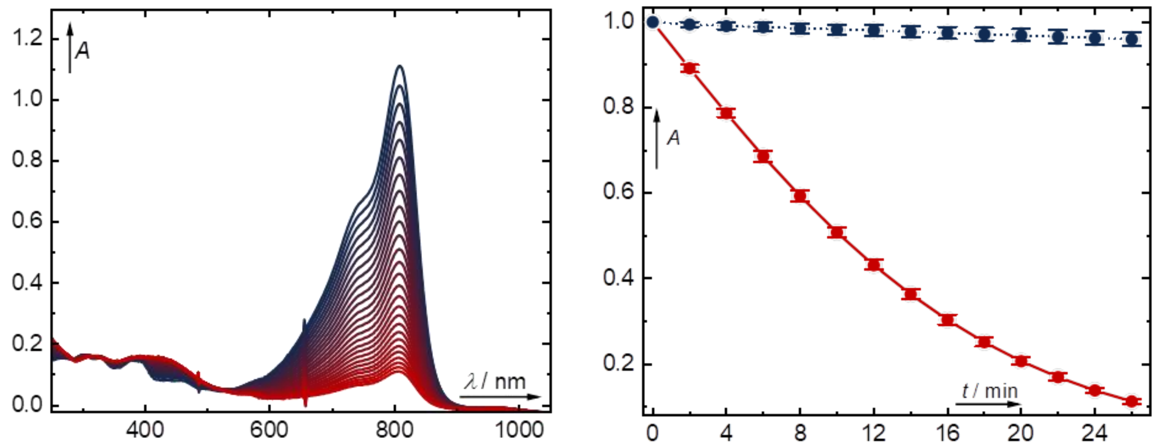

Figure S14. (left) Irradiation of **1d** at 820 nm in PBS (100 mM, pH = 7.4) with 20% of DMSO at ambient conditions followed by UV-vis spectroscopy in 60-sec intervals (blue to red). (right) Kinetic traces measured at λ = 786 nm for **1d** in dark (blue) and under irradiation at 820 nm (red) PBS (100 mM, pH = 7.4) with 20% of DMSO. Normalized to A = 1.0 at t = 0 min. The error bars represent standard deviation of the mean from three independent samples.

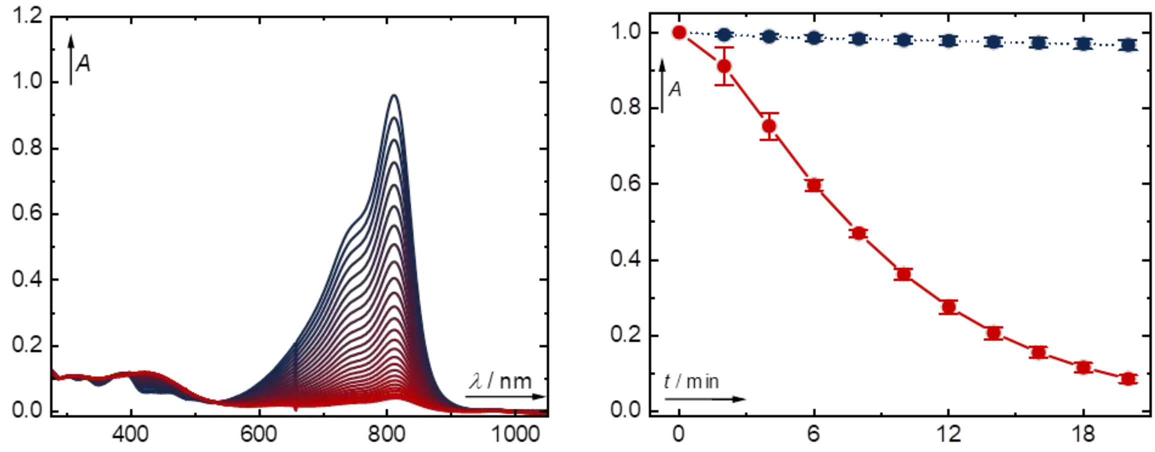

Figure S15. (left) Irradiation of **1e** at 820 nm in PBS (100 mM, pH = 7.4) at ambient conditions with 20% of DMSO followed by UV-vis spectroscopy in 60-sec intervals (blue to red). (right) Kinetic traces

measured at λ = 786 nm for **1e** in dark (blue) and under irradiation at 820 nm (red) PBS (100 mM, pH = 7.4) with 20% of DMSO. Normalized to A = 1.0 at t = 0 min. The error bars represent standard deviation of the mean from three independent samples.

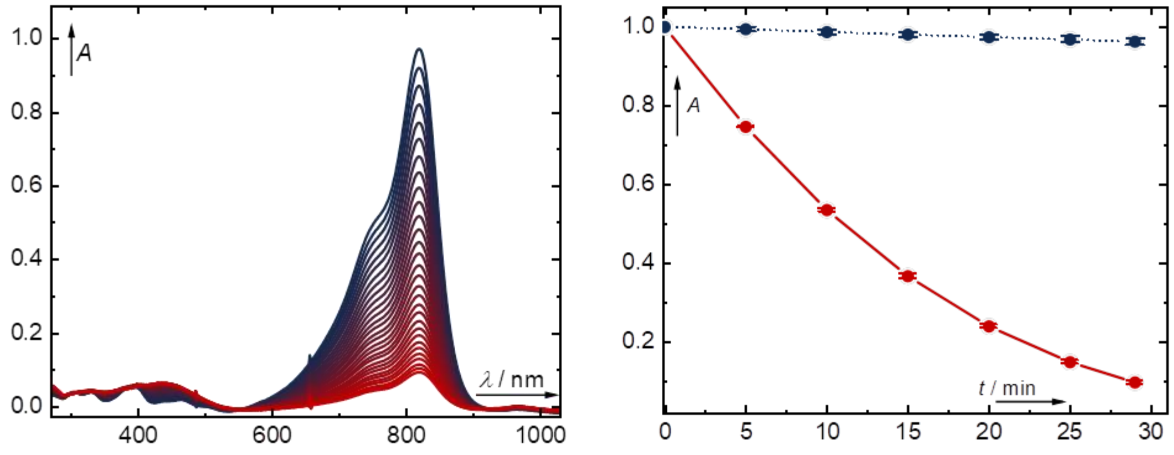

Figure S16. (left) Irradiation of **1f** at 820 nm in PBS (100 mM, pH = 7.4) with 20% of DMSO at ambient conditions followed by UV-vis spectroscopy in 60-sec intervals (blue to red). (right) Kinetic traces measured at λ = 786 nm for **1f** in dark (blue) and under irradiation at 820 nm (red) PBS (100 mM, pH = 7.4) with 20% of DMSO. Normalized to A = 1.0 at t = 0 min. The error bars represent standard deviation of the mean from three independent samples.

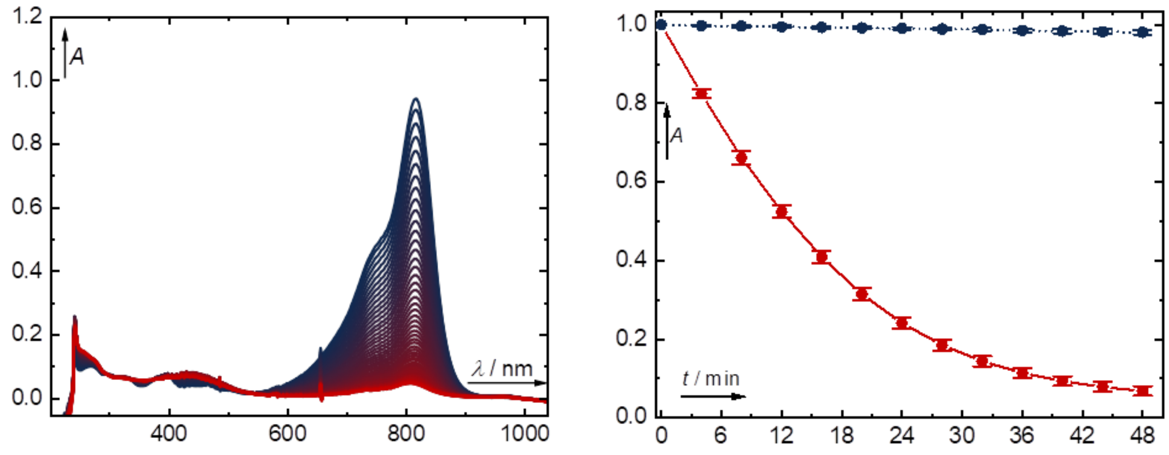

Figure S17. (left) Irradiation of **1h** at 820 nm in MeOH at ambient conditions followed by UV-vis spectroscopy in 60-sec intervals (blue to red). (right) Kinetic traces measured at λ = 786 nm for **1h** in dark (blue) and under irradiation at 820 nm (red) MeOH. Normalized to A = 1.0 at t = 0 min. The error bars represent standard deviation of the mean from three independent samples.

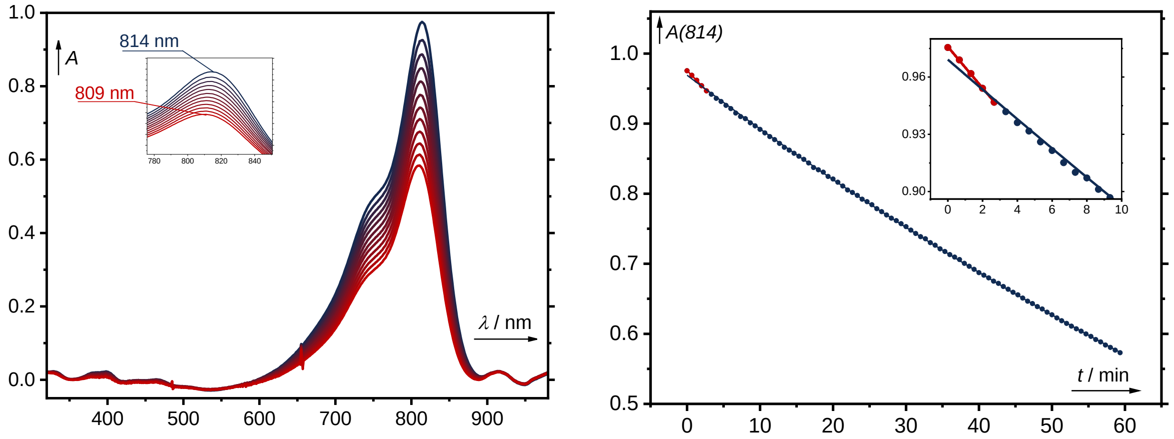

Figure S18. UV-Vis spectra (left) and the respective traces (right) of photocage **1a** irradiated in MeOH (820 nm) under ambient conditions. Minor change in the slope suggests the presence of two distinct photoprocesses (i.e. photoheterolysis followed by photooxidation).

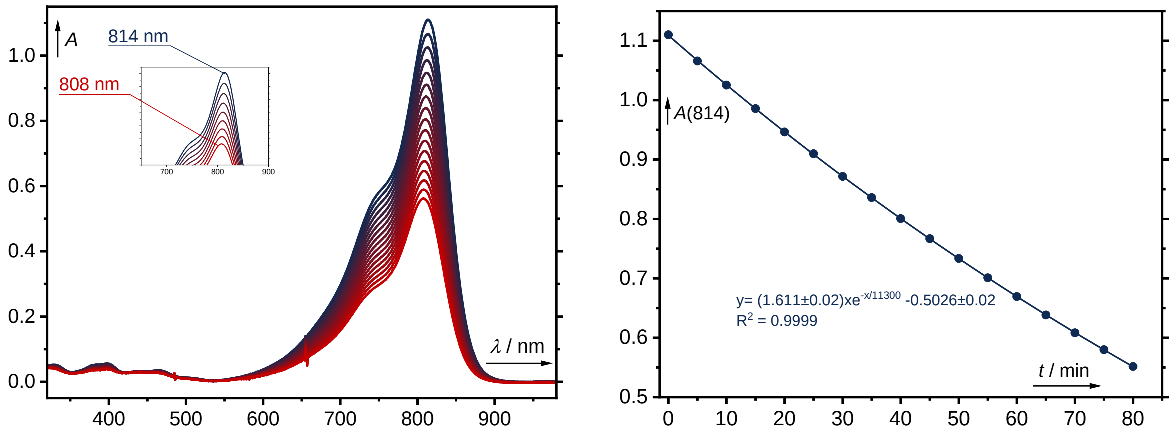

Figure S19. UV-Vis spectra (left) and the respective traces (right) of photocage **1a** irradiated in MeOH (820 nm) under O_2–free conditions.

Irradiation of 1k Followed by Emission Spectroscopy

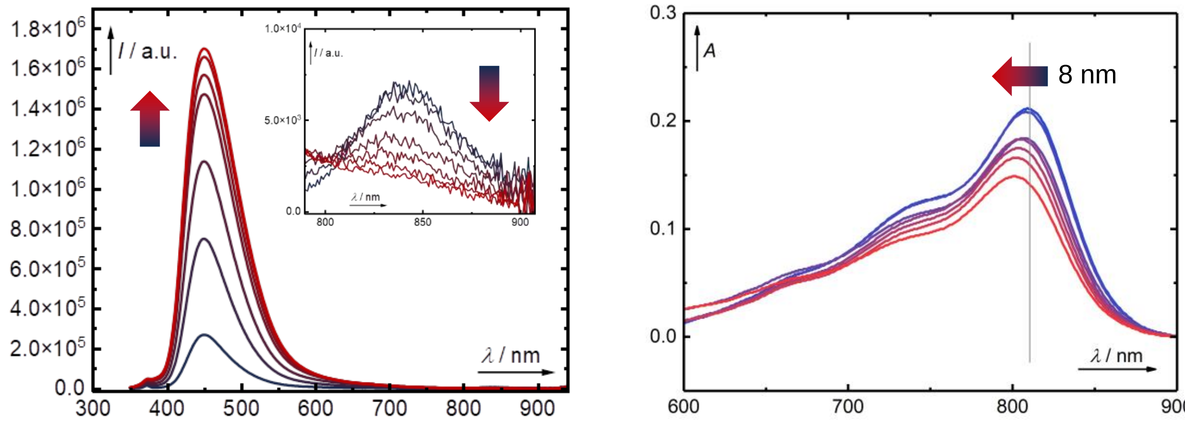

Figure S19.(left) Emission spectra (λ_{exc} = 340 nm) of **1k** (c ~ 3 × 10^{-6} M) irradiated at 820 nm in PBS (100 mM, pH = 7.4 with 25% of DMSO) at ambient conditions. Spectra at 0, 30, 90, 180, 240, 300, 360 s are given (blue to red). The inset shows the Cy7 region of the spectra. (right) UV-Vis absorption spectra from the same experiment but performed in PBS purged with Ar for 5 min. Note that the majority of the absorbance remains after the irradiation, but a noticable 8 nm blue shift is still observed.

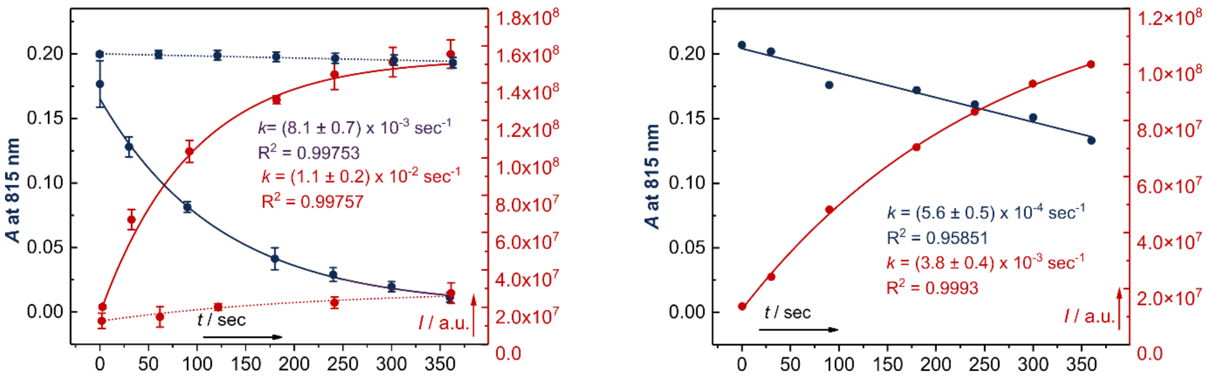

Figure S20. Traces of the absorbance (blue) and emission intensity of 4-methylumbelliferone (red) observed upon irradiation of **1k** (c ~ 3 × 10^{-6} M) at 820 nm in PBS (100 mM, pH = 7.4 with 25% of DMSO) under (left) ambient conditions and (right) purged by Ar bubbling for 5 mins. The solid lines represent the exponential fit of the traces. The given rate constants are calculated from the exponential fit. The dotted lines represent the control experiment kept in the dark.

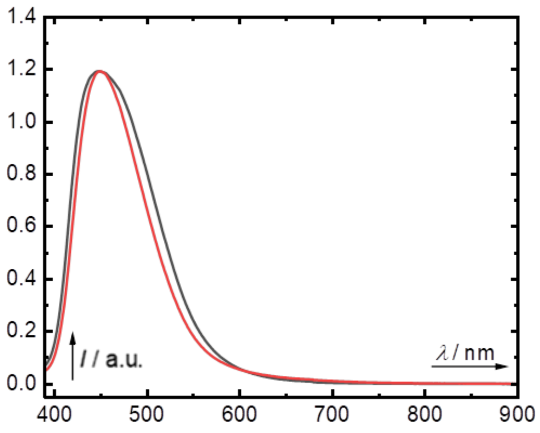

Figure S21. Overlay of emission spectra of 4-methylumbelliferone (black) in PBS (100 mM, pH = 7.4 with 25% of DMSO) and **1k** (c ~ 3 × 10^{-6} M) irradiated at 820 nm for 6 min in aerated PBS (100 mM, pH = 7.4 with 25% of DMSO).

Irradiation in of 1a and 1e in Oxygen-Free Aqueous Media

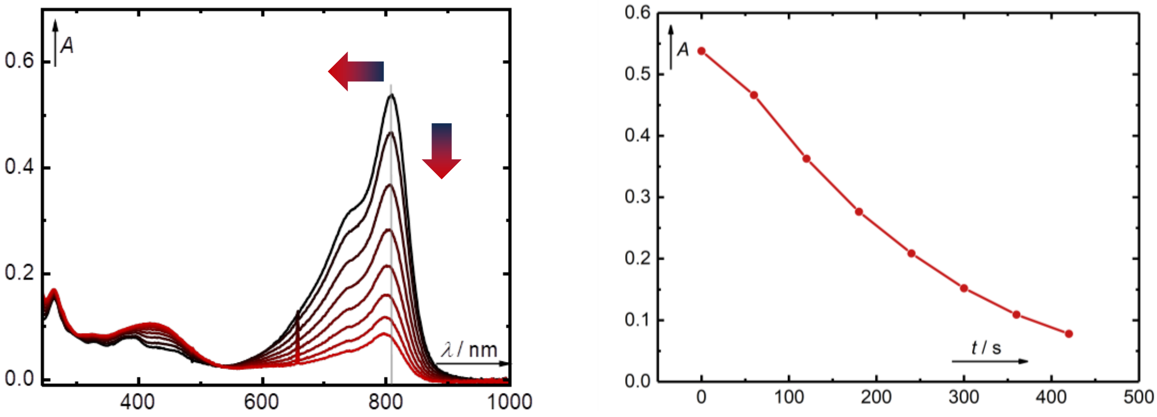

Figure S22. (left) Irradiation of **1a** (c ~ 6 × 10^{-6} M) at 820 nm in PBS (pH 7.4, 10 mM, with 25% DMSO) degassed by freeze-pump-thaw followed by UV-vis spectroscopy in 1-min intervals (black to red). (right) Kinetic traces measured at λ_{max} = 809 nm.

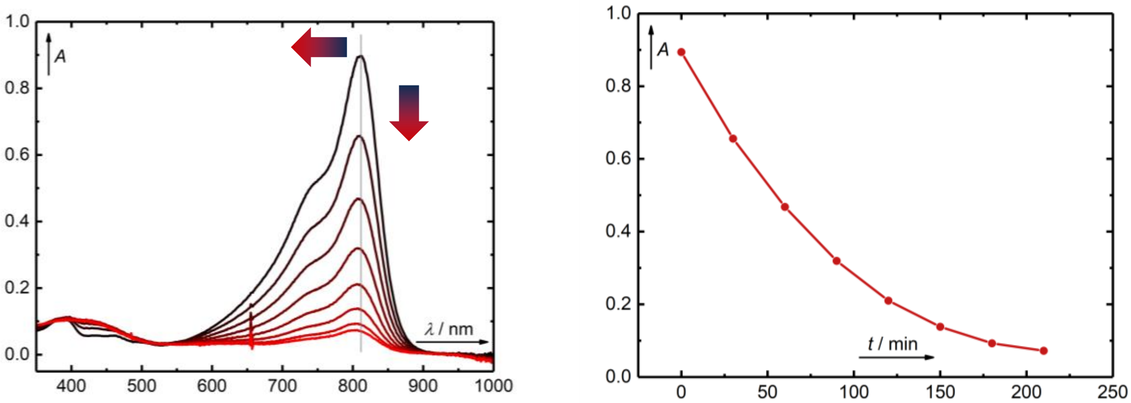

Figure S23. (left) Irradiation of **1e** (c ~ 1.5 × 10^{-5} M) at 820 nm in PBS (pH 7.4, 10 mM, with 25% DMSO) degassed by freeze-pump-thaw followed by UV-vis spectroscopy in 30-min intervals (black to red). (right) Comparison of normalized spectra before (black) and after (red) irradiation at 820 nm.

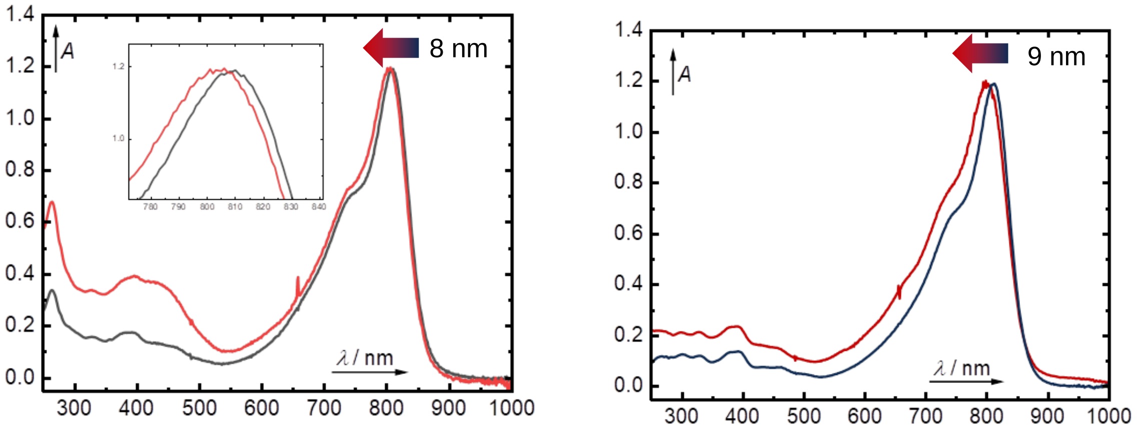

Figure S24. Comparison of normalized spectra before (blue) and after (red) irradiation of (left) **1a** or (right) **1e** at 820 nm in O_2-free PBS (pH 7.4, 10 mM, with 25% DMSO; samples above).

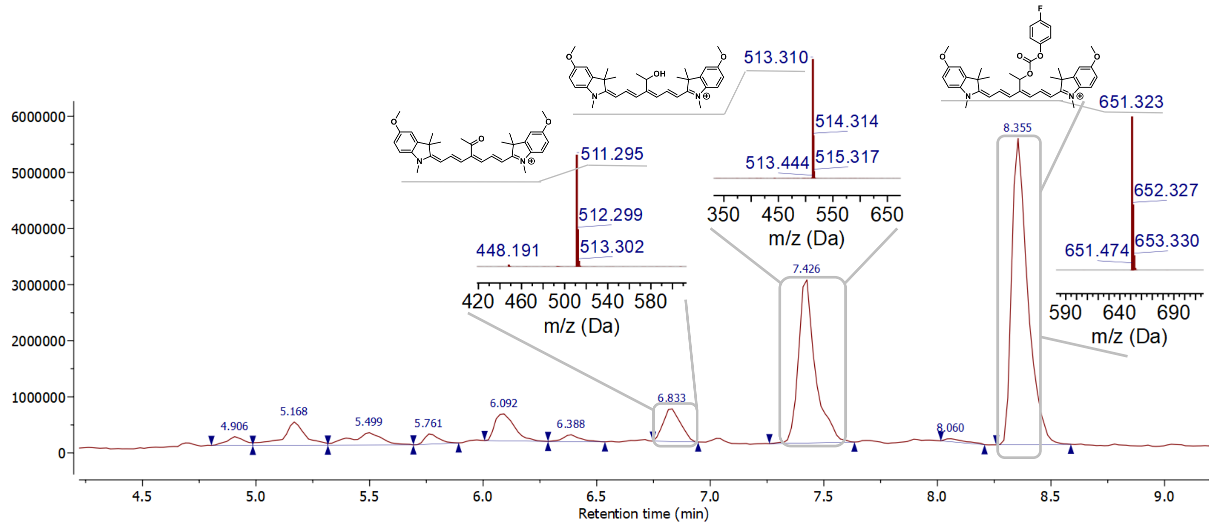

Figure S25. Total Ion Count (TIC, extracted from LC-HRMS) chromatogram of photocage **1a** in MeCN/H_2O (1:1, c = 6×10^{-5} M) irradiated under O_2-free conditions with light at 820 nm for 90 minutes. Presence of m/z 513.29 and 511.3 as a proof of direct release from carbonate photocages in water media.

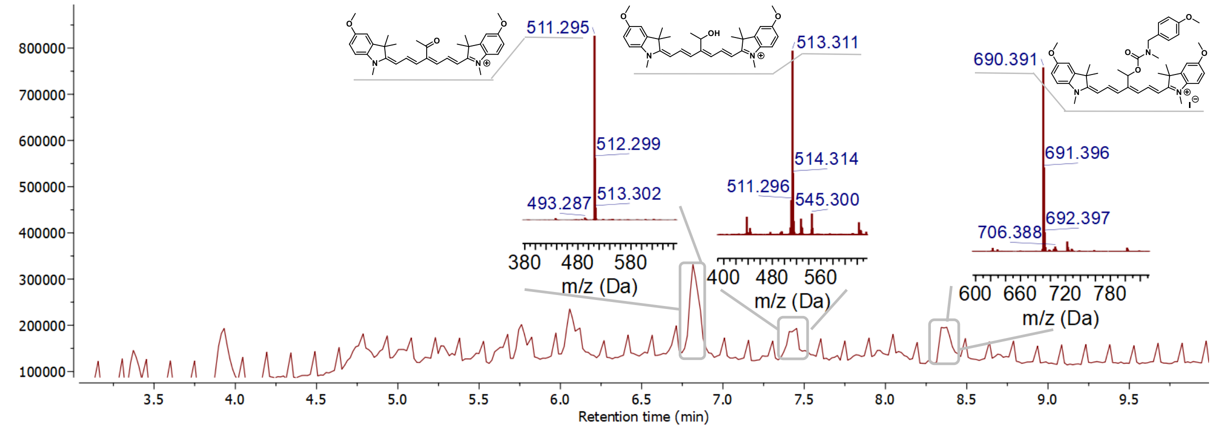

Figure S26. Total Ion Count (TIC, extracted from LC-HRMS) chromatogram of photocage **1e** in DMSO/H_2O (25:75, c = 1.5×10^{-5} M) irradiated under O_2-free conditions with light at 820 nm for 90 minutes. Presence of m/z 513.29 and 511.3 as a proof of direct release from carbonate photocages in water media.

Irradiation Experiments Followed by NMR Spectroscopy

In the following figures, the green boxes highlight the peaks of the product **8** corresponding to the carbocationic intermediate trapped by the CD_3OD, whereas the red boxes highlight the uncaged payloads.

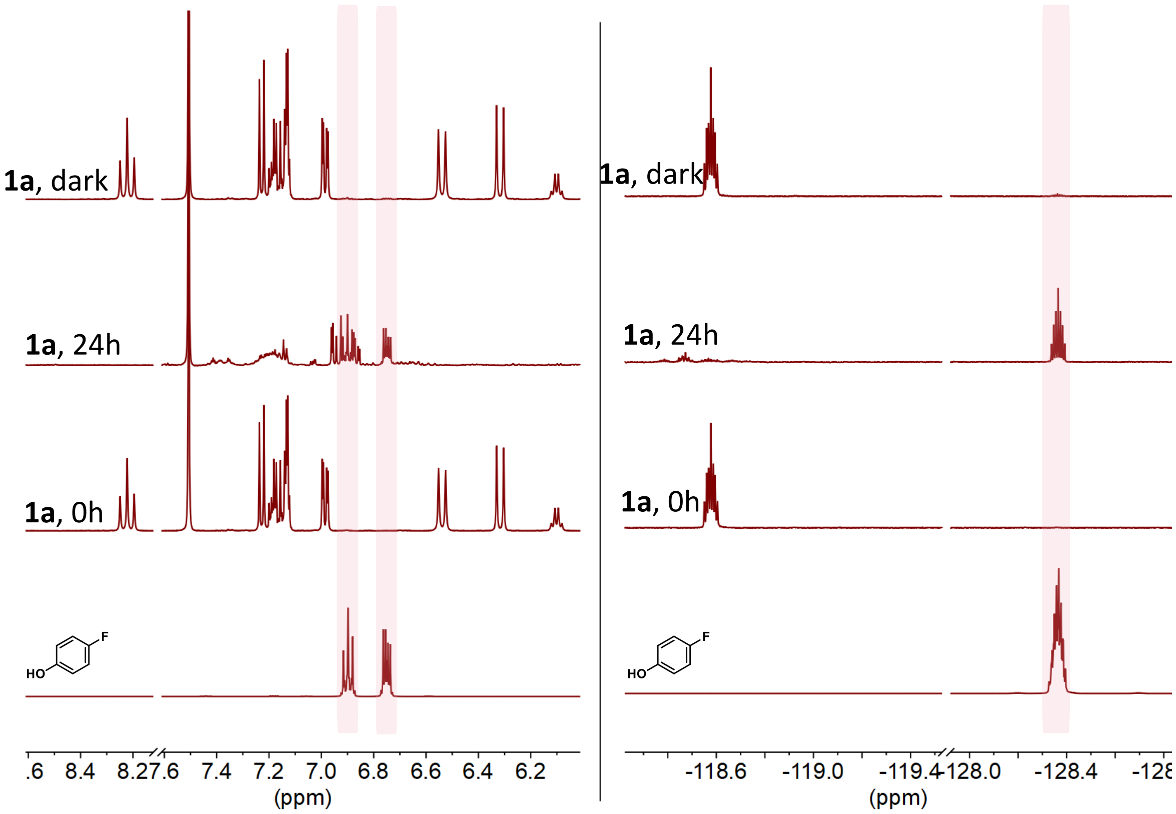

Figure S27. ^{1}H NMR (left) and ^{19}F NMR (right) spectra of a photocage **1a** irradiated with 820 nm LEDs under ambient conditions and the reference spectra.

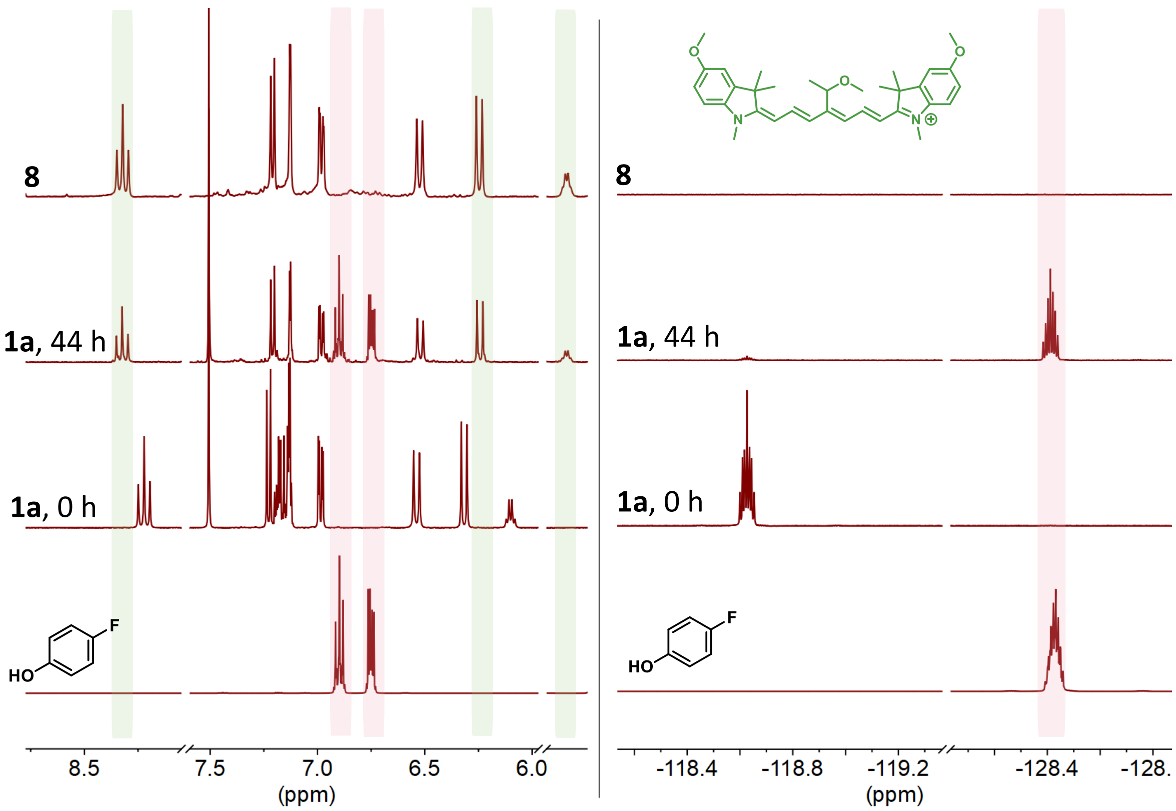

Figure S28. ^{1}H NMR (left) and ^{19}F NMR (right) spectra of a photocage **1a** irradiated with 810 nm LEDs under O_2–free conditions and the reference spectra.

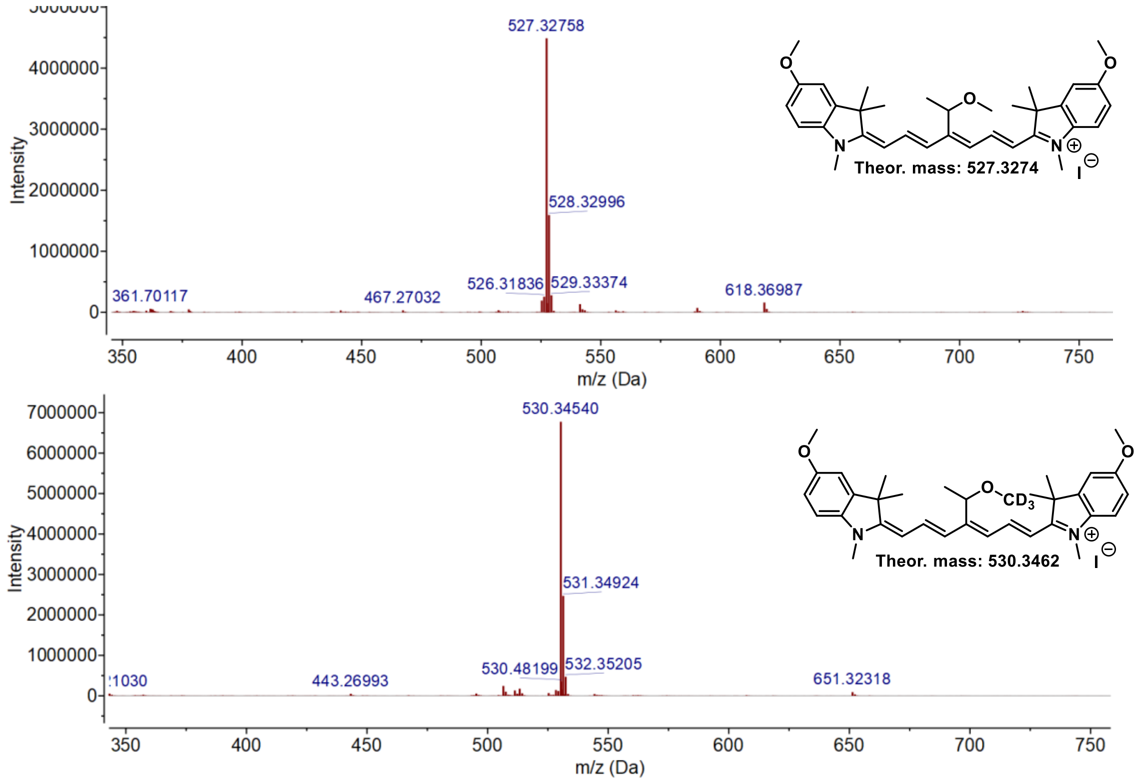

Figure S29. (top) ESI-HRMS spectra of the independently synthesized product of cation-trapping **8** and (bottom) photocage **1a** irradiated in CD_4OD with 810 nm light in O_2–free conditions for 44 h, the same sample as in Figure S28.

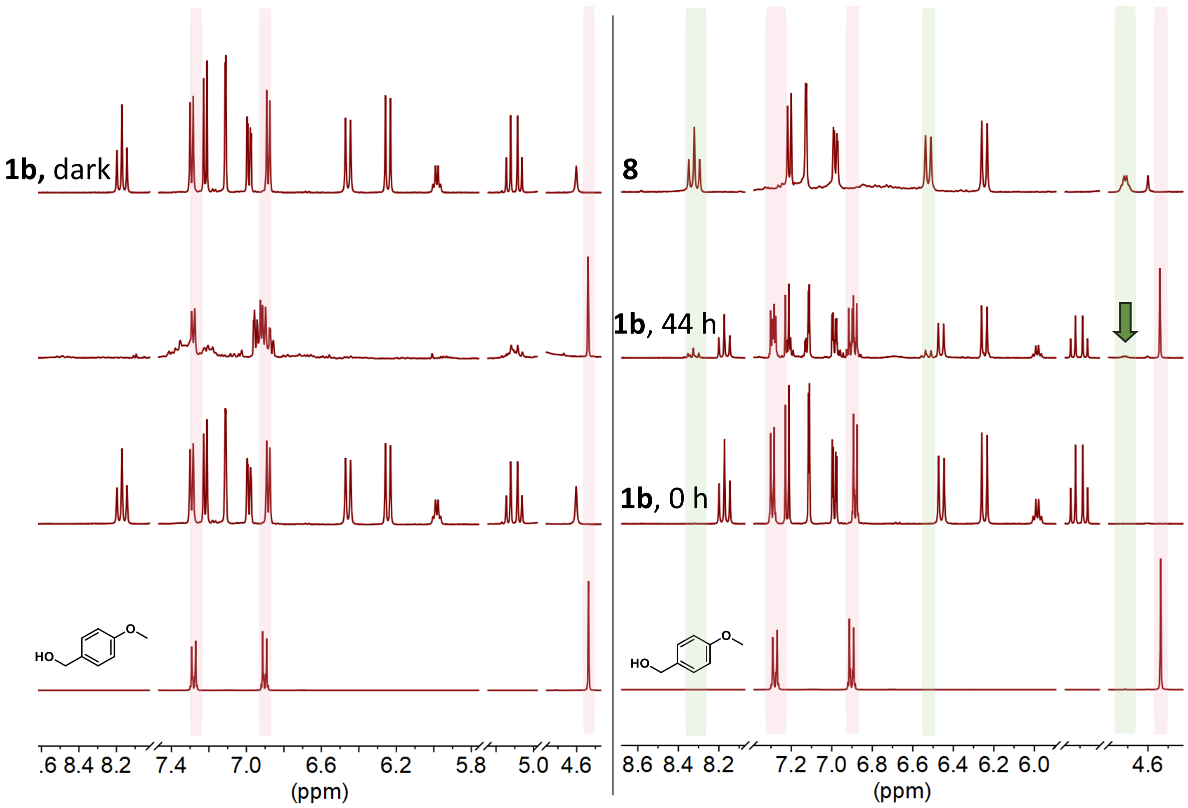

Figure S30. ^{1}H NMR spectra of photocage **1b** irradiated with 820 nm LEDs under ambient (left) and 810 nm under O_2–free conditions (right) measured at indicated time intervals.

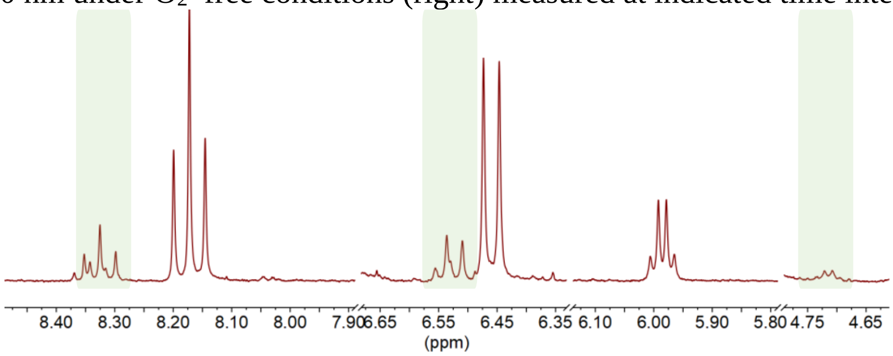

Figure S31. The same NMR spectra as in Figure S30, but zoomed in to show formation of the cyanine photoproduct **8**, and presumably other related cyanine photoproducts.

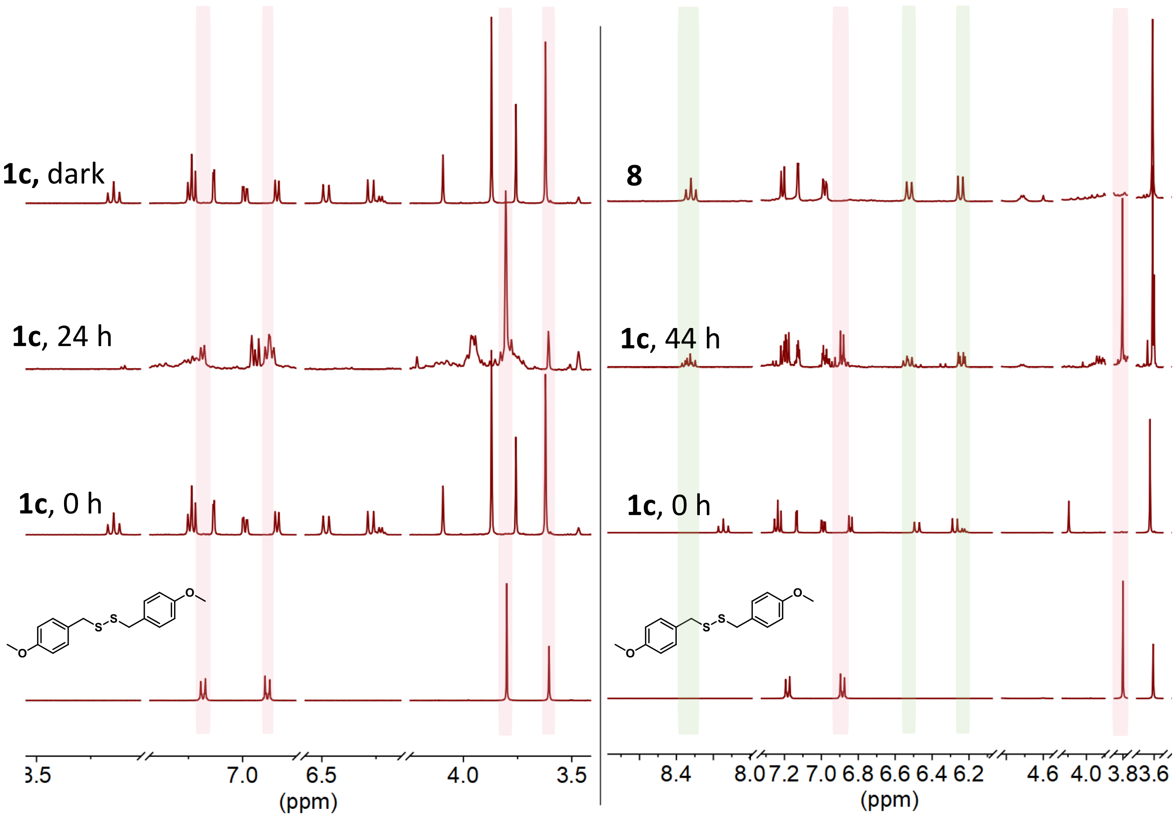

Figure S32. ^{1}H NMR spectra of photocage **1c** irradiated with 820 nm LEDs under ambient (left) and 810 nm under O_2–free conditions (right) measured at indicated time intervals. Thiol uncaged as dithiol.

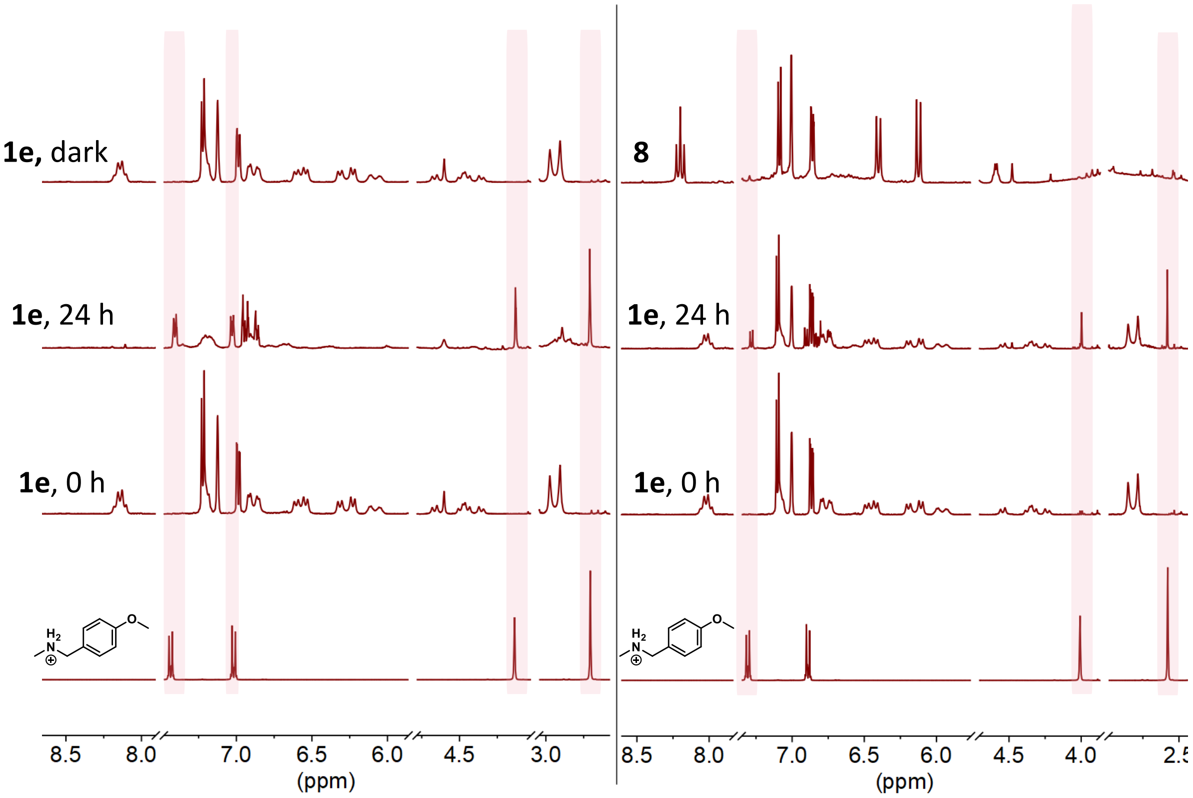

Figure S33. ^{1}H NMR spectra of photocage **1e** with irradiated 820 nm LEDs under ambient (left) and 810 nm under O_2–free conditions (right) measured at indicated time intervals.

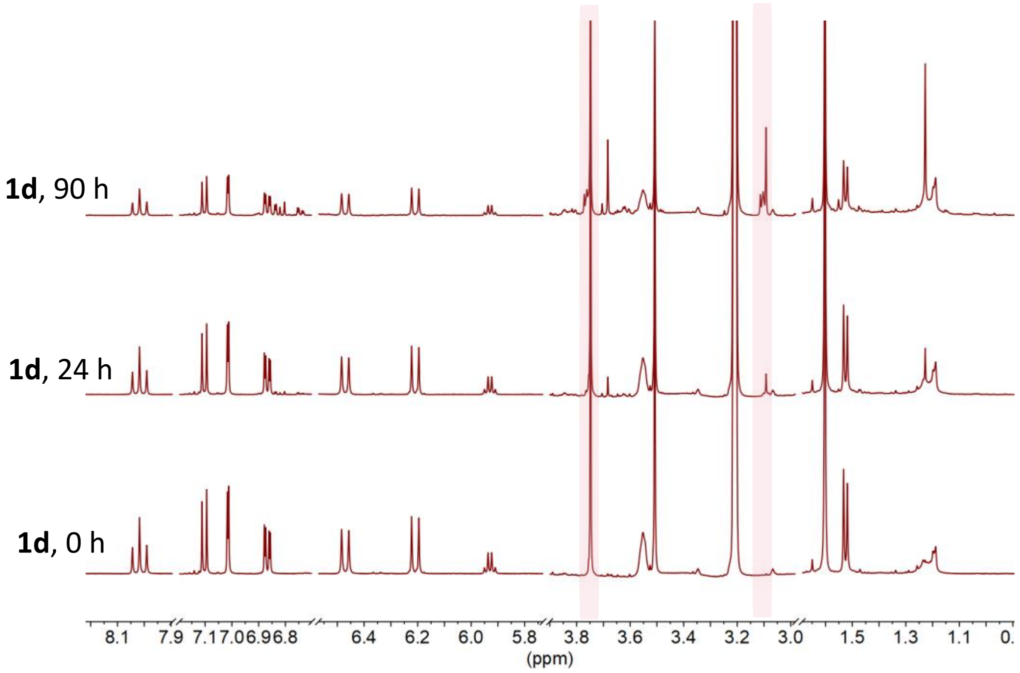

Figure S34. ^{1}H NMR spectra of photocage **1d** irradiated with 810 nm LEDs under O_2-free conditions measured after indicated time intervals. Red rectangle highlights the multiplets corresponding to the uncaging of morpholine.

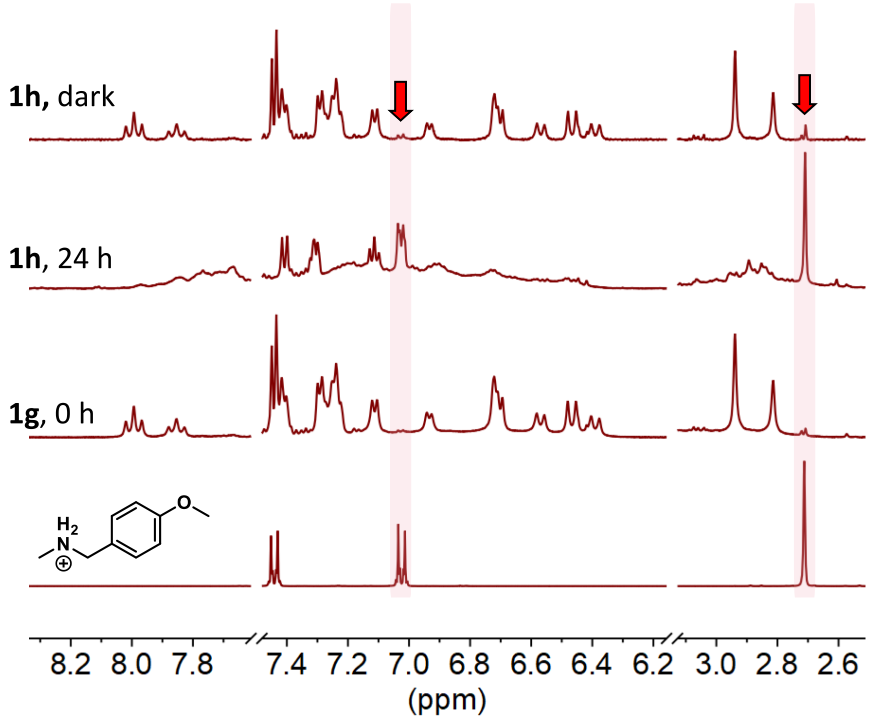

Figure S35. ^{1}H NMR spectra of photocage **1h** irradiated with 820 nm LEDs under ambient conditions measured at indicated time intervals. The red arrow highlights uncaging also in the control sample kept in the dark (compromised solvolytic stability).

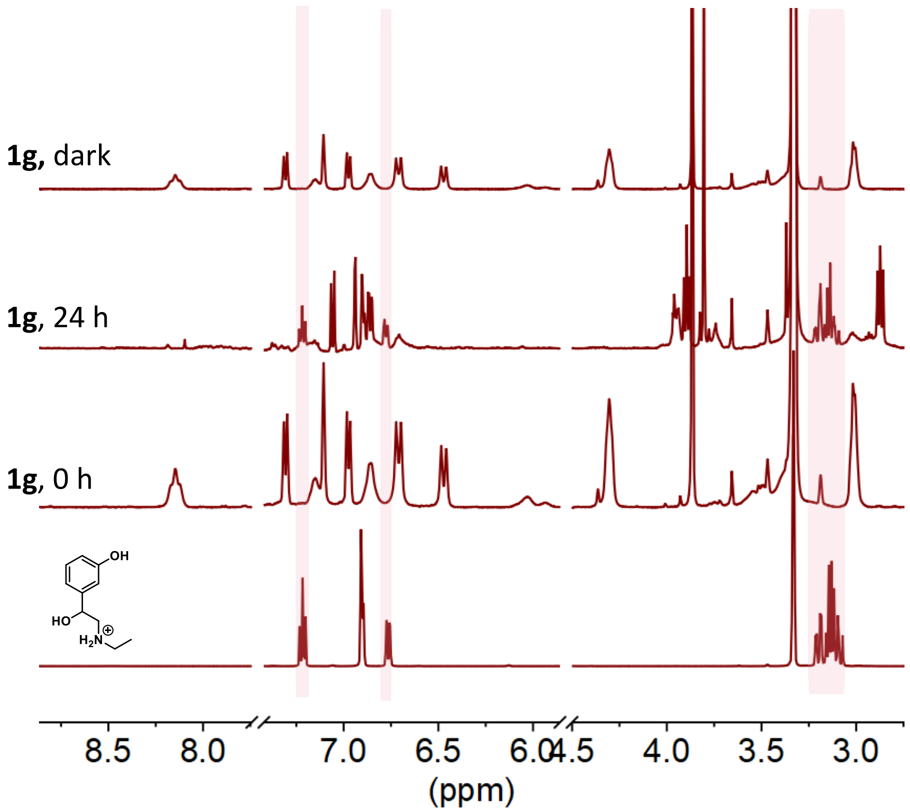

Figure S36. ^{1}H NMR spectra of photocage **1g** irradiated with 820 nm LEDs under ambient conditions measured at indicated time intervals.

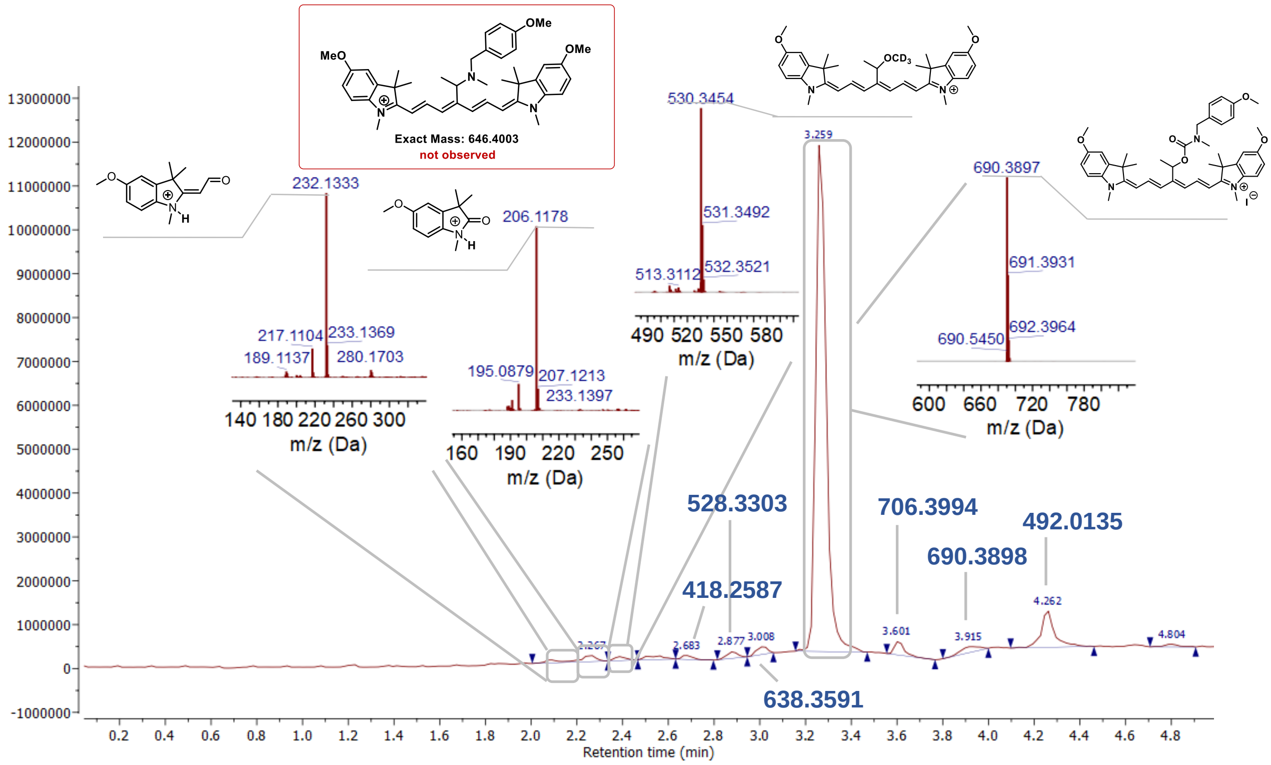

Figure S37. Total Ion Count (TIC, extracted from LC-HRMS) chromatogram of photocage **1e** in CD_3OD (1.80 mg in 0.6 mL) irradiated under O_2–free conditions at 810 nm for 90 hours. The process is accompanied by small amount of photooxidation products due to the presence of residual oxygen. No evidence of the putative cation trapped by amine payload is observed.

Effect of Increased Ionic Strength on Uncaging

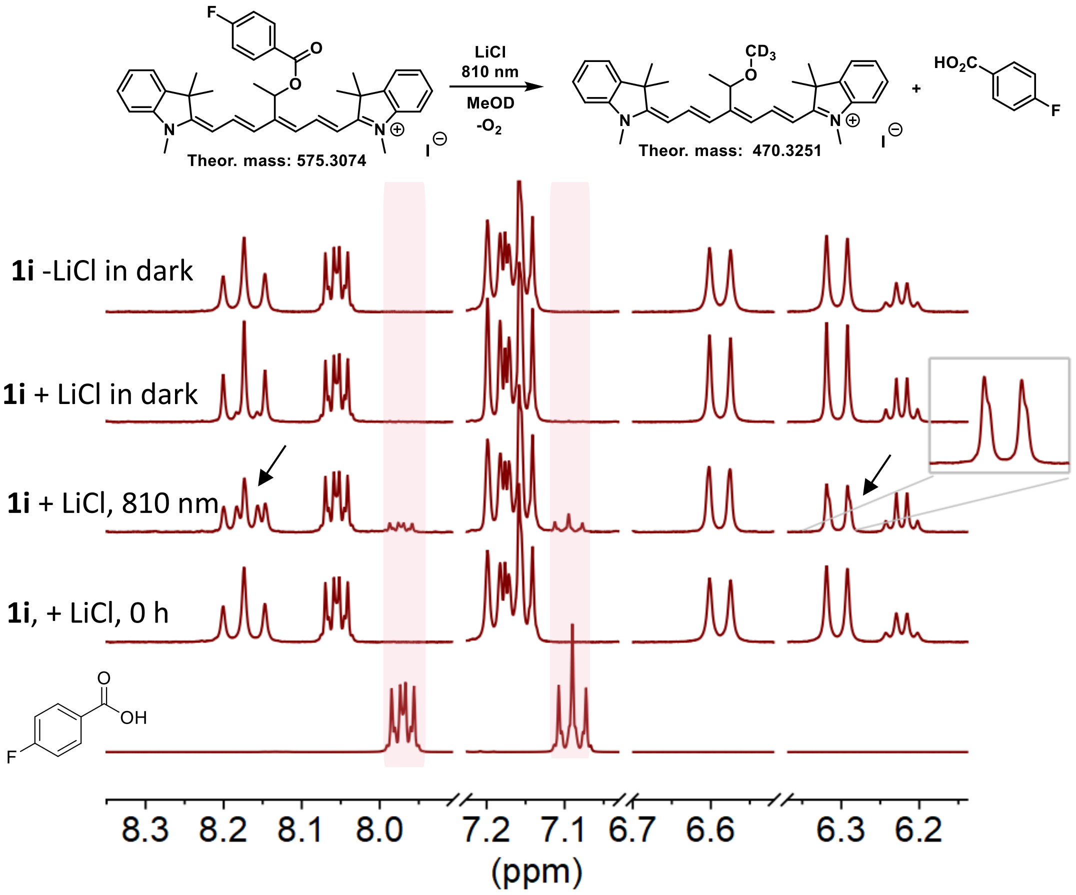

Figure S38. ^{1}H NMR spectrum of photocage **1i** bearing carboxylate cargo irradiated with light at 810 nm for 46 h under O_2–free conditions in CD_4OD containing LiCl (100 mM). The arrows denote the formation of the second cyanine species.

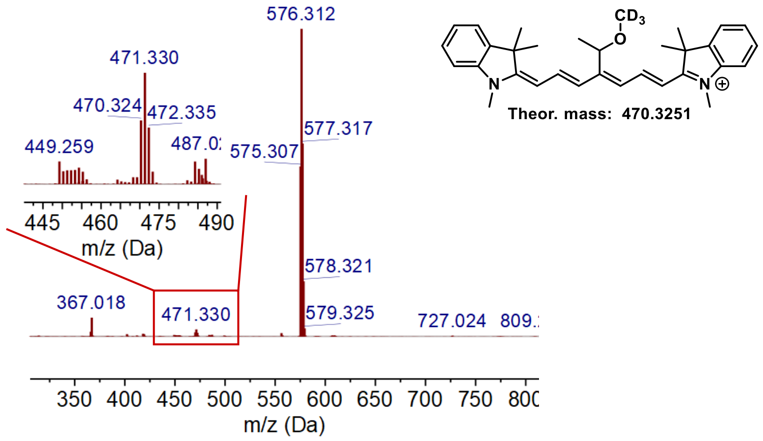

Figure S39. ESI-HRMS analysis of the sample from Figure S103.

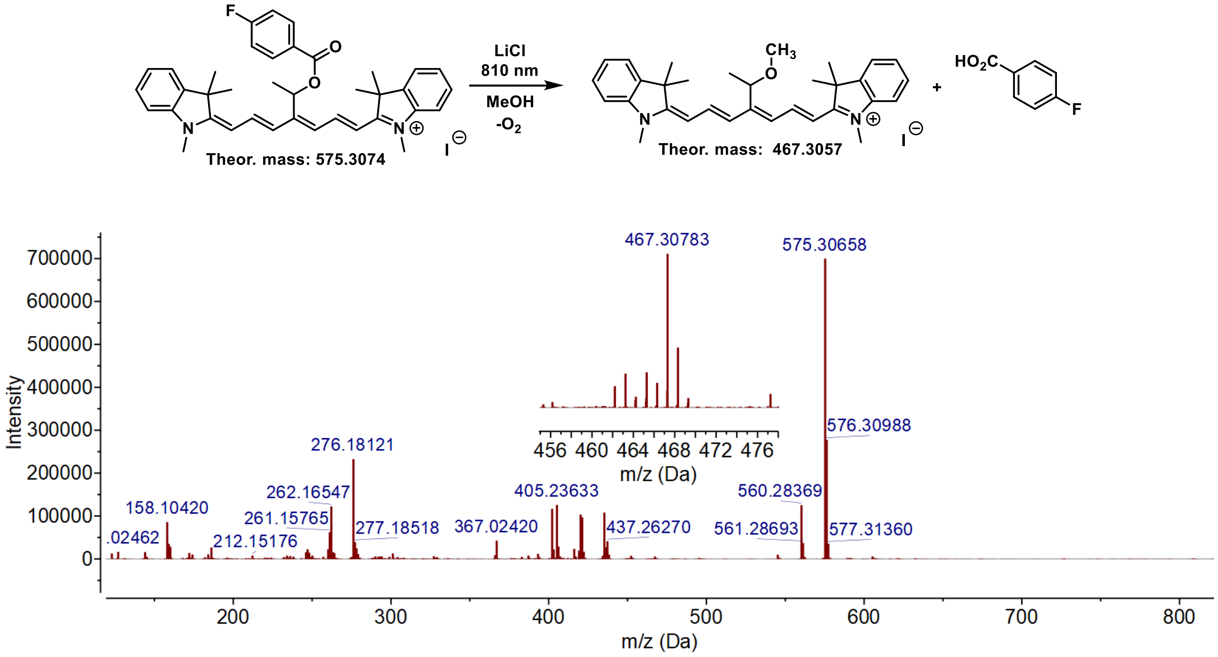

Figure S40. ESI-HRMS spectrum of photocage **1i** bearing carboxylate cargo irradiated with light at 810 nm under O_2–free conditions in regular CH_3OH containing LiCl (100 mM).

Irradiation of 1a or 1e in MeOH/H_2O Followed by HRMS

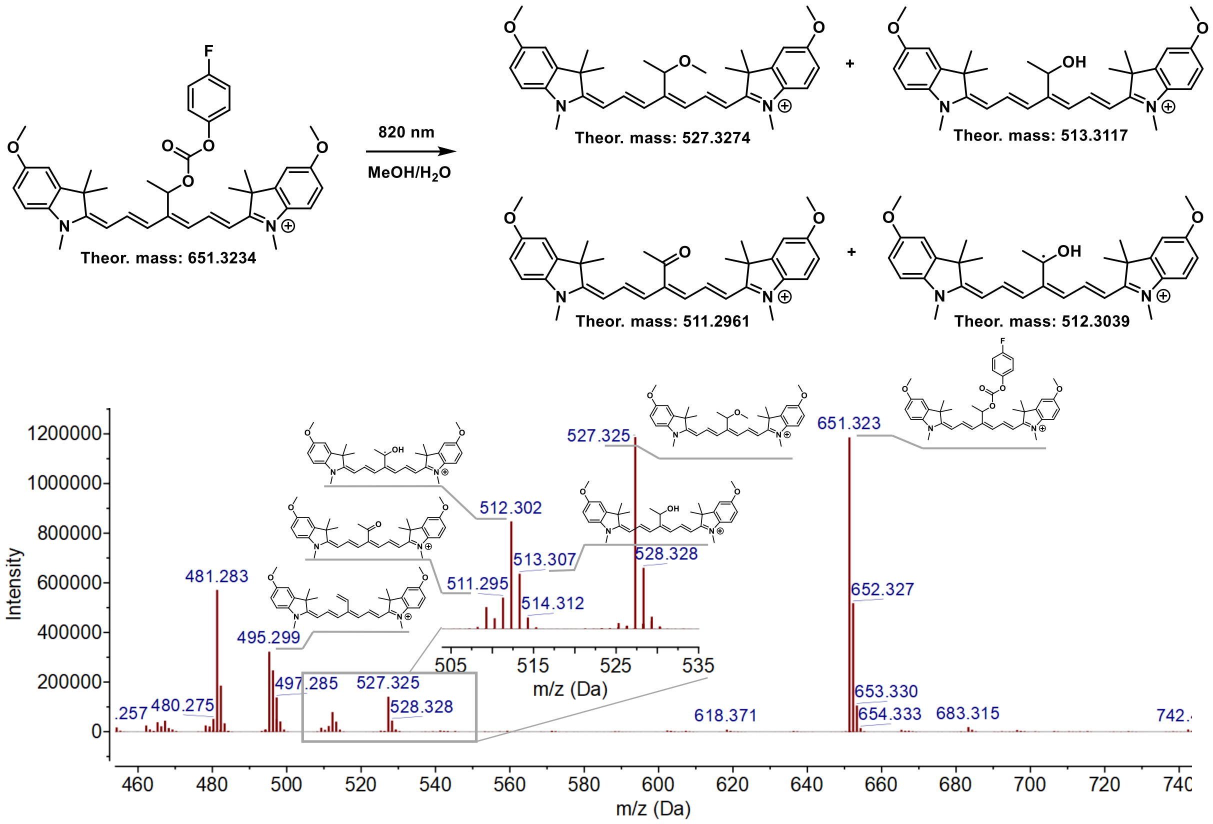

Figure S41. ESI-HRMS spectrum of photocage **1a** in MeOH/H_2O (1:1, 600 µg in 3 mL) irradiated under ambient conditions with light at 820 nm for 40 minutes.

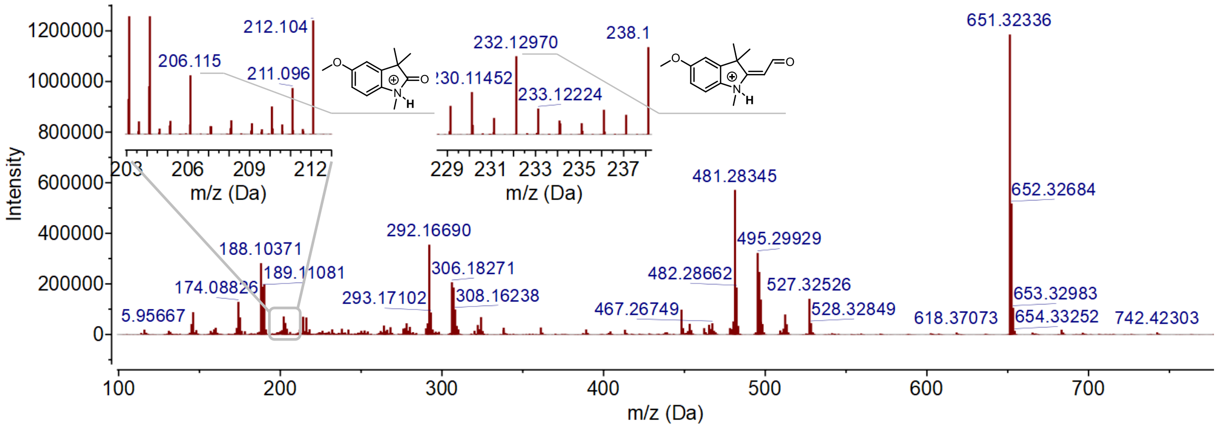

Figure S42. ESI-HRMS (including the low m/z section) spectrum of photocage **1a** in MeOH/H_2O (1:1, 600 µg in 3 mL) irradiated under ambient conditions with light at 820 nm for 40 minutes (same sample as in Figure S109). The MS spectrum shows also evidence of the photooxidation products, demonstrating that both pathways take place simultaneously at ambient conditions and this concentration.

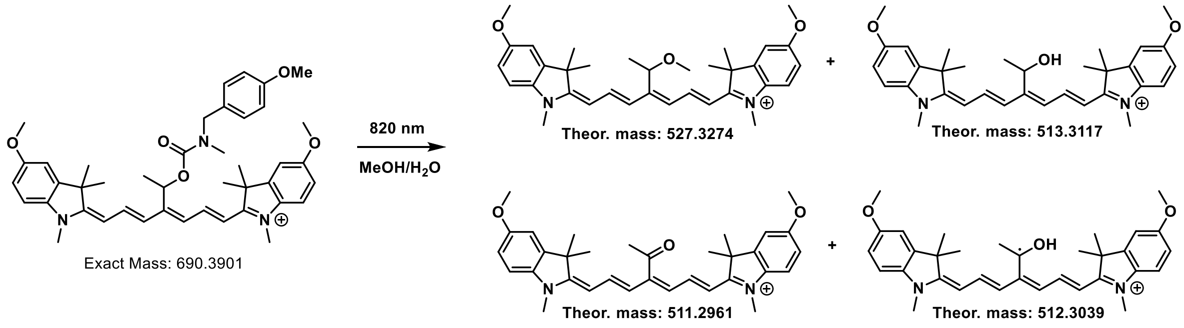

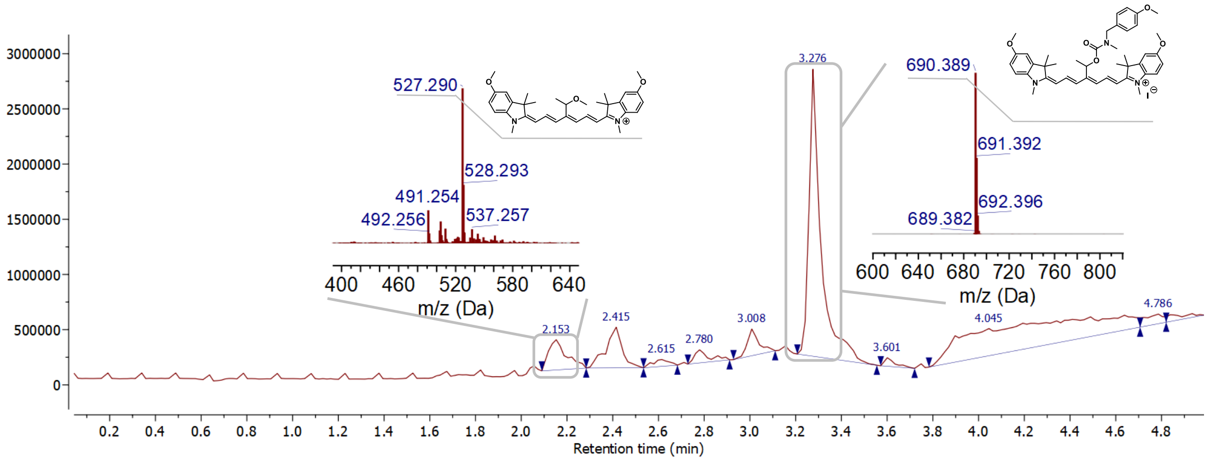

Figure S43. LC-HRMS spectrum of photocage **1e** in MeOH/H_2O (1:1, 600 µg in 3 mL) irradiated under ambient conditions with light at 820 nm for 40 minutes. Presence of m/z 527.29 as a proof of direct release from carbamate photocages

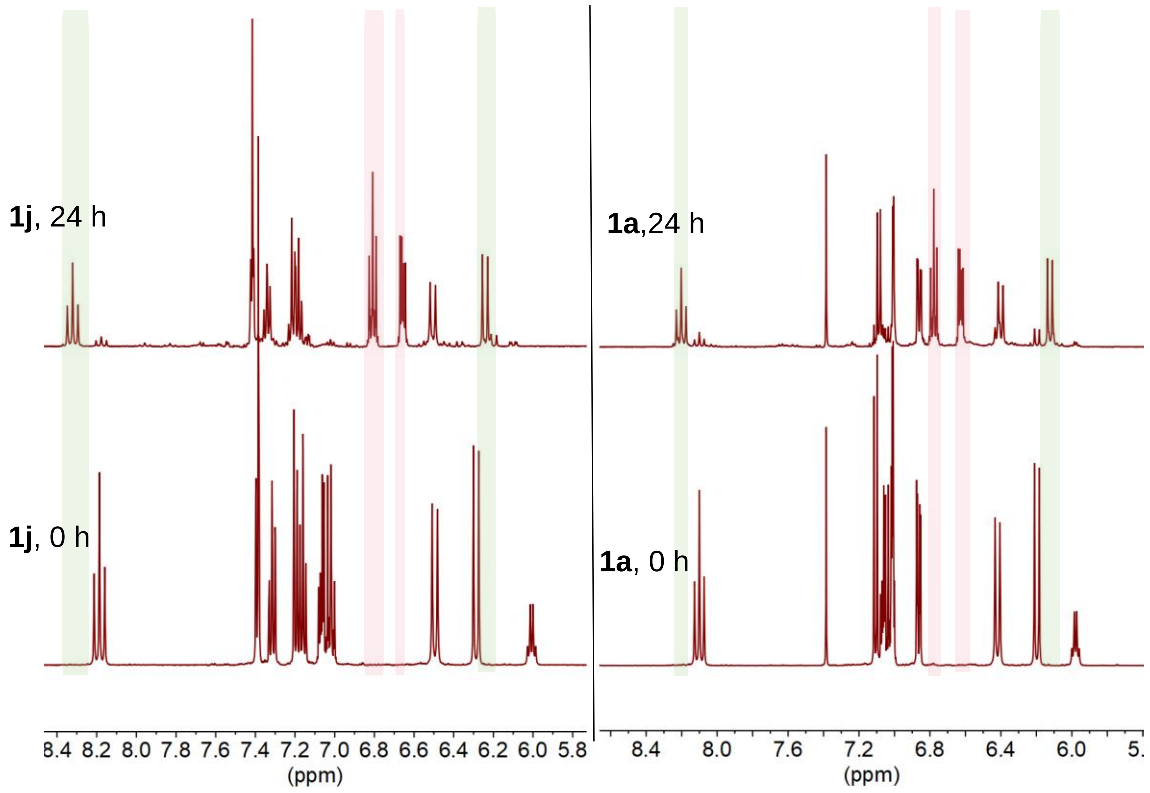

Figure S44. ^{1}H NMR spectra of photocage **1j** irradiated under O_2–free conditions (left) and photocage **1a** irradiated under O_2–free conditions (right) with 810 nm measured at indicated time intervals. Uncaging of phenol (red) accompanied by formation of cyanine **8** or its *des*-Ome analogue (green). The irradiation resulted in conversion of 93 % for **1j** and 91 % for **1a** under identical conditions.

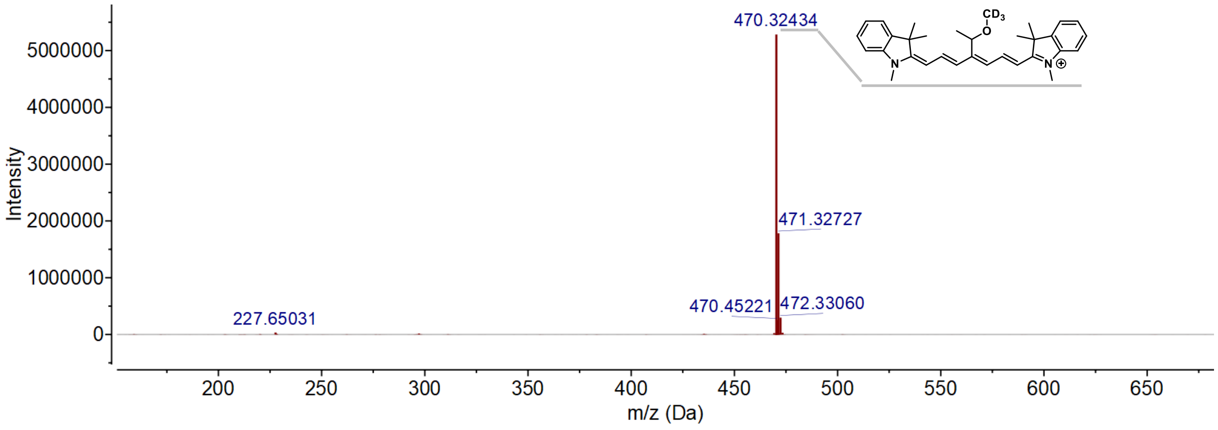

Figure S45. HRMS spectrum of photocage **1j** irradiated under O_2–free conditions with 810 nm LEDs for 24 h (analysis of the same sample from Figure S44).

Quantum Yield of Photooxidation Pathway

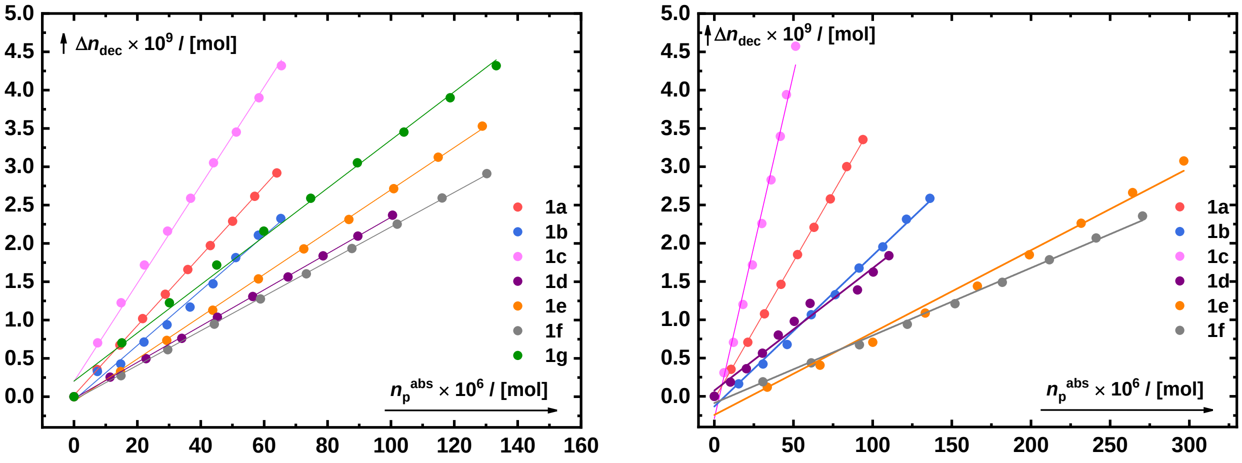

Figure S46. Quantum yield of decomposition of photocages **1a-g** in PBS (100 mM, 20% DMSO) under ambient conditions (left) and in sample purged by bubbling with Ar (right).

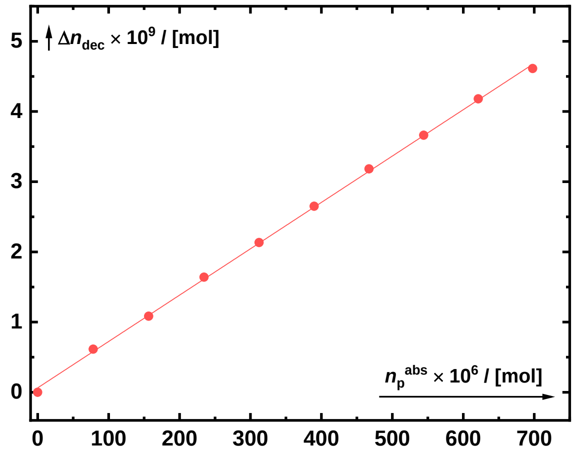

Figure S47. Quantum yield of decomposition of photocage **1h** in MeOH under ambient conditions.

Quatum Yield of the Direct Bond Scission Pathway

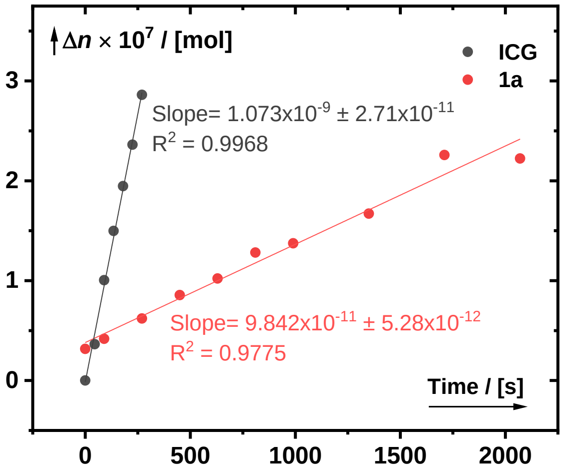

Figure S48. Quantum yield of release of fluorophenol from photocage **1a** in MeOH under O_2 free conditions. ICG used as a reference.

Summary of Quantum Yields

Table S1. Quantum yields of Cy7 degradation (Φ_{dec}), and quantum yields of uncaging ($\Phi_{uncaging}$) under ambient and in oxygen-free conditions in CD_3OD. Determined by ^1H NMR spectroscopy at ~4 mM concentrations of photocages.

Photocage	Φ_{dec} (ambient)	$\Phi_{uncaging}$ (ambient)	$\Phi_{uncaging}$ (oxygen-free)
**1a**	3.6×10^{-3} $(2\times10^{-6})^{a}$	1.8×10^{-3}	6.8×10^{-4}
**1e**	2.7×10^{-3}	9.3×10^{-4}	1.1×10^{-5}
**1i**	3.7×10^{-3}	$4.1\times10^{-3\ b}$	$<10^{-6\ c}$

[a]Determined by UV-vis spectroscopy at c ~5.0 µM, demonstrating concentration dependence of the photooxidation process.
[b]Number is overestimated due to a partial overlap of peaks corresponding to cargo with photooxidation side products.
[c]Cannot be observed, therefore we estimate the value to $<10^{-6}$.

Stability in Aqueous Media in Dark

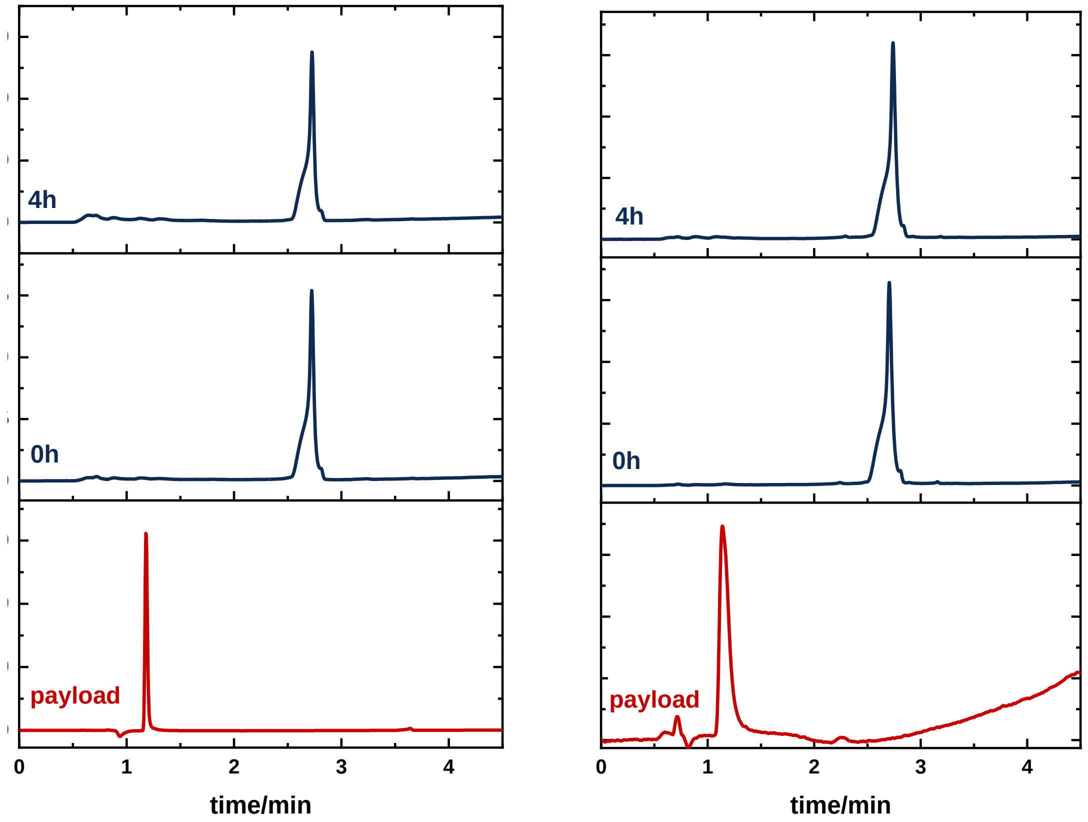

Figure S49. UHPLC chromatogram of photocage **1a** (left) and **1e** (right) (~80 µg/mL) incubated in aqueous media (10% MeCN in H_2O) in the dark for 4 h, and compared to the payload reference. Monitored at 278 and 240 nm, respectively. Samples show no release in the dark.

Irradiation Setups

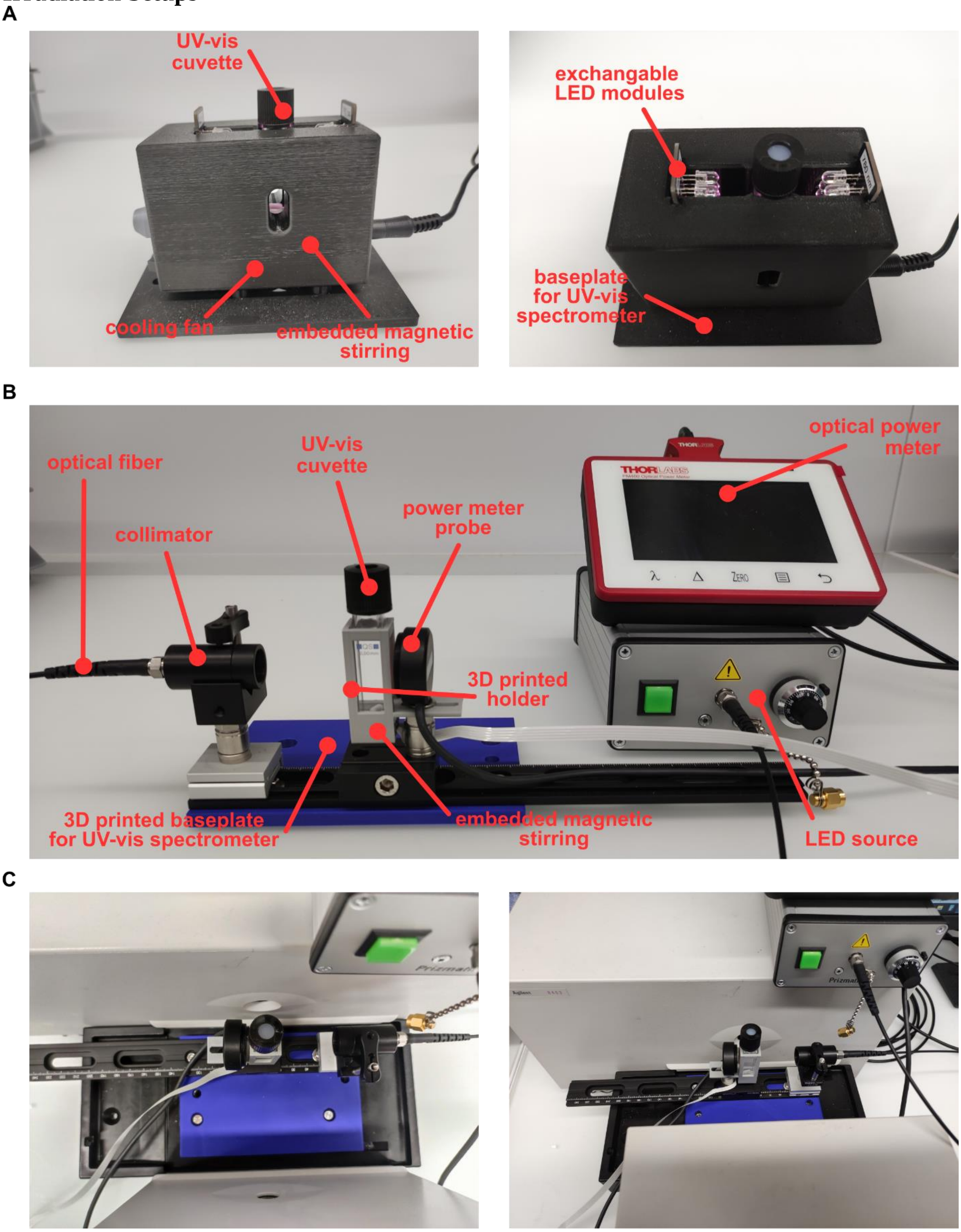

Figure S50. A) Irradiation module with magnetic stirring and exchangeable LED modules. B) In-house built irradiation setup with collimated light beam (780 nm) with magnetic stirring and coupled to optical power meter. C) Setup from (B) mounted inside a UV-vis spectrometer.

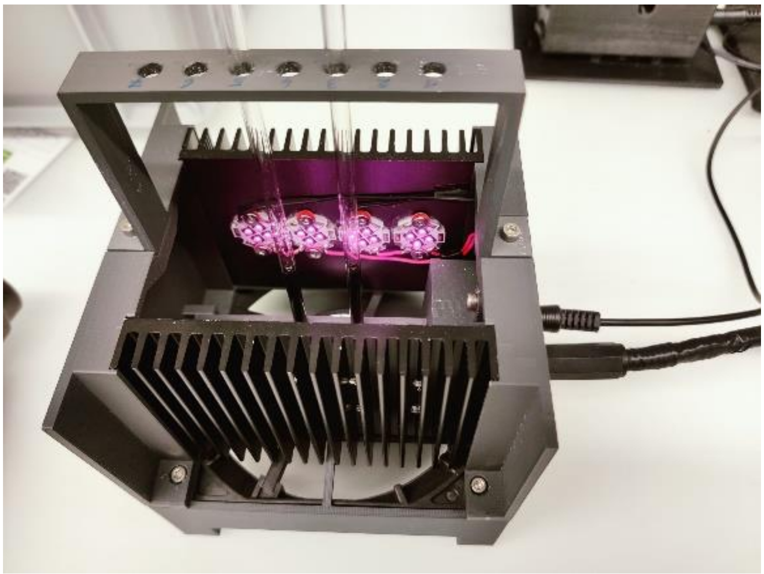

Figure S51. Home-made device for irradiation of NMR tubes (810 nm LEDs, ~300 mW cm^{-2}, at a fixed distance of ~3 cm, cooled by a fan at 1200 rpm).

Cell Viability Assays

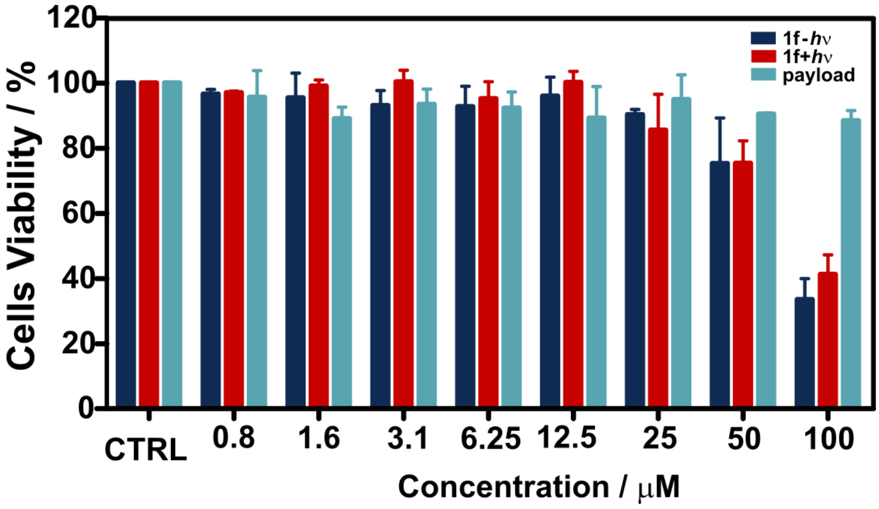

Figure S52. Cell-viability assay on HeLa cells in the presence of **1f** (blue), photoproducts of **1f** (red) and the free benzylamine payload (cyan). Average of three experiments and standard deviations of the mean are given.

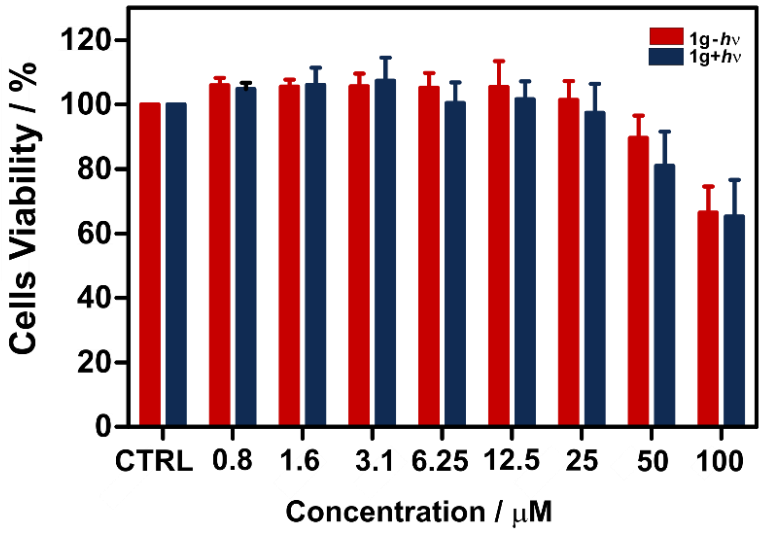

Figure S53. Cell-viability of HeLa cells in the presence of **1g** (blue), and photoproducts of **1g** (red). Average of two biological replicates and total of 15 technical replicates. Average and standard deviations of the mean are given.

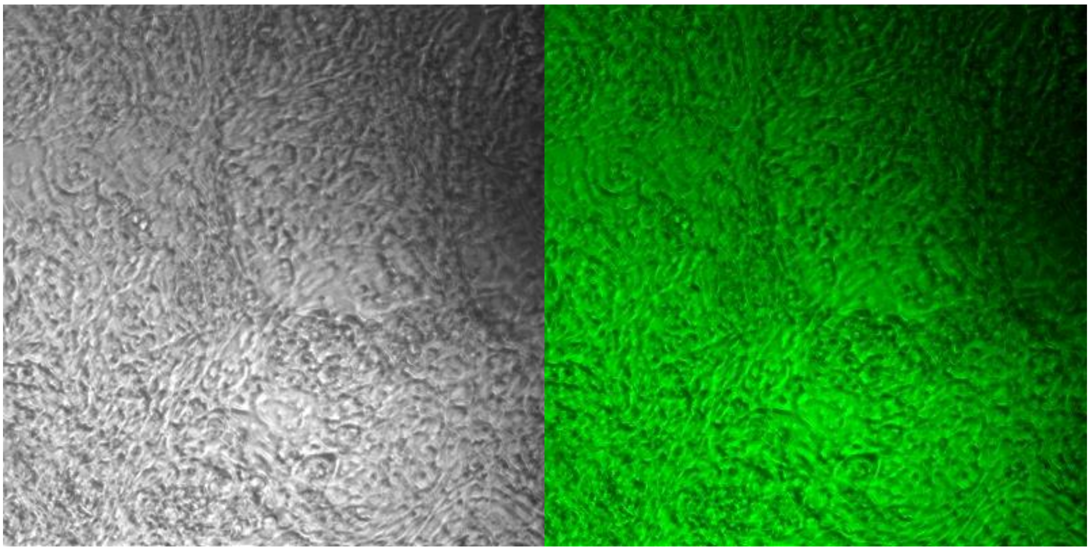

Figure S54. Representative images of the syncytium formed by iPSC-derived cardiomyocytes observed under light microscopy under 20× magnification (left) brightfield channel, (right) calcium probe Fluo-8AM in the green channel.

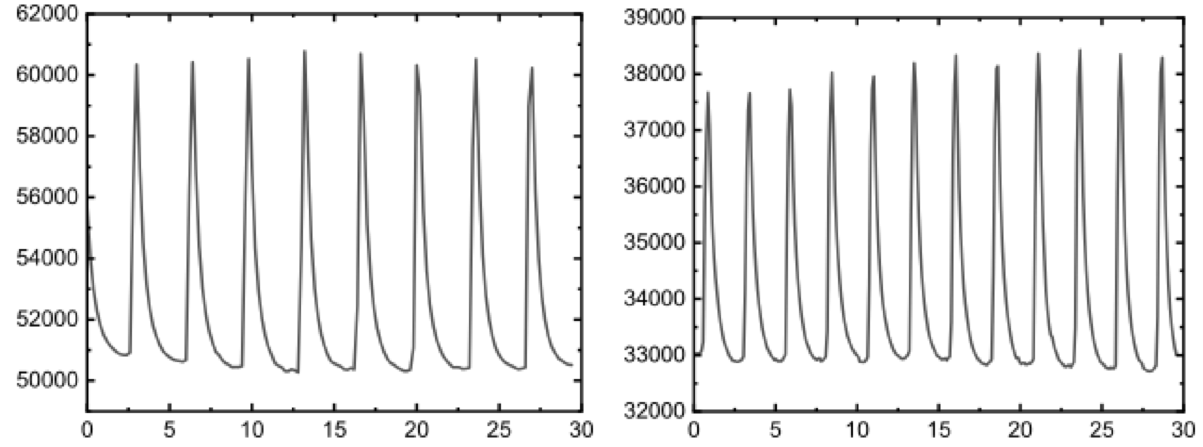

Figure S55. Representative traces from the calcium flux experiments imaged by Fluo-8AM in the beating iPSC-derived cardiomyocytes in the presence of **1g** in the dark (left) and irradiated for 5 min at 780 nm (right).

References

[1] Štacková, L.; Štacko, P.; Klán, P. *J. Am. Chem. Soc.* **2019**, *141*, 7155–7162.

[2] Štacková, L.; Russo, M.; Muchová, L.; Orel, V.; Vítek, L.; Štacko, P.; Klán, P. *Chem.Eur.J.* **2020**, *26*, 13184–13190.

[3] Liu, J.; Tang, W.; Sheng, L.; Du, Z.; Zhang, T.; Su, X.; Zhang, S. X.-A. *Chem. Asian J.*, **2019**, *14*, 438–445.

[4] C.H. Boehringer Sohn AG & Co. KG - US5422353 **1995**, A.

[5] Karimi, B.; Hazarkhani, H.; Zareyee D. *Synthesis* **2002**, *17*, 2513–2516.

[6] Janeková, H.; Russo, M.; Ziegler, U.; Štacko, P. *Angew. Chem. Int. Ed.* **2022**, e202204391.

[7] Štacková, L.; Muchová, L.; Russo, M.; Slavíček, P.; Štacko, P.; Klán, P. *J. Org. Chem.* **2020**, *15*, 9776–9790.

[8] Rodgers, M. A. J. *J. Am. Chem. Soc.* **1983**, 105, 6202–6205.

[9] Young, R. H.; Brewer, D.; Keller, R. A. *J. Am. Chem. Soc.***1973**, *95*, 375.

[10] Miura, K. *F1000Research*, **2020**, *9*, 1494.

[11]Wilkinson, F., Helman, W. P., Ross, A. B., *J. Phys. Chem. Ref. Data*, **1993**, 22, 1, 121
